# Metal-free visible-light-induced hydroxy-perfluoroalkylation of conjugated olefins using enamine catalyst†

**DOI:** 10.1039/d2ra06679c

**Published:** 2022-11-15

**Authors:** Koto Tagami, Yu Ofuji, Tadashi Kanbara, Tomoko Yajima

**Affiliations:** Department of Chemistry, Faculty of Science, Ochanomizu University Otsuka, Bunkyo-ku Tokyo 112-8610 Japan yajima.tomoko@ocha.ac.jp

## Abstract

We developed a simple and sustainable method for the hydroxy-perfluoroalkylation of electron-deficient conjugated olefins and styrenes. In this protcol, *in situ* generated enamine forms electron-donor–accepter (EDA) complexes with perfluoroalkyl iodide, and reaction proceed with visible-light irradiation. Tertiary amine also interacts with perfluoroalkyl iodide *via* halogen-bonding, promoting the perfluoroalkyl radical generation. This reaction does not require any transition-metal or photoredox catalyst, and gaseous oxygen is used as the green hydroxy source. Moreover, various commercially available substrates and perfluoroalkyl iodides were tolerated, affording the desired hydroxy-perfluoroalkylated products in good to moderate yields (>50 examples, up to 90%).

## Abstract 2

A sustainable, visible-light-induced hydroxy-perfluoroalkylation of electron-deficient olefins and styrenes catalyzed by ena mine were successfully developed.

## Graphical abstract

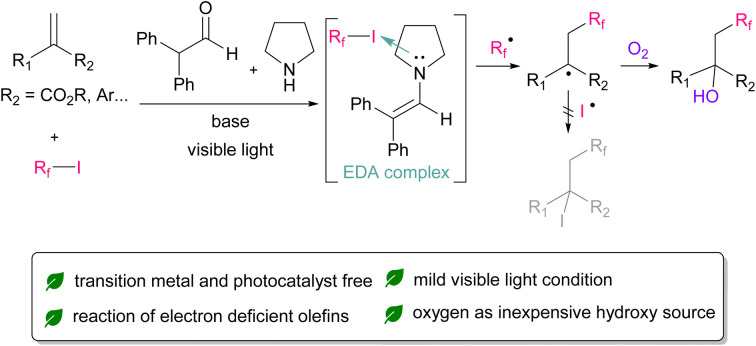

## Introduction

Fluorine atoms have unique properties, such as the third smallest van der Waals radius, the highest electronegativity, and a strong carbon–fluorine bond.^1^ Therefore, the introduction of fluorine into organic compounds has attracted attention from various fields, such as pharmaceuticals,^2^ agrochemicals,^3^ and functional materials,^4^ owing to the substantial property changes induced by the presence of fluorine. In addition to mono-fluorination reactions,^5^ a number of synthetic methods for fluoroalkylation have been established.^6^ In particular, radical protocols^7^ using commonly available perfluoroalkyl iodides as radical precursors have been widely explored.^8^ Furthermore, the reaction of simple terminated olefins is well established because of the high electrophilicity of perfluoroalkyl radicals.^9^ However, the handing of electron-deficient conjugated olefins remains challenging because they can easily self-polymerize and have low reactivity toward perfluoroalkyl radicals.^10^ Therefore, perfluoroalkylation reactions for electron-deficient olefins are in high demand.

**Scheme 1:**
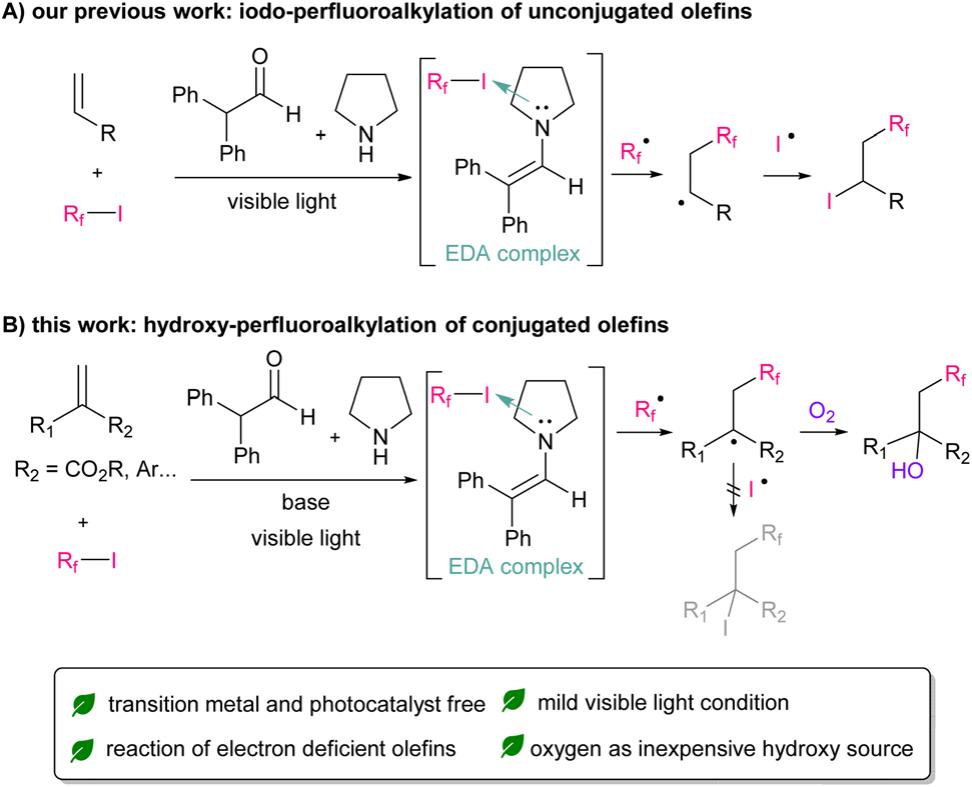
Perfluoroalkylation of olefins.

In recent years, reactions using photoredox catalysts under mild visible-light conditions have received significant attention owing to their sustainability.^11^ Thus far, various types of perfluoroalkylation reactions using valuable Ir or Ru catalysts have been reported.^12^ More recently, from the perspective of eco-friendliness and cost reduction, significant efforts have been devoted to develop metal-free photo-organocatalysed reactions. Research on the reaction using organic dye have been widely reported.^13^ Furthermore, simple molecules, such as enamine, amine, or phosphine could be used as an organocatalyst, which can form electron-donor–accepter (EDA) complexes with perfluoroalkyl iodide.^14^ Our group has also reported that *in situ* generated enamine can function as a photo-organocatalyst for the iodo-perfluoroalkylation of electron-rich unconjugated olefins (Scheme 1A).^14*f*^

Focusing on the significant potential of enamine as an organocatalyst, we applied our perfluoroalkylation reactions on electron-deficient conjugated olefins. As a result, iodo-perfluoroalkylation reaction did not proceed owing to the instability of iodide adduct product.^15^ Instead, we found that hydroxy-perfluoroalkylation proceeded in the presence of molecular oxygen and tertiary amine as organic base (Scheme 1B). Herein, we present the first example of the metal-free visible-light-induced hydroxy-perfluoroalkylation of electron-deficient conjugated olefins. This reaction could be applied to various perfluoroalkyl iodides, electron-deficient olefins, and styrenes.

## Results and discussion

We selected the reaction of ethyl methacrylate (1a) and 3.0 eq. of C_6_F_13_I (2a) to optimize the reaction conditions (Table 1). In our initial attempt, 10 mol% of diphenylacetaldehyde (3),^14*f*^ 40 mol% of pyrrolidine (4), and 0.8 eq. of oxygen in 1,2-dichoro ethane (DCE) with white light-emitting-diode (LED) irradiation at 25 °C external temperature for 3 h afforded the desired hydroxy-perfluoroalkylated product 5aa with only 3% yield (entry 1). We hypothesized that the iodine ions generated in the system would inactivate and stop the catalytic cycle of enamine. Therefore, we increased the equivalent of 4 in hopes that 4 would capture iodine,^14*f*^ but the yield did not improve (entry 2). In this case, an amide type by-product was observed in which 4 reacted with 2 and oxygen (Scheme S2†).^16^ Therefore, we considered that the use of excess amount of secondary amine is unsuitable because it was consumed as a by-product before it captured iodine. Afterwards, we added 1.0 eq. of *N*,*N*-diisopropylethylamine (DIPEA), which is tertiary amine and have no possibility to produce amide type by-product, instead of 4 and found that the yield improved to 37% (entry 3). Moreover, using 2.0 eq. of DIPEA further increased the yield to 53% (entry 4). From these results, we considered the possibility of generating perfluoroalkyl radicals *via* halogen-bond interaction between DIPEA and 2a same as previously reported.^17^ Therefore, experiments without 3, 4, or both 3 and 4 were conducted to confirm the necessity of the enamine catalyst (entries 5–7). In all cases, 5aa was obtained in 34–44% yield, suggesting that DIPEA also promote this reaction. However, the yields of entries 3–5 decreased compared to that of entry 2, and it indicated that both enamine and DIPEA were necessary for satisfactory yields. Additionally, replacing the enamine catalyst with 10 mol% of EY–2Na, which is known to be an effective photoredox organocatalyst for radical perfluoroalkylations,^13*d*,*g*^ decreased the yield to 31% (entry 8). This indicates that the photoredox catalyst is incompatible with the reaction in the presence of oxygen, which emphasizes the efficacy of the enamine catalyst in this system. Subsequently, we screened several tertiary amines without enamine (entries 9–11). As a result, using of triethylamine (TEA) or 1,8-diazabicyclo[5.4.0]undec-7-ene (DBU) decreased the yield to 9–14%, and reaction did not proceed in the presence of 1,4-diazabicyclo[2.2.2]octane (DABCO). Next, we optimized the reaction time using enamine and DIPEA, and it was found that 24 h of irradiation generated product 5aa with 75% yield (entries 12–13). In addition, the oxygen equivalent was investigated. Increasing the oxygen equivalent to 1.0–2.0 eq., considerable amounts of oxygen-derived amide-type by-products were obtained,^16^ and the product yields decreased to 30–50% yields (entries 14 and 15, Table S1†). However, reducing the amount of oxygen to 0.5 eq. led to the oligomerization of 1a, and the yield decreased to 58% (entry 16). Therefore, 0.8 eq. of oxygen is the optimum amount for this reaction. Moreover, the reaction without oxygen yielded trace amounts of the desired product, and oligomerization of 1a proceeded instead of iodo-perfluoroalkyation (entry 17). Also, the reaction in air produced a low yield of 38% (entry 18). Finally, the reaction under the dark at 80 °C was conducted and found that thermal radical generation is not a main route (entry 19). Furthermore, we confirmed that the reaction also proceeds by using pre-synthesized enamine (Table S3†).

**Table 1:** Optimization of the reaction conditions^a^

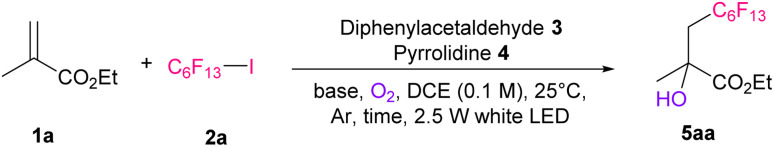						
Entry	3 (mol%)	4 (mol%)	Base (eq.)	O_2_ (eq.)	Time (h)	Yield (%)^b^
1	10	40	—	0.8	3	3
2	10	140	—	0.8	3	4
3	10	40	DIPEA (1.0)	0.8	3	37
4	10	40	DIPEA (2.0)	0.8	3	53
5	—	—	DIPEA (2.0)	0.8	3	34
6	10	—	DIPEA (2.0)	0.8	3	35
7	—	40	DIPEA (2.0)	0.8	3	44
8^c^	—	—	DIPEA (2.0)	0.8	3	31
9	—	—	TEA (2.0)	0.8	3	14
10	—	—	DBU (2.0)	0.8	3	9
11	—	—	DABCO (2.0)	0.8	3	n.r
12	10	40	DIPEA (2.0)	0.8	6	61
13	10	40	DIPEA (2.0)	0.8	24	75 (80)
14	10	40	DIPEA (2.0)	1.0	24	53
15	10	40	DIPEA (2.0)	2.0	24	30
16	10	40	DIPEA (2.0)	0.5	24	58
17	10	40	DIPEA (2.0)	—	24	Trace
18^d^	10	40	DIPEA (2.0)	—	24	38
19^e^	10	40	DIPEA (2.0)	0.8	24	10

a Reaction conditions: 1a (0.25 mmol), 2a (0.75 mmol, 3.0 eq.), 3 (0.025 mmol, 10 mol%), 4 (0.01 mmol, 40 mol%), DIPEA (0.5 mmol, 2.0 eq.), O_2_ (0.2 mmol, 0.8 eq.), DCE (2.5 mL), at 25 °C, in argon atmosphere, for 24 h, and under white LED irradiation.

b Yields based on ^19^F NMR spectroscopy using benzotrifluoride as an internal standard; isolated yields are given in parentheses.

c 10 mol% Eosin Y–2Na was used instead of 3 and 4.

d Under normal atmosphere.

e In the dark at 80 °C.

After determining the optimized conditions, *i.e.*, entry 13, we investigated the substrate scope of the electron-deficient conjugated olefins (Table 2). Significantly, the use of various methacrylates with different ester groups (ethyl, methyl, tertiary butyl, benzyl, phenyl, and cyclohexyl) afforded the corresponding hydroxy-perfluoroalkylated products 5aa–5fa in 69–81% yields. The reaction using benzyl methacrylate (1d) can be scaled up to 6.25 mmol, producing 1.22 g of 5da (Scheme S4†). In addition, the use of methacrylate, which has glycidyl, trifluoromethyl, isobornyl, menthyl, and 8-phenylmenthyl groups, resulted in good yields of the desired products 5ga–5ka, respectively. The reaction tolerance to amides was low and produced the corresponding products 5la–5na in 18–24% yields. Notably, the reaction of the substrate with camphorsultum (1l) produced 5la with a 95 : 5 diastereoselectivity. This is due to the high bulkiness of the camphorsultum group, which also gave high selectivity in our previous perfluoroalkylation reactions.^10*c*,*f*^ Thereafter, the reactions of more electron-deficient several acrylates (1o–1r) were examined and the corresponding products 5oa–5ra were obtained in 17–32% yields. When the yield was low (5la–5ra), considerable amounts of oxygen-derived by-product (Scheme S2†) was obtained. Furthermore, ethyl 2-phenethyl-propenoate (1s) and itaconic acid diesters (1t and 1u) produced the corresponding products 5sa–5ua in 36–64% yields.

**Table 2:** Substrate scope of the electron-deficient olefins^a^^,^^b^

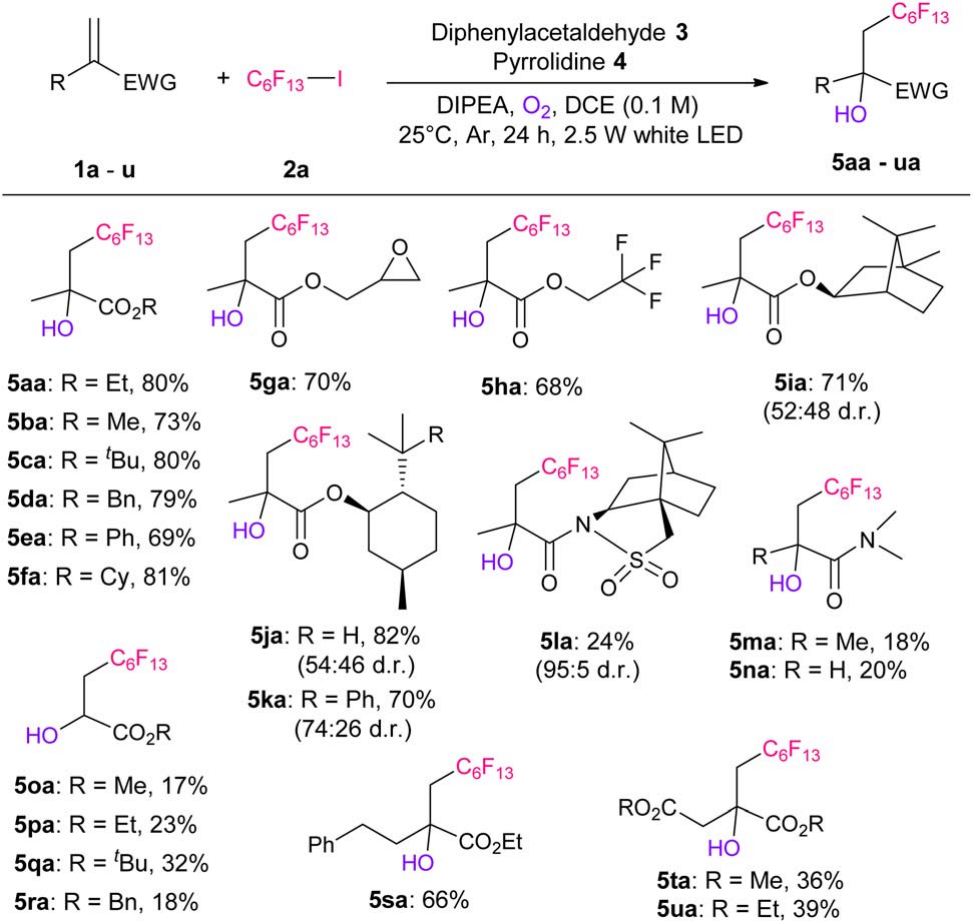

a Reaction conditions: 1 (0.25 mmol), 2a (0.75 mmol, 3.0 eq.), 3 (0.025 mmol, 10 mol%), 4 (0.01 mmol, 40 mol%), DIPEA (0.5 mmol, 2.0 eq.), O_2_ (0.2 mmol, 0.8 eq.), DCE (2.5 mL), at 25 °C, in argon atmosphere, for 24 h, and under white LED irradiation.

b Isolated yields.

Next, we investigated the scope of perfluoroalkyl iodides using 1a (Table 3). Initially, the corresponding hydroxy-perfluoroalkylated products 5aa–5ad were obtained in 76–80% yields. Perfluorobenzyl iodide (2e) can also be used for this reaction. Subsequently, we performed the same reactions using short-chain length perfluoroalkyl iodides (*n* < 5) and successfully obtained the desired products in good to moderate yields, which were measured using crude ^19^F NMR (Table S7†). However, because of their high volatility, these products are difficult to isolate *via* silica gel column chromatography. Therefore, we selected 1d as the substrate, which has a higher boiling point than 1a, and examined the scope of shorter chain length perfluoroalkyl iodides. The desired products 5df–5dj with short liner perfluoroalkylated groups (*n* = 1–5) were obtained in 71–76% yields. Likewise, bulkier perfluoroisopropyl iodide (2k) and perfluorocyclohexyl iodide (2l) were also tolerated, producing the corresponding products 5dk and 5dl in 64% and 27% yields, respectively. In addition, less nucleophiloc fluorine sources (2m and 2n) with a methylene group next to the iodine atom resulted in products 5dm and 5dn, respectively, in 20–27% yields.

**Table 3:** Screening of perfluoroalkyl iodides^a^^,^^b^

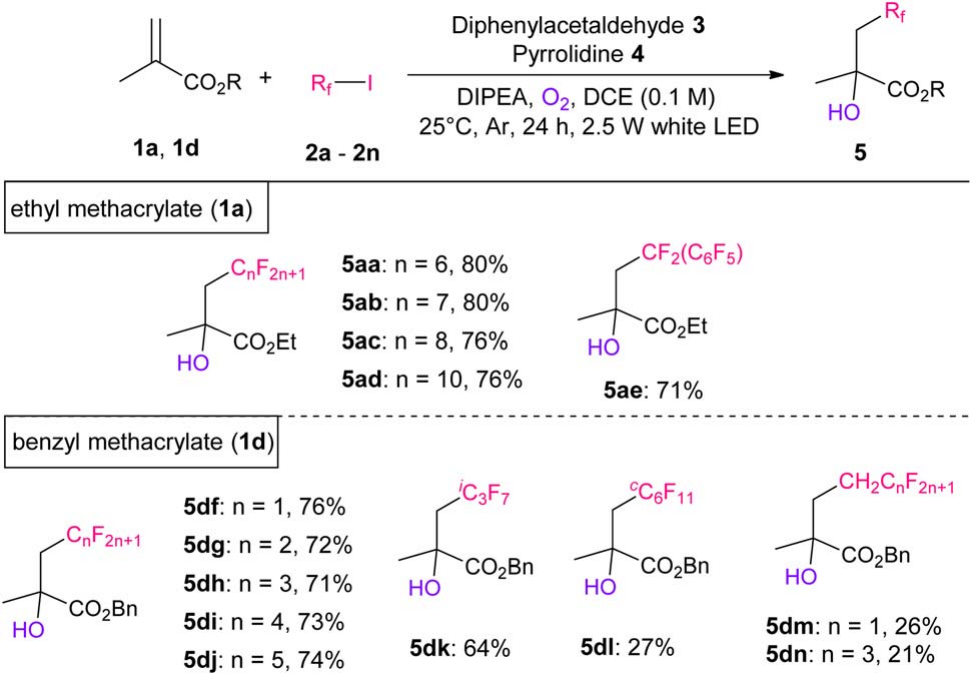

a Reaction conditions: 1 (0.25 mmol), 2 (0.75 mmol, 3.0 eq.), 3 (0.025 mmol, 10 mol%), 4 (0.01 mmol, 40 mol%), DIPEA (0.5 mmol, 2.0 eq.), O_2_ (0.2 mmol, 0.8 eq.), DCE (2.5 mL), at 25 °C, in argon atmosphere, for 24 h, and under white LED irradiation.

b Isolated yields.

We also applied our reactions to various styrenes (Table 4). First, a series of styrenes with electron-donating or electron-withdrawing groups at the *p*-position (6a–6h) were examined, affording the corresponding hydroxy-perfluoroalkylated products 7aa–7ha in excellent yields (74–89%). In the case of *p*-nitrostyrene, 6i afforded the desired product 7ia in a 39% yield. Furthermore, *o*- or *m*-substituted chlorostyrene (6j or 6k) and 2,3,4,5,6-pentafluorostyrene (6l) gave good yields of products 7ja–7la (63–80%). α-Methyl- or phenyl-substituted styrene (6m–6o) also gave hydroxy-perfluoroalkylated products 7ma–7oa in 69–90% yields. The reaction was then applied to naphthalene substrate 6p, and the desired product 7pa was obtained in 56% yield. In addition, 1,2-dihydronaphthalene (6q) and β-methylstyrene (6r) afforded the corresponding products 7qa and 7ra, respectively, in moderate yields and diastereo-selectivities.

**Table 4:** Substrate scope of styrenes^a^^,^^b^

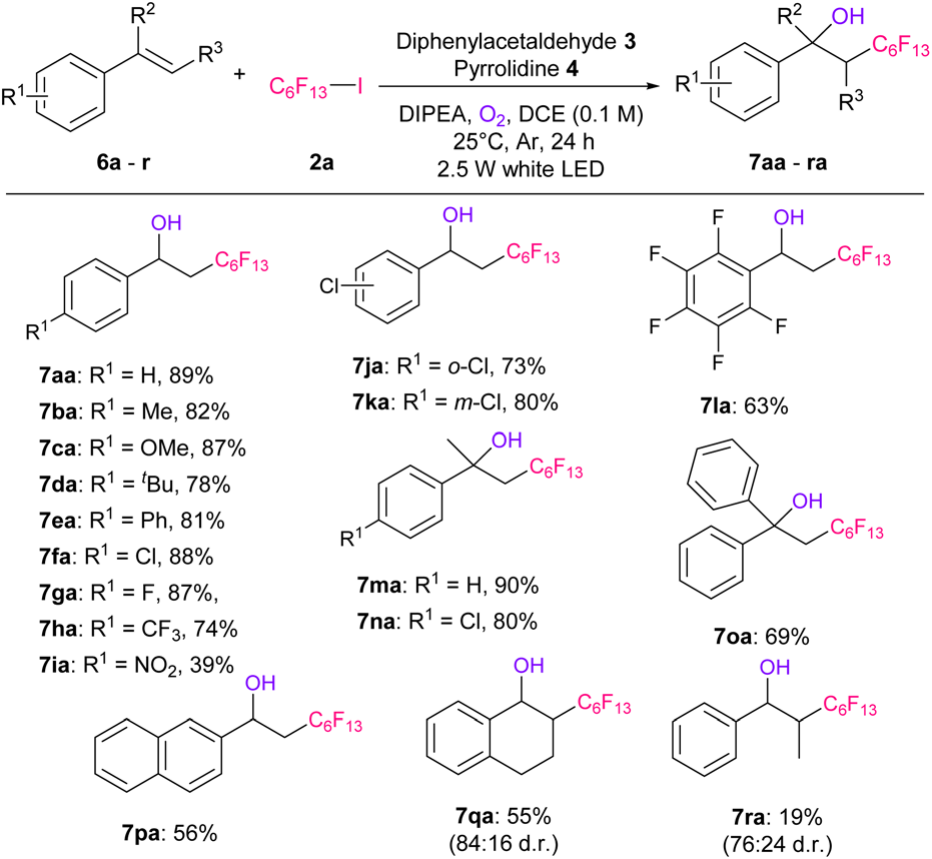

a Reaction conditions: 6 (0.25 mmol), 2a (0.75 mmol, 3.0 eq.), 3 (0.025 mmol, 10 mol%), 4 (0.01 mmol, 40 mol%), DIPEA (0.5 mmol, 2.0 eq.), O_2_ (0.2 mmol, 0.8 eq.), DCE (2.5 mL), at 25 °C, in argon atmosphere, for 24 h, and under white LED irradiation.

b Isolated yields.

**Scheme 2:**
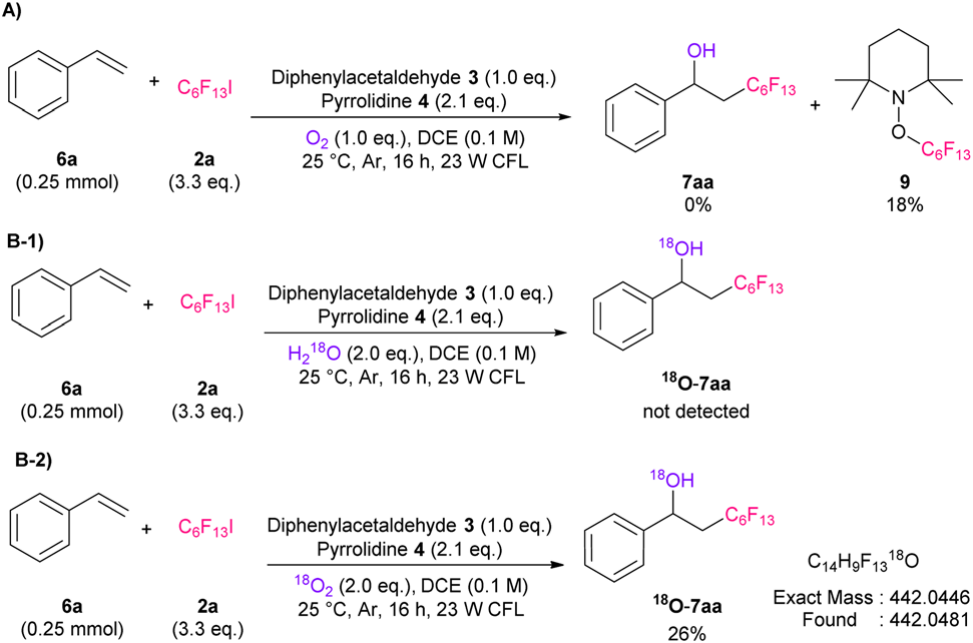
Control experiments. Yields based on ^19^F NMR spectroscopy using benzotrifluoride as an internal standard, CFL: compact fluorescent lamps.

To confirm the reaction mechanism, we carried our several control experiments. First, radical trapping experiment for 6a using (2,2,6,6-tetramethylpiperidin-1-yl)oxyl (TEMPO) as radical scavenger was performed (Scheme 2A). As a result, only TEMPO-perfluoroalkylated product 9 was observed, which indicates radical pathway is involved in this reaction. In addition, we conducted labeled experiments using H_2_^18^O or ^18^O_2_ (Scheme 2B). The results suggested that the hydroxy source of this reaction is molecular oxygen. Subsequently, ^19^F NMR titration experiment, determination of binding stoichiometry, and calculation of association constant (*K*_a_) between 2 and enamine or DIPEA were performed, respectively, to confirm the radical generation mechanism (Fig. S4–S9†).^17^ As a result, we found that *in situ* generated enamine would preferentially interact with 2 over DIPEA, and worked effectively as catalyst.

**Scheme 3:**
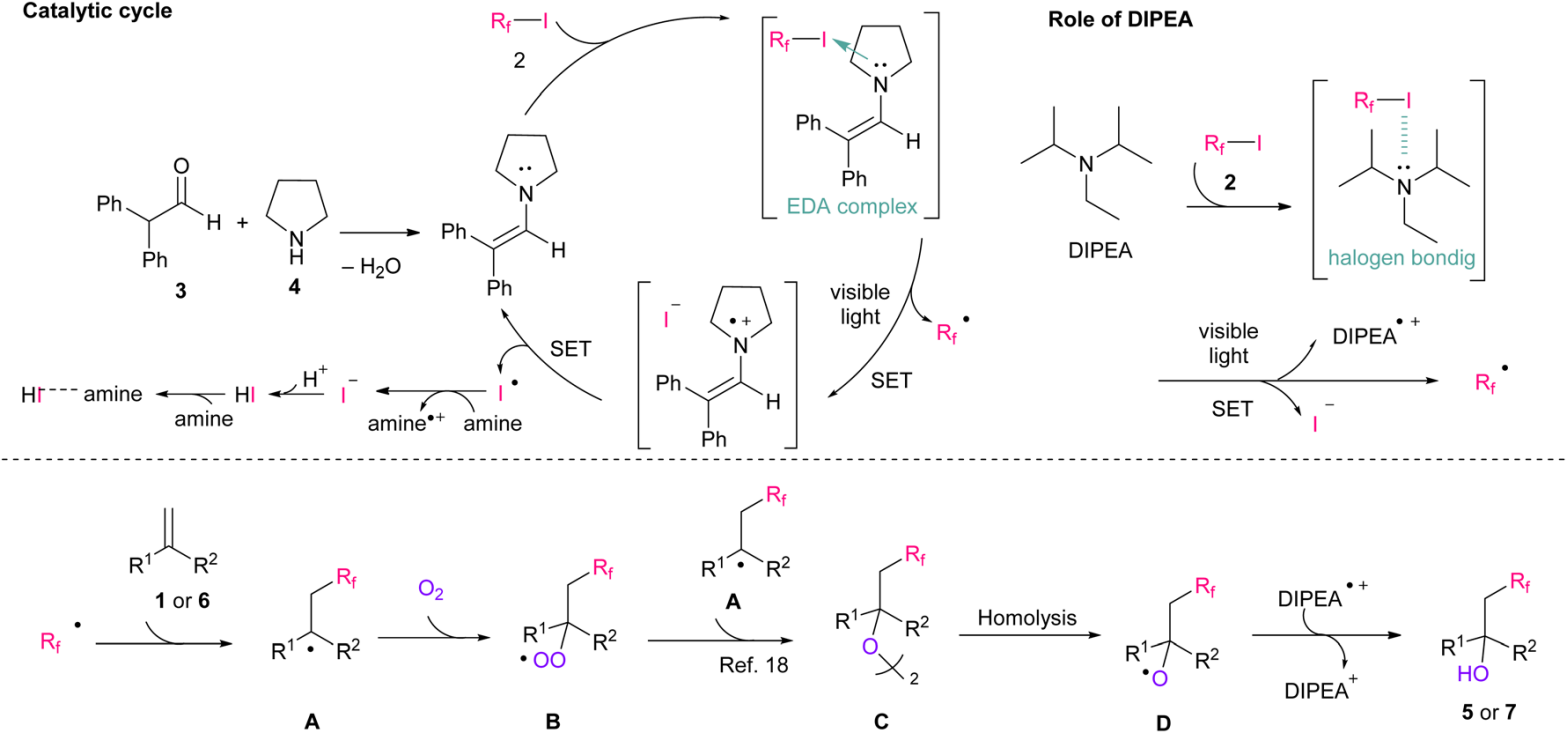
Proposed mechanism of hydroxy-perfluoroalkylation.

Based on our previous report^14*f*^ and other literature,^18^ we proposed a plausible reaction mechanism (Scheme 3). From the results of entries 4–7 in Table 1 and mechanistic studies, we proposed that both enamine and DIPEA are involved in the perfluoroalkyl radical formation. In a catalytic cycle, enamine was produced by condensation of 3 and 4; then, it generated an EDA complex with 2.^14*f*^ The results of UV-vis absorption spectra shows the EDA complexation between enamine and 2 (Fig. S10†). After the visible-light irradiation of the EDA complex, perfluoroalkyl radicals, iodide ions, and enamine radical cations were produced. The enamine catalyst was then regenerated *via* single-electron transfer (SET) from iodine ions,^19^ and iodine radicals were then reconverted to iodine ions by amine (DIPEA˙^+^/DIPEA = +0.68 V *vs.* SCE,^20^ I_2_/2I^−^ = +0.54 V (ref. 21)). Finally, HI derived from the iodine ions, formed salt with amine (Fig. S12†).^15*b*^ Simultaneously, DIPEA was responsible of a halogen bonding interaction with 2 and then perfluoroalkyl radicals were generated by visible-light irradiation.^17^ Next, the produced perfluoroalkyl radicals attacked the substrate 1 or 6, and the subsequent radical intermediate A was promptly trapped by gaseous oxygen to produce peroxyl intermediate B. Based on the finding that less than 1.0 eq. of oxygen is sufficient for the reaction (Table 1, entry 13), it is assumed that the reaction involves the formation of dimer C, which was formed *via* the reaction of B and A.^18^ Finally, the desired product 5 or 7 was produced *via* hydrogen atom transfer from DIPEA radical cations, as previously reported.^18^

## Conclusions

In conclusion, we demonstrated the transition metal-free visible-light-induced hydroxy-perfuoroalkylation of electron-deficient conjugated olefins and styrenes using enamine and DIPEA as photo-organocatalysts. This green protocol could be applied to various commercially available substrates and perfluoroalkyl iodides. Further investigations on the reaction mechanism and substrate scope are currently underway in our laboratory.

## Conflicts of interest

There are no conflicts to declare.

## Supplementary Material

RA-012-D2RA06679C-s001
