# Supplementary material for: Metal-free visible-light-induced hydroxy-perfluoroalkylation of conjugated olefins using enamine catalyst
Source: RSC Adv. 2022 Nov 15;12(51):32790–5. doi: 10.1039/d2ra06679c (PMC9667149; doi:10.1039/d2ra06679c)

Electronic Supplementary Material (ESI) for RSC Advances.
This journal is © The Royal Society of Chemistry 2022

Supporting information

Metal-free visible-light-induced hydroxy-perfluoroalkylation of conjugated olefins using enamine catalyst

Koto Tagami, Yu Ofuji, Tadashi Kanbara and Tomoko Yajima

Department of Chemistry, Faculty of Science, Ochanomizu University, 2-1-1, Otsuka, Bunkyo-ku, Tokyo 112-8610, Japan

E-mail address: yajima.tomoko@ocha.ac.jp

Tables of Contents

1. General information S3
2. General procedures S3
3. Optimization studies S4
3–1. Evaluation of oxygen equivalents S4
3–2. Evaluation of **3** and **4** equivalents S5
3–3. Reactions with presynthesized enamine complex S5
3–4. Evaluation of tertiary amine S6
3–5. Evaluation of photocatalyst S6
3–6. Limitation of the substrate scope S7
3–7. Evaluation of **2** for **1a** S7
3–8. Reaction using other CF_2 radical precursors S8
4. Stereochemistry S8
4–1. Determination of diastereoselectivity by HPLC S8
4–2. Stereo determination of compounds **5la** S9
5. Gram scale reactions S10
6. Mechanistic studies S11
6–1. Labelled experiments S11
6–2. Titration experiment S12
6–3. Determination of binding stoichiometry S14
6–4. Determination of the association constant (K_a) S16
6–5. UV-Vis absorption spectra S18
6–6. Crude ^{1}H NMR chart analysis S19
7. Characterization of the products S21
8. References S37
9. ^{1}H, ^{13}C and ^{19}F NMR spectra S38

1. General information

Unless otherwise noted, all reactions were performed under argon atmosphere. ^{1}H, ^{13}C and ^{19}F NMR spectra were recorded on a JEOL GSX-400 spectrometer (400 MHz for ^{1}H, 126 MHz for ^{13}C, and 376 MHz for ^{19}F), a JEOL ECX-500 spectrometer (500 MHz for ^{1}H and 471 MHz for ^{19}F), or a BRUKER AVANCE 600 spectrometer (600 MHz for ^{1}H and 151 MHz for ^{13}C) with $CDCl_3$ as solvent, tetramethylsilane (TMS: δ 0 ppm for ^{1}H), chloroform-d ($CDCl_3$: δ 76.9 ppm for ^{13}C) and hexafluorobenzene (C_6F_6: -162.2 ppm for ^{19}F) as an internal standard. IR spectra were taken on SHIMADZU IRSpirit-T, and HRMS were obtained with a JEOL JMS-T100TD (DART-TOF), JEOL JMS-700 (EI), or BRUKER Compact (ESI). Precoated Merck Kieselgel 60 F254 and Kanto silica gel 60 (spherical neutral) were used for thin layer chromatography and flash chromatography, respectively. Visualization was accomplished by UV light (254 nm). All commercially available reagents and solvents were used as received, without further purification. Starting materials **1j**[1], **1k**[1], **1l**[2] and **1s**[3] were synthesized according to the previous literature. For irradiation, 2.5 W white light-emitting diodes (HLV3-22SW-1, CCS Inc.) were used (https://www.ccs-grp.com/products/model/3803).

2. General procedures

Scheme S1. General procedures for hydroxy-perfluoroalkylation.

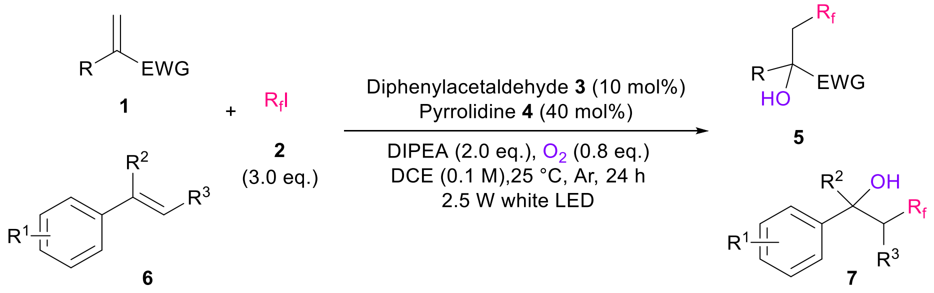

To an oven-dried 20 ml two-necked flask equipped with a magnetic stirrer bar was fitted with a septum and degassed through alternating vacuum evacuation/argon backfill (×10) before dry 1,2-dichloroethane (2.5 ml) was added. Then oxygen (5.0 mL, 0.8 eq.), diphenylacetaldehyde (**3**, 4.4μL, 10 mmol), pyrrolidine (**4**, 8.4 μL, 40 mol%), DIPEA (88 μL, 2.0 eq.), electron-deficient olefin **1** or styrene **6** (0.25 mmol), and perfluoroalkyl iodide **2** (3.0 eq.) were added respectively. The mixture was stirred for 24 hours at 25°C under white LED irradiation. The resulting mixture was concentrated *in vacuo*. The residue was purified by column chromatography on silica gel to give **5** or **7** as a yellow oil or white solid.

3. Optimization studies

3–1. Evaluation of oxygen equivalents

Table S1. Evaluation of oxygen equivalents

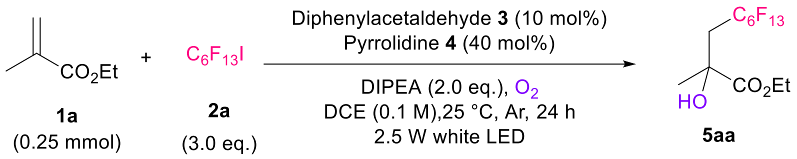

Entry	O_2	yield[a]
1	-	trace
2	0.5 eq. (3.1 mL)	58%
3	0.6 eq. (3.7 mL)	61%
4	0.7 eq. (4.3 mL)	68%
**5**	**0.8 eq. (5.0 mL)**	**75%**
6	0.9 eq. (5.5 mL)	68%
7	1.0 eq. (6.1 mL)	53%
8	2.0 eq. (12.3 mL)	30%
9	excess (50 mL)	9%

[a]Yields based on ^{19}F NMR spectroscopy using benzotrifluoride as an internal standard.

The reaction does not proceeded without oxygen (entry 1). With low amount of oxygen, oligomerization of **1a** was observed (entries 2-4). However, with high amount of oxygen, by-product derived from **4**, **2a**, and oxygen were obtained (Scheme S1), and only 9% of **5aa** were produced in the oxygen atmosphere (entry 9).[4]

Scheme S2. Side reaction from **4**, **2a**, and oxygen.[4]

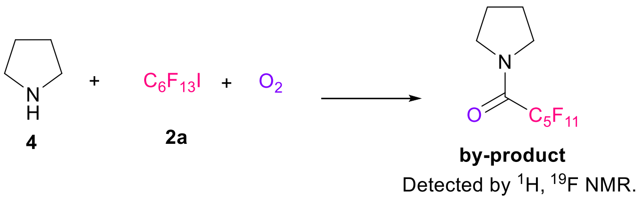

3–2. Evaluation of 3 and 4 equivalents

Table S2. Evaluation of adding quantity of **3** and **4**

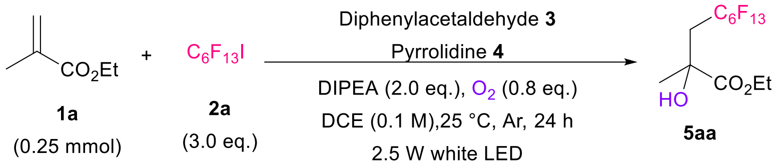

Entry	3	4	yield[a]
1	10 mol% (4.9 mg)	10 mol% (2.1 μL)	59%
**2**	**10 mol% (4.9 mg)**	**40 mol% (8.4 μL)**	**75%**
3	1 mol% (0.5 mg)	1 mol% (0.2 μL)	53%
4	1 mol% (0.5 mg)	40 mol% (8.4 μL)	73%

[a]Yields based on ^{19}F NMR spectroscopy using benzotrifluoride as an internal standard.

3–3. Reactions with pre-synthesized enamine

Enamine was synthesized according to the previous literature.[5]

Table S3. Reaction with pre-synthesized enamine.

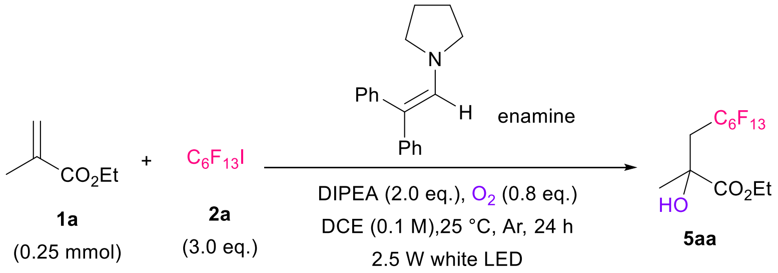

Entry	enamine	pyrrolidine (**4**)	yield[a]
1	10 mol% (6.2 mg)	-	60%
2	40 mol% (24.9 mg)	-	72%
3	10 mol% (6.2 mg)	30 mol% (6.3 μL)	74%

[a]Yields based on ^{19}F NMR spectroscopy using benzotrifluoride as an internal standard.

3–4. Evaluation of tertiary amine

Table S4. Evaluation of tertiary amine.

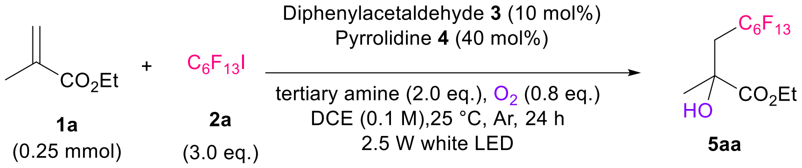

entry	tertiary amine	yield[a]
1	*N*-methyl pyrrolidine	51%
2	TEA	49%
**3**	**DIPEA**	**75%**
4	DIPEA[b]	74%
5	TBA	73%
6	TMEDA	63%
7	TEEDA	55%
8	PMDETA	48%

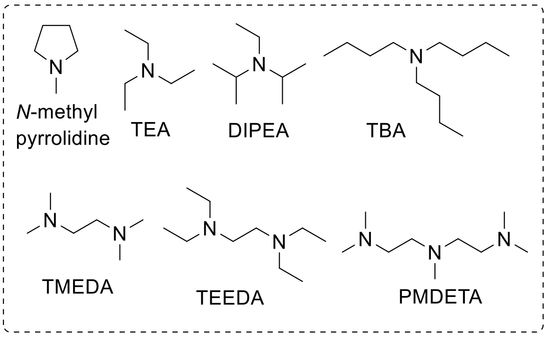

[a]Yields based on ^{19}F NMR spectroscopy using benzotrifluoride as an internal standard. [b]3.0 eq. of DIPEA.

3–5. Evaluation of photocatalyst

Table S5. Evaluation of photocatalyst.

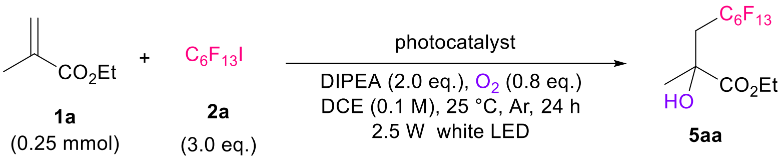

entry	Photocatalyst		yield[a]
**1**	**pyrrolidine**<br>**diphenylacetaldehyde**	**10mol%**<br>**40mol%**	**75%**
2	Eosin Y-2Na	10 mol%	36%
3	Phloxine B-2Na	10 mol%	40%
4	Methylene Blue-3H_2O	10 mol%	37%
5	[Ir(dtbbpy)(ppy)$_2$]PF_6	1 mol%	65%
6	Ru(bpy)$_3$$Cl_2-6H_2O$	1 mol%	63%

[a]Yields based on ^{19}F NMR spectroscopy using benzotrifluoride as an internal standard.

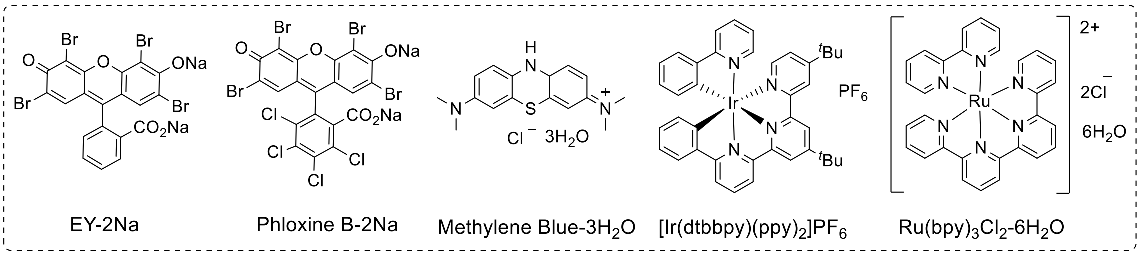

3–6. Limitation of the substrate scope

Table S6. Limitation of the substrate scope.

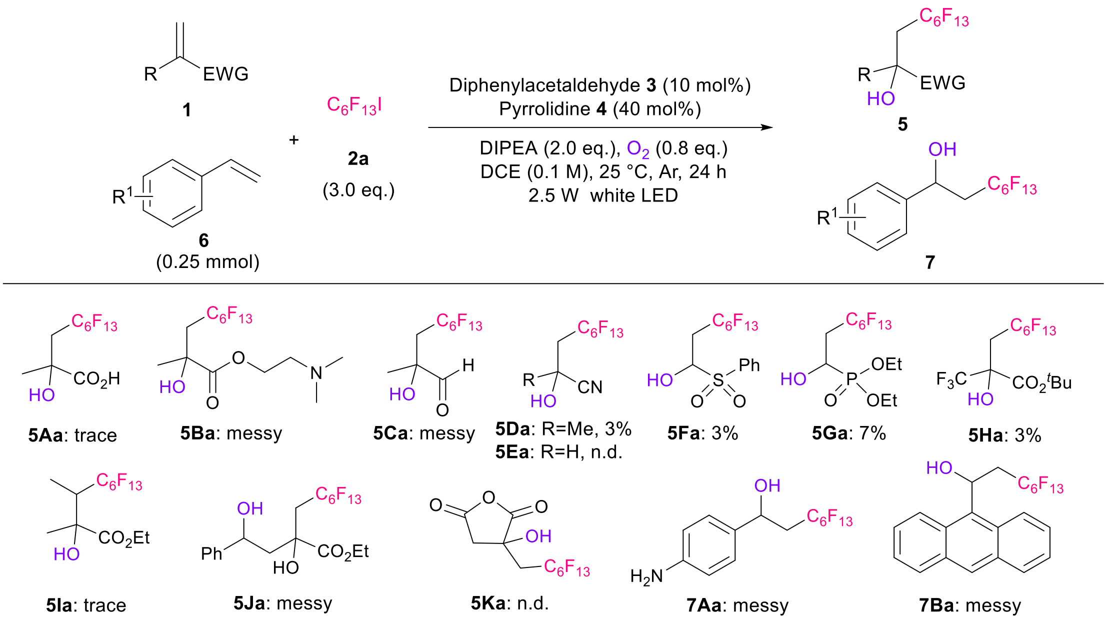

Yields based on ^{19}F NMR spectroscopy using benzotrifluoride as an internal standard.

3–7. Evaluation of 2 for 1a

Table S7. Evaluation of **2** for **1a**.

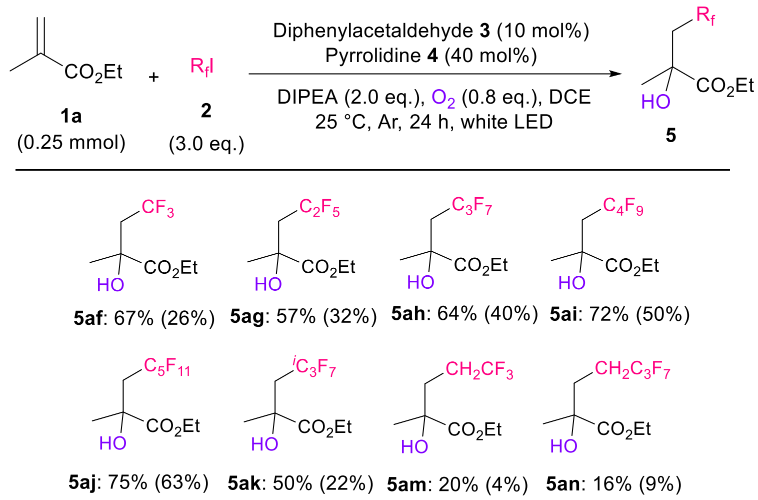

Yields based on ^{19}F NMR spectroscopy using benzotrifluoride as an internal standard; isolated yields are given in parentheses.

Owing to their low boiling point, the isolated yields were lower than the ^{19}F NMR yields.

3–8. Reaction using XCF_2CO_2Et

Table S8. Reaction using XCF_2CO_2Et.

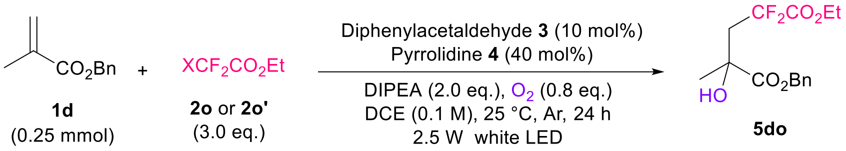

Entry	2	yield (%)[a]
1	**2o**: ICF_2CO_2Et	9
2	**2o'**: $BrCF_2CO_2Et$	n.r.

[a]based on ^{19}F NMR spectroscopy using benzotrifluoride as an internal standard.

4. Stereochemistry

4–1. Determination of diastereoselectivity of compound 5la by HPLC

HPLC: GL Science Inc. Inertsil® Diol column; detected at 294 nm; hexane/ethanol, 95/5; flow = 1.0 mL/min; retention times: 6.40 min, 6.92 min.

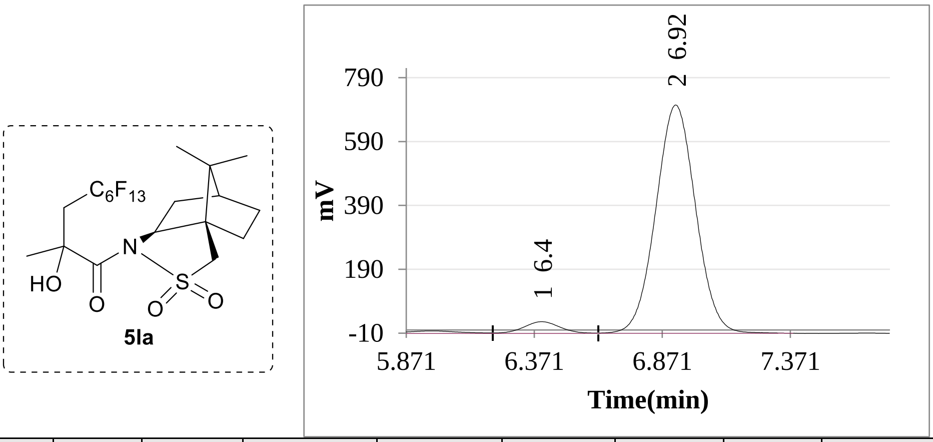

No.	Rt	Peak Name	Area	Area(%)	Height	NTP	Tf	Resolution
1	6.4		366987.5	4.5187	36970	9392.6	0.975	1.894
2	6.92		7754629	95.4813	714956	9207.3	1.033	----
Total			8121617	100	751926			

d.r. = 95:5.

Figure S1. Determination of diastereoselectivity of compound **5la** by HPLC

4–2. Determination of stereochemistry (compounds 5la).[6]

Stereochemistry of **5la** was determined by comparing the optical rotation of compound **5lf'** with known compound.[6]

Scheme S3. Determination of stereochemistry of compound **5lf'**

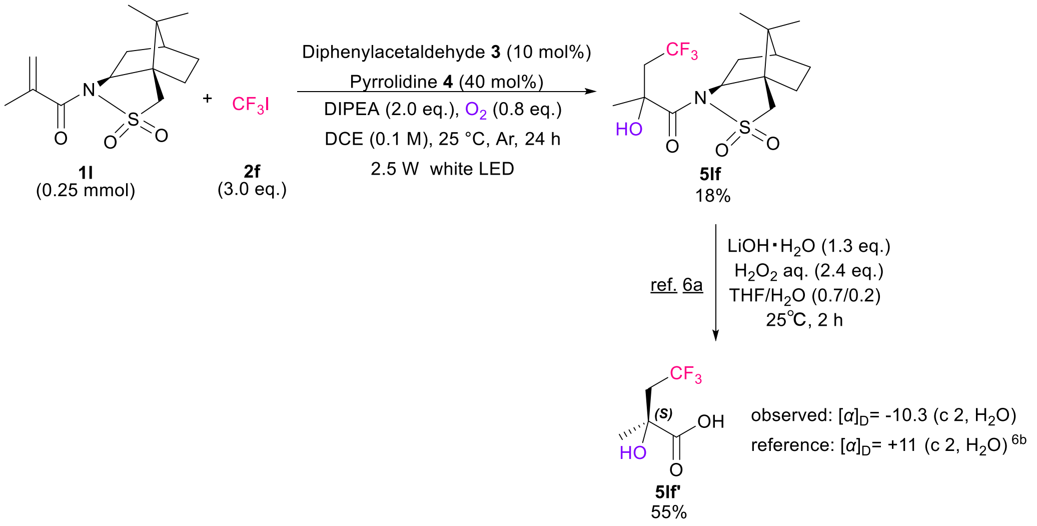

Yields based on ^{19}F NMR spectroscopy using benzotrifluoride as an internal standard.

5. Gram scale reaction

Scheme S4. Gram scale reaction.

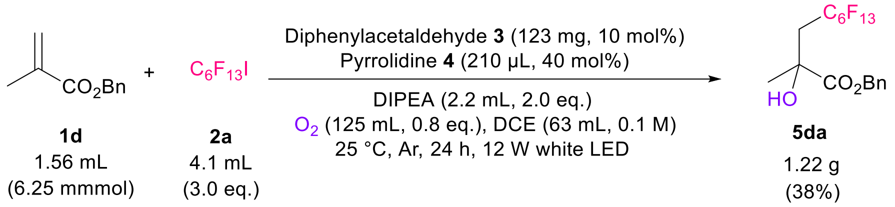

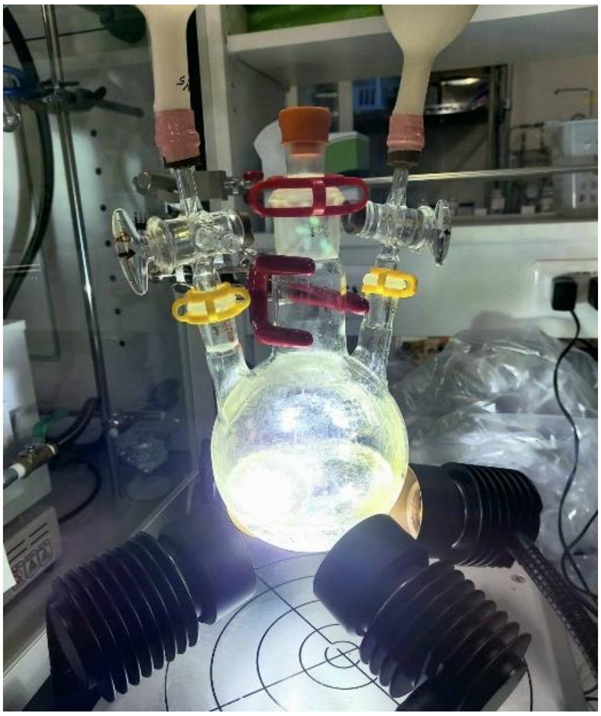

Figure S2. Gram scale reaction apparatus.

6. Mechanistic studies

6–1. Labelled experiments

Scheme S5. Labelled experiments with $H_2{}^{18}O$ or $^{18}O_2$.

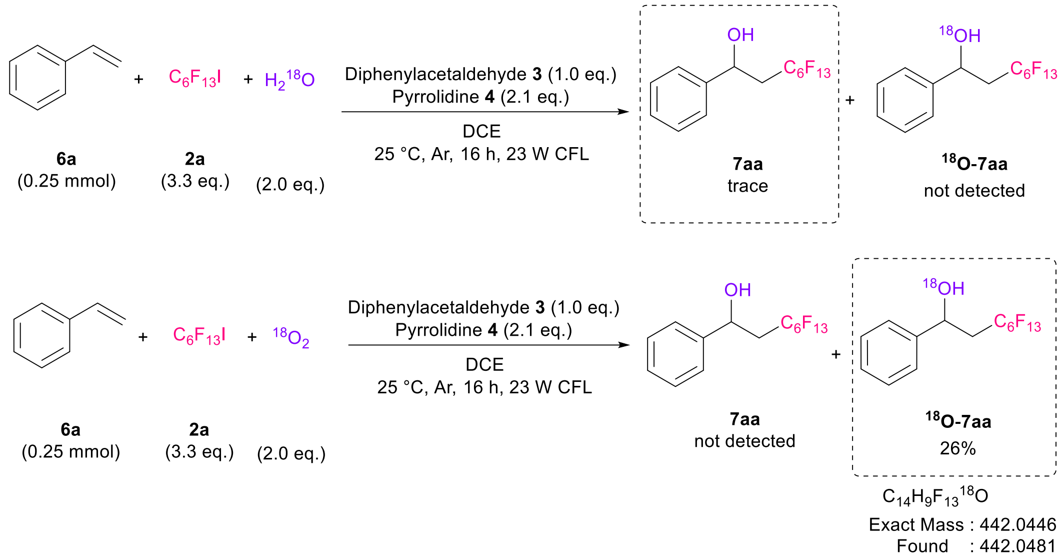

RT : 0.21 min Scan# : 6
Elements : C 24/0, H 9/9, F 13/12, 16O 1/0, 18O 1/0
Mass Tolerance : 5ppm, 5mmu if m/z < 1000, 10mmu if m/z > 2000
Unsaturation (U.S.) : -0.5 - 10.0

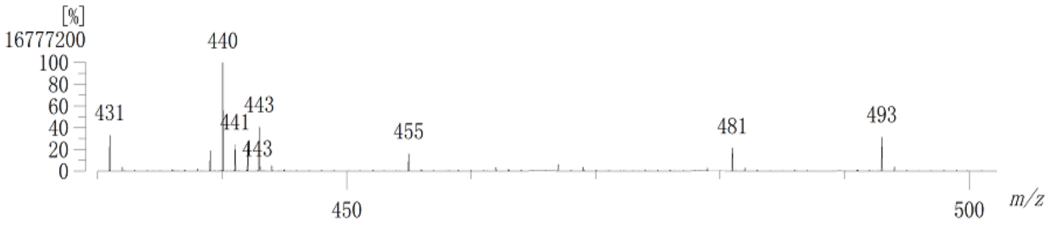

	Observed m/z	Int%	Err[ppm / mmu]	U.S.	Composition
	430.9735	33.63			
	439.0371	18.81			
1	440.0453	100.00	+1.6 / +0.7	4.0	C14 H9 F13 16O
	441.0428	24.75			
2	442.0481	27.30	-1.6 / -0.7	4.0	C14 H9 F13 18O
	442.9717	41.07			

Figure S3. MS spectra of labelled experiments with or $^{18}O_2$.

6–2. Titration experiment[7]

6–2–1. Titration experiment of $C_6F_{13}I$ with enamine

^{19}F NMR spectra of seven samples of the mixtures of $C_6F_{13}I$ (**2a**), diphenylacetaldehyde (**3**) and pyrrolidine (**4**) (**3**:**4** = 1:1) in $CDCl_3$ were recorded (hexafluorobenzene (C_6F_6: -162.2 ppm for ^{19}F) was used as an internal standard). The amount of **2a** was kept constant at 0.025 mmol while that of **3** and **4** was varied from 0 to 0.125 mmol, respectively. The molar ratio of **2a**: (**3**+**4**) were 1:0, 1:1, 1:2, 1:3, 1:4, 1:7, and 1:10.

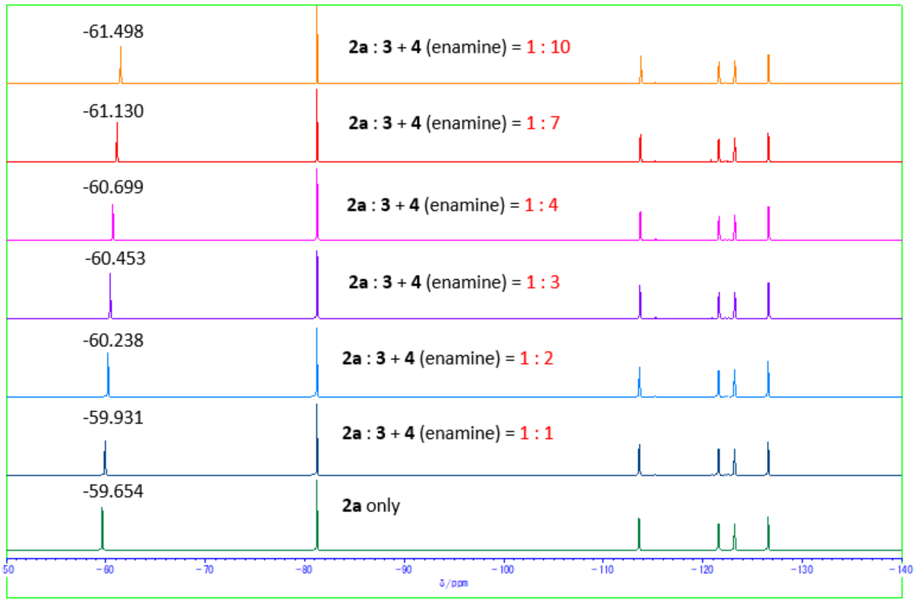

Figure S4–1. ^{19}F Titration experiment of $C_6F_{13}I$ with enamine.

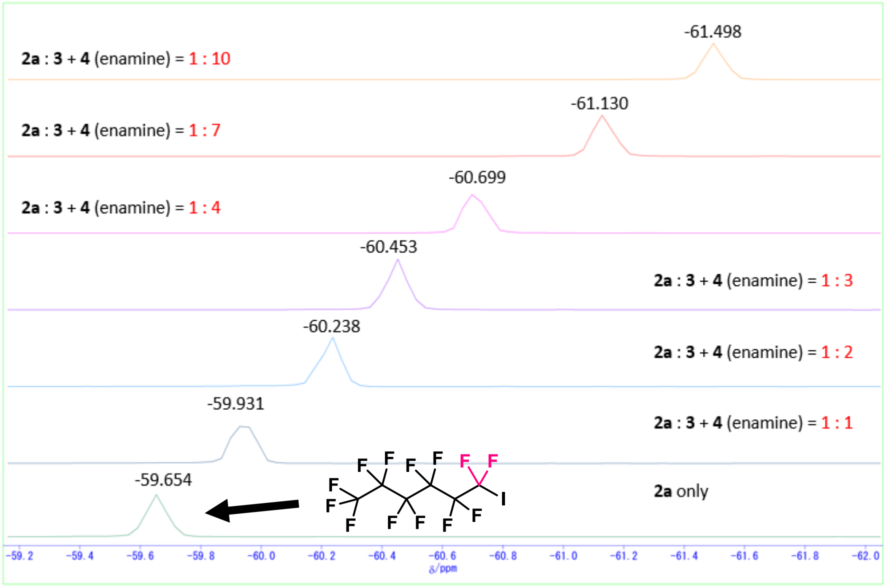

Figure S4–2. ^{19}F Titration experiment of $C_6F_{13}I$ with enamine.

6–2–2. Titration experiment of $C_6F_{13}I$ with DIPEA

^{19}F NMR spectra of seven samples of the mixtures of $C_6F_{13}I$ (**2a**) and DIPEA in $CDCl_3$ were recorded (hexafluorobenzene (C_6F_6: -162.2 ppm for ^{19}F) was used as an internal standard). The amount of_**2a** was kept constant at 0.025 mmol while that of DIPEA was varied from 0 to 0.25 mmol. The molar ratio of **2a**: DIPEA were 1:0, 1:1, 1:2, 1:3, 1:4, 1:5, and 1:10.

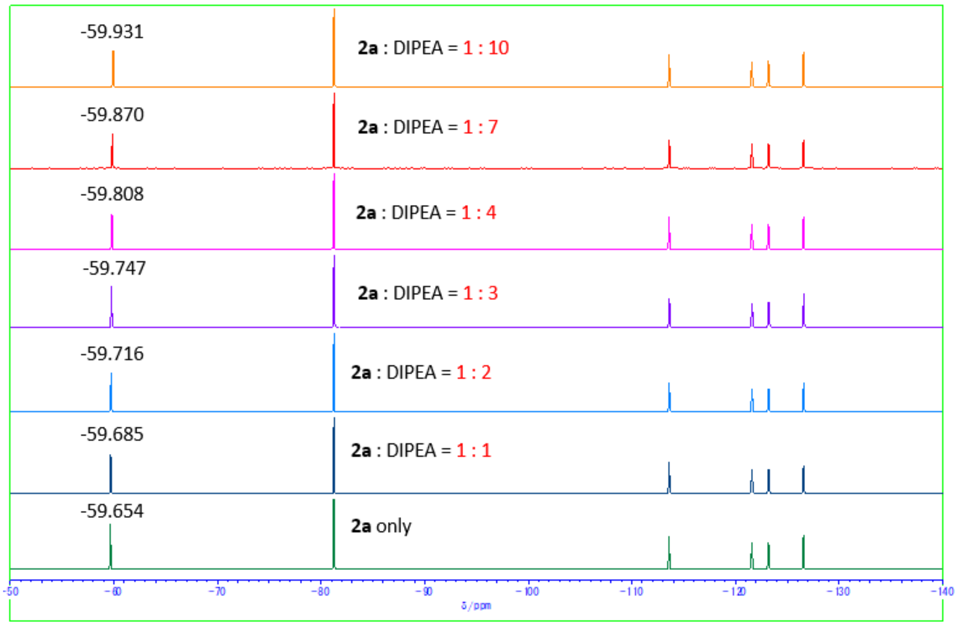

Figure S5–1. ^{19}F Titration experiment of $C_6F_{13}I$ with DIPEA.

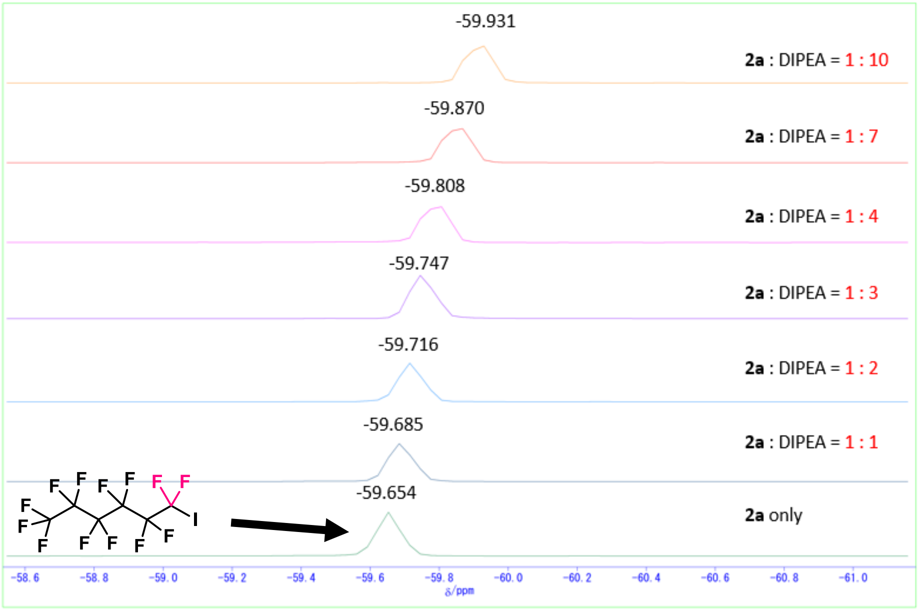

Figure S5–2. ^{19}F Titration experiment of $C_6F_{13}I$ with DIPEA.

The -CF_2I upfield-shift of the ^{19}F NMR was observed with the addition of enamine or DIPEA into **2a**. It indicated the formation of halogen-bonding between **2a** and enamine or DIPEA. Figure S4 shows a clearer upfield-shift than Figure S5, indicating that the interaction between **2a** and enamine was stronger than that of DIPEA.

6–3. Determination of binding stoichiometry

Determination of binding stoichiometry experiments were conducted by Job's plot analysis[8] according to the previous literature.[7]

6–3–1. Determination of binding stoichiometry between $C_6F_{13}I$ and enamine

^{19}F NMR spectra of eleven samples of the mixtures of $C_6F_{13}I$ (**2a**), diphenylacetaldehyde (**3**) and pyrrolidine (**4**) (**3**:**4** = 1:1) in $CDCl_3$ were recorded (hexafluorobenzene (C_6F_6: -162.2 ppm for ^{19}F) was used as an internal standard). The total amount of **2a**, **3**, and **4** were kept constant at 0.25 mmol (0.5 M), while the amount of **2a** was varied from 0 to 0.25 mmol (0–0.5 M). The molar ratio of **2a**/: {**2a**+(**3**+**4**)} were 0.0, 0.1, 0.2, 0.3, 0.4, 0.5, 0.6, 0.7 0.8, 0.9, 1.0. ^{19}F NMR for each sample was recorded to measure the change in chemical shift for the F of CF_2I. The stoichiometry was determined by plotting ratios of [**2a**]×Δδ against ratios of [**2a**]/ [**2a**+**3**+**4**]. Figure S6 show the maximum at ratio [**2a**]/[**2a**+**3**+**4**] = 0.5, which meant a 1:1 complex ratio between **2a** and enamine.

[**2a**] (M)	Δδ(ppm)	[**2a**] /[**2a**+**3**+**4**]	[**2a**]×Δδ(M ppm)
0	0.000	0.0	0
0.05	2.550	0.1	0.255
0.1	2.181	0.2	0.4362
0.15	1.905	0.3	0.5715
0.2	1.567	0.4	0.6268
0.25	1.352	0.5	0.676
0.3	1.106	0.6	0.6636
0.35	0.922	0.7	0.6454
0.4	0.676	0.8	0.5408
0.45	0.368	0.9	0.3312
0.5	0.000	1.0	0

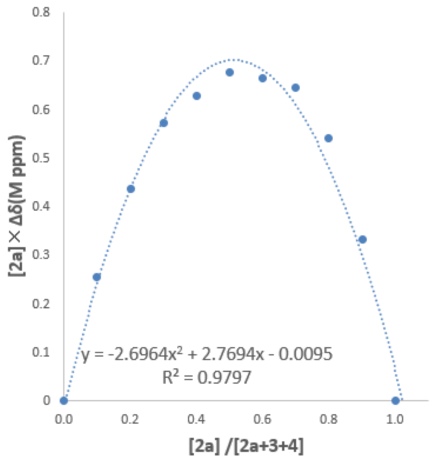

Figure S6. Job's plot between $C_6F_{13}I$ and enamine.

6–3–2. Determination of binding stoichiometry between $C_6F_{13}I$ and DIPEA

^{19}F NMR spectra of eleven samples of the mixtures of $C_6F_{13}I$ (**2a**), DIPEA in $CDCl_3$ were recorded (hexafluorobenzene (C_6F_6: -162.2 ppm for ^{19}F) was used as an internal standard). The total amount of **2a** and DIPEA were kept constant at 0.25 mmol (0.5 M), while the amount of **2a** was varied from 0 to 0.25 mmol (0–0.5 M). The molar ratio of **2a**/: {**2a**+DIPEA} were 0.0, 0.1, 0.2, 0.3, 0.4, 0.5, 0.6, 0.7 0.8, 0.9, 1.0. ^{19}F NMR for each sample was recorded to measure the change in chemical shift for the F of CF_2I. The stoichiometry was determined by plotting ratios of [**2a**]×Δδ against ratios of [**2a**]/ [**2a**+DIPEA]. Figure S7 show the maximum at ratio [**2a**]/[**2a**+ DIPEA] = 0.5, which meant a 1:1 complex ratio between **2a** and DIPEA.

[**2a**] (M)	Δδ(ppm)	[**2a**] /[**2a**+DIPEA]	[**2a**]×Δδ(M ppm)
0	0.000	0.0	0
0.05	0.491	0.1	0.0491
0.1	0.461	0.2	0.0922
0.15	0.399	0.3	0.1197
0.2	0.338	0.4	0.1352
0.25	0.276	0.5	0.138
0.3	0.245	0.6	0.147
0.35	0.184	0.7	0.1288
0.4	0.123	0.8	0.0984
0.45	0.061	0.9	0.0549
0.5	0.000	1.0	0

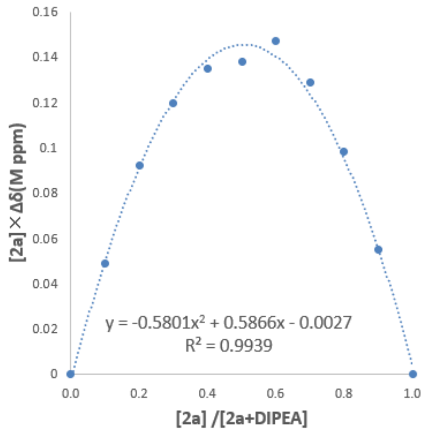

Figure S7. Job's plot between $C_6F_{13}I$ and DIPEA.

6–4. Determination of the association constant (K_a)

The association constant (K_a) was calculated using Hanna and Ashbaugh's method,[9] according to the previous literature.[7]

6–4–1. Determination of the association constant (K_a) between $C_6F_{13}I$ and enamine

^{19}F NMR spectra of six samples of the mixtures of $C_6F_{13}I$ (**2a**), diphenylacetaldehyde (**3**) and pyrrolidine (**4**) (**3**:**4** = 1:1) in $CDCl_3$ were recorded (hexafluorobenzene (C_6F_6: -162.2 ppm for ^{19}F) was used as an internal standard). The amount of **2a** was kept constant at 0.025 mmol while that of **3** and **4** was varied from 0.0125 to 0.125 mmol, respectively. The molar ratio of **2a**: (**3**+**4**) were 1:1, 1:2, 1:3, 1:4, 1:7, and 1:10. ^{19}F NMR for each sample was recorded to measure the change in chemical shift for the F of CF_2I.

**3 + 4** (mmol)	**3 + 4** (mol/L)	1/**3 + 4** (L/mol)	Δδ (ppm)	1/Δδ (ppm^{-1})
0.25	0.42	2.4	1.844	0.5423
0.175	0.29	3.4	1.476	0.6775
0.1	0.17	6.0	1.045	0.9569
0.075	0.13	8.0	0.799	1.2516
0.05	0.08	12.0	0.584	1.7123
0.025	0.04	24.0	0.277	3.6101

K = 0.1377/1.1419

= **0.97**

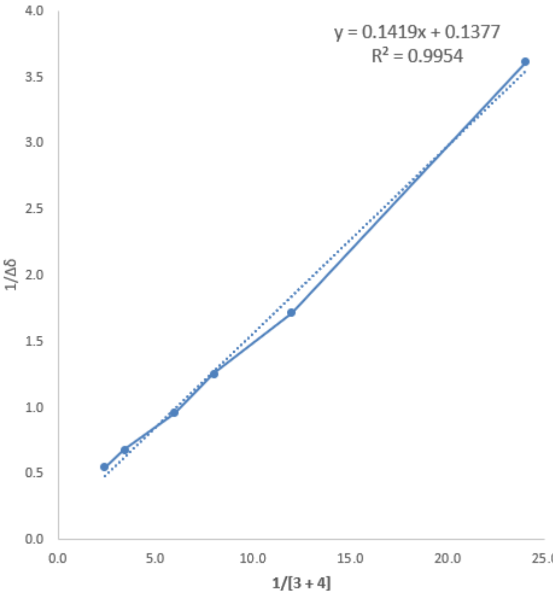

Figure S8. association constant (K_a) between $C_6F_{13}I$ and enamine

The association constant of **2a** and enamine ($K_{enamine}$) was calculated to be 0.97 ($K_{enamine}$ = 0.97 M^{-1}).

6–4–2. Determination of the association constant (K_a) between $C_6F_{13}I$ and DIPEA

^{19}F NMR spectra of six samples of the mixtures of $C_6F_{13}I$ (**2a**) and DIPEA in $CDCl_3$ were recorded (hexafluorobenzene (C_6F_6: -162.2 ppm for ^{19}F) was used as an internal standard). The amount of **2a** was kept constant at 0.025 mmol while that of DIPEA was varied from 0.025 to 0.25 mmol. The molar ratio of **2a**:DIPEA were 1:1, 1:2, 1:3, 1:4, 1:7, and 1:10. ^{19}F NMR for each sample was recorded to measure the change in chemical shift for the F of CF_2I.

DIPEA (mmol)	DIPEA (mol/L)	1/DIPEA (L/mol)	Δδ (ppm)	1/Δδ (ppm^{-1})
0.25	0.42	2.4	0.277	3.6101
0.175	0.29	3.4	0.216	4.6296
0.1	0.17	6.0	0.154	6.4935
0.075	0.13	8.0	0.093	10.7527
0.05	0.08	12.0	0.062	16.1290
0.025	0.04	24.0	0.031	32.2581

K = 0.2648/1.3517
= **0.20**

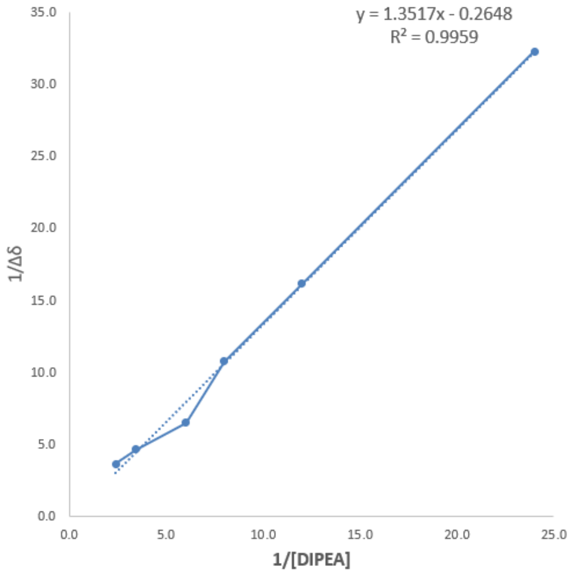

Figure S9. association constant (K_a) between $C_6F_{13}I$ and DIPEA

The association constant of **2a** and DIPEA (K_{DIPEA}) was calculated to be 0.20 (K_{DIPEA} = 0.20 M^{-1}).

A comparison of the association constant (K_a) in Figure S8 and S9 suggested that enamine interacted with $C_6F_{13}I$ more effectively that DIPEA.

6–5. UV-Vis absorption spectra

Optical absorption spectra were recorded 1,2-dichloroethane solution (0.1 M) in 1 mm path quartz cuvettes using a JASCO V-650 UV-visible Spectrophotometer.

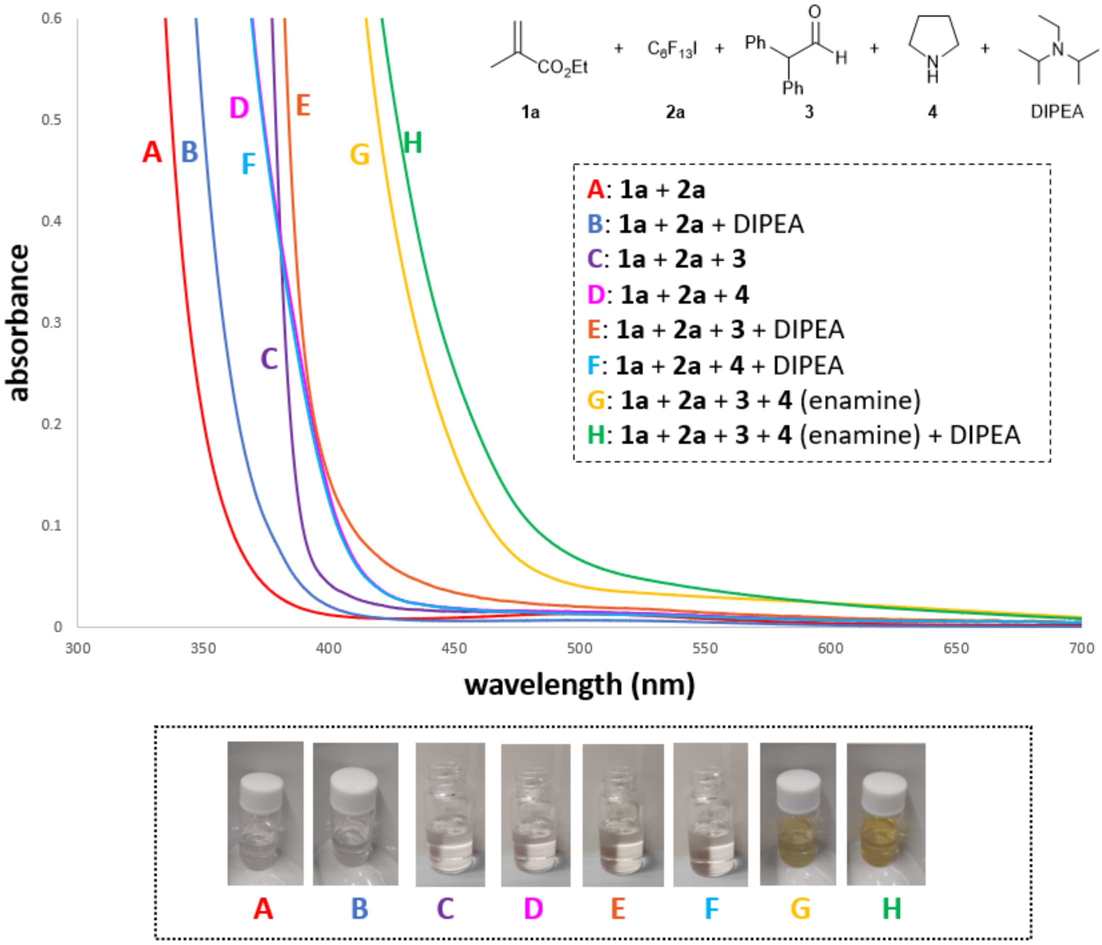

Figure S10. UV-Vis absorption spectra and visual appearance of EDA complexation.

The biggest long-wavelength shifted peak was observed for the solution **G** and **H** (with both **3** and **4**; enamine), as well as a visual appearance (yellow colour). It indicated that EDA complex was formed between **2a** and enamine. One the other hand, there are no colour change and little peak shift for solution **B**–**F** (with only **DIPEA**, **3**, or **4**) compared to solution **A**. This reslut exclude the possibility of the EDA complexation between DIPEA, **3**, or **4** with **2a**, respectively.

6–6. Crude ^{1}H NMR

6–6–1. Crude ^{1}H NMR compared with **3**, **4** and enamine.

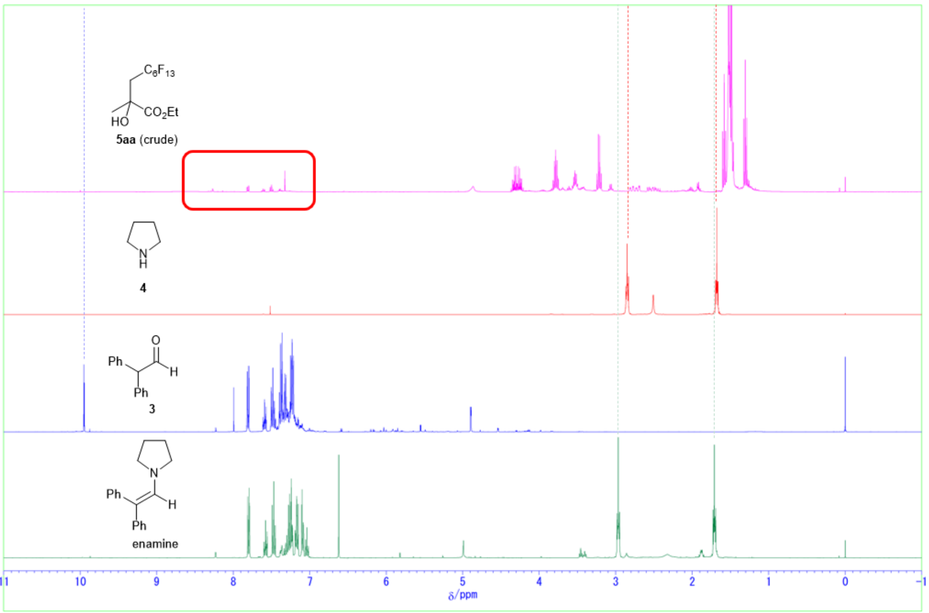

Figure S11. Crude ^{1}H NMR compared with **3**, **4** and enamine.

Crude ^{1}H NMR (top row) shows that aldehyde **3** remained after the reaction (the red circled part).

6–6–2. Crude ^{1}H NMR compared with DIPEA.

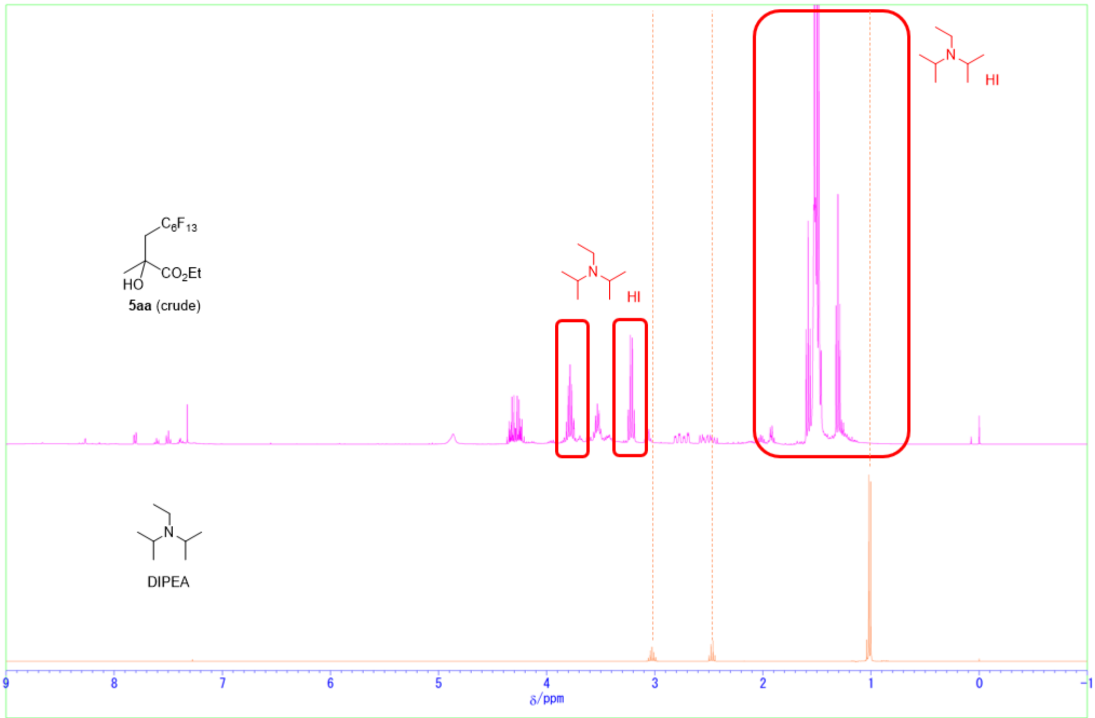

Figure S12. DIPEA after the reaction.

In Crude ^{1}H NMR, remaining free DIPEA was not observed. A comparison with previous studies[10] suggests that the red circled peak is the DIPEA and HI salt.

7. Characterization of the products

Ethyl 4,4,5,5,6,6,7,7,8,8,9,9,9-tridecafluoro-2-hydroxy-2-methylnonanoate (**5aa**)

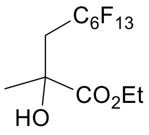

5aa

Yellow oil, 90.2 mg, yield: 80%. ^{1}H NMR (400 MHz, $CDCl_3$) δ: 4.37–4.22 (2H, m), 3.50 (1H, s), 2.81–2.68 (1H, m), 2.58–2.44 (1H, m), 1.52 (3H, s), 1.31 (3H, t, J = 7.3 Hz). ^{13}C N MR (151 MHz, $CDCl_3$) δ:175.3, 120.4–106.6 (6C, m), 71.4 (d, J = 1.5 Hz), 62.8, 39.4 (t, J = 20.4 Hz), 28.0, 14.0.^{19}F NMR (376 MHz, $CDCl_3$) δ: -81.3 (3F, s), -110.3 (1F, d, J = 288.9 Hz), -114.7 (1F, d, J = 288.9 Hz), -122.2 (2F, s), -123.4 (2F, s), -124.5 (2F, s), -126.7 (2F, s). IR (neat, cm^{-1}) 3389, 3061, 3028, 2922, 1780, 1279, 1236, 1186, 1142. HRMS (ESI^{+}) calcd for $C_{12}H_{11}F_{13}O_3$ $[M+Na]^{+}$: 473.0393, found 473.0406.

Methyl 4,4,5,5,6,6,7,7,8,8,9,9,9-tridecafluoro-2-hydroxy-2-methylnonanoate (**5ba**)

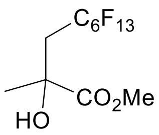

5ba

Yellow oil, 79.0 mg, yield: 73%. ^{1}H NMR (500 MHz, $CDCl_3$) δ: 3.84 (3H, s), 3.46 (1H, s), 2.76–2.66 (1H, m), 2.57–2.46 (1H, m), 1.52 (3H, s). ^{13}C NMR (151 MHz, $CDCl_3$) δ: 175.7, 118.2-108.9 (6C, m), 71.4 (d, J = 1.5 Hz), 53.6, 39.5 (t, J = 19.6 Hz), 27.9. ^{19}F NMR (471 MHz, $CDCl_3$) δ: -81.3 (3F, s), -111.3 (1F, d, J = 277.9 Hz), -115.1 (1F, d, J = 277.9 Hz), -122.3 (2F, s), -123.4 (2F, s), -124.5 (2F, s), -126.7 (2F, s). IR (neat, cm^{-1}) 3059, 2924, 2855, 1719, 1317, 1238, 1177. HRMS (DART^{+}) calcd for $C_{11}H_9F_{13}O_3$ $[M+H]^{+}$: 437.0422, found 437.0442.

tert-Butyl 4,4,5,5,6,6,7,7,8,8,9,9,9-tridecafluoro-2-hydroxy-2-methylnonanoate (**5ca**)

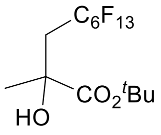

5ca

White solid, 95.9 mg, yield: 80%. ^{1}H NMR (500 MHz, $CDCl_3$) δ: 3.53 (1H, s), 2.77-2.67 (1H, m), 2.51–2.41 (1H, m), 1.49 (9H, s), 1.46 (3H, s).^{13}C NMR (151 MHz, $CDCl_3$) δ: 174.4, 120.2-108.1 (6C, m), 83.7, 71.4 (d, J = 3.0 Hz), 39.3 (t, J = 19.6 Hz), 31.1, 28.2, 27.7 (2C).^{19}F NMR (471 MHz,$CDCl_3$) δ: -81.3 (3F, s), -109.3 (1F, d, J = 272.5 Hz), -114.5 (1F, d, J = 272.5 Hz), -122.3 (2F, s), -123.4 (2F, s), -124.7 (2F, s), -126.7 (2F, s). IR (neat, cm^{-1}) 3482, 3092, 3063, 3036, 1653, 1279, 1233, 1188, 1142, 1121. HRMS (ESI^{+}) calcd for $C_{14}H_{15}F_{13}O_3$ $[M+Na]^{+}$: 501.0706, found 501.0707.

Benzyl 4,4,5,5,6,6,7,7,8,8,9,9,9-tridecafluoro-2-hydroxy-2-methylnonanoate (**5da**)

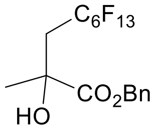

5da

Yellow solid, 101.57 mg, yield: 79%. ^{1}H NMR (500 MHz, $CDCl_3$) δ: 7.40–7.26 (5H, m), 5.23 (2H, dd, J = 16.0, 12.0 Hz), 3.46 (1H, s), 2.79–2.68 (1H, m), 2.56–2.48 (1H, m), 1.51 (3H, s). ^{13}C NMR (151 MHz, $CDCl_3$) δ: 175.1, 134.6, 129.0, 128.8 (2C), 128.7, 119.5–108.3 (6C, m), 71.5 (d, J = 3.0 Hz), 68.7, 39.4 (t, J = 21.1 Hz), 28.0. ^{19}F-NMR (471 MHz, $CDCl_3$) δ: -81.3 (3F, s), -110.8 (1F, d, J = 272.5 Hz), -114.8 (1F, d, J = 272.5 Hz), -122.3 (2F, s), -123.4 (2F, s), -124.4 (2F, s), -126.7 (2F, s). IR (neat, cm^{-1}) 3503,

3094, 3036, 2982, 2893, 1742, 1233, 1184, 1142, 746, 696. HRMS (DART$^+$) calcd for $C_{17}H_{13}F_{13}O_3$ [M+H]$^+$: 513.0735, found 513.0741.

Phenyl 4,4,5,5,6,6,7,7,8,8,9,9,9-tridecafluoro-2-hydroxy-2-methylnonanoate (**5ea**)

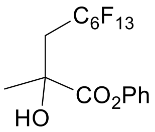

5ea

White solid, 85.9 mg, yield: 69%. ^{1}H NMR (400 MHz, $CDCl_3$) δ: 7.45–7.40 (2H, m), 7.31–7.26 (1H, m), 7.08 (2H, d, J = 7.8 Hz), 3.47 (1H, s), 3.01–2.88 (1H, m), 2.73–2.59 (1H, m), 1.71 (3H, s). ^{13}C NMR (151 MHz, $CDCl_3$) δ: 174.1, 150.3, 129.9 (2C), 126.8, 121.1, 120.3–108.2 (6C, m), 71.7 (d, J = 3.0 Hz), 39.6 (t, J = 20.4 Hz), 28.2. ^{19}F NMR (376 MHz, $CDCl_3$) δ: -81.3 (3F, s), -110.3 (1F, d, J = 283.2 Hz), -113.9 (1F, d, J = 283.2 Hz), -122.2 (2F, s), -123.3 (2F, s), -124.4 (2F, s), -126.6 (2F, s). IR (neat, cm^{-1}) 3466, 2580, 1762, 1366, 1237, 1189, 1144, 1121, 749, 700. HRMS (ESI$^+$) calcd for $C_{16}H_{11}F_{13}O_3$ [M+Na]$^+$: 521.0393, found 521.0407.

Cyclohexyl 4,4,5,5,6,6,7,7,8,8,9,9,9-tridecafluoro-2-hydroxy-2-methylnonanoate (**5fa**)

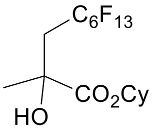

5fa

Yellow oil, 101.89 mg, yield: 81%. ^{1}H NMR (500 MHz, $CDCl_3$) δ: 4.92 (1H, m), 3.52 (1H, s), 2.80–2.69 (1H, m), 2.55–2.44 (1H, m), 1.86 (2H, m), 1.73 (2H, m), 1.56 (2H, m), 1.51–1.35 (6H, m), 1.50 (3H, s). ^{13}C NMR (151 MHz, $CDCl_3$) δ: 174.8, 119.5–107.6 (6C, m), 75.6, 71.4 (d, J = 3.0 Hz), 39.3 (t, J = 21.1 Hz), 31.5, 31.1, 28.2, 25.3, 23.7, 23.6. ^{19}F NMR (471 MHz, $CDCl_3$) δ: -81.3 (3F, s), -110.3 (1F, d, J = 272.5 Hz), -114.7 (1F, d, J = 272.5 Hz), -122.3 (2F, s), -123.4 (2F, s), -124.5 (2F, s), -126.7 (2F, s). IR (neat, cm^{-1}) 3509, 2942, 2864, 1732, 1451, 1235, 1180, 1144, 1123. HRMS (DART$^+$) calcd for $C_{16}H_{17}F_{13}O_3$ [M+H]$^+$: 505.1048, found 505.1016.

Oxiran-2-ylmethyl 4,4,5,5,6,6,7,7,8,8,9,9,9-tridecafluoro-2-hydroxy-2-methylnonanoate (**5ga**)

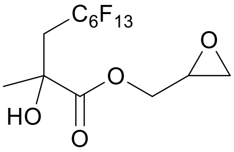

5ga

Yellow oil, 84.1 mg, yield: 70%. ^{1}H NMR (400 MHz, $CDCl_3$) δ: 4.37–4.28 (1H, m), 3.92–3.86 (1H, m), 3.61 (1H, s), 3.41–3.23 (2H, m), 2.85–2.70 (1H, m), 2.64–2.48 (2H, m), 1.56 (3H, s).^{13}C NMR (151 MHz, $CDCl_3$) δ: 175.1 (d, J = 4.5 Hz), 119.4–106.4 (6C, m), 71.7, 68.9 (d, J = 1.5 Hz), 68.7, 39.5 (t, J = 19.6 Hz), 28.1 8.6 (d, J = 15.1 Hz). ^{19}F NMR (376 MHz, $CDCl_3$) δ: 81.3 (3F, s), -110.7 (1F, d, J = 277.4 Hz), -114.4 (1F, d, J = 277.4 Hz), -122.2 (2F, s), -123.4 (2F, s), -124.4 (2F, s), -126.6 (2F, s). IR (neat, cm^{-1}) 3415, 2958, 1743, 1317, 1232, 1187, 1139, 799, 707. HRMS (ESI$^+$) calcd for $C_{13}H_{11}F_{13}O_4$ [M+Na]$^+$: 501.0342, found 501.0340.

2,2,2-Trifluoroethyl 4,4,5,5,6,6,7,7,8,8,9,9,9-tridecafluoro-2-hydroxy-2-methylnonanoate (**5ha**)

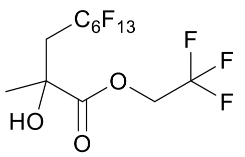

5ha

Yellow oil, 83.7 mg, yield: 68%. ^{1}H NMR (400 MHz, $CDCl_3$) δ: 4.67-4.51 (2H, m), 3.30 (1H, s), 2.86–2.72 (1H, m), 2.64–2.50 (1H, m), 1.58 (3H, s). ^{13}C NMR (151 MHz, $CDCl_3$) δ: 173.9, 128.5–108.3, (7C, m), 71.7 (d, *J* = 3.0 Hz), 61.9 (dd, *J* = 74.7, 37.0 Hz), 39.5 (t, *J* = 20.4 Hz), 27.9. ^{19}F NMR (376 MHz, $CDCl_3$) δ: -74.5 (3F, s), -81.3 (3F, s), -110.5 (1F, d, *J* = 266.96 Hz), -114.6 (1F, d, *J* = 266.96 Hz), -122.3 (2F, s), -123.4 (2F, s), -124.5 (2F, s), -126.6 (2F, s). IR (neat, cm^{-1}) 3519, 2987, 1763, 1364, 1287, 1235, 1136, 814, 700, 654. HRMS (ESI^+) calcd for $C_{12}H_8F_{16}O_3$ $[M+Na]^+$: 527.0110, found 527.0126.

(1*R*,2*R*,4*R*)-1,7,7-trimethyl-2-bicyclo[2.2.1]heptanyl 4,4,5,5,6,6,7,7,8,8,9,9,9-tridecafluoro-2-hydroxy-2-methylnonanoate (**5ia**)

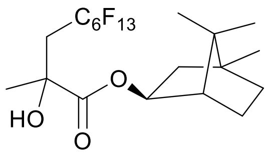

5ia

(52: 48 diastereomer mixture, dr was measured by HPLC.)

Yellow oil, 98.5 mg, yield: 71%. ^{1}H NMR (400 MHz, $CDCl_3$) δ: 4.80 (minor, 1H, dd, *J* = 6.9, 2.7 Hz), 4.64 (major, 1H, dd, *J* = 7.8, 3.2 Hz), 3.56 (minor, 1H, s), 3.53 (major, 1H, s), 2.73–2.60 (major and minor, 1H, m), 2.57–2.43 (major and minor, 1H, m), 1.92 (major, 1H, dd, *J* = 13.7, 7.8 Hz), 1.85–1.79 (minor, 1H, m), 1.85–1.68 (major and minor, 3H, m), 1.63–1.56 (major and minor, 1H, m), 1.49 (major and minor, 3H, d, *J* = 6.4 Hz), 1.23–1.08 (major and minor, 2H, m), 0.96 (major and minor, 3H, s), 0.87 (major and minor, 3H, s), 0.86 (major and minor, 3H, s). ^{13}C NMR (151 MHz, $CDCl_3$) δ: 174.9 (minor), 174.8 (major), 129.0–106.6 (major and minor, 6C, m), 84.5 (major), 83.9 (minor), 71.4 (major, d, *J* = 1.5 Hz), 71.2 (minor, d, *J* = 1.5 Hz), 49.2 (major), 48.7 (minor), 47.2 (major), 47.1 (minor), 45.1 (major), 45.0 (minor), 39.2 (major and minor, m), 38.0 (major and minor), 33.9 (major), 33.7 (minor), 28.2 (minor), 27.9 (major), 27.0 (major and minor), 20.1 (major and minor), 19.9 (major), 19.7 (minor), 11.5 (major), 11.3 (minor). ^{19}F NMR (376 MHz, $CDCl_3$) δ: -81.4 (3F, s), -110.9 (1F, dd, *J* = 267.1, 67.7 Hz), -114.6 (1F, dd, *J* = 312.2, 45.1 Hz),-122.4 (2F, s), -123.5 (2F, s), -124.3 (2F, d, *J* = 69.4 Hz), -126.8 (2F, s). IR (neat, cm^{-1}) 3518, 2958, 1736, 1458, 1232, 1144, 814, 773, 732, 700. HRMS (ESI^+) calcd for $C_{20}H_{23}F_{13}O_3$ $[M+Na]^+$: 581.1332, found 581.1350. HPLC: GL Science Inc. Inertsil® Diol column; detected at 294 nm; hexanes/ethanol, 95/5; flow = 1.0 mL/min; retention times: 5.50 min, 7.04 min.

(1*R*,2*S*,5*R*)-5-Methyl-2-(1-methylethyl)cyclohexyl 4,4,5,5,6,6,7,7,8,8,9,9,9-tridecafluoro-2-hydroxy-2-methylnonanoate (**5ja**)

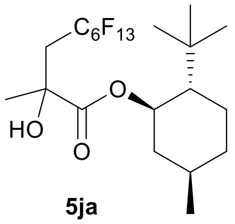

5ja

(54: 46 diastereomer mixture, dr was measured by HPLC.)

Yellow oil, 115.9 mg, yield: 82%. ^{1}H NMR (400 MHz, $CDCl_3$) δ: 4.83–4.73 (major and minor, 1H, m), 3.55 (major, 1H, s), 3.54 (minor, 1H, s), 2.82–2.70 (major and minor, 1H, m), 2.56–2.42 (major and minor, 1H, m), 2.04–1.98 (major and minor, 1H, m), 1.90–1.79 (major and minor, 1H, m), 1.71 (major and minor, 2H, d, *J* = 11.9 Hz), 1.55–1.40 (major and minor, 5H, m), 1.12–0.96 (major and minor, 2H, m), 0.94–0.83 (major and minor, 7H, m), 0.74 (major and minor, 3H, dd, *J* = 15.1, 7.1 Hz). ^{13}C NMR (151 MHz, $CDCl_3$) δ: 175.0 (major and minor), 119.5–108.7 (major and minor, 6C, m), 77.5 (major and minor), 71.5 (minor, m), 71.4 (major, m), 47.1 (minor), 47.0 (major), 40.4 (major), 40.0 (minor), 39.3 (major, t, *J* = 20.4 Hz), 38.7 (minor, t, *J* = 20.4 Hz), 34.2 (major and minor), 31.5 (major and minor), 28.4 (major). 28.2 (minor), 26.2 (major), 25.6 (minor), 23.1 (minor), 22.8 (major), 22.1 (major), 22.0 (minor), 21.0 (minor), 20.9 (major), 15.8 (major), 15.4 (minor). ^{19}F NMR (376 MHz, $CDCl_3$) δ: -81.3 (3F, s), -109.7 (1F, dd, *J* = 278.4, 184.3 Hz), -114.6 (1F, t, *J* = 289.6 Hz), -122.3 (2F, d, *J* = 34.7 Hz), -123.4 (2F, s), -124.4 (2F, d, *J* = 57.8 Hz), -126.7 (2F, s). IR (neat, cm^{-1})3453, 2960, 1748, 1458, 1232, 1187, 1144, 1009, 814, 748, 699. HRMS (ESI^+) calcd for $C_{20}H_{25}F_{13}O_3$ $[M+Na]^+$: 583.1488, found 583.1501. HPLC: GL Science Inc. Inertsil® Diol column; detected at 294 nm; hexane/ethanol, 95/5; flow = 1.0 mL/min; retention times: 3.09 min, 3.86 min.

(1*R*,2*S*,5*R*)-5-Methyl-2-(1-methy-1-phenylethyl)cyclohexyl 4,4,5,5,6,6,7,7,8,8,9,9,9-tridecafluoro-2-hydroxy-2-methylnonanoate (**5ka**)

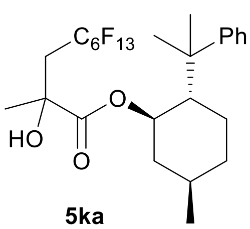

5ka

(74: 26 diastereomer mixture, dr was measured by HPLC.)

Yellow oil, 111.9 mg, yield: 70%. ^{1}H NMR (400 MHz, $CDCl_3$) δ: 7.37–7.28 (major and minor, 4H, m), 7.22–7.16 (major and minor, 1H, m), 4.90–4.79 (major and minor, 1H, m), 2.54–2.38 (major and minor, 1H, m), 2.30–2.14 (major and minor, 2H, m), 2.04–1.95 (major and minor, 1H, m), 1.83–1.79 (major and minor, 1H, m), 1.73–1.66 (major and minor, 1H, m), 1.55–1.47 (major and minor, 1H, m), 1.34 (major and minor, 3H, s), 1.27 (major and minor, 3H, s), 1.22–1.17 (major and minor, 5H, m), 1.16–0.99 (major and minor, 2H, m), 0.96–0.86 (major and minor, 3H, m). ^{13}C NMR (151 MHz, $CDCl_3$) δ: 174.4 (minor), 173.1 (major), 152.2 (major), 151.6 (minor), 128.5 (major, 2C), 128.4 (minor, 2C), 125.7 (major), 125.6 (minor), 125.4 (minor), 125.3 (major), 121.9–106.8 (major and minor, 6C, m), 78.5 (minor), 78.0 (major), 72.3 (major), 71.8 (minor), 49.5 (major), 49.4 (minor), 40.7 (minor), 40.5 (major), 39.7 (minor), 39.6 (major), 39.0 (major, t, *J* = 19.6 Hz), 38.5 (minor, t, *J* = 19.6 Hz), 34.6 (major and minor), 31.4 (major and minor), 29.5 (major), 28.7 (minor), 27.3 (major and minor), 26.9 (minor), 26.7 (major), 25.3 (minor), 24.0 (major), 21.8 (major and minor).

^{19}F NMR (376 MHz, $CDCl_3$) δ: -81.3 (3F, s), -108.0–-114.4 (2F, m), -122.3 (2F, s), -123.4 (2F, s), -124.3 (2F, d, *J* = 57.8 Hz), -126.7 (2F, s). IR (neat, cm^{-1}) 3564, 2958, 1729, 1459, 1364, 1235, 1187, 1144, 732, 700. HRMS (ESI^+) calcd for $C_{26}H_{29}F_{13}O_3$ $[M+Na]^+$: 659.1801, found 659.1801. HPLC: DAICEL. Chiralpak OH column; detected at 294 nm; hexane/IPA, 90/10; flow = 0.25 mL/min; retention times: 18.09 min, 19.06 min.

4,4,5,5,6,6,7,7,8,8,9,9,9-Tridecafluoro-2-hydroxy-2-methyl-1-(10,10-dimethyl-3,3-dioxo-3λ^{6}-thia-4-azatricyclo[5.2.1.0^{1,5}]decan-4-yl)nonane-1-one (**5la**)

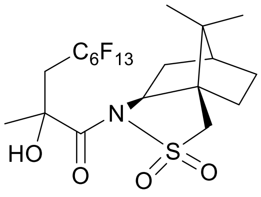

5la

(95: 5 diastereomer mixture, dr was measured by HPLC.)

Yellow oil, 37.1 mg, yield: 24%. ^{1}H NMR (500 MHz, $CDCl_3$, major) δ: 4.04 (1H, dd, *J* = 7.7, 4.6 Hz), 3.75 (1H, s), 3.58 (1H, d, *J* = 13.5 Hz), 3.47 (1H, d, *J* = 13.5 Hz), 3.09–3.04 (1H, m), 2.46–2.42 (1H, m), 2.04–1.87 (5H, m), 1.63 (3H, s), 1.46–1.41 (1H, m), 1.36–1.34 (1H, m), 1.18 (3H, s), 0.99 (3H, s). ^{13}C NMR (151 MHz, $CDCl_3$, major) δ: 175.8, 143.2–97.9 (6C, m), 75.7, 67.7, 53.9, 48.7, 47.9, 44.9, 41.2 (t, *J* = 19.6 Hz), 38.2, 33.4, 26.9, 26.5, 21.0, 20.1. ^{19}F NMR (471 MHz, $CDCl_3$, major) δ: -81.0 (3F, s), -107.7 (1F, d, *J* = 289.0 Hz), -102.5 (1F, d, *J* = 289.0 Hz), -121.9 (2F, s), -123.1 (2F, s), -124.1 (2F, s), -126.4 (2F, s). IR (neat, cm^{-1}) 3495, 2984, 2976, 2959, 2889, 1699, 1314, 1236, 1173, 1159, 1142, 1123. $[\alpha]_D^{21}$ -22.3 (c 1, $CHCl_3$). HRMS ($DART^+$) calcd for $C_{20}H_{22}F_{13}NO_4S$ $[M+H]^+$: 620.1140, found 620.1134. HPLC: GL Science Inc. Inertsil® Diol column; detected at 294 nm; hexane/ethanol, 95/5; flow = 1.0 mL/min; retention times: 6.40 min, 6.92 min.

4,4,5,5,6,6,7,7,8,8,9,9,9-Tridecafluoro-2-hydroxy-*N*,*N*,2-trimethylnonanamide (**5ma**)

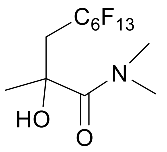

5ma

Yellow oil, 21.0 mg, yield: 18%. ^{1}H NMR (500 MHz, $CDCl_3$) δ: 4.84 (1H, s), 3.11 (6H, s), 2.76–2.59 (2H, m), 1.61 (3H, s). ^{13}C NMR (151 MHz, $CDCl_3$) δ: 173.5, 132.6–108.5 (6C, m), 71.4, 39.7 (t, *J* = 21.1 Hz), 38.6, 27.2 (2C). ^{19}F NMR (471 MHz, $CDCl_3$) δ: -81.3 (3F, s), -112.3 (1F, d, *J* = 267.0 Hz), -114.3 (1F, d, *J* = 267.0 Hz), -122.2 (2F, s), -123.3 (2F, s), -124.2 (2F, s), -126.6 (2F, s). IR (neat, cm^{-1}) 3268, 2982, 2955, 2934, 1616, 1233, 1184, 1165, 1140, 1123. HRMS ($DART^+$) calcd for $C_{12}H_{12}F_{13}NO_2$ $[M+H]^+$: 450.0739, found 450.0699.

4,4,5,5,6,6,7,7,8,8,9,9,9-Tridecafluoro-2-hydroxy-*N*,*N*-dimethylnonanamide (**5na**)

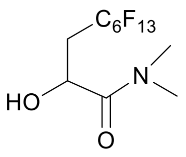

5na

Yellow oil, 20.6 mg, yield: 20%. ^{1}H NMR (400 MHz, $CDCl_3$) δ: 4.85–4.80 (1H, m), 3.90 (1H, d, *J* = 7.8 Hz), 3.05 (3H, s), 3.02 (3H, s), 2.43–2.29 (2H, m). ^{13}C NMR (151 MHz, $CDCl_3$) δ: 172.2, 119.6–105.8 (6C, m), 61.9, 36.8 (t, *J* = 23.4 Hz), 36.4 (2C). ^{19}F NMR (376 MHz, $CDCl_3$) δ: -81.3 (3F, s), -113.5 (1F, d, *J* = 277.4 Hz), -114.4 (1F, d, *J* = 277.4 Hz), -122.3 (2F, s), -123.4 (2F, s), -124.0 (2F, s), -

126.6 (2F, s). IR (neat, cm^{-1}) 3347, 1634, 1228, 1185, 1141, 1121, 1086, 716, 656. HRMS (ESI$^+$) calcd for $C_{11}H_{10}NO_2$ [M+Na]$^+$: 458.0396, found 458.0414.

Methyl 4,4,5,5,6,6,7,7,8,8,9,9,9-tridecafluoro-2-hydroxynonanoate (**5oa**)

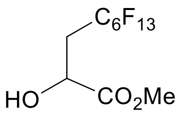

5oa

Yellow oil, 17.9 mg, yield: 17%. ^{1}H NMR (400 MHz, $CDCl_3$) δ: 4.62–4.58 (1H, m), 3.87 (3H, s), 3.02 (1H, d, J = 5.0 Hz), 2.76–2.62 (1H, m), 2.54–2.39 (1H, m). ^{13}C NMR (151 MHz, $CDCl_3$) δ: 173.5, 119.3–110.3 (6C, m), 64.9, 53.5, 35.4 (t, J = 21.1 Hz). ^{19}F NMR (376 MHz, $CDCl_3$) δ: -81.3 (3F, s), -113.2 (1F, d, J = 277.4 Hz), -114.2 (1F, d, J = 277.4 Hz), -121.3 (2F, s), -123.4 (2F, s), -124.1 (2F, s), -126.6 (2F, s). IR (neat, cm^{-1}) 3459, 2963, 1748, 1565, 1442, 1230, 1191, 1142, 815, 705. HRMS (ESI$^+$) calcd for $C_{10}H_7F_{13}O_3$ [M+Na]$^+$: 445.0080, found 445.0078.

Ethyl 4,4,5,5,6,6,7,7,8,8,9,9,9-tridecafluoro-2-hydroxynonanoate (**5pa**)

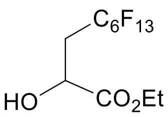

5pa

Yellow oil, 25.2 mg, yield: 23%. ^{1}H NMR (400 MHz, $CDCl_3$) δ: 4.59–4.56 (1H, m), 4.32 (2H, dd, J = 14.4, 7.6 Hz), 3.06 (1H, d, J = 5.0 Hz), 2.75-2.61 (1H, m), 2.54–2.39 (1H, m), 1.33 (3H, t, J = 7.1 Hz). ^{13}C NMR (151 MHz, $CDCl_3$) δ 170.5, 122.1–106.8 (6C, m), 65.0, 63.0, 35.3 (t, J = 20.4 Hz), 14.2. ^{19}F NMR (376 MHz, $CDCl_3$) δ: -81.3 (3F, s), -113.5 (2F, t, J = 294.8 Hz), -122.3 (2F, s), -123.4 (2F, s), -124.1 (2F, s), -126.6 (2F, s). IR (neat, cm^{-1}) 3461, 2989, 1743, 1565, 1531, 1231, 1122, 779, 707. HRMS (ESI$^+$) calcd for $C_{11}H_{19}F_{13}O_3$ [M+Na]$^+$: 459.0236, found 459.0236.

tert-Butyl 4,4,5,5,6,6,7,7,8,8,9,9,9-tridecafluoro-2-hydroxynonanoate (**5qa**)

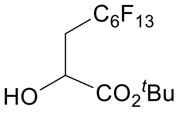

5qa

Yellow oil, 37.3 mg, yield: 32%. ^{1}H NMR (400 MHz, $CDCl_3$) δ: 4.45–4.41 (1H, m), 3.11 (1H, d, J = 4.6 Hz), 2.69–2.55 (1H, m), 2.51–2.36 (1H, m), 1.51 (9H, s). ^{13}C NMR (151 MHz, $CDCl_3$) δ: 172.2, 119.5–108.5 (6C, m), 84.2, 65.3, 35.3 (t, J = 21.1 Hz), 28.0 (3C). ^{19}F NMR (376 MHz, $CDCl_3$) δ: -81.1 (3F, s), -112.9 (2F, s), -122.2 (2F, s), -123.3 (2F, s), -124.0 (2F, s), -126.5 (2F, s). IR (neat, cm^{-1}) 3433, 2981, 1727, 1166, 1139, 1086, 829, 752, 703. HRMS (ESI$^+$) calcd for $C_{13}H_{13}F_{13}O_3$ [M+Na]$^+$: 487.0549, found 487.0549.

Benzyl 4,4,5,5,6,6,7,7,8,8,9,9,9-tridecafluoro-2-hydroxynonanoate (**5ra**)

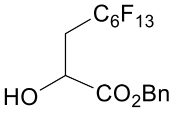

5ra

Yellow oil, 21.9 mg, yield: 18%. ^{1}H NMR (400 MHz, $CDCl_3$) δ: 7.42–7.34 (5H, m), 5.26 (2H, dd, J = 17.9, 12.4 Hz), 4.63–4.59 (1H, m), 3.03 (1H, d, J = 5.0 Hz), 2.75–2.61 (1H, m), 2.54–2.39 (1H, m). ^{13}C NMR (151 MHz, $CDCl_3$) δ: 172.9, 134.5, 129.1, 128.9 (2C), 128.8, 119.3–108.4 (6C, m), 68.6, 65.1, 35.2 (t, J = 21.1 Hz). ^{19}F NMR (376 MHz, $CDCl_3$) δ: -81.3 (3F, s), -113.4 (2F, t, J = 295.2 Hz), -122.3 (2F, s), -123.4 (2F, s), -124.1 (2F, s), -126.7 (2F, s). IR (neat, cm^{-1}) 3464, 2969, 1743, 1529, 1232, 1142, 812, 732,

697. HRMS (ESI$^+$) calcd for $C_{16}H_{11}F_{13}O_3$ [M+Na]$^+$: 521.0393, found 521.0392.

Ethyl 4,4,5,5,6,6,7,7,8,8,9,9,9-tridecafluoro-2-hydroxy-2-phenylethylnonanoate (**5sa**)

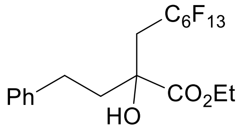

5sa

Yellow oil, 89.4 mg, yield: 66%. ^{1}H NMR (500 MHz, $CDCl_3$) δ: 7.30–7.15 (5H, m), 4.30 (1H, dq, *J* = 10.9, 7.2 Hz), 4.22 (1H, dq, *J* = 10.9, 7.2 Hz), 3.62 (1H, s), 2.83–2.80 (1H, m), 2.79–2.66 (1H, m), 2.58–2.52 (1H, m), 2.43 (1H, m), 2.06 (2H, dt, *J* = 11.5, 5.2 Hz), 1.31 (3H, t, *J* = 7.2 Hz). ^{13}C NMR (151 MHz, $CDCl_3$) δ: 174.5, 140.8, 128.7, 128.5 (2C), 126.3, 120.2–110.5 (6C, m), 73.7, 63.0, 42.2, 39.0 (t, *J* = 21.1 Hz), 29.3, 14.1. ^{19}F NMR (471 MHz, $CDCl_3$) δ: -81.3 (3F, s), -110.5 (1F, d, *J* = 279.3 Hz), -114.1 (1F, d, *J* = 279.3 Hz), -122.3 (2F, s), -123.4 (2F, s), -124.5 (2F, s), -126.7 (2F, s). IR (neat, cm^{-1}) 3528, 3067, 2996, 2951, 1732, 1362, 1229, 1184, 1138, 1121, 745, 696. HRMS (DART$^+$) calcd for $C_{19}H_{17}F_{13}O_3$ [M+H]$^+$: 541.1048, found 541.1083.

Dimethyl 2-hydroxy-2-(2,2,3,3,4,4,5,5,6,6,7,7,7-tridecafluorohexyl)-butanedioate (**5ta**)

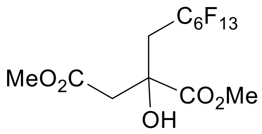

5ta

Yellow oil, 44.3 mg, yield: 36%. ^{1}H NMR (400 MHz, $CDCl_3$) δ: 4.01 (1H, s), 3.87 (1H, s), 3.72 (1H, s), 2.90 (2H, dd, *J* = 60.2, 15.8 Hz), 2.72–2.55 (2H, m). ^{13}C NMR (151 MHz, $CDCl_3$) δ:173.5 170.2, 122.5–106.6 (6C, m), 72.3, 53.8, 52.4, 43.8, 38.6 (t, *J* = 34.7 Hz). ^{19}F NMR (376 MHz, $CDCl_3$) δ: -81.3 (3F, s), -112.0 (1F, d, *J* = 265.9 Hz), -113.8 (1F, d, *J* = 265.9 Hz), -122.2 (2F, s), -123.4 (2F, s), -124.2 (2F, s), -126.6 (2F, s). IR (neat, cm^{-1}) 3532, 1732, 1568, 1364, 1270, 1182, 1159, 1138, 694, 660. HRMS (ESI$^+$) calcd for $C_{13}H_{11}F_{13}O_5$ [M+Na]$^+$: 517.0291, found 517.0306.

Diethyl 2-hydroxy-2-(2,2,3,3,4,4,5,5,6,6,7,7,7-tridecafluorohexyl)-butanedioate (**5ua**)

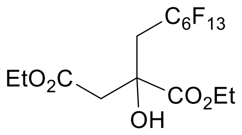

5ua

Yellow oil, 50.6 mg, yield: 39%. ^{1}H NMR (400 MHz, $CDCl_3$) δ: 4.37–4.26 (2H, m), 4.17 (2H, dd, *J* = 7.0, 3.5 Hz), 4.05 (1H, s), 2.87 (2H, dd, *J* = 63.0, 15.8 Hz), 2.69–2.58 (2H, m), 1.32 (3H, t, *J* = 7.1 Hz), 1.26 (3H, t, *J* = 7.1 Hz). ^{13}C NMR (151 MHz, $CDCl_3$) δ: 173.0, 169.7, 120.6–105.9 (6C, m), 72.3, 63.1, 61.4, 44.0, 38.6 (t, *J* = 20.2 Hz), 14.2, 14.0. ^{19}F NMR (376 MHz, $CDCl_3$) δ: -81.3 (3F, s), -111.5 (1F, d, *J* = 277.4 Hz), -113.5 (1F, d, *J* = 277.4 Hz), -122.2 (2F, s), -123.4 (2F, s), -124.2 (2F, s), -126.6 (2F, s). IR (neat, cm^{-1}) 3498, 2989, 1743, 1231, 1189, 1122, 814, 745, 697. HRMS (ESI$^+$) calcd for $C_{15}H_{15}F_{13}O_5$ [M+H]$^+$: 523.0785, found 523.0784.

Ethyl 4,4,5,5,6,6,7,7,8,8,9,9,10,10,10-pentadecafluoro-2-hydroxy-2-methyldecanoate (**5ab**)

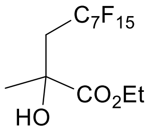

5ab

Yellow oil, 100.29 mg, yield: 80%. ^{1}H NMR (400 MHz, $CDCl_3$) δ: 4.37–4.22 (2H, m), 3.49 (1H, s), 2.81–2.68 (1H, m), 2.57–2.44 (1H, m), 1.51 (3H, s), 1.31 (3H, t, *J* = 7.1 Hz). ^{13}C NMR (151 MHz, $CDCl_3$) δ 175.3, 120.2-106.6 (7C, m), 71.4 (d, *J* = 1.5 Hz), 62.9, 39.4 (t, *J* = 20.4 Hz), 28.0, 14.0. ^{19}F NMR (376 MHz, $CDCl_3$) δ: -81.3 (3F, s), -110.3 (1F, d, *J* = 283.2 Hz), -114.7 (1F, d, *J* = 283.2 Hz), -122.1 (2F, s), -122.6 (2F, s), -123.2 (2F, s), -124.5 (2F, s), -126.7 (2F, s). IR (neat, cm^{-1}) 3511, 2990, 1739, 1452, 1237, 1141, 1014, 721, 702. HRMS (ESI^{+}) calcd for $C_{13}H_{11}F_{15}O_3$ $[M+Na]^{+}$: 523.0361, found 523.0372.

Ethyl 4,4,5,5,6,6,7,7,8,8,9,9,10,10,11,11,11-heptadecafluoro-2-hydroxy-2-methylundecanoate (**5ac**)

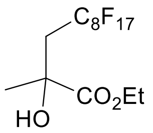

5ac

Yellow solid, 103.0 mg, yield: 76%. ^{1}H NMR (400 MHz, $CDCl_3$) δ: 4.37–4.21 (2H, m), 3.50 (1H, s), 2.80–2.68 (1H, m), 2.57–2.44 (1H, m), 1.52 (3H, s), 1.31 (3H, t, *J* = 7.3 Hz). ^{13}C NMR (151 MHz, $CDCl_3$) δ 175.3, 120.4–106.4 (8C, m), 71.4 (d, *J* = 1.5 Hz), 62.9, 39.4 (t, *J* = 19.6 Hz), 28.0, 14.0. ^{19}F NMR (376 MHz, $CDCl_3$) δ: -81.2 (3F, s), -110.3 (1F, d, *J* = 283.2 Hz), -114.6 (1F, d, *J* = 283.2 Hz), -122.0 (2F, s), -122.4 (4F, s), -123.2 (2F, s), -124.5 (2F, s), -126.6 (2F, s). IR (neat, cm^{-1}) 3504, 1725, 1370, 1326, 1198, 1144, 885, 703, 660. HRMS (ESI^{+}) calcd for $C_{14}H_{11}F_{17}O_3$ $[M+Na]^{+}$: 573.0329, found 573.0333.

Ethyl 4,4,5,5,6,6,7,7,8,8,9,9,10,10,11,11,12,12,13,13,13-henicosafluoro-2-hydroxy-2-methyltridecanoate (**5ad**)

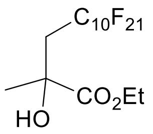

5ad

White solid, 122.6 mg, yield: 76%. ^{1}H NMR (400 MHz, $CDCl_3$) δ: 4.37–4.21 (2H, m), 3.51 (1H, s), 2.80–2.68 (1H, m), 2.57–2.44 (1H, m), 1.51 (3H, s), 1.31 (3H, t, *J* = 7.1 Hz). ^{13}C NMR (151 MHz, $CDCl_3$) δ 175.3, 120.3-106.5 (10C, m), 71.4 (d, *J* = 1.5 Hz), 62.9, 39.4 (t, *J* = 19.6 Hz), 28.0, 14.0. ^{19}F NMR (376 MHz, $CDCl_3$) δ: -81.2 (3F, s), -110.2 (1F, d, *J* = 277.4 Hz), -114.6 (1F, d, *J* = 277.4 Hz), -122.0 (2F, s), -122.3 (8F, s), -123.2 (2F, s), -124.5 (2F, s), -126.6 (2F, s). IR (neat, cm^{-1}) 3505, 1726, 1343, 1214, 1184, 1149, 875, 799, 666. HRMS (ESI^{+}) calcd for $C_{16}H_{11}F_{21}O_3$ $[M+Na]^{+}$: 673.0265, found 673.0264.

Ethyl 4,4-difluoro-4-(2,3,4,5,6-pentafluorophenyl)-2-hydroxy-2-methylbutanoate (**5ae**)

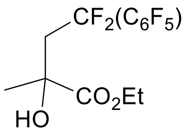

5ae

Yellow oil, 61.5 mg, yield: 71%. ^{1}H NMR (400 MHz, $CDCl_3$) δ: 4.35–4.23 (2H, m), 3.32 (1H, d, *J* = 0.9 Hz), 2.94–2.84 (1H, m), 2.80–2.68 (1H, m), 1.44 (3H, s), 1.34 (3H, t, *J* = 7.1 Hz). ^{13}C NMR (151 MHz, $CDCl_3$) δ 175.7, 145.4-117.9 (7C, m), 71.5 (dd, *J* = 7.6, 1.5 Hz), 62.9, 46.4 (t, *J* = 25.7 Hz), 28.0, 14.1. ^{19}F NMR (376 MHz, $CDCl_3$) δ: -81.9 (1F, d, *J* = 260.7 Hz), -92.9 (1F, dd, *J* = 268.0, 36.7 Hz), -140.7–-140.9 (2F, m), -151.7 (1F, d, *J* = 23.1 Hz), -161.7 (2F, t, *J* = 23.1 Hz). IR (neat, cm^{-1}) 3497, 2942, 1732, 1656, 1528, 1336, 1178, 1047, 990, 852, 684. HRMS (ESI^{+}) calcd for $C_{13}H_{11}F_7O_3$

$[M+Ma]^+$: 371.0489, found 371.0489.

<u>Benzyl 4,4,4-trifluoro-2-hydroxy-2-methylbutanoate (**5df**)</u>

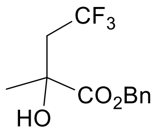

5df

Colorless oil, 50.0 mg, yield: 76%. ^{1}H NMR (400 MHz, $CDCl_3$) δ: 7.42–7.34 (5H, m), 5.23 (2H, dd, *J* = 29.8, 11.9 Hz), 3.43 (1H, s), 2.76–2.65 (m), 2.61–2.50 (1H, m), 1.49 (3H, s). ^{13}C NMR (151 MHz, $CDCl_3$) δ 175.0, 134.7, 128.9 (2C), 128.6 (2C), 128.0–122.5 (1C, m), 71.3 (d, *J* = 3.0 Hz), 68.6, 43.0 (dd, *J* = 55.9, 27.9 Hz), 27.2. ^{19}F NMR (376 MHz, $CDCl_3$) δ: -62.1 (3F, s). IR (neat, cm^{-1}) 3511, 2987, 1739, 1369, 1268, 1182, 1144, 1075, 966, 911, 750. HRMS (ESI$^+$) calcd for $C_{12}H_{13}F_3O_3$ $[M+Na]^+$: 285.0709, found 285.0708.

<u>Benzyl 4,4,5,5,5-pentafluoro-2-hydroxy-2-methylpentanoate (**5dg**)</u>

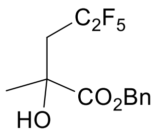

5dg

Yellow oil, 56.0 mg, yield: 72%. ^{1}H NMR (400 MHz, $CDCl_3$) δ: 7.41–7.33 (5H, m), 5.22 (2H, dd, *J* = 11.6, 5.8 Hz), 3.47 (1H, s), 2.74–2.61 (1H, m), 2.54–2.41 (1H, m), 1.51 (3H, s). ^{13}C NMR (151 MHz, $CDCl_3$) δ 175.1, 134.7, 129.0, 128.9, 128.7 (2C), 121.6–113.1 (2C, m), 71.4 (d, *J* = 1.5 Hz), 68.7, 39.3 (t, *J* = 19.6 Hz), 27.8. ^{19}F NMR (376 MHz, $CDCl_3$) δ: -86.9 (3F, s), -115.2 (1F, dd, *J* = 268.8, 26.0 Hz), -118.6 (1F, dd, *J* = 268.8, 26.0 Hz). IR (neat, cm^{-1}) 3511, 2987, 1739, 1333, 1197, 1144, 1020, 961, 910, 752, 697. HRMS (ESI$^+$) calcd for $C_{13}H_{13}F_5O_3$ $[M+Na]^+$: 335.0677, found 335.0672.

<u>Benzyl 4,4,5,5,6,6,6-heptafluoro-2-hydroxy-2-methylhexanoate (**5dh**)</u>

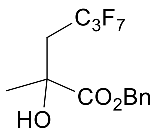

5dh

Yellow oil, 64.0 mg, yield: 71%. ^{1}H NMR (400 MHz, $CDCl_3$) δ: 7.40–7.33 (5H, m), 5.22 (2H, dd, *J* = 18.2, 12.1 Hz), 3.46 (1H, s), 2.78–2.66 (1H, m), 2.56–2.43 (1H, m), 1.52 (3H, s). ^{13}C NMR (151 MHz, $CDCl_3$) δ 175.1, 134.6, 129.0, 128.8 (2C), 128.7, 118.8–108.3 (3C, m), 71.5 (d, *J* = 1.5 Hz), 68.7, 39.1 (t, *J* = 19.6 Hz), 28.0. ^{19}F NMR (376 MHz, $CDCl_3$) δ: -80.8 (3F, s), -111.6 (1F, dd, *J* = 277.4, 23.1 Hz), -115.8 (1F, dd, *J* = 277.4, 23.1 Hz), -128.7 (2F, s). IR (neat, cm^{-1}) 3511, 2987, 1736, 1353, 1277, 1221, 1121, 971, 911, 726, 697. HRMS (ESI$^+$) calcd for $C_{14}H_{13}F_7O_3$ $[M+Na]^+$: 385.0645, found 385.0646.

<u>Benzyl 4,4,5,5,6,6,7,7,7-nonafluoro-2-hydroxy-2-methylheptanoate (**5di**)</u>

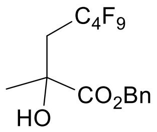

5di

Yellow oil, 75.5 mg, yield: 73%. ^{1}H NMR (400 MHz, $CDCl_3$) δ: 7.39–7.33 (5H, m), 5.23 (2H, dd, *J* = 15.1, 11.9 Hz), 3.47 (1H, s), 2.80–2.67 (1H, m), 2.57–2.43 (1H, m), 1.52 (3H, s). ^{13}C NMR (151 MHz, $CDCl_3$) δ 175.1, 134.7, 129.0, 128.8 (2C), 128.7, 119.5–108.6 (4C, m), 71.5 (d, *J* = 3.0 Hz), 68.7, 39.3 (t, *J* = 20.4 Hz), 28.0. ^{19}F NMR (376 MHz, $CDCl_3$) δ: -81.6 (3F, s), -110.7 (1F, d, *J* = 265.9 Hz), -114.9 (1F, d, *J* = 265.9 Hz), -125.3 (2F, s), 126.4 (2F, s). IR (neat, cm^{-1}) 3518, 2987, 1739, 1353, 1301, 1217,

1132, 911, 882, 735, 697. HRMS (ESI$^+$) calcd for $C_{15}H_{13}F_9O_3$ $[M+Na]^+$: 435.0613, found 435.0608.

Benzyl 4,4,5,5,6,6,7,7,8,8,8-undecafluoro-2-hydroxy-2-methyloctanoate (**5dj**)

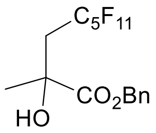

5dj

Yellow oil, 85.4 mg, yield: 74%. ^{1}H NMR (400 MHz, $CDCl_3$) δ: 7.41–7.33 (5H, m), 5.23 (2H, dd, J = 14.2, 11.9 Hz), 3.47 (1H, s), 2.80–2.67 (1H, m), 2.57–2.44 (1H, m), 1.52 (3H, s). ^{13}C NMR (151 MHz, $CDCl_3$) δ 175.1, 134.6, 129.0, 128.8 (2C), 128.7, 119.4–108.6 (5C, m), 71.5 (d, J = 3.0 Hz), 68.7, 39.4 (t, J = 20.4 Hz), 28.0. ^{19}F NMR (376 MHz, $CDCl_3$) δ: -81.2 (3F, s), -110.5 (1F, d, J = 289.0 Hz), -114.7 (1F, d, J = 289.0 Hz), -123.0 (2F, s), -124.6 (2F, s), 126.8 (2F, s). IR (neat, cm^{-1}) 3508, 2987, 1740, 1458, 1232, 1185, 1139, 874, 824, 697. HRMS (ESI$^+$) calcd for $C_{16}H_{13}F_{11}O_3$ $[M+Na]^+$: 485.0581, found 485.0581.

Benzyl 4,5,5,5-tetrafluoro-2-hydroxy-2-methyl-4-(trifluoromethyl)pentanoate (**5dk**)

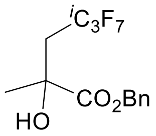

5dk

Yellow oil, 57.9 mg, yield: 64%. ^{1}H NMR (400 MHz, $CDCl_3$) δ: 7.41–7.33 (5H, m), 5.21 (2H, dd, J = 75.6, 11.9 Hz), 3.47 (1H, s), 2.78–2.72 (1H, m), 2.55–2.46 (1H, m), 1.51 (3H, s). ^{13}C NMR (151 MHz, $CDCl_3$) δ 175.0, 134.6, 129.0, 128.8, 128.7 (2C), 126.0-90.2 (3C, m), 71.6 (d, J = 4.5 Hz), 68.7, 37.0 (d, J = 18.1 Hz), 28.8. ^{19}F NMR (376 MHz, $CDCl_3$) δ: -76.0 (3F, s), -78.2 (3F, m), -187.4 (1F, s). IR (neat, cm^{-1}) 3518, 2987, 1739, 1277, 1224, 1155, 1129, 996, 960, 908, 696. HRMS (ESI$^+$) calcd for $C_{14}H_{13}F_7O_3$ $[M+Na]^+$: 385.0645, found 385.0639.

Benzyl 3-(1,2,2,3,3,4,4,5,5,6,6-undecafluorocyclohexyl)-2-hydroxy-2-methylpropanoate (**5dl**)

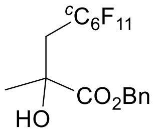

5dl

Yellow oil, 31.6 mg, yield: 27%. ^{1}H NMR (400 MHz, $CDCl_3$) δ: 7.41–7.33 (5H, m), 5.22 (2H, dd, J = 67.8, 12.4 Hz), 3.49 (1H, s), 2.85–2.79 (1H, m), 2.65–2.56 (1H, m), 1.54 (3H, s). ^{13}C NMR (151 MHz, $CDCl_3$) δ 175.0, 134.6, 129.0, 128.8 (3C), 119.1-90.5 (6C, m), 71.3 (d, J = 4.5 Hz), 68.7, 34.7 (d, J = 19.6 Hz), 28.9. ^{19}F NMR (376 MHz, $CDCl_3$) δ:-117.6 (1F, d, J = 300.06 Hz), -119.4 (1F, d, J = 300.06 Hz), -122.9 (2F, dd, J = 277.4, 173.4 Hz), -124.6 (1F, d, J = 277.4 Hz), -131.3 (1F, d, J = 300.06 Hz), -134.5 (1F, d, J = 300.06 Hz), -139.5 (2F, dd, J = 289.0, 57.8 Hz), -142.7 (1F, d, J = 289.0 Hz), -187.2 (1F, s).IR (neat, cm^{-1}) 3517, 2987, 1739, 1458, 1316, 1221, 1142, 983, 967, 904, 697. HRMS (ESI$^+$) calcd for $C_{17}H_{13}F_{11}O_3$ $[M+Na]^+$: 497.0581, found 497.0573.

Benzyl 5,5,5-trifluoro-2-hydroxy-2-methylpentanoate (**5dm**)

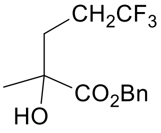

5dm

Yellow oil, 18.0 mg, yield: 26%. ^{1}H NMR (400 MHz, $CDCl_3$) δ: 7.42–7.34 (5H, m), 5.23 (2H, dd, *J* = 20.6, 12.4 Hz), 3.20 (1H, d, *J* = 0.9 Hz), 2.32-2.18 (1H, m), 2.06-1.99 (1H, m), 1.95-1.87 (1H, m), 1.86-1.76 (1H, m), 1.45 (3H, s). ^{13}C NMR (151 MHz, $CDCl_3$) δ 176.3, 135.0, 128.9 (2C), 128.5 (2C), 109.7-99.9 (m), 73.3, 68.2, 31.9 (q, *J* = 3.0 Hz), 28.7 (dd, *J* = 58.1, 29.4 Hz), 26.4. ^{19}F NMR (376 MHz, $CDCl_3$) δ: -67.0 (3F, s). IR (neat, cm^{-1}) 3507, 2958, 1729, 1456, 1320, 1221, 1144, 1078, 981, 750, 697. HRMS (ESI^+) calcd for $C_{13}H_{15}F_3O_3$ $[M+Na]^+$: 299.0865, found 299.0859.

Benzyl 5,5,6,6,7,7,7-heptafluoro-2-hydroxy-2-methylheptanoate (**5dn**)

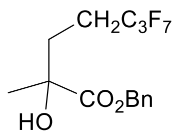

5dn

Colorless oil, 20.0 mg, yield: 21%. ^{1}H NMR (400 MHz, $CDCl_3$) δ: 7.41–7.34 (5H, m), 5.24 (2H, dd, *J* = 40.3, 12.4 Hz), 3.21 (1H, d, *J* = 0.9 Hz), 2.32–2.16 (1H, m), 2.10–2.03 (1H, m), 1.98–1.90 (1H, m), 1.85–1.68 (1H, m), 1.47 (3H, s). ^{13}C NMR (151 MHz, $CDCl_3$) δ 176.3, 135.0, 129.0, 128.9, 128.5 (2C), 119.4–108.7 (3C, m), 73.4, 68.2, 30.1 (t, *J* = 3.0 Hz), 26.5, 25.5 (t, *J* = 21.9 Hz). ^{19}F NMR (376 MHz, $CDCl_3$) δ: -80.6 (3F, s), -115.5 (2F, s), -127.8 (2F, s). IR (neat, cm^{-1}) 3502, 2953, 1732, 1458, 1354, 1217, 1171, 1076, 956, 908, 697. HRMS (ESI^+) calcd for $C_{15}H_{15}F_7O_3$ $[M+Na]^+$: 399.0802, found 399.0797.

3,3,4,4,5,5,6,6,7,7,8,8,8-Tridecafluoro-1-phenyloctan-1-ol (**7aa**)

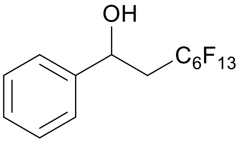

7aa

Yellow oil, 99.1 mg, yield: 89%. ^{1}H NMR (500 MHz, $CDCl_3$) δ: 7.42–7.32 (4H, m), 5.23 (1H, dd, *J* = 9.0, 3.5 Hz), 2.69–2.57 (1H, m), 2.48–2.37 (1H, m). ^{13}C NMR (151 MHz, $CDCl_3$) δ: 141.7, 128.1 (2C), 127.6, 124.8 (2C), 118.7–105.4 (6C, m), 67.1, 39.0 (t, *J* = 21.1 Hz). ^{19}F NMR (471 MHz, $CDCl_3$) δ: -81.3 (3F, s), -112.9 (1F, d, *J* = 286.1 Hz), -114.3 (1F, d, *J* = 286.1 Hz), -122.3 (2F, s), -123.4 (2F, s), -124.2 (2F, s), -126.7 (2F, s). IR (neat, cm^{-1}) 3397, 1319, 1233, 1142, 1121, 727, 698. HRMS (EI^+) calcd for $C_{14}H_9F_{13}O$ $[M]^+$: 440.0446, found 440.0452.

3,3,4,4,5,5,6,6,7,7,8,8,8-Tridecafluoro-1-(4-tolyl)octan-1-ol (**7ba**)

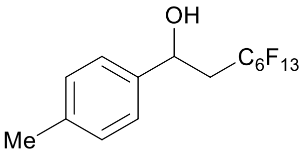

7ba

Yellow oil, 93.1 mg, yield: 82%. ^{1}H NMR (400 MHz, $CDCl_3$) δ: 7.29 (2H, d, *J* = 8.2 Hz), 7.20 (2H, d, *J* = 7.8 Hz), 5.20 (1H, dd, *J* = 8.7, 3.2 Hz), 2.70-2.55 (1H, m), 2.47–2.32 (1H, m), 2.36 (3H, s), 2.11 (1H, s). ^{13}C NMR (126 MHz, $CDCl_3$) δ: 139.8, 138.4, 130.2, 129.7, 125.7, 122.8, 122.8-108.7 (6C, m), 68.0, 39.9 (d, *J* = 20.4 Hz), 21.3. ^{19}F NMR (376 MHz, $CDCl_3$) δ: -81.3 (3F, s), -112.9 (1F, d, *J* = 138.7 Hz), -114.4 (1F, d, *J* = 138.7 Hz), -122.3 (2F,

s), -123.4 (2F, s), -124.1 (2F, s), -126.6 (2F, s). IR (neat, cm^{-1}) 3385, 1517, 1364, 1228, 1121, 1073, 921, 746, 704. HRMS (EI^+) calcd for $C_{15}H_{11}F_{13}O$ $[M]^+$: 454, 0602, found 454.0602.

3,3,4,4,5,5,6,6,7,7,8,8,8-Tridecafluoro-1-(4-methoxyphenyl)octan-1-ol (**7ca**)

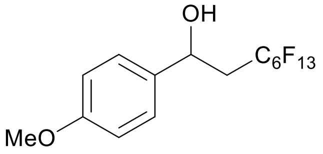

7ca

Yellow oil, 102.1 mg, yield: 87%. ^{1}H NMR (400 MHz, $CDCl_3$) δ: 7.31 (2H, d, *J* = 6.4 Hz), 6.91 (2H, d, *J* = 6.4 Hz), 5.17 (1H, dd, *J* = 8.7, 3.7 Hz), 3.81 (3H, s), 2.70–2.54 (1H, m), 2.47–2.32 (1H, m). ^{13}C NMR (126 MHz, $CDCl_3$) δ: 159.8, 135.0, 132.9-108.8 (6C, m), 127.1 (2C), 114.4 (2C), 67.7, 55.5, 39.9 (t, *J* = 21.0 Hz). ^{19}F NMR (376 MHz, $CDCl_3$) δ: -81.3 (3F, s), -113.0 (1F, d, *J* = 288.9 Hz), -114.4 (1F, d, *J* = 288.9 Hz), -122.3 (2F, s), -123.4 (2F, s), -124.1 (2F, s), -126.6 (2F, s). IR (neat, cm^{-1}) 3436, 2842, 1660, 1614, 1515, 1235, 1144, 1036, 834, 731. HRMS (EI^+) calcd for $C_{15}H_{11}F_{13}O_2$ $[M]^+$: 470.0551, found 470.0591.

1-(4-(*Tert*-butyl)phenyl)-3,3,4,4,5,5,6,6,7,7,8,8,8-tridecafluorooctan-1-ol (**7da**)

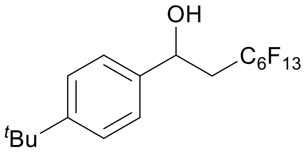

7da

Yellow oil, 96.7 mg, yield: 78%. ^{1}H NMR (400 MHz, $CDCl_3$) δ:7.43–7.40 (2H, m), 7.33–7.25 (2H, m), 5.20 (1H, dd, *J* = 10.0, 5.0 Hz), 2.69–2.56 (1H, m), 2.47–2.36 (1H, m), 2.18 (1H, s), 1.32 (9H, s). ^{13}C NMR (126 MHz, $CDCl_3$) δ: 151.7, 139.8, 130.2, 128.4, 126.0, 125.5, 119.7–109.4 (6C, m), 67.9, 39.9 (*J* = 20.4 Hz), 34.7, 31.4 (3C). ^{19}F NMR (471 MHz, $CDCl_3$) δ: -81.3 (3F, s), -112.9 (1F, d, *J* = 270.8 Hz), -114.5 (1F, d, *J* = 270.8 Hz), -122.3 (2F, s), -123.4 (2F, s), -124.1 (2F, s), -126.6 (2F, s). IR (neat, cm^{-1}) 3408, 2967, 2874, 1364, 1235, 1144, 1073, 836, 731, 702. HRMS (EI^+) calcd for $C_{18}H_{17}F_{13}O$ $[M]^+$: 496.1071, found 496.1085.

1-(Biphenyl-4-yl)-3,3,4,4,5,5,6,6,7,7,8,8,8-tridecafluorooctan-1-ol (**7ea**)

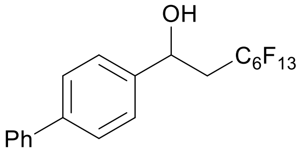

7ea

White solid, 104.9 mg, yield: 81%. ^{1}H NMR (400 MHz, $CDCl_3$) δ:7.62–7.57 (4H, m), 7.50–7.43 (4H, m), 7.38–7.34 (1H, m), 5.29–5.25 (1H, m), 2.74–2.56 (1H, m), 2.53–2.39 (1H, m), 2.29 (1H, d, *J* = 1.8 Hz). ^{13}C NMR (151 MHz, $CDCl_3$) δ:141.7, 141.6, 140.6, 132.6, 130.2, 129.0, 128.4, 127.8, 127.7, 127.3, 126.2, 120.2–108.4, 67.9, 40.0 (t, *J* = 20.4 Hz). ^{19}F NMR (471 MHz, $CDCl_3$) δ: -81.2 (3F, s), -112.8 (1F, d, *J* = 134.9 Hz), -114.2 (1F, d, *J* = 134.9 Hz), -122.2 (2F, s), -123.3 (2F, s), -124.0 (2F, s), -126.6 (2F, s). IR (neat, cm^{-1}) 3299, 1234, 1189, 1144, 1007, 839, 766, 692, 651. HRMS (ESI^+) calcd for $C_{20}H_{13}F_{13}O$ $[M+Na]^+$: 539.0651, found 539.0644.

1-(4-Chlorophenyl)-3,3,4,4,5,5,6,6,7,7,8,8,8-tridecafluorooctan-1-ol (**7fa**)

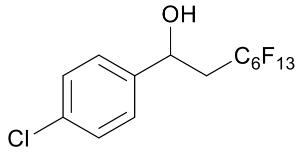

7fa

Yellow oil, 105.0 mg, yield: 88%. ^{1}H NMR (500 MHz, $CDCl_3$) δ: 7.36–7.34 (4H, m), 5.22 (1H, dt, *J* = 5.9, 2.8 Hz), 2.63–2.57 (1H, m), 2.44–2.33 (1H, m), 2.20 (1H, s). ^{13}C NMR (151 MHz, $CDCl_3$) δ: 141.1, 134.3, 129.3–106.9 (6C, m), 129.2 (2C), 127.2 (2C), 129.7–106.9 (6C, m), 67.5, 40.0 (t, *J* = 21.1 Hz). ^{19}F NMR (471 MHz, $CDCl_3$) δ: -81.3 (3F, s), -113.2 (1F, d, *J* = 136.2 Hz), -114.4 (1F, d, *J* = 136.2 Hz), -122.3 (2F, s), -123.4 (2F, s), -124.1 (2F, s), -126.7 (2F, s). IR (neat, cm^{-1}) 3385, 3069, 2984, 2903, 1233, 1190, 1142, 1121, 1092, 812, 700. HRMS (EI^{+}) calcd for $C_{14}H_8ClF_{13}O$ $[M]^{+}$: 474.0056, found 474.0043.

3,3,4,4,5,5,6,6,7,7,8,8,8-Tridecafluoro-1-(4-fluorophenyl)octan-1-ol (**7ga**)

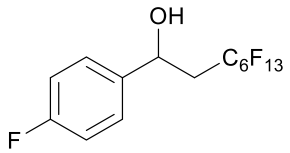

7ga

Yellow oil, 100.0 mg, yield: 87%. ^{1}H NMR (500 MHz, $CDCl_3$) δ: 7.38 (2H, m), 7.10–7.06 (2H, m), 5.22 (1H, dd, *J* = 8.7, 3.3 Hz), 2.67–2.55 (1H, m), 2.45-2.34 (1H, m). ^{13}C NMR (151 MHz, $CDCl_3$) δ: 162.7 (d, *J* = 247.5 Hz), 138.5 (d, *J* = 3.0 Hz), 127.6 (2C, d, *J* = 9.0 Hz), 119.6–110.3 (6C, m), 115.9 (2C, d, *J* = 22.6 Hz), 67.5, 40.1 (t, *J* = 21.1 Hz). ^{19}F NMR (471 MHz, $CDCl_3$) δ: -81.3 (3F, s), -113.2 (1F, d, *J* = 286.1 Hz), -114.4 (1F, d, *J* = 286.1 Hz), -114.0 (1F, s), -122.3 (2F, s), -123.4 (2F, s), -124.1 (2F, s), -126.7 (2F, s). IR (neat, cm^{-1}) 3405, 2357,1670, 1607, 1229, 1188, 1142, 1121, 837, 704. HRMS (EI^{+}) calcd for $C_{14}H_8F_{14}O$ $[M]^{+}$: 458.0352, found 458.0394.

3,3,4,4,5,5,6,6,7,7,8,8,8-Tridecafluoro-1-(4-(trifluoromethyl)phenyl)octan-1-ol (**7ha**)

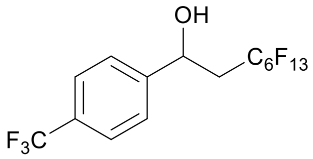

7ha

Yellow oil, 94.0 mg, yield: 74%. ^{1}H NMR (500 MHz, $CDCl_3$) δ: 7.66 (2H, d, *J* = 8.0 Hz), 7.54 (2H, d, *J* = 8.0 Hz), 5.31 (1H, d, *J* = 8.6 Hz), 2.68–2.56 (1H, m), 2.48–2.36 (1H, m), 2.28 (1H, s). ^{13}C NMR (151 MHz, $CDCl_3$) δ: 146.3, 132.6, 130.8 (dd, *J* = 64.9, 33.2 Hz), 130.2, 128.3, 126.2, 126.0 (q, *J* = 3.0 Hz), 121.3–106.4 (6C, m), 67.5, 40.1 (t, *J* = 21.1 Hz). ^{19}F NMR (471 MHz, $CDCl_3$) δ: -63.2 (3F, s), -81.3 (3F, s), -112.9 (1F, d, *J* = 277.9 Hz), -114.0 (1F, d, *J* = 277.9 Hz), -122.2 (2F, s), -123.3 (2F, s), -124.1 (2F, s), -126.6 (2F, s). IR (neat, cm^{-1}) 3374, 3038, 1669, 1622, 1325, 1235, 1167, 1123, 1069, 845, 696. HRMS (EI^{+}) calcd for $C_{15}H_8F_{16}O$ $[M]^{+}$: 508.0320, found 508.0271.

3,3,4,4,5,5,6,6,7,7,8,8,8-Tridecafluoro-1-(4-nitrophenyl)octan-1-ol (**7ia**)

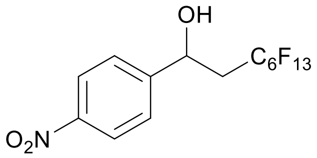

7ia

Yellow oil, 47.9 mg, yield: 39%. ^{1}H NMR (500 MHz, $CDCl_3$) δ: 8.28–8.24 (2H, m), 7.60 (2H, d, *J* = 9.2 Hz), 5.39–5.35 (1H, m), 2.71–2.56 (1H, m), 2.49-2.36 (2H, m). ^{13}C NMR (151 MHz, $CDCl_3$) δ: 149.6, 147.9, 126.8 (2C), 124.3 (2C), 120.1–108.4 (6C, m), 67.2, 40.1 (t, *J* =

21.1 Hz). ^{19}F NMR (471 MHz, $CDCl_3$) δ: -81.3 (3F, s), -112.8 (1F, d, *J* = 272.5 Hz), -113.7 (1F, d, *J* = 272.5 Hz), -122.2 (2F, s), -123.3 (2F, s), -124.0 (2F, s), -126.6 (2F, s). IR (neat, cm^{-1}) 3424, 2899, 1601, 1518, 1352, 1233, 1188, 1142, 1123, 855, 694. HRMS (EI^+) calcd for $C_{14}H_8F_{13}NO_3$ $[M]^+$: 485.0297, found 485.0250.

<u>1-(2-Chlorophenyl)- 3,3,4,4,5,5,6,6,7,7,8,8,8-tridecafluorooctan-1-ol (**7ja**)</u>

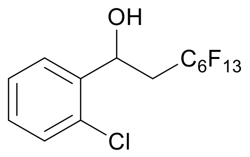

7ja

Yellow oil, 86.4 mg, yield: 73%. ^{1}H NMR (500 MHz, $CDCl_3$) δ: 7.66 (1H, dd, *J* = 7.7, 1.7 Hz), 7.36 (2H, ddd, *J* = 15.8, 7.7, 1.4 Hz), 7.27 (1H, td, *J* = 7.7, 1.7 Hz), 5.65 (1H, dd, *J* = 9.2, 1.4 Hz), 2.59-2.39 (2H, m), 2.31 (1H, s). ^{13}C NMR (151 MHz, $CDCl_3$) δ: 139.9, 131.3, 129.8, 129.5, 127.6, 127.1, 120.0-106.6 (6C, m), 64.8 (d, *J* = 4.5 Hz), 38.4 (t, *J* = 21.1 Hz). ^{19}F NMR (471 MHz, $CDCl_3$) δ: -81.3 (3F, s), -112.8 (1F, d, *J* = 272.5 Hz), -115.1 (1F, d, *J* = 272.5 Hz), -122.3 (2F, s), -123.4 (2F, s), -124.2 (2F, s), -126.6 (2F, s). IR (neat, cm^{-1}) 3462, 3065, 3003, 2971, 2947, 1231, 1204, 1142, 1074, 754, 702. HRMS (EI^+) calcd for $C_{14}H_8ClF_{13}O$ $[M]^+$: 474.0056, found 474.0029.

<u>1-(3-Chlorophenyl)- 3,3,4,4,5,5,6,6,7,7,8,8,8-tridecafluorooctan-1-ol (**7ka**)</u>

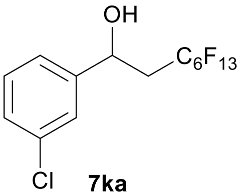

7ka

Yellow oil, 95.0 mg, yield: 80%. ^{1}H NMR (500 MHz, $CDCl_3$) δ: 7.42 (1H, t, *J* = 1.7 Hz), 7.36–7.27 (3H, m), 5.22 (1H, dd, *J* = 8.9, 4.4 Hz), 2.63–2.57 (1H, m), 2.42–2.38 (1H, m), 2.24 (1H, s). ^{13}C NMR (151 MHz, $CDCl_3$) δ: 144.5, 135.0, 130.4, 128.7, 126.0, 123.9, 120.2–108.5 (6C, m), 67.5, 40.1 (t, *J* = 21.1 Hz). ^{19}F NMR (471 MHz, $CDCl_3$) δ: -81.3 (3F, s), -113.2 (1F, d, *J* = 140.8 Hz), -114.4 (1F, d, *J* = 140.8 Hz), -122.3 (2F, s), -123.4 (2F, s), -124.1 (2F, s), -126.6 (2F, s). IR (neat, cm^{-1}) 3401, 3065, 2978, 2895, 1669, 1233, 1188, 1142, 1074, 812, 694. HRMS (EI^+) calcd for $C_{14}H_8ClF_{13}O$ $[M]^+$: 474.0056, found 474.0011.

<u>3,3,4,4,5,5,6,6,7,7,8,8,8-Tridecafluoro-1-(2,3,4,5,6-pentafluorophenyl)octan-1-ol (**7la**)</u>

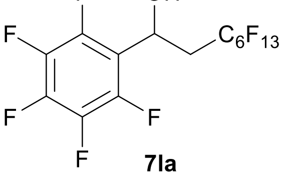

7la

Yellow oil, 83.3 mg, yield: 63%. ^{1}H NMR (500 MHz, $CDCl_3$) δ: 5.60 (1H, dd, *J* = 12.9, 6.6 Hz), 3.01–2.89 (1H, m), 2.73–2.61 (1H, m), 2.44 (1H, d, *J* = 6.6 Hz). ^{13}C NMR (151 MHz, $CDCl_3$) δ: 145.8–135.1 (5C, m), 132.6, 118.2–110.3 (6C, m), 59.5, 37.3 (t, *J* = 21.1 Hz). ^{19}F NMR (471 MHz, $CDCl_3$) δ: -81.5 (3F, s), -113.8 (1F, d, *J* = 272.5 Hz), -114.6 (1F, d, *J* = 272.5 Hz), -122.5 (2F, s), -123.6 (2F, s), -124.3 (2F, s), -126.9 (2F, s), -143.9 (2F, s), -153.3 (1F, t, *J* = 21.6 Hz), -161.4 (2F, t, *J* = 21.6 Hz). IR (neat, cm^{-1}) 3495, 1505, 1233, 1190, 1142, 1121. HRMS (EI^+) calcd for $C_{14}H_4F_{18}O$ $[M]^+$: 529.9975, found 529.9951.

4,4,5,5,6,6,7,7,8,8,9,9,9-Tridecafluoro-2-phenylnonan-2-ol (**7ma**)

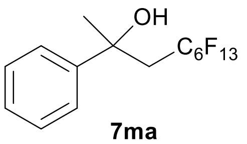

7ma

Yellow oil, 102.3 mg, yield: 90%. ^{1}H NMR (400MHz, $CDCl_3$) δ: 7.50–7.25 (5H, m), 2.74–2.43 (2H, m), 1.76 (3H, d, *J* = 4.0 Hz). ^{13}C NMR (126 MHz, $CDCl_3$) δ: 146.6, 128.5 (2C), 127.5, 124.4 (2C), 118.7–105.7 (6C, m), 72.9, 42.6 (t, *J* = 19.8 Hz), 30.2. ^{19}F NMR (376MHz, $CDCl_3$) δ: -81.3 (3F, s), -110.9 (1F, d, *J* = 274.6 Hz), -113.5 (1F, d, *J* = 274.6 Hz), -122.2 (2F, s), -123.4 (2F, s), -124.2 (2F, s), -126.7 (2F, s). IR (neat, cm^{-1}) 2953, 2918, 2851, 1235, 1190, 1167, 1145, 1121, 698. HRMS (EI^{+}) calcd for $C_{15}H_{11}F_{13}O$ $[M]^{+}$: 454.0602, found 454.0595.

2-(4-Chlorophenyl)-4,4,5,5,6,6,7,7,8,8,9,9,9-tridecafluorononan-2-ol (**7na**)

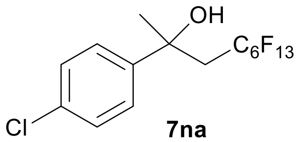

7na

Yellow oil, 98.0 mg, yield: 80%. ^{1}H NMR (500 MHz, $CDCl_3$) δ: 7.42 (2H, d, *J* = 8.7 Hz), 7.34 (2H, d, *J* = 8.7 Hz), 2.71–2.48 (2H, m), 2.28 (1H, s), 1.75 (3H, s). ^{13}C NMR (151 MHz, $CDCl_3$) δ: 145.0, 133.4, 132.6, 130.2, 128.7, 128.4, 126.1, 120.3–108.4 (6C, m), 72.7, 42.6 (t, *J* = 19.6 Hz), 30.5. ^{19}F NMR (471 MHz, $CDCl_3$) δ: -81.3 (6F, s), -110.9 (1F, d, *J* = 271.7 Hz), -113.4 (1F, d, *J* = 271.7 Hz), -122.2 (2F, s), -123.4 (2F, s), -124.2 (2F, s), -126.7 (2F, s). IR (neat, cm^{-1}) 3464, 2984, 1491, 1233, 1190, 1165, 1142, 1119, 1013, 831. HRMS (EI^{+}) calcd for $C_{15}H_{10}ClF_{13}O$ $[M]^{+}$: 488.0213, found 488.0188.

3,3,4,4,5,5,6,6,7,7,8,8,8-Tridecafluoro-1,1-diphenyloctan-1-ol (**7oa**)

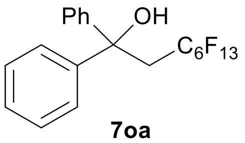

7oa

Yellow oil, 89.3 mg, yield: 69%. ^{1}H NMR (400 MHz, $CDCl_3$) δ: 7.45–7.42 (4H, m), 7.36–7.31 (4H, m), 7.28–7.24 (2H, m), 3.17 (2H, t, *J* = 18.3 Hz), 2.74 (1H, t, *J* = 2.1 Hz). ^{13}C NMR (151 MHz, $CDCl_3$) δ: 145.5 (2C), 137.7, 132.6, 130.2, 128.6 (2C), 128.4, 127.7 (2C), 125.5 (2C), 120.1-108.4 (6C, m), 76.6, 41.0 (t, *J* = 19.6 Hz). ^{19}F NMR (376 MHz, $CDCl_3$) δ: -81.3 (3F, s), -109.5 (2F, s), -122.1 (2F, s), -123.3 (2F, s), -124.1 (2F, s), -126.6 (2F, s). IR (neat, cm^{-1}) 3470, 3088, 3061, 2971, 3028, 1655, 1495, 1233, 1188, 1142, 1121, 812, 696. HRMS (ESI^{-}) calcd for $C_{20}H_{13}F_{13}O$ $[M-H]^{-}$: 515.0681, found 515.0641.

3,3,4,4,5,5,6,6,7,7,8,8,8-Tridecafluoro-1-(naphthalen-2-yl)octan-1-ol (**7pa**)

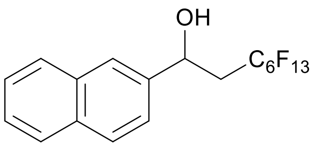

7pa

White solid, 69.6 mg, yield: 56%. ^{1}H NMR (400 MHz, $CDCl_3$) δ: 7.89–7.79 (4H, m), 7.53–7.25 (3H, m), 5.41–5.37 (1H, m), 2.79–2.63 (1H, m), 2.57–2.44 (1H, m), 2.34 (1H, d, *J* = 11.8 Hz). ^{13}C NMR (151 MHz, $CDCl_3$) δ: 140.1, 133.3 (d, *J* = 3.0 Hz), 130.2, 129.1, 128.4, 128.2, 127.9. 126.6 (d, *J* = 24.2 Hz), 124.8, 123.4, 119.7–108.4 (6C, m), 68.2, 40.0 (t, *J* = 21.1 Hz). ^{19}F NMR (376 MHz, $CDCl_3$) δ: -81.3 (3F, s), -112.8 (1F, d, *J* = 129.1 Hz), -

114.2 (1F, d, J = 129.1 Hz), -122.2 (2F, s), -123.3 (2F, s), -124.1 (2F, s), -126.6 (2F, s). IR (neat, cm^{-1}) 3321, 1364, 1234, 1194, 1144, 1073, 1017, 826, 745, 692. HRMS (EI$^+$) calcd for $C_{18}H_{11}F_{13}O$ [M]$^+$: 490.0602, found 490.0620.

2-(1,1,2,2,3,3,4,4,5,5,6,6,6-Tridecafluorohexyl)-1,2,3,4-tetrahydronaphthalen-1-ol (**7qa**)

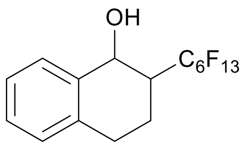

7qa

(84: 16 diastereomer mixture, dr was measured by HPLC)

Yellow oil, 75.2 mg, yield: 55%. ^{1}H NMR (500 MHz, $CDCl_3$) δ: 7.51–7.12 (major and minor, 4H, m), 5.14 (major, 1H, d, J = 6.5 Hz), 5.11 (minor, 1H, s), 2.92–2.75 (major and minor, 3H, m), 2.33–2.01 (major and minor, 2H, m) , 1.92–1.84 (major and minor, 1H, m). ^{13}C NMR (151 MHz, $CDCl_3$) δ: 136.7 (minor), 136.4 (major), 132.6 (minor), 130.2 (major), 129.3 (minor), 129.0 (minor), 128.8 (major), 128.5 (major), 128.4 (minor), 128.2 (major), 127.0 (major), 126.8 (minor), 121.0–106.9 (major and minor, 6C, m), 66.9 (major), 66.1 (minor), 45.6 (major, t, J = 19.6 Hz), 42.4 (minor, t, J = 19.6 Hz), 28.5 (minor), 27.5 (major), 20.6 (major), 16.2 (minor). ^{19}F NMR (471 MHz, $CDCl_3$) δ: -81.3 (3F, s), -113.8 (major, 1F, d, J = 283.3 Hz), -114.7 (minor, 1F, d, J = 283.3 Hz), -116.2 (minor, 1F, d, J = 283.3 Hz), -117.3 (major, 1F, d, J = 283.3 Hz), -120.6–-1234.0 (6F, m), -125.8–-127.3 (2F, m). IR (neat, cm^{-1}) 3447, 3063, 3013, 2986, 2936, 2884, 1491, 1451, 1227, 1190, 1179, 1142, 1121, 1045, 743. HRMS (EI$^+$) calcd for $C_{16}H_{11}F_{13}O$ [M]$^+$: 466.0602, found 466.0565. HPLC: GL Science Inc. Inertsil® Diol column; detected at 294 nm; hexane/ethanol, 95/5; flow = 1.0 mL/min; retention times: 6.20 min, 8.65 min.

3,3,4,4,5,5,6,6,7,7,8,8,8-Tridecafluoro-2-methyl-1-phenyloctan-1-ol (**7ra**)

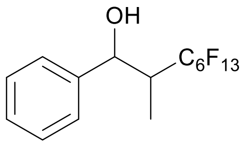

7ra

(76: 24 diastereomer mixture, dr was measured by HPLC.)

Yellow oil, 21.9 mg, yield: 19%. ^{1}H NMR (500 MHz, $CDCl_3$) δ: 7.47–7.27 (major and minor, 5H, m), 5.44 (major, 1H, s), 5.04 (minor, 1H, dd, J = 6.7, 1.0 Hz), 2.88–2.79 (minor, 1H, m), 2.62–2.51 (major, 1H, m), 2.15 (minor, 1H, s), 1.95 (major, 1H, d, J = 3.2 Hz), 1.08 (major, 3H, dd, J = 7.0, 1.0 Hz), 0.91 (minor, 3H, d, J = 6.9 Hz). ^{13}C NMR (151 MHz, $CDCl_3$) δ: 141.6 (major), 140.9 (minor), 128.7 (minor, 2C), 128.6 (major, 2C), 127.8 (major), 127.2 (minor), 125.6 (major, 2C), 125.5 (minor, 2C), 119.5–109.2 (major and minor, 6C, m), 73.1 (minor, d, J = 4.5 Hz), 69.7 (major, d, J = 4.5 Hz), 43.4 (major and minor, t, J = 19.6 Hz), 10.1 (minor), 5.3 (major). ^{19}F NMR (471 MHz, $CDCl_3$) δ: -81.3 (3F, s), -112.1 (minor, 1F, d, J = 286.1 Hz), -114.9 (major, 1F, d, J = 272.5 Hz), -116.8 (major, 1F, d, J = 272.5 Hz), -117.3 (minor, 1F, d, J = 286.1 Hz), -120.1-123.3 (6F, m), -124.1--127.3 (2F, m). IR (neat, cm^{-1}) 3464, 3065, 3028, 2998, 2970, 1670, 1233, 1194, 1142, 1123, 746, 698. HRMS (EI$^+$) calcd for $C_{15}H_{11}F_{13}O$ [M]$^+$: 454.0602, found 454.0560. HPLC: GL Science Inc. Inertsil® Diol column; detected at 294 nm; hexane/ethanol, 95/5; flow = 1.0 mL/min; retention times: 4.44 min, 4.77 min.

8. References

1. E. He. Min, K. H. Wong, E. Setijadi, F. Ladouceur, M. Straton and A. Argyros, *Polym. Chem.*, 2011, **2**, 2045–2051.
2. N. S. Sheikh, *RSC Adv.*, 2015, **5**, 3941−3953.
3. T. Yajima, C. Saito and H. Nagano, *Tetrahedron*, 2005, **61**, 10203–10215.
4. Y. Xiao, Y.-K. Chun, S.-C. Cheng, C.-O. Ng, M.-K. Tse, N.-Y. Lei, R. Liu and C.-C. Ko, *Catal. Sci. Technol*., 2021, **11**, 556–562.
5. G. Bélanger, M. Doré, F. Ménard and V. Darsigny, *J. Org. Chem.*, 2006, **71**, 7481–7484.
6. (a) T. Yajima, K. Yamaguchi, R. Hirokane and E. Nogami, *J. Fluorine Chem.*, 2013, **150**, 1–7; (b) H. Schedel, W. Dmowski and K. Burger, *Synthesis*, 2000, **12**, 1681–1688.
7. (a) X. Sun, W. Wang, Y. Li, J. Ma and S. Yu, *Org. Lett*., 2016, **18**, 4638–4641; (b) Y. Wang, J. Wang, G.-X. Li, G. He and G. Chen, *Org. Lett*., 2017, **19**, 1442–1445.
8. P. Job, *Justus Liebigs Ann. Chem*. 1928, **9**, 113–203.
9. (a) P. Berkeley and M. W. Hanna, *J. Physic. Chem*. 1963, **67**, 846–849; (b) M. W. Hanna and A. L. Ashbaugh. *J. Physic. Chem*. 1964, 68, 811–816; (c) R. Foster and C. A. Fyfe, *Trans. Faraday Soc.* 1965, 61, 1626–1631.
10. Y. Shen, N. Lei, C. Lu, D. Xi, X. Geng, P. Tao, Z. Su and K. Zheng, *Chem. Sci.*, 2021, **12**, 15399–15406.

9. [1] H, ^{13}C and ^{19}F NMR spectra

5aa: ^{1}H NMR ($CDCl_3$, 400 MHz)

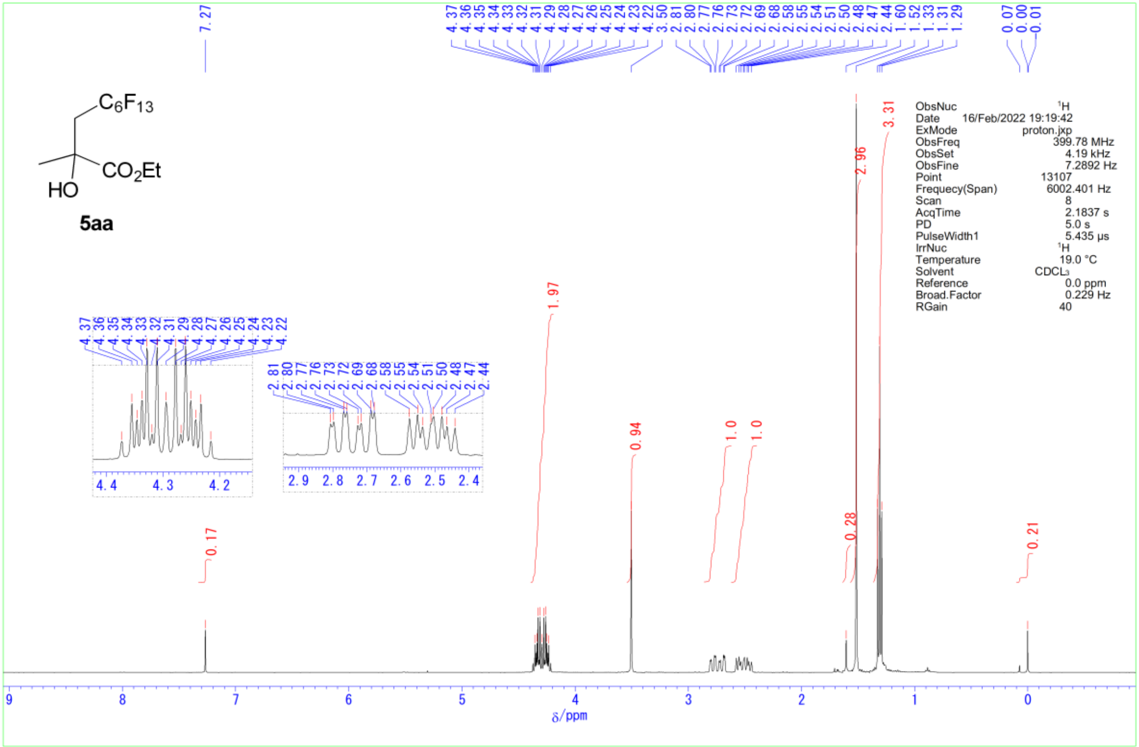

5aa: ^{13}C NMR ($CDCl_3$, 151 MHz)

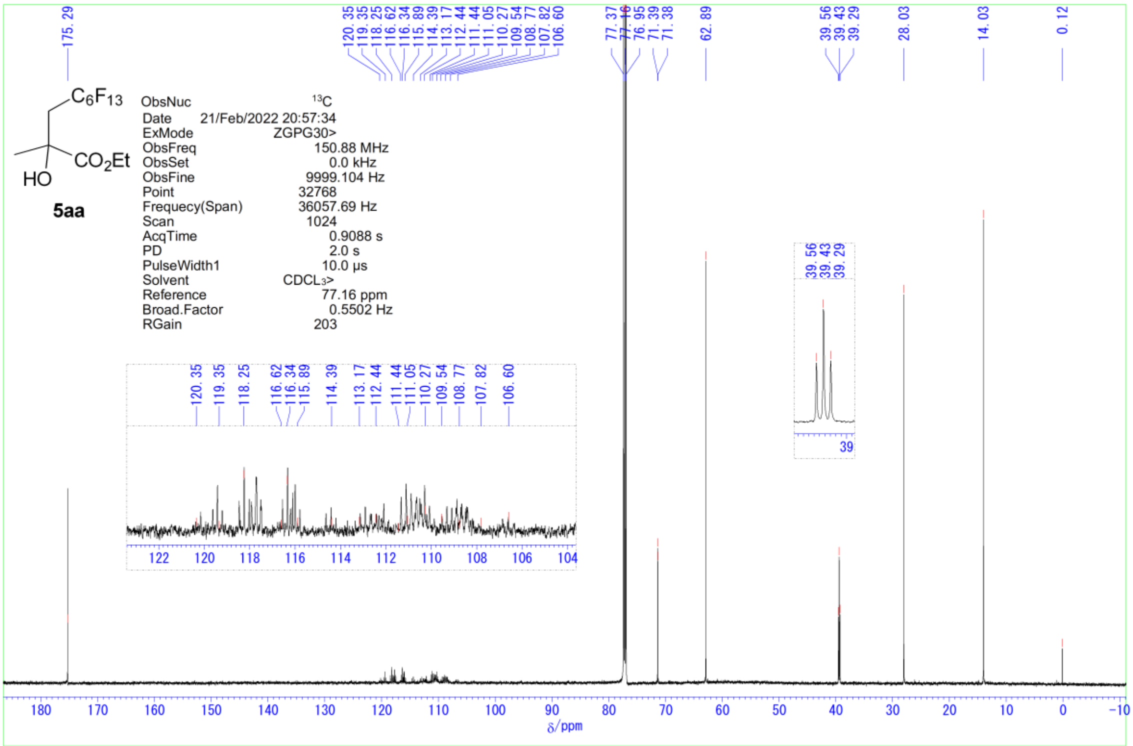

5aa: ^{19}F NMR ($CDCl_3$, 376 MHz)

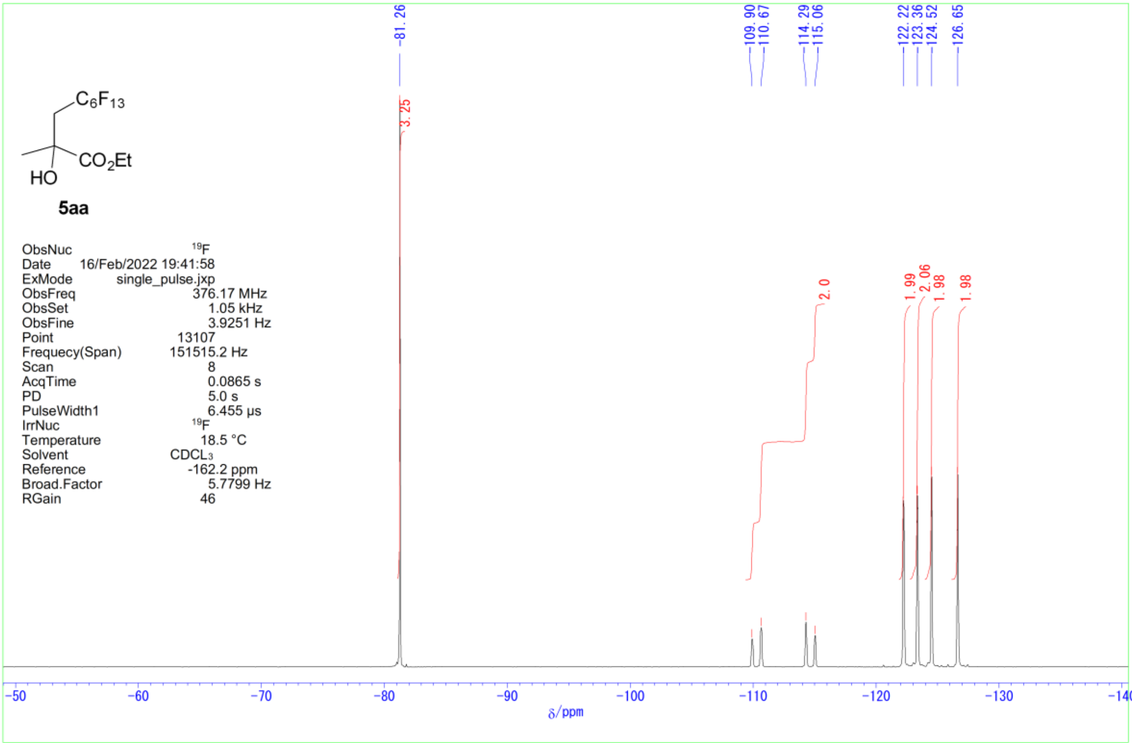

5ba: ^{1}H NMR ($CDCl_3$, 500 MHz)

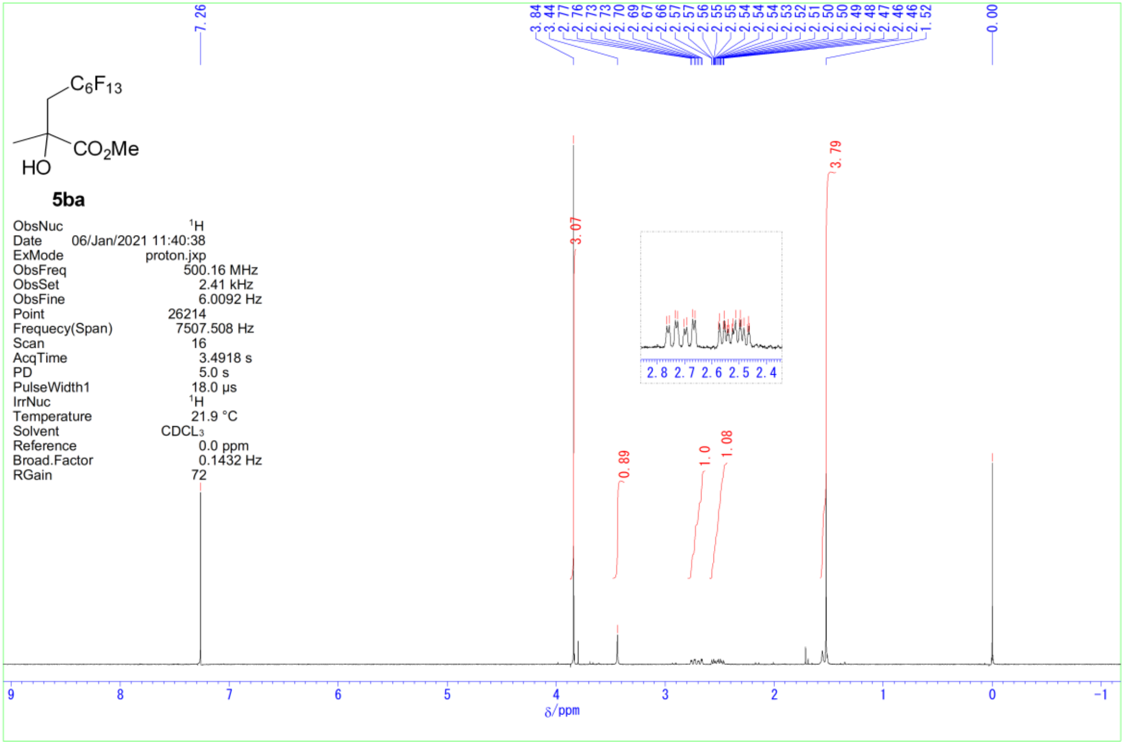

5ba: ^{13}C NMR ($CDCl_3$, 151 MHz)

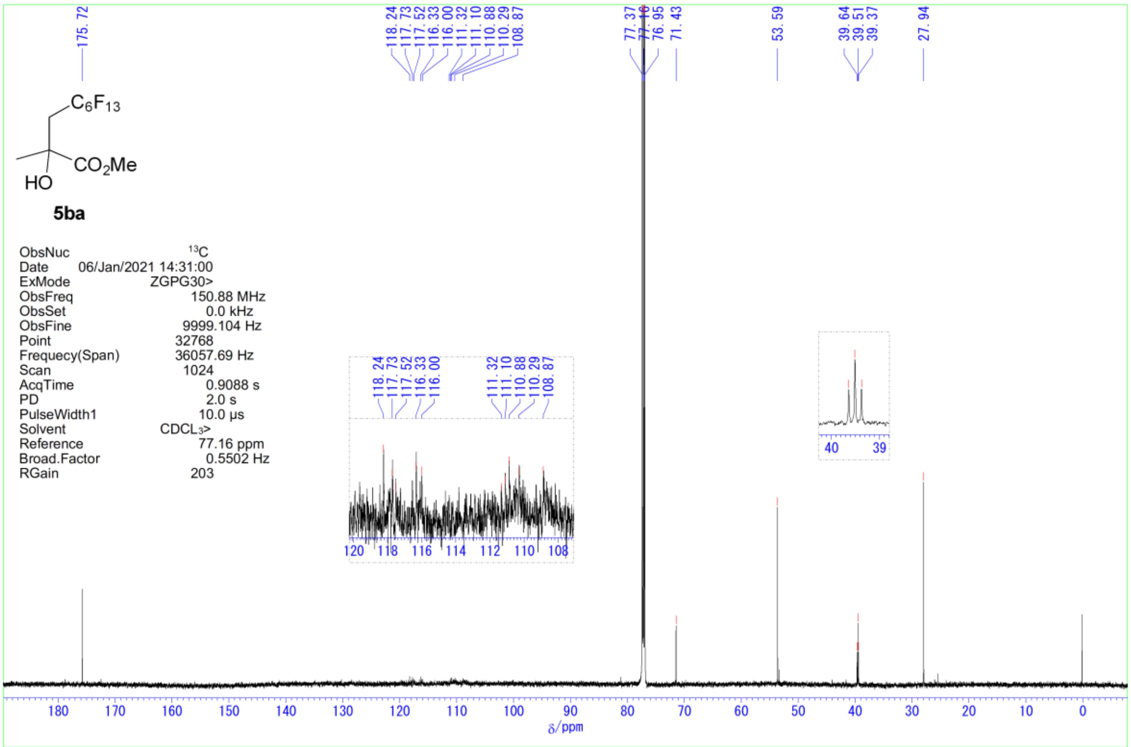

5ba: ^{19}F NMR ($CDCl_3$, 471 MHz)

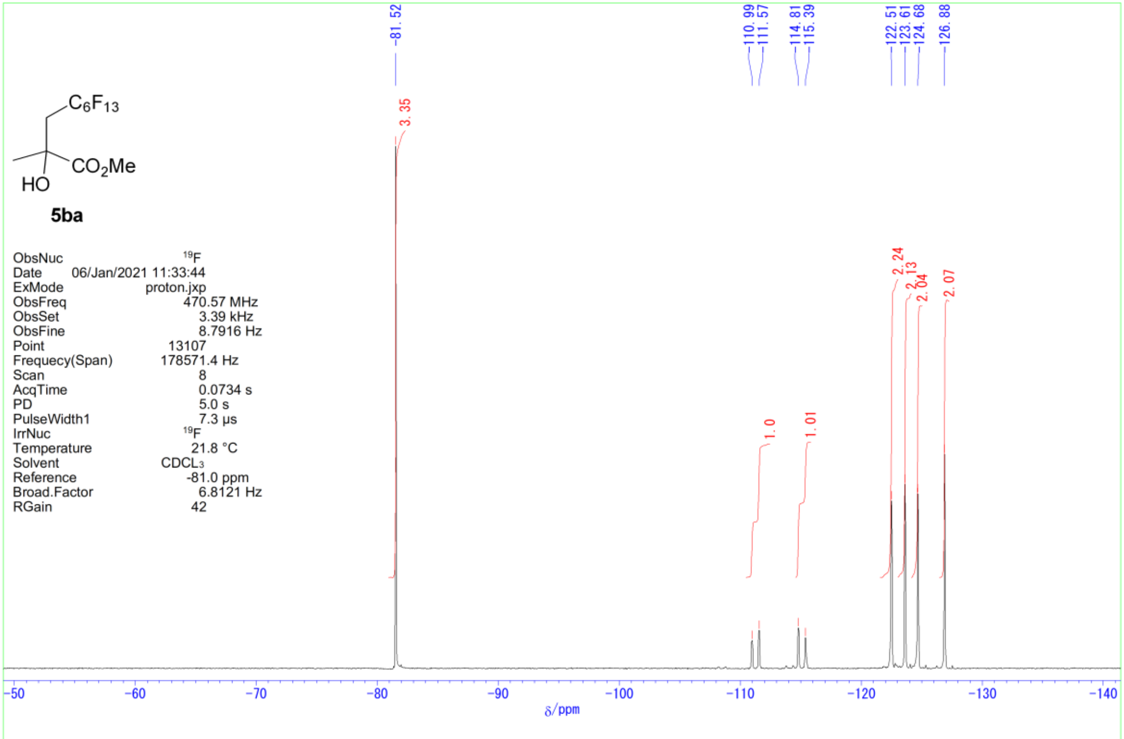

5ca: ^{1}H NMR ($CDCl_3$, 500 MHz)

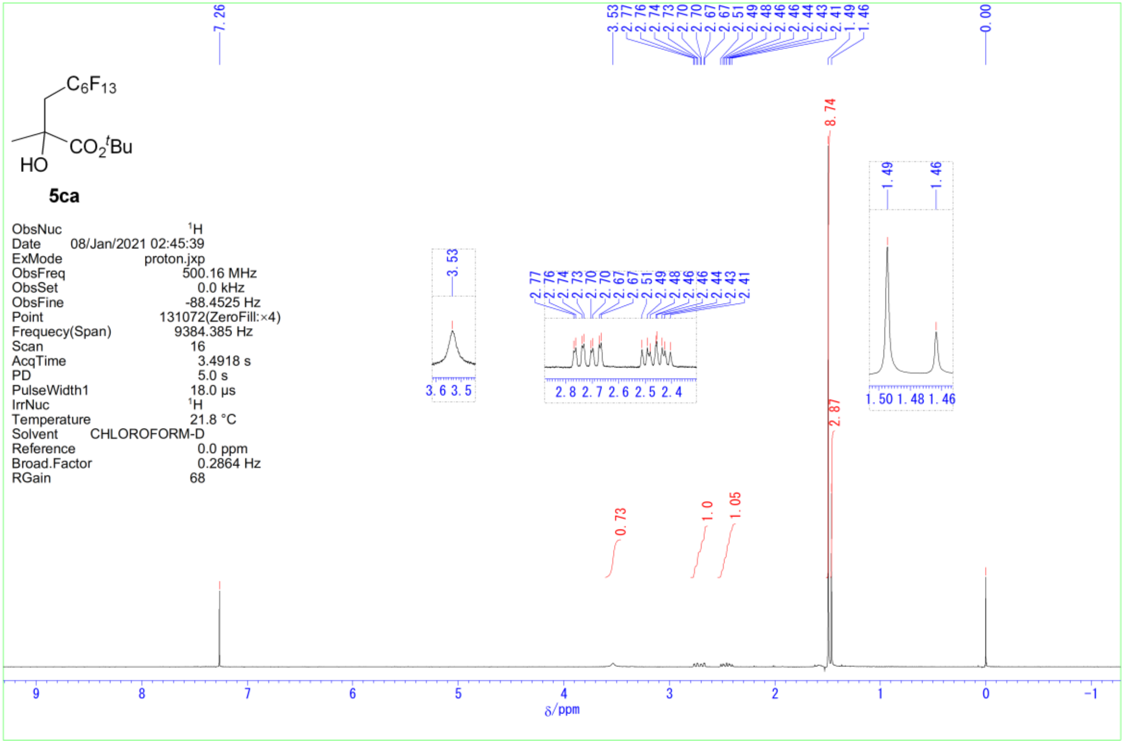

5ca: ^{13}C NMR ($CDCl_3$, 151 MHz)

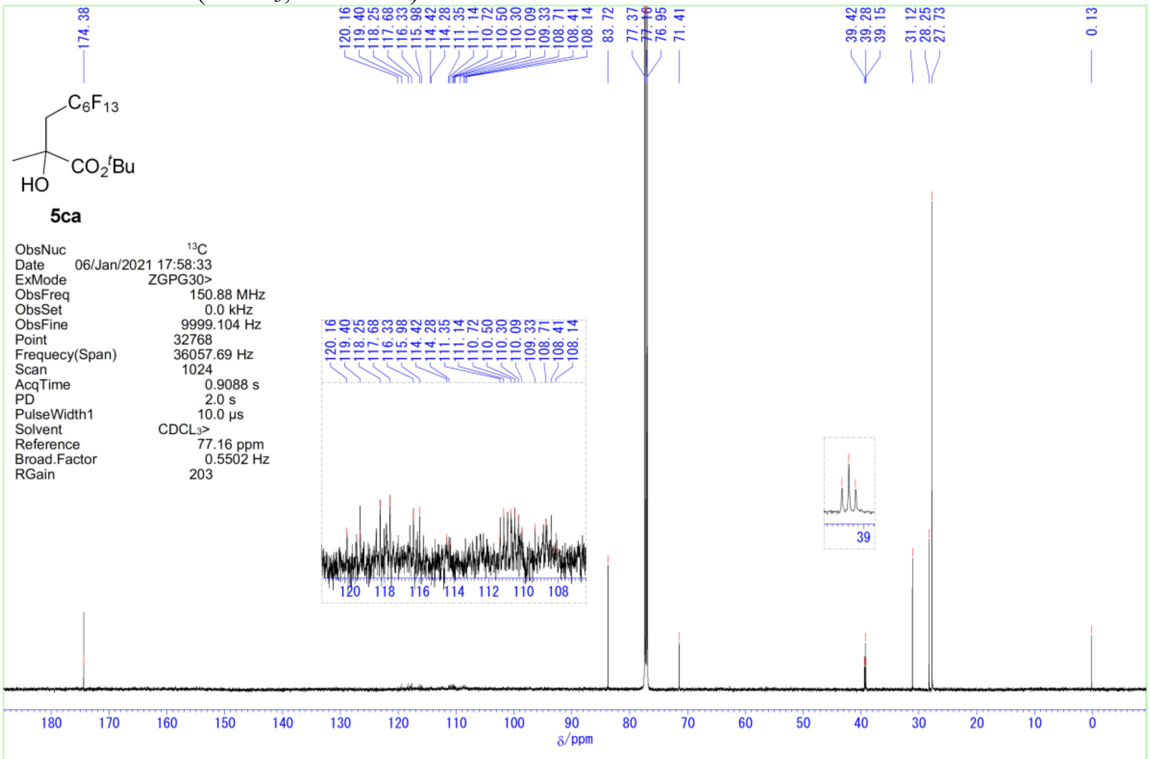

5ca: ^{19}F NMR (CDCl$_3$, 471 MHz)

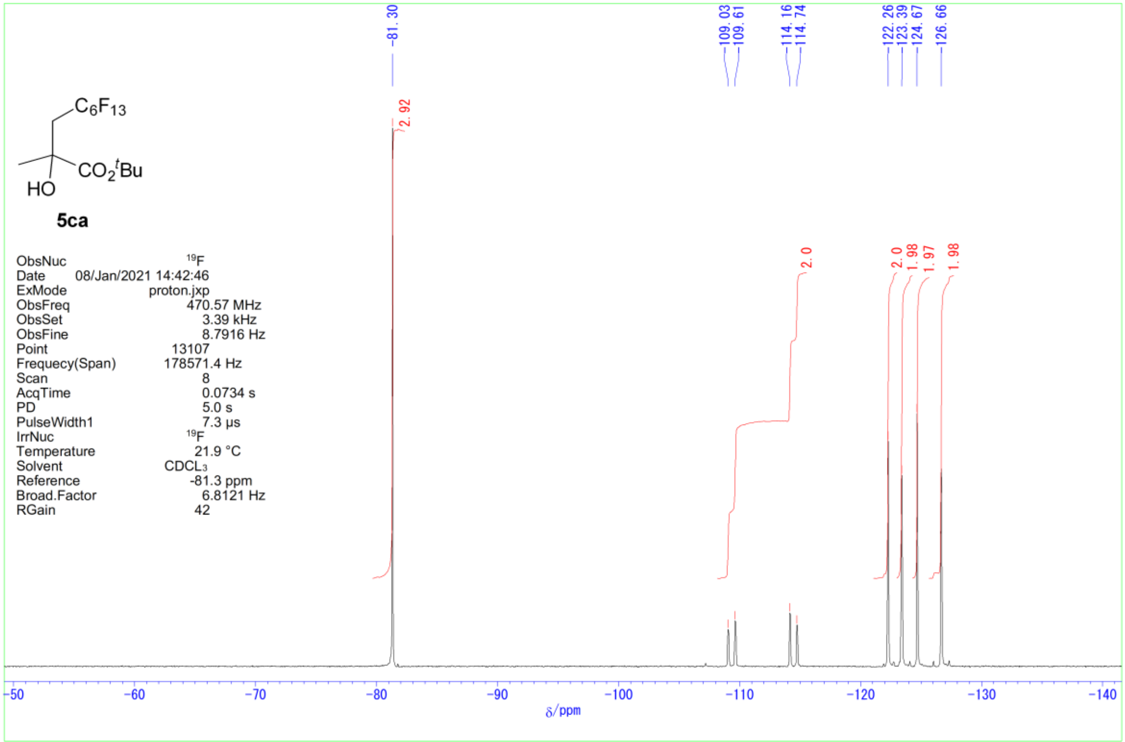

5da: ^{1}H NMR (CDCl$_3$, 500 MHz)

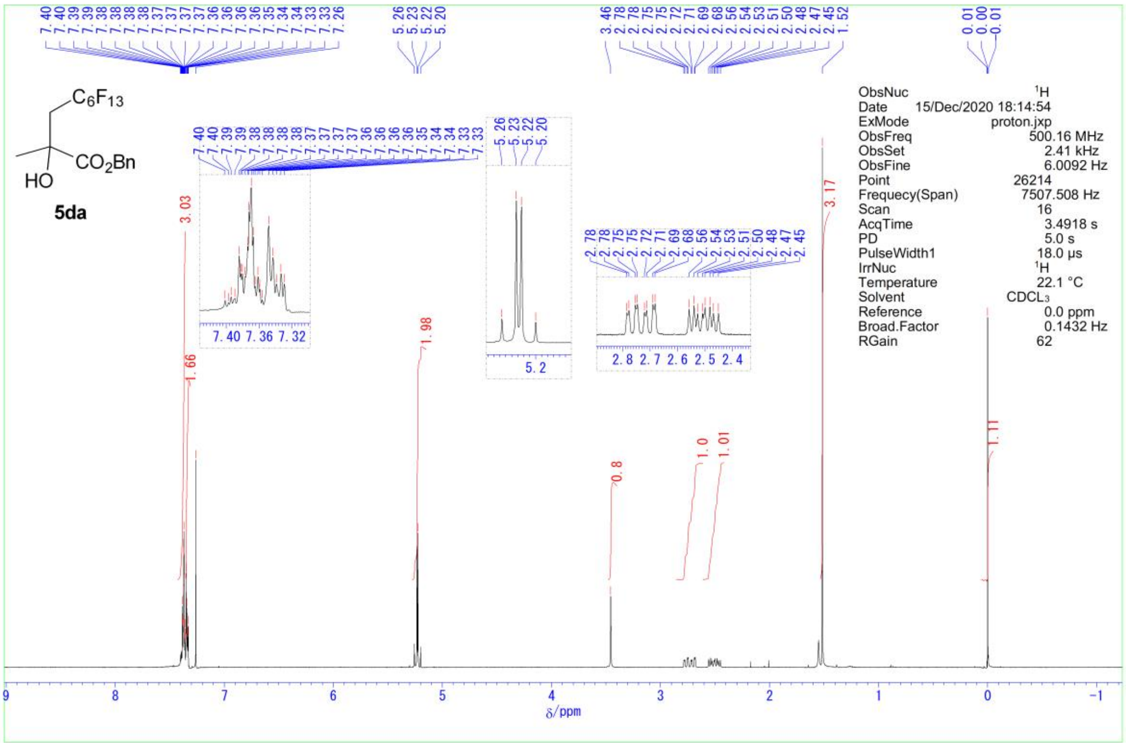

5da: ^{13}C NMR ($CDCl_3$, 151 MHz)

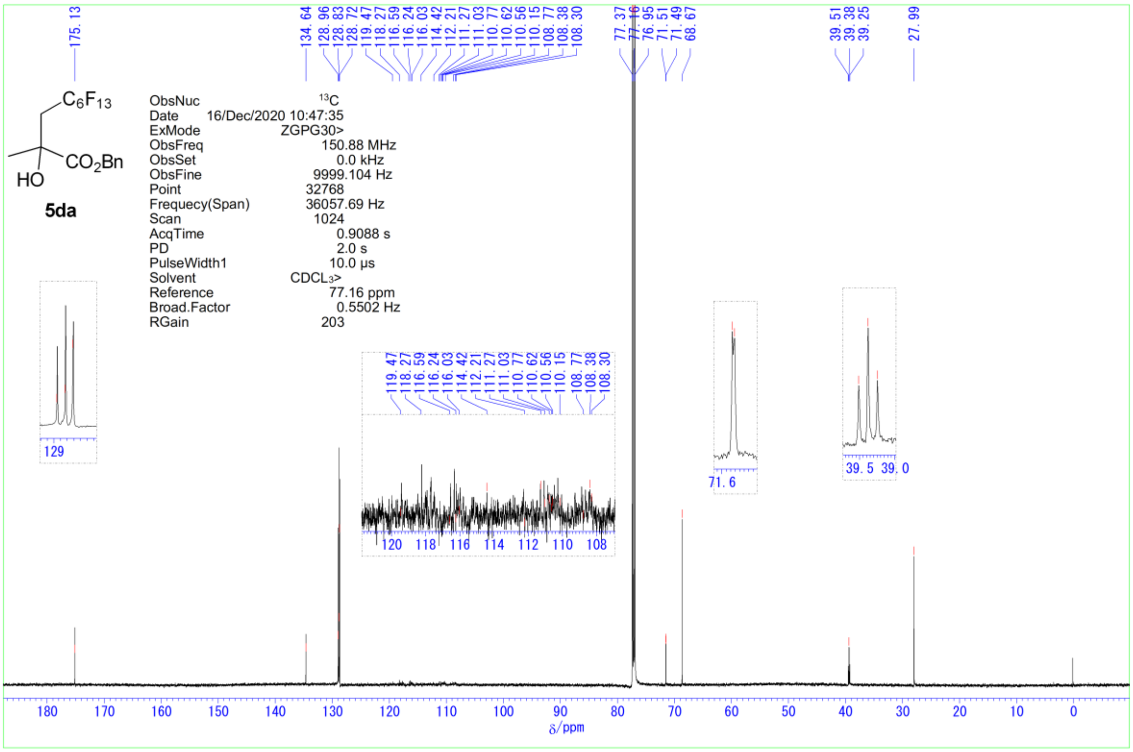

5da: ^{19}F NMR ($CDCl_3$, 471 MHz)

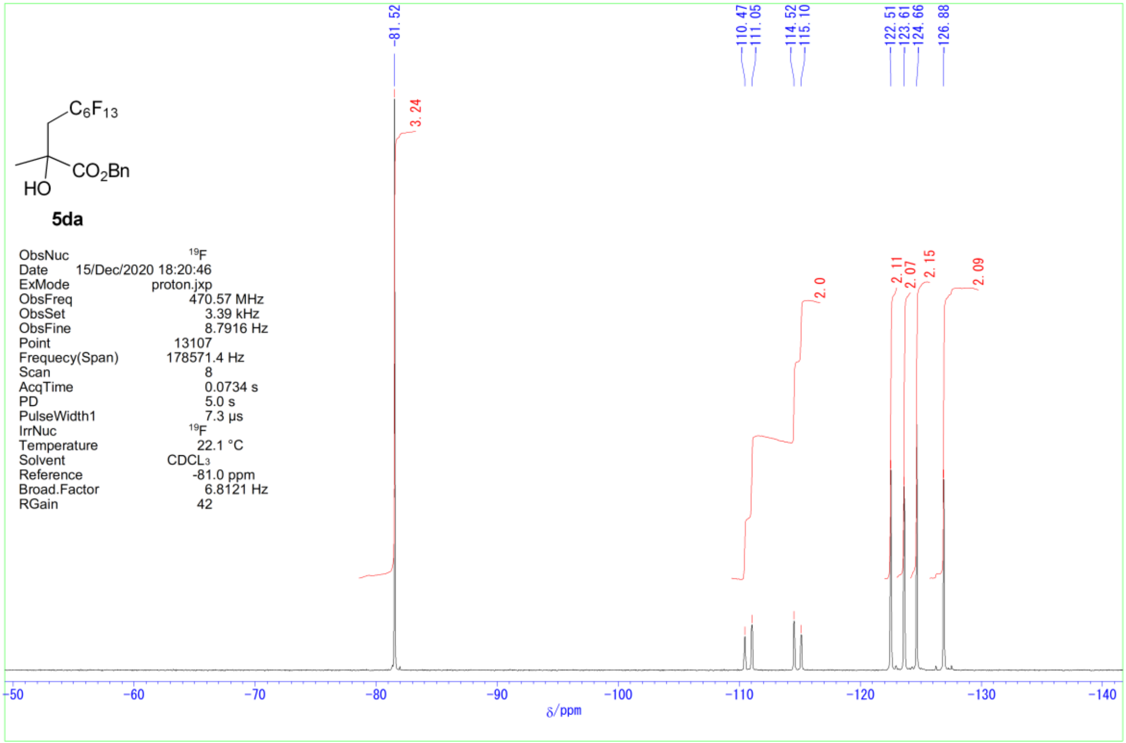

5ea: ^{1}H NMR ($CDCl_3$, 400 MHz)

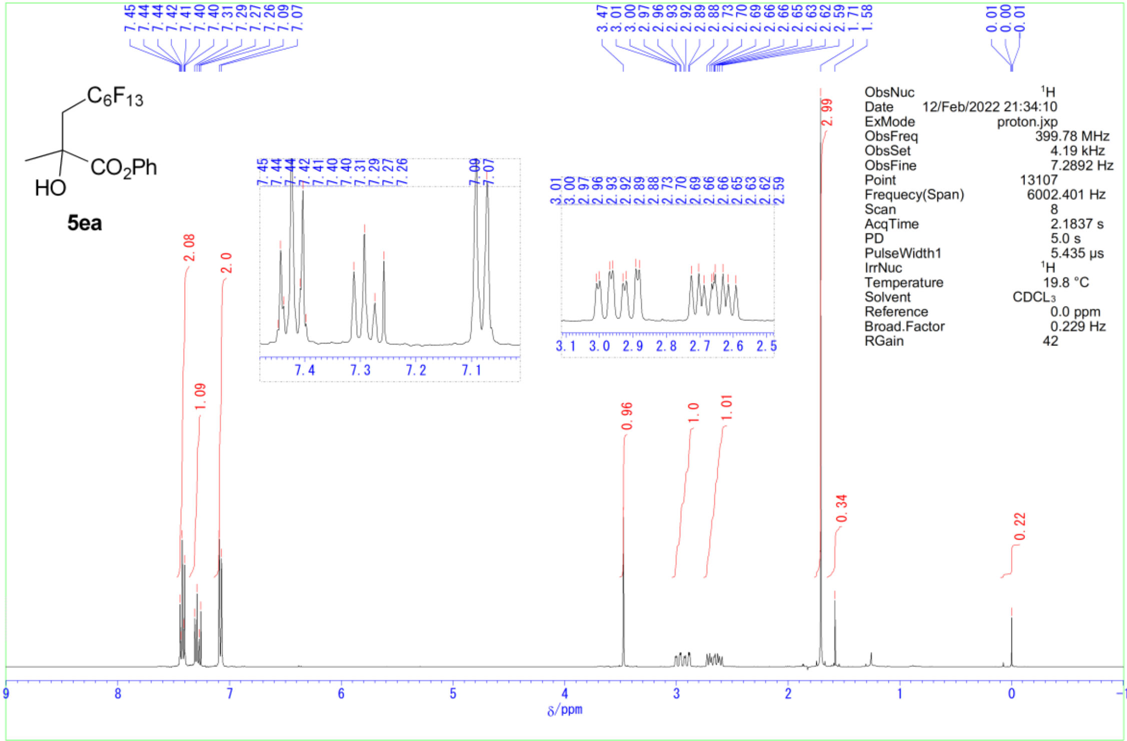

5ea: ^{13}C NMR ($CDCl_3$, 151 MHz)

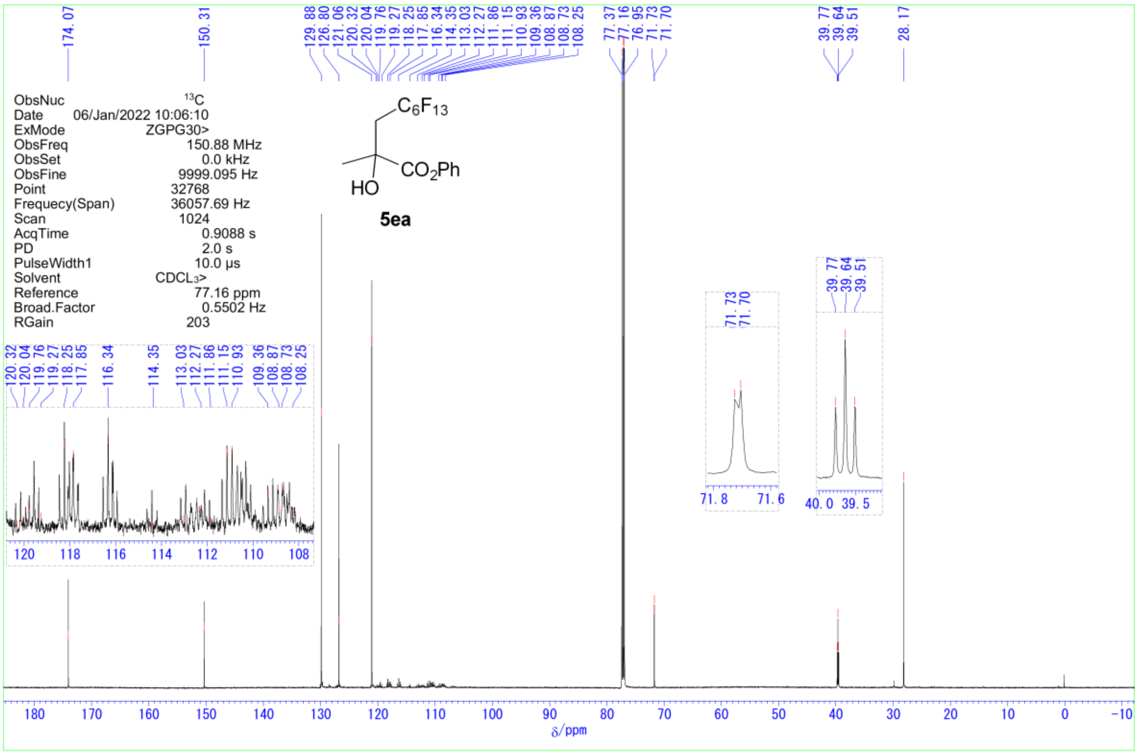

5ea: ^{19}F NMR ($CDCl_3$, 376 MHz)

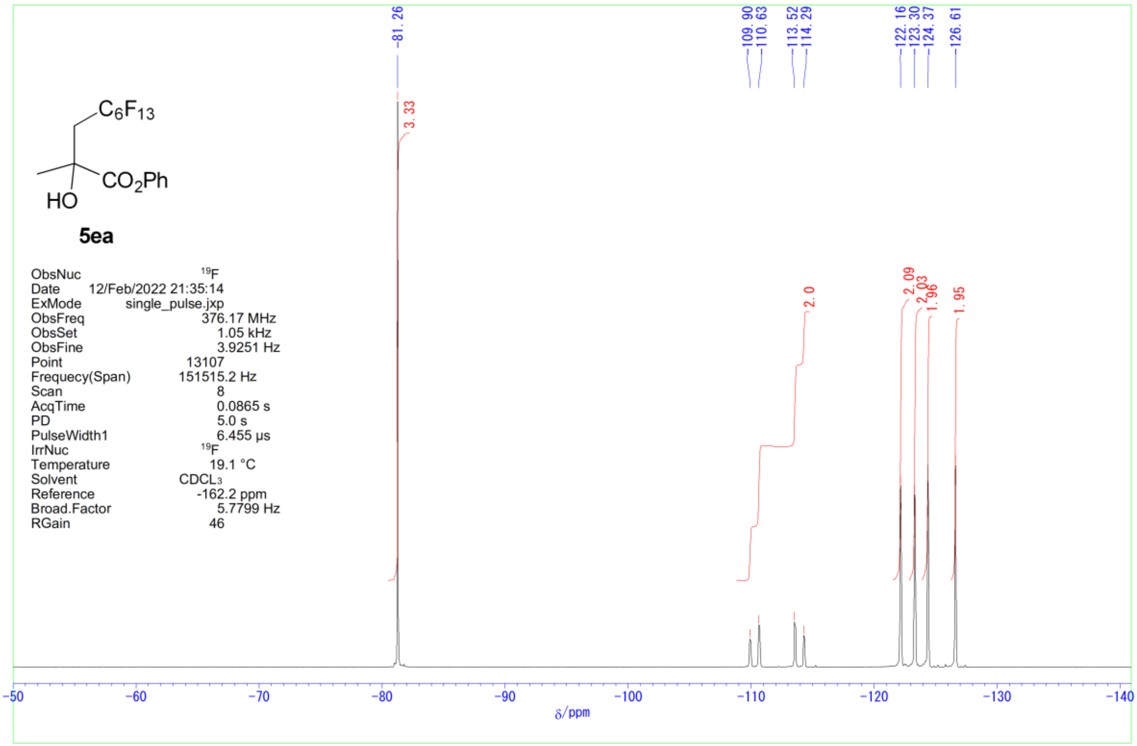

5fa: ^{1}H NMR ($CDCl_3$, 500 MHz)

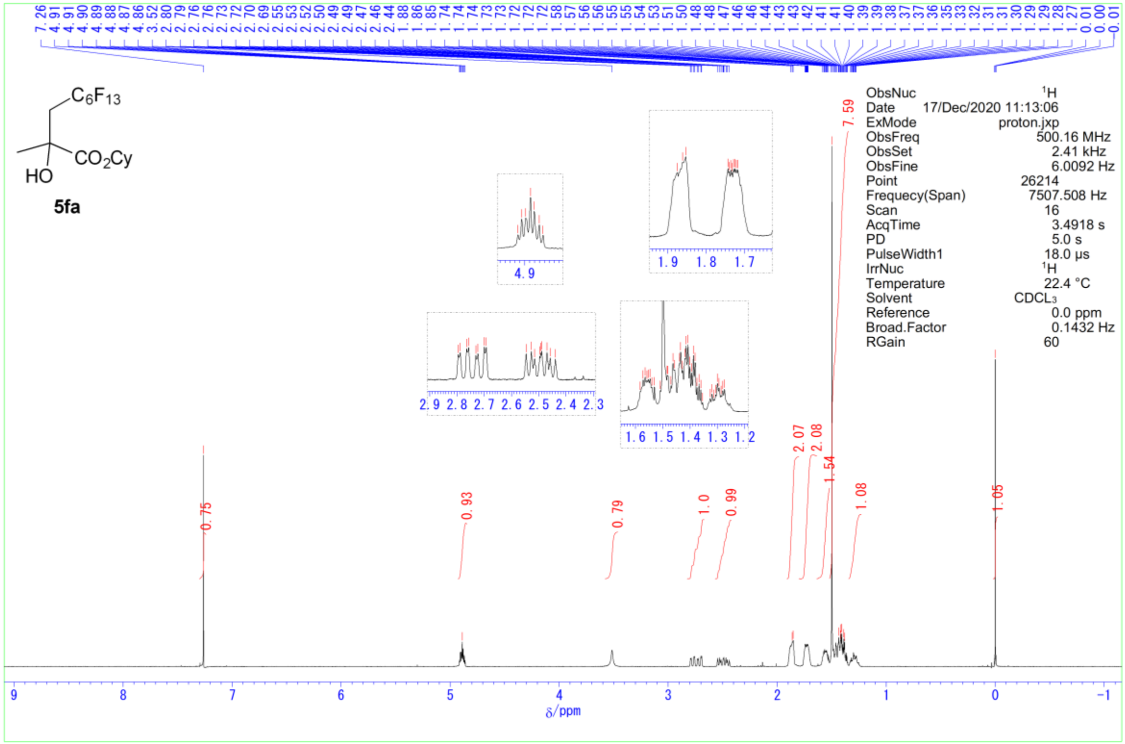

5fa: ^{13}C NMR ($CDCl_3$, 151 MHz)

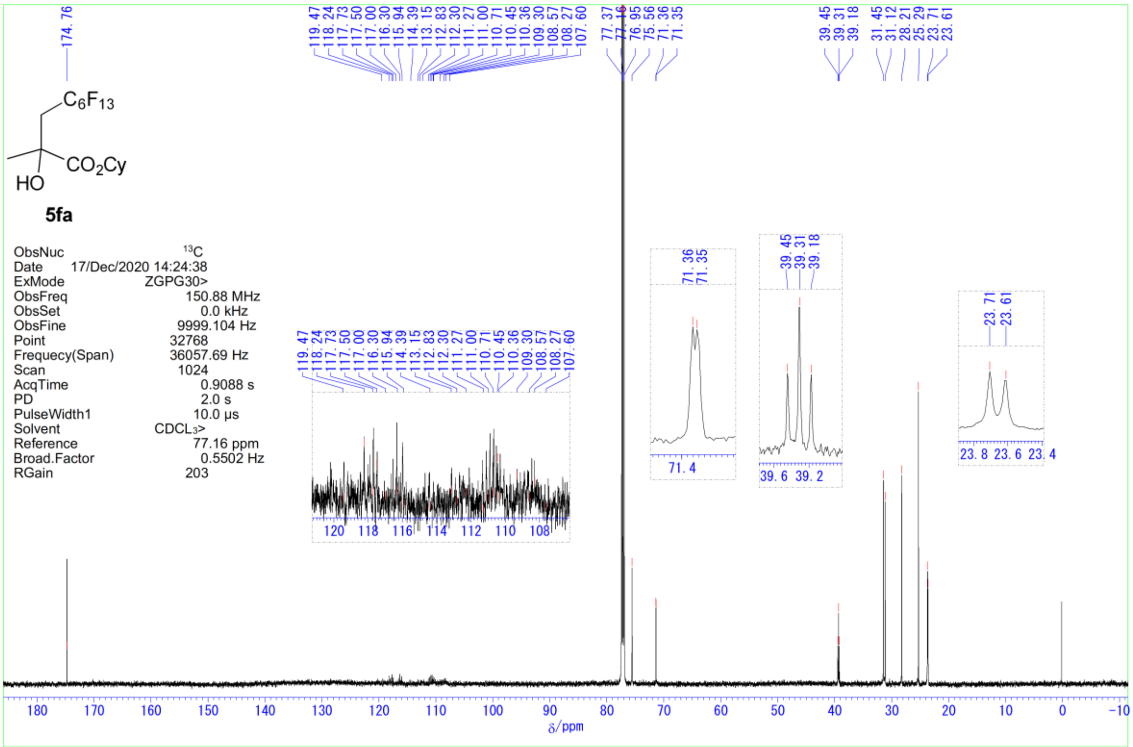

5fa: ^{19}F NMR ($CDCl_3$, 471 MHz)

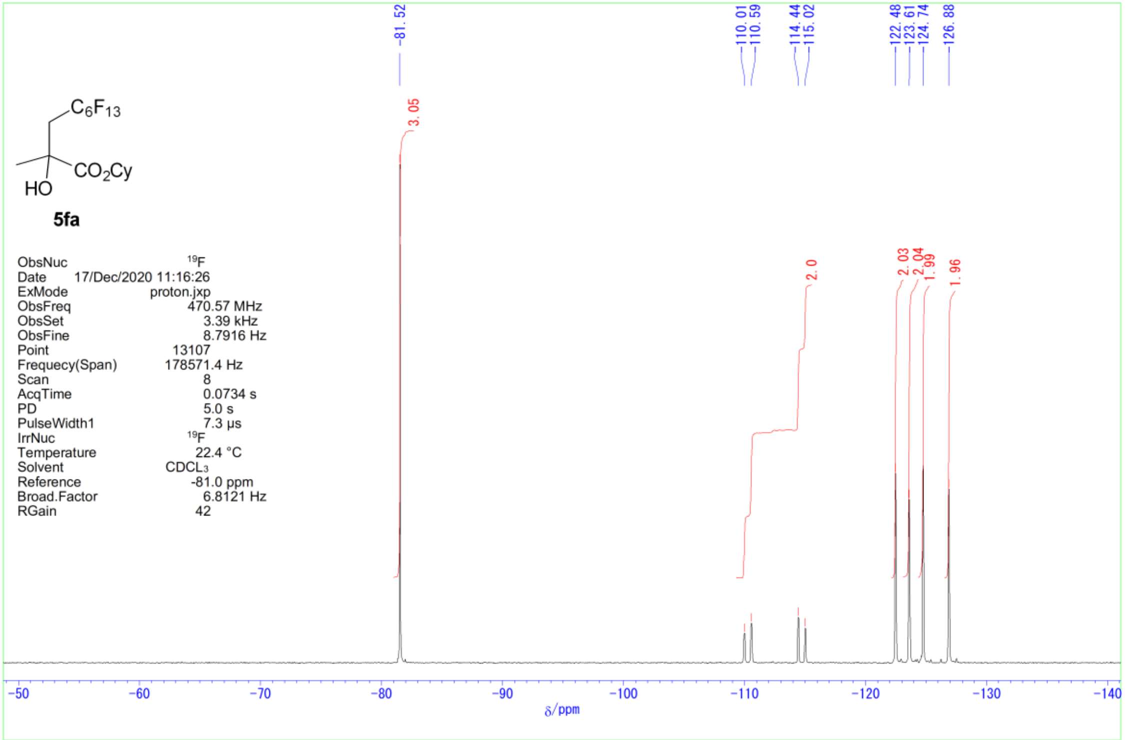

5ga: ^{1}H NMR (CDCl$_3$, 400 MHz)

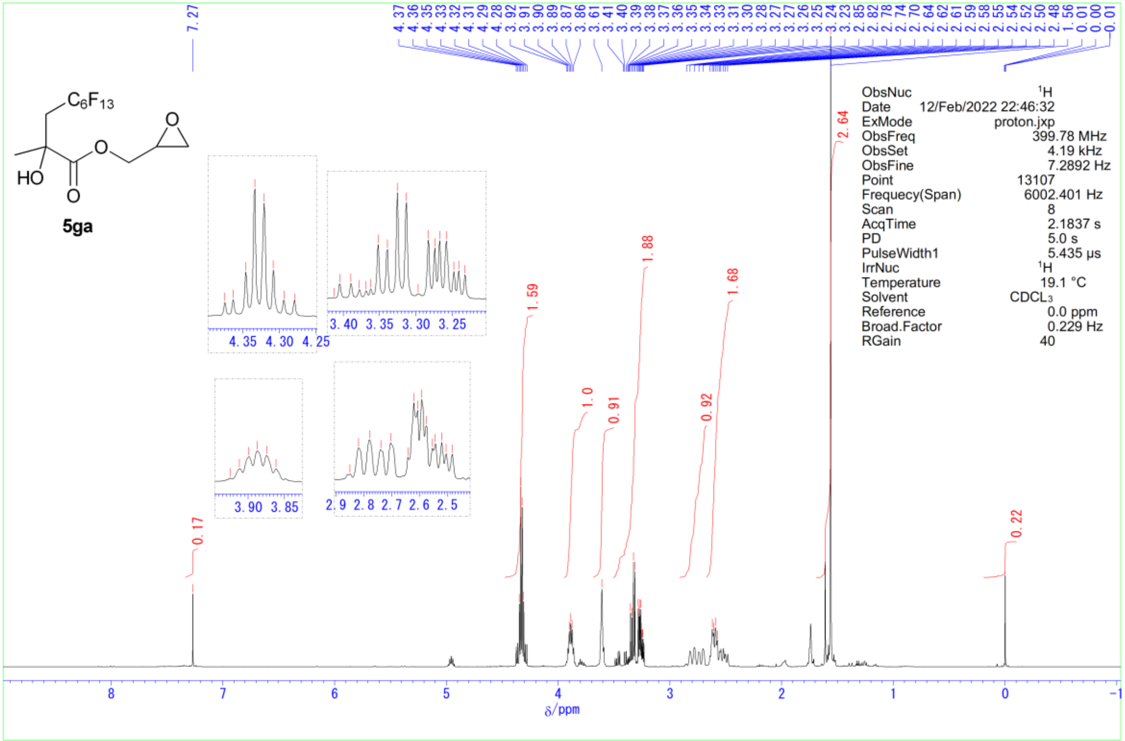

5ga: ^{13}C NMR (CDCl$_3$, 151 MHz)

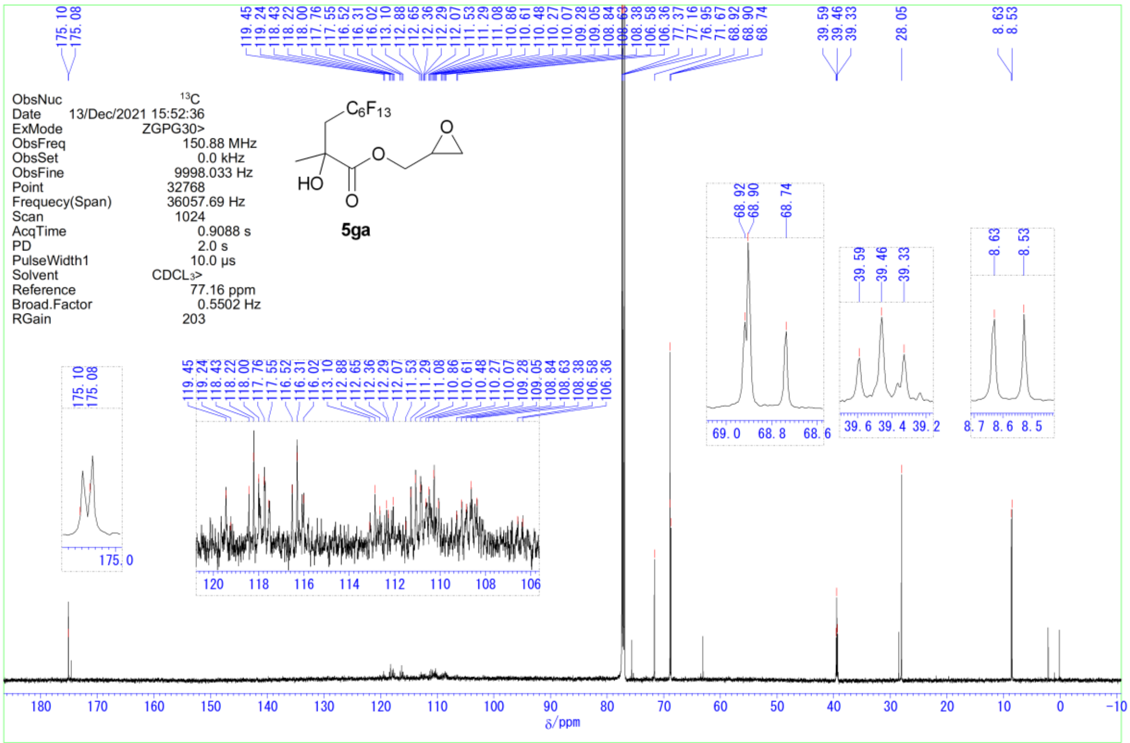

5ga: ^{19}F NMR ($CDCl_3$, 376 MHz)

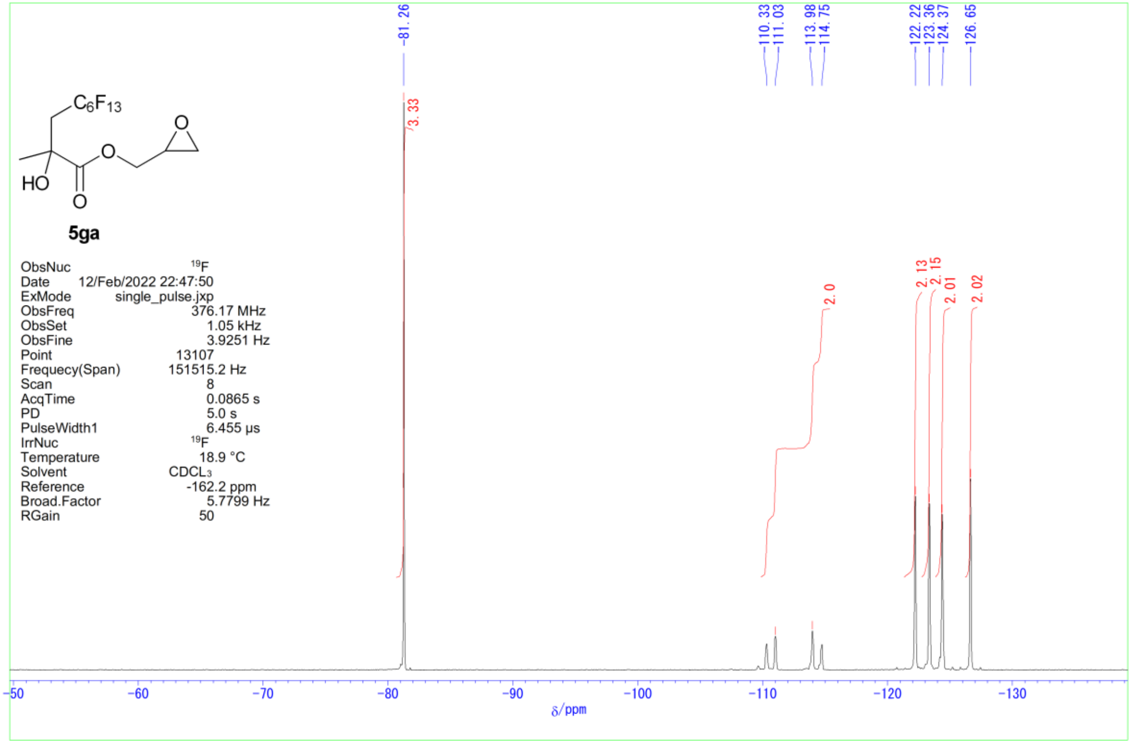

5ha: ^{1}H NMR ($CDCl_3$, 400 MHz)

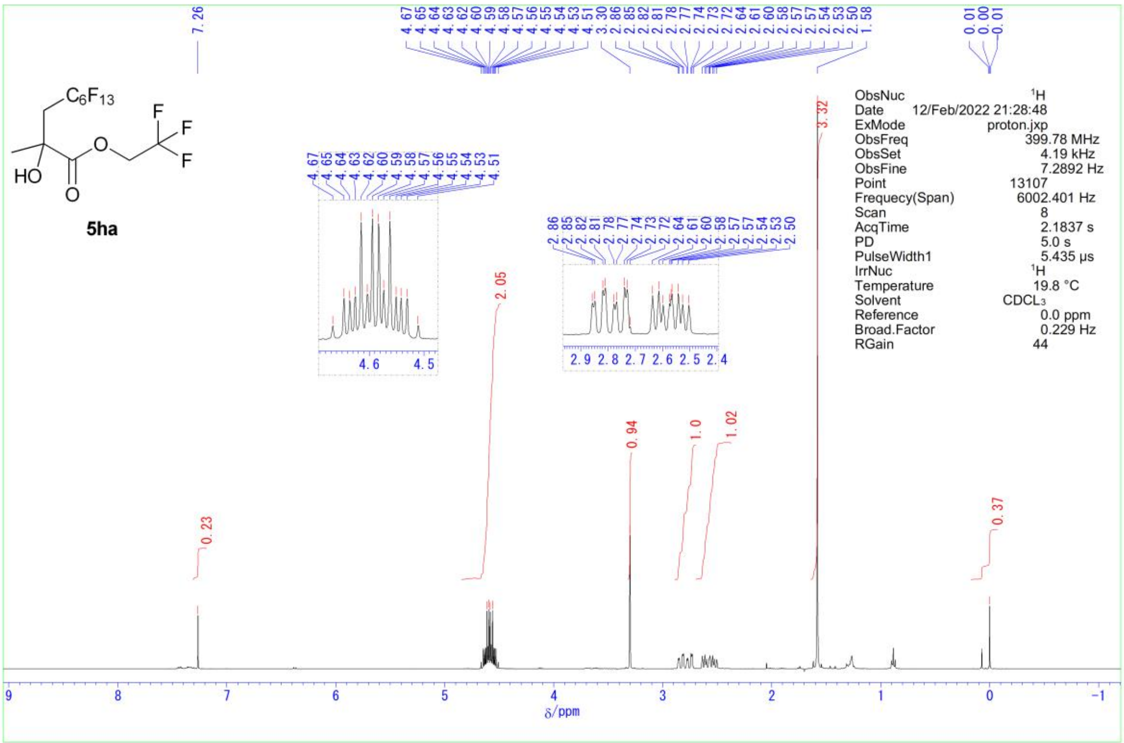

5ha: ^{13}C NMR ($CDCl_3$, 151 MHz)

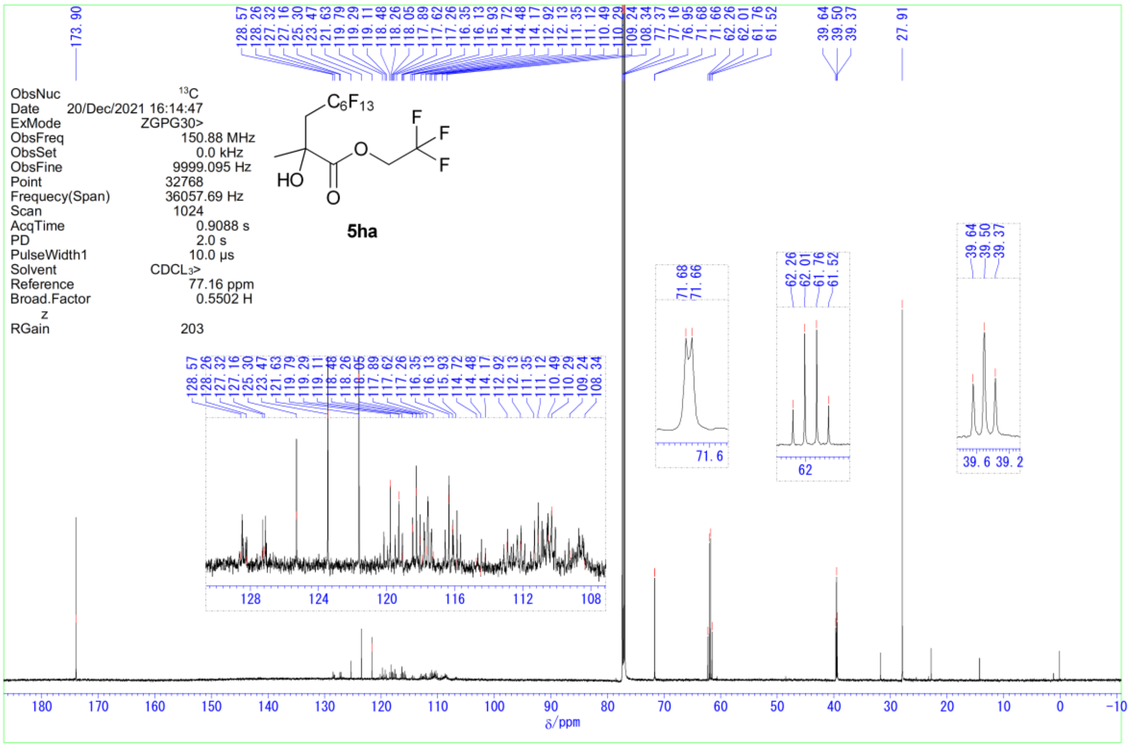

5ha: ^{19}F NMR ($CDCl_3$, 376 MHz)

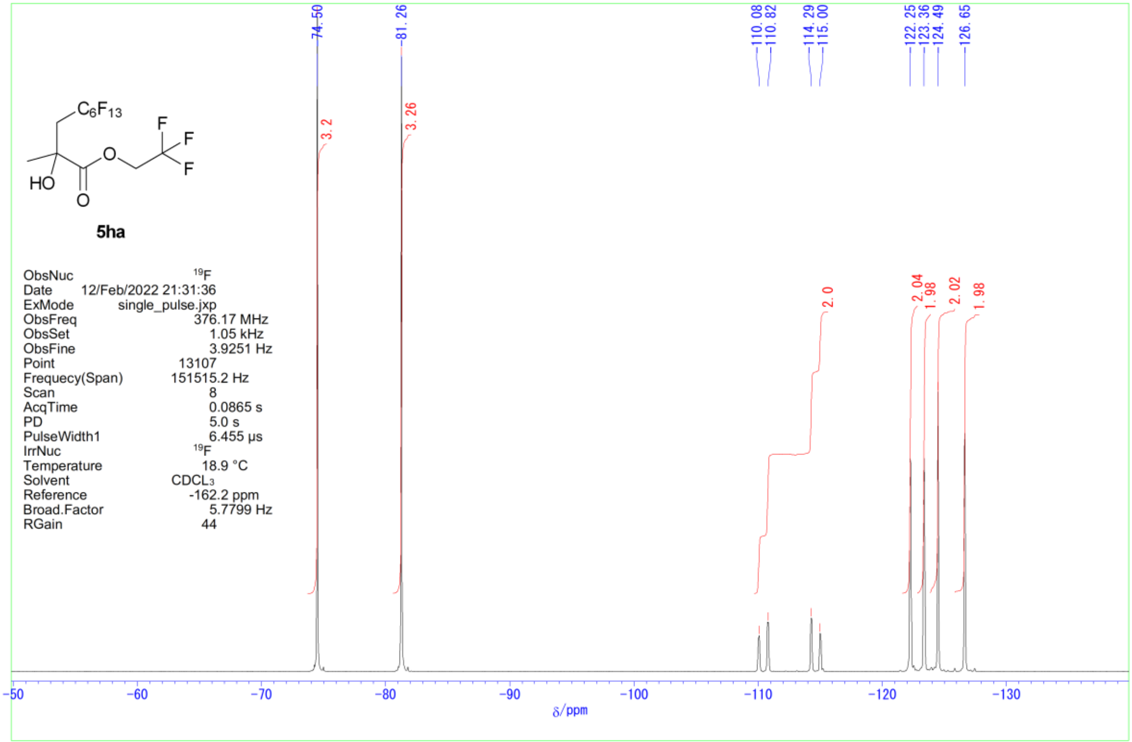

5ia: ^{1}H NMR ($CDCl_3$, 400 MHz)

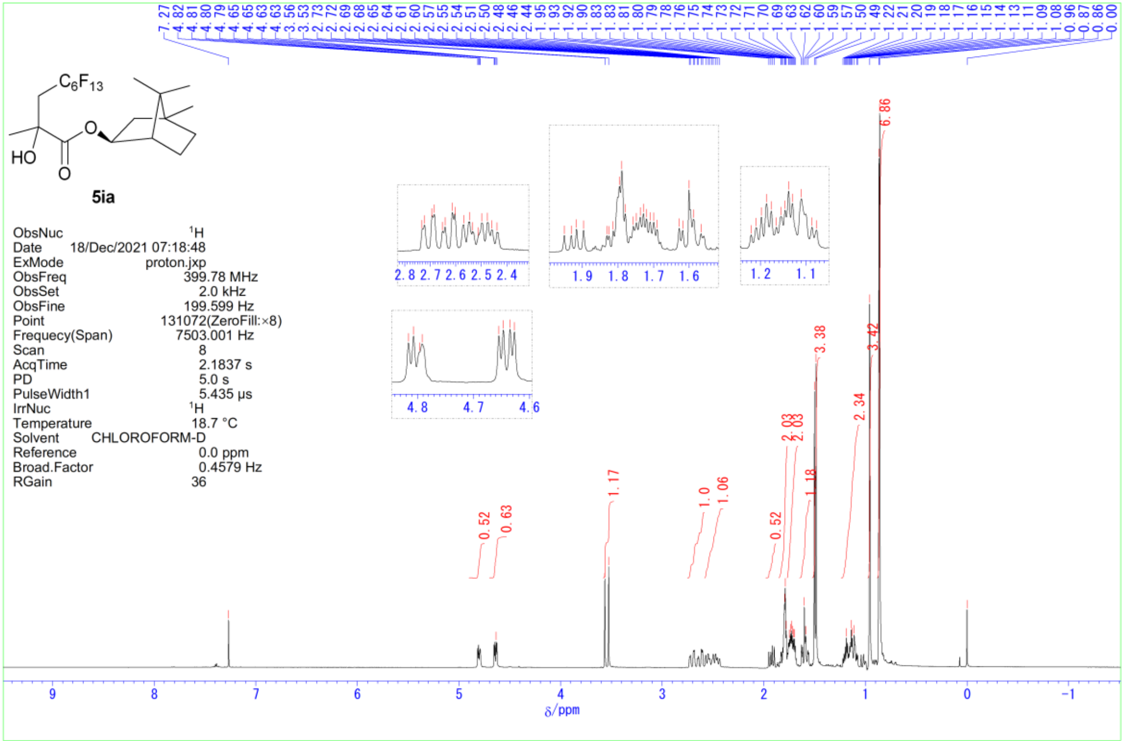

5ia: ^{13}C NMR ($CDCl_3$, 151 MHz)

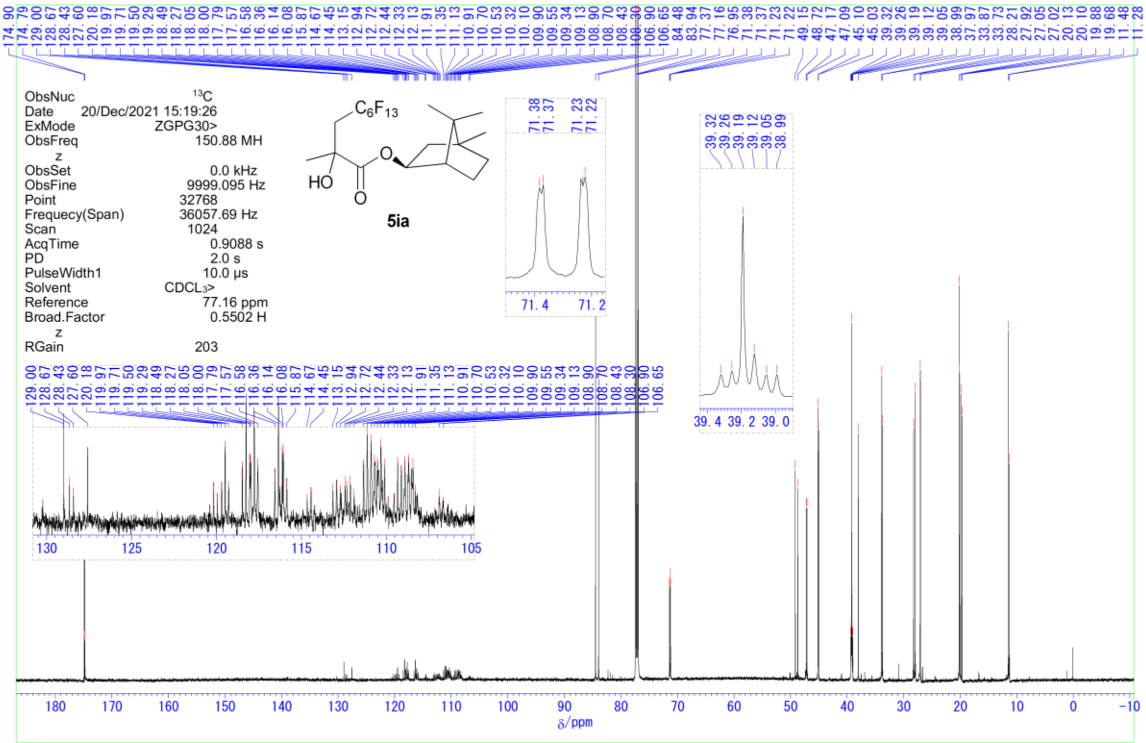

5ia: ^{19}F NMR ($CDCl_3$, 376 MHz)

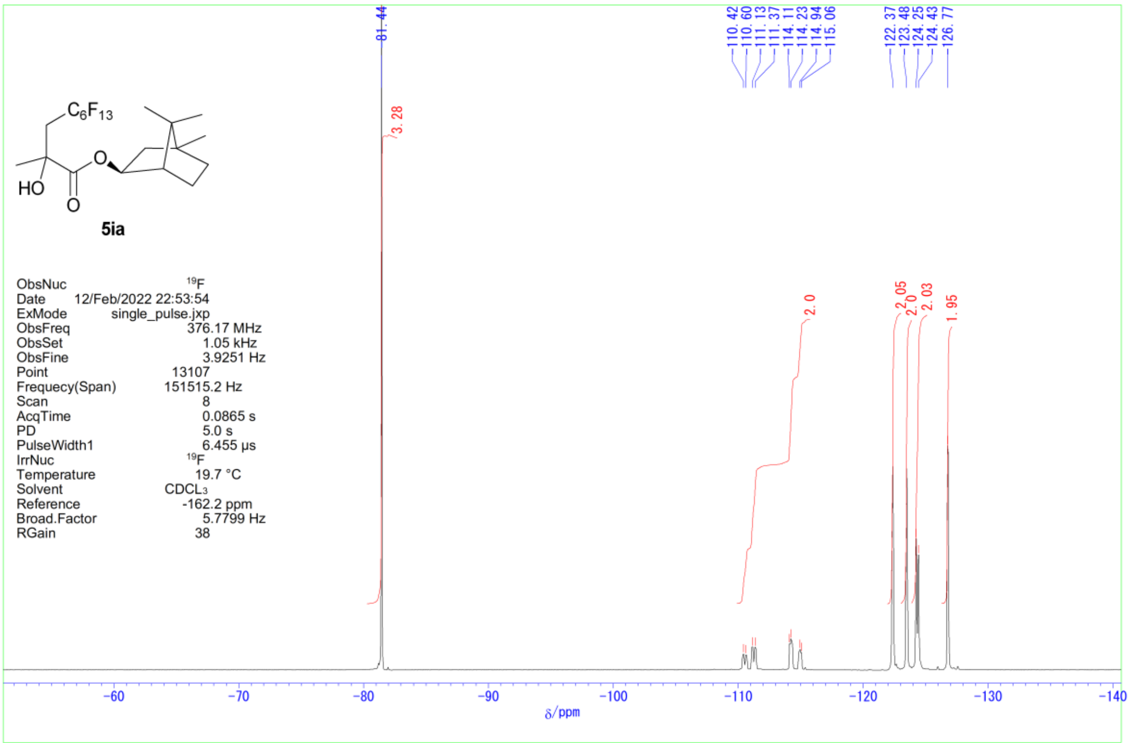

5ja: ^{1}H NMR ($CDCl_3$, 400 MHz)

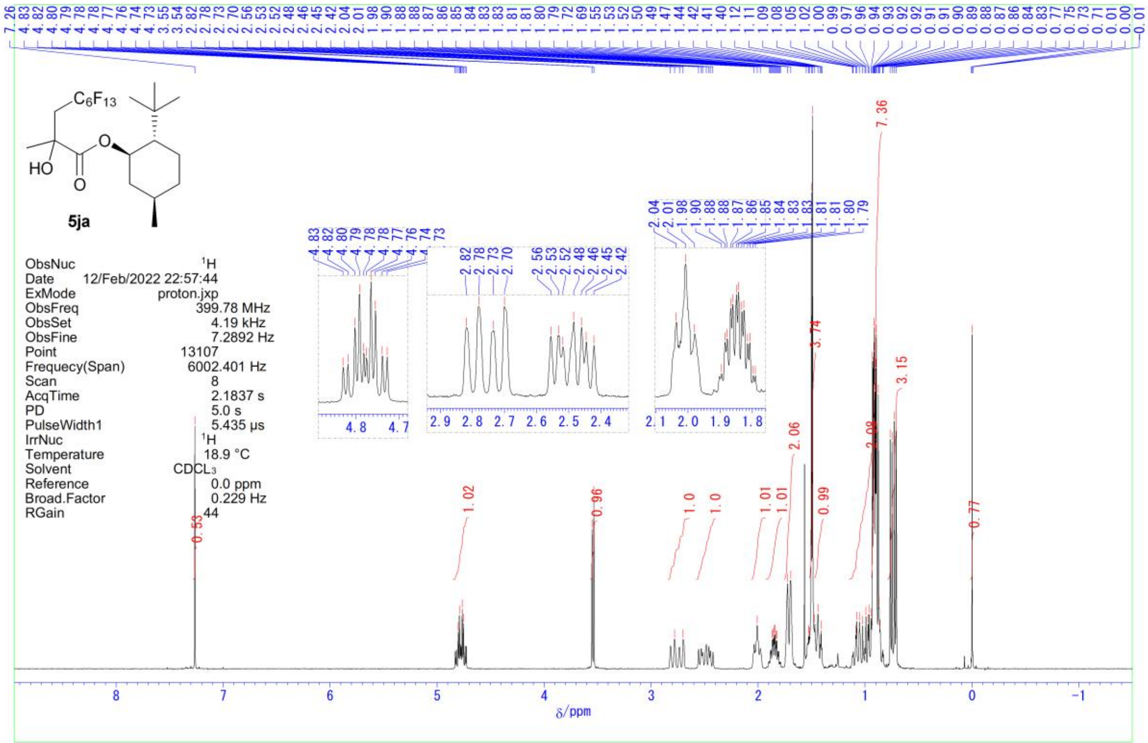

5ja: ^{13}C NMR ($CDCl_3$, 151 MHz)

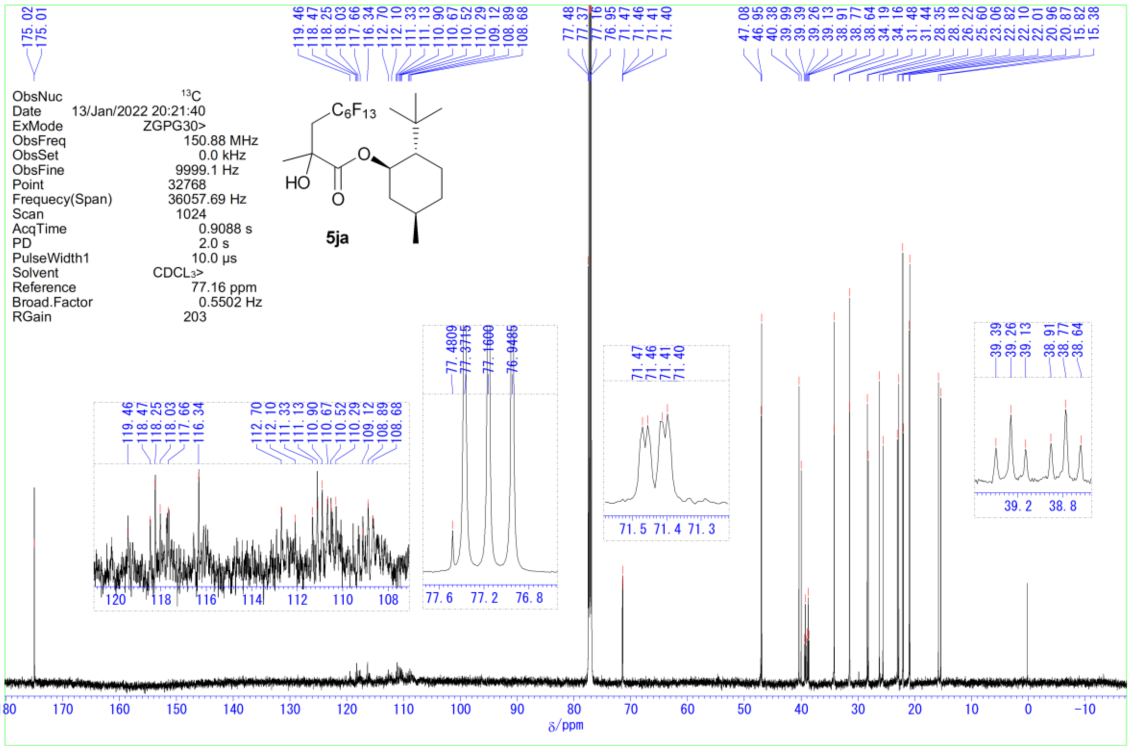

5ja: ^{19}F NMR ($CDCl_3$, 376 MHz)

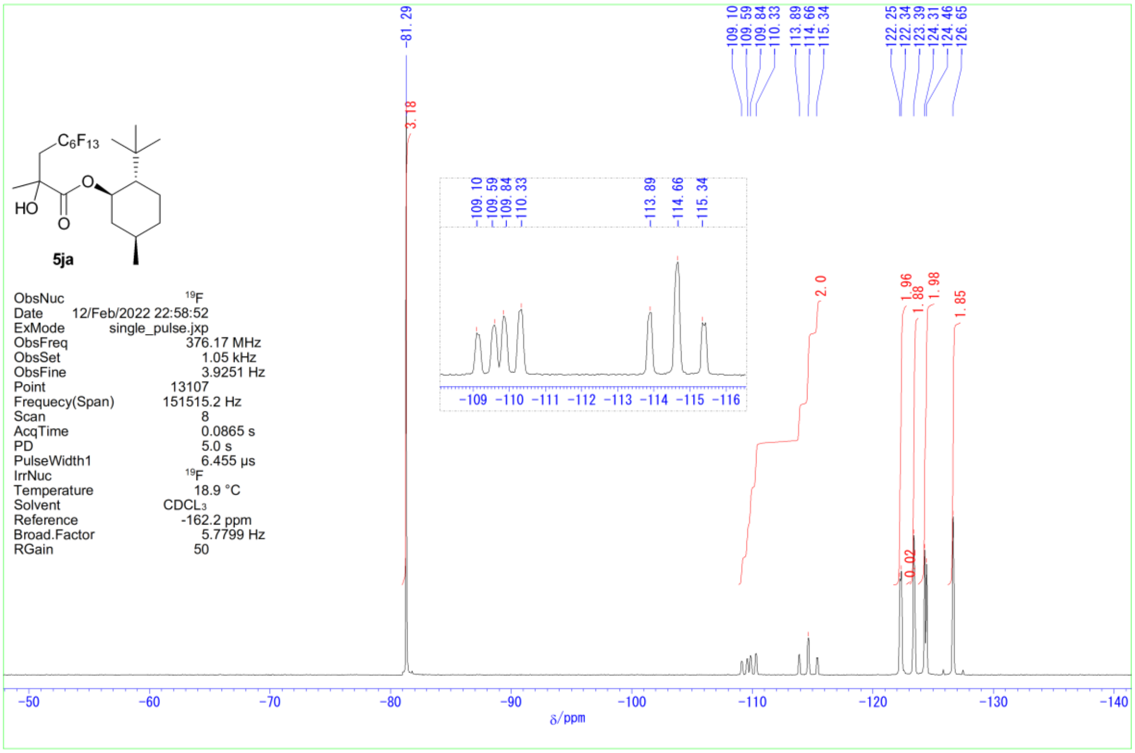

5ka: ^{1}H NMR ($CDCl_3$, 400 MHz)

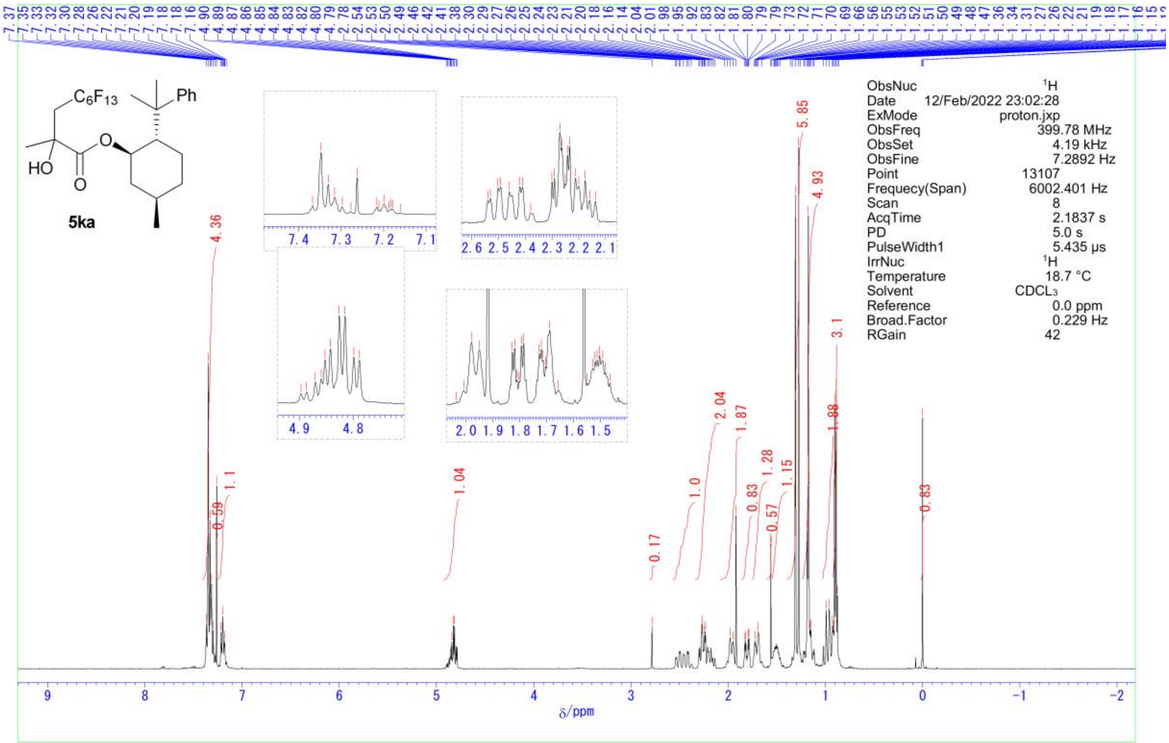

5ka: ^{13}C NMR ($CDCl_3$, 151 MHz)

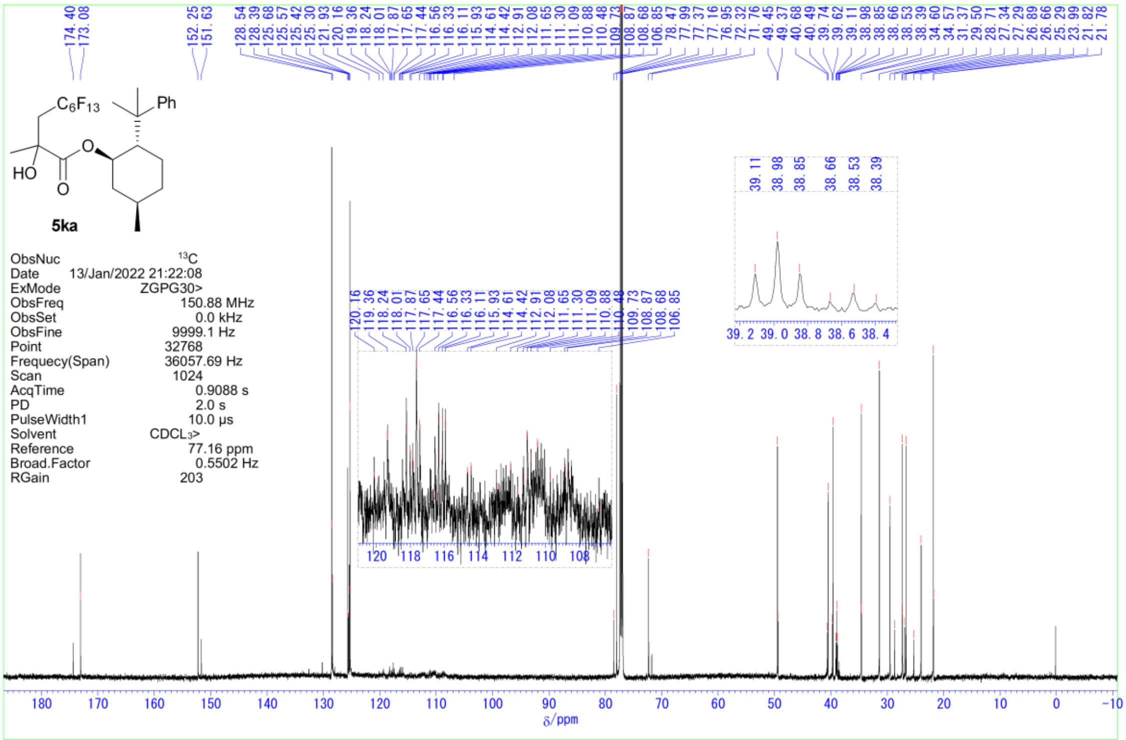

5ka: ^{19}F NMR ($CDCl_3$, 376 MHz)

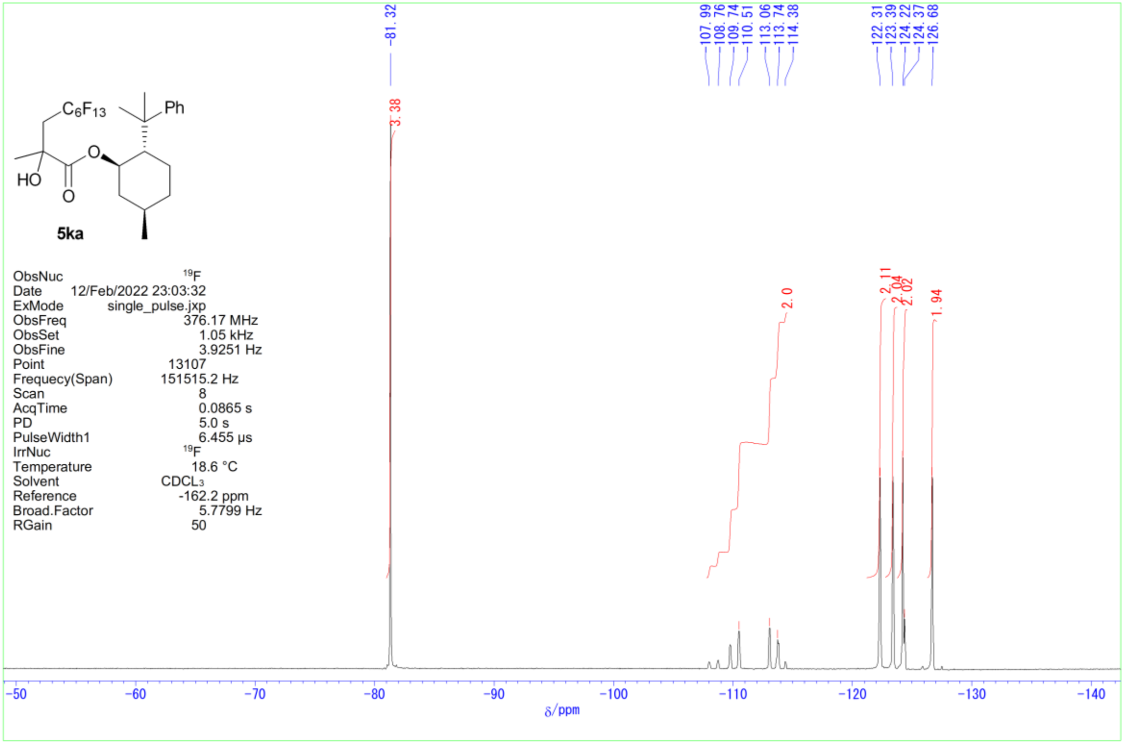

5la: ^{1}H NMR ($CDCl_3$, 500 MHz)

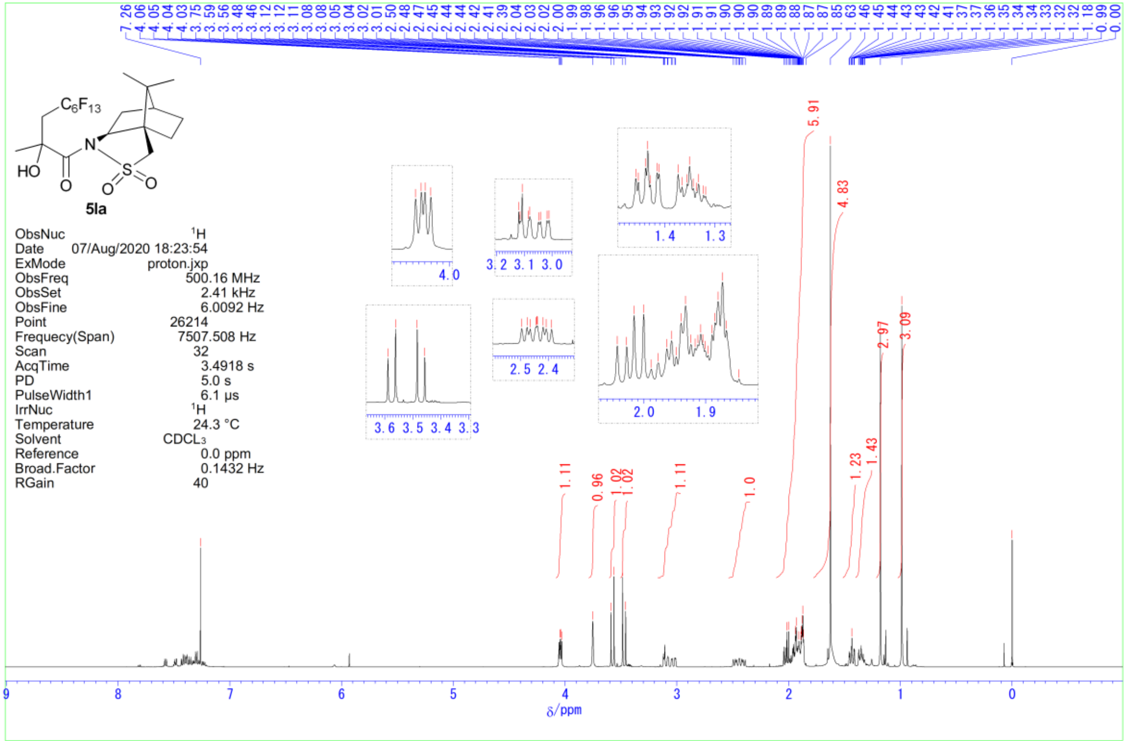

5la: ^{13}C NMR ($CDCl_3$, 151 MHz)

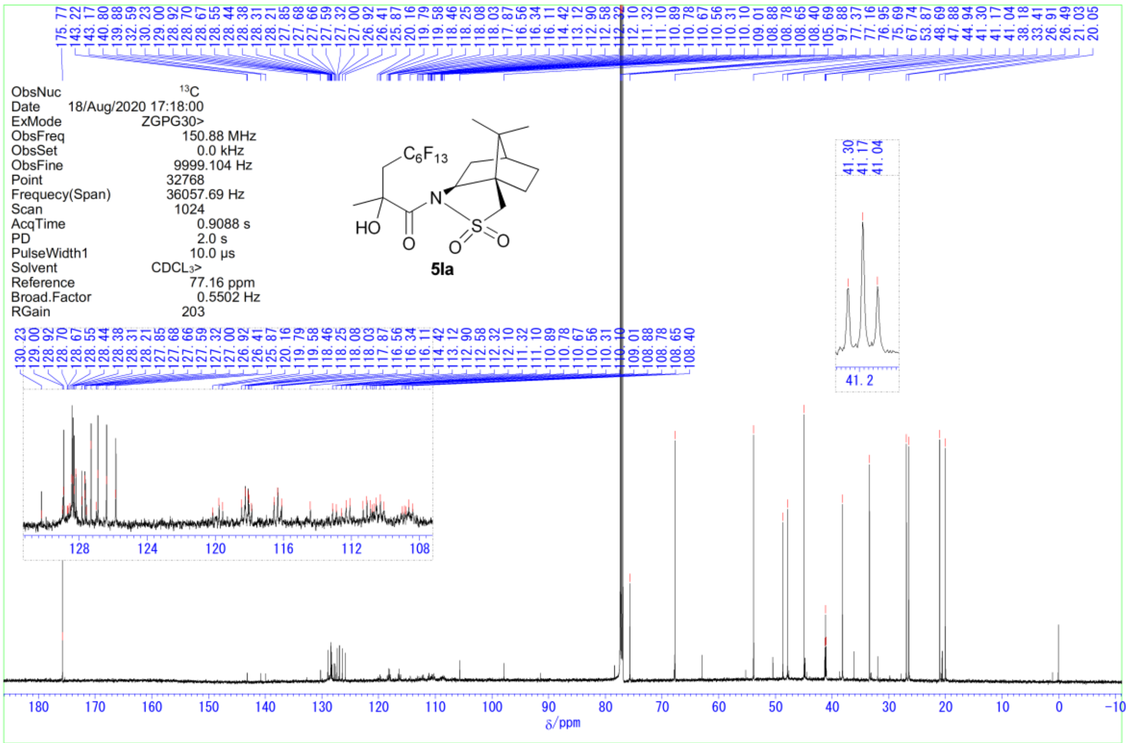

5la: ^{19}F NMR ($CDCl_3$, 376 MHz)

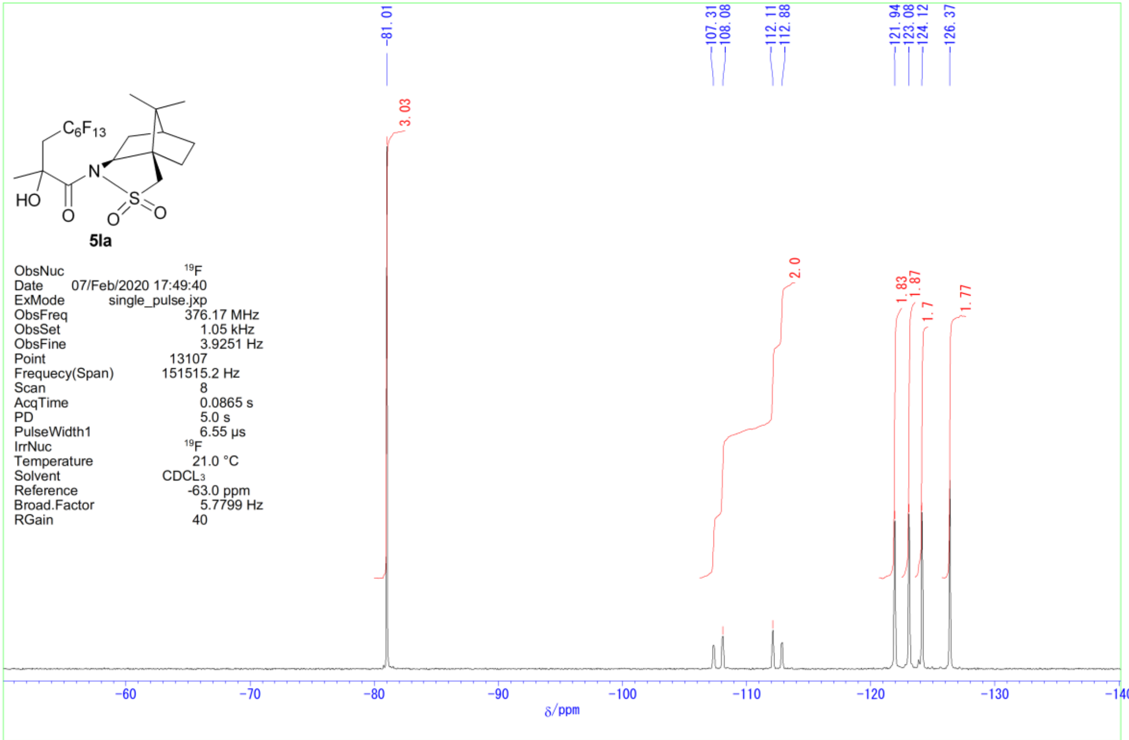

5ma: ^{1}H NMR ($CDCl_3$, 500 MHz)

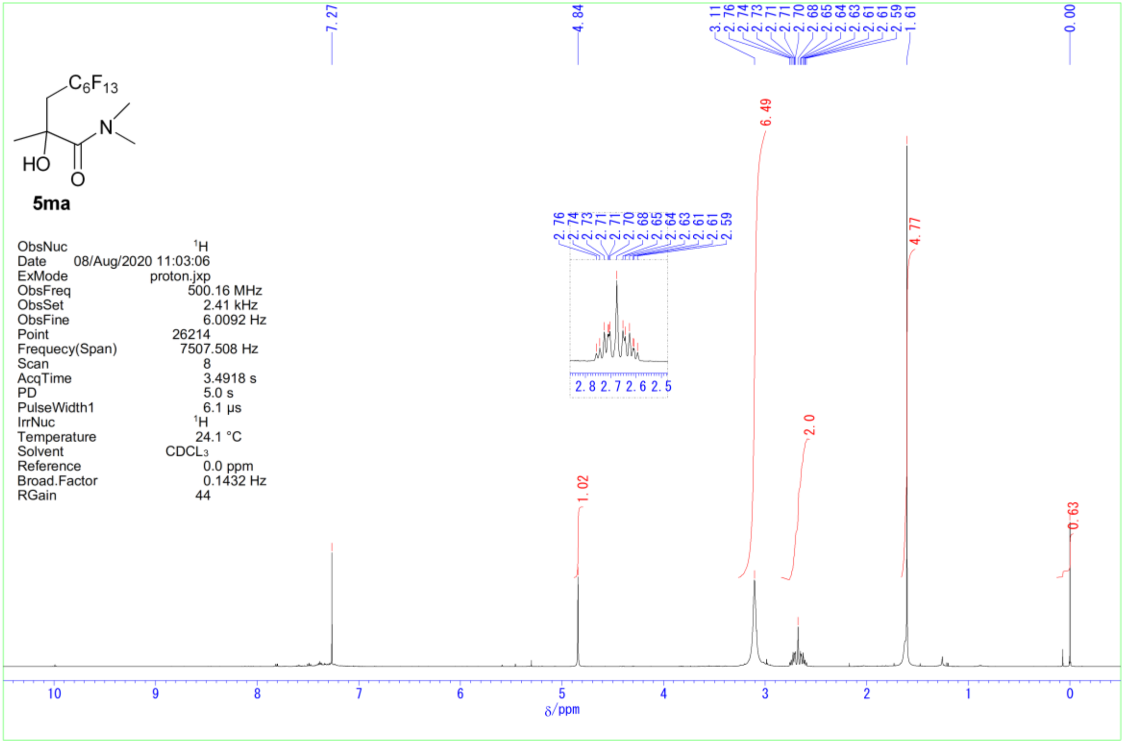

5ma: ^{13}C NMR ($CDCl_3$, 151 MHz)

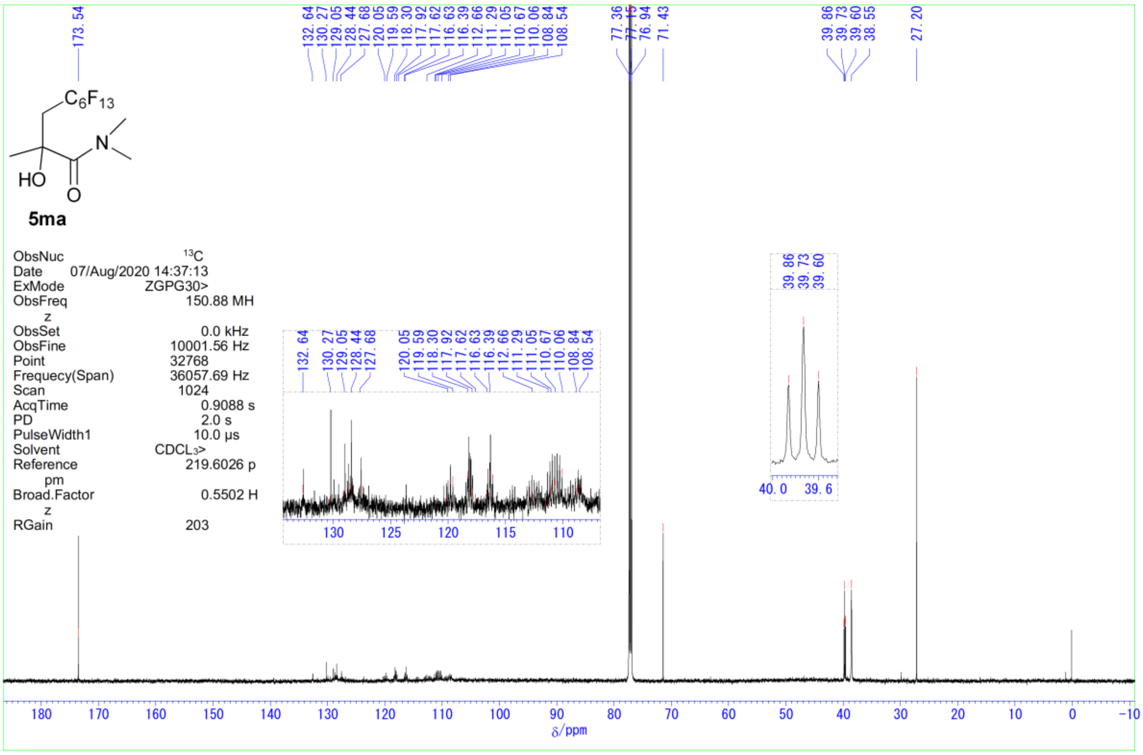

5ma: ^{19}F NMR ($CDCl_3$, 471 MHz)

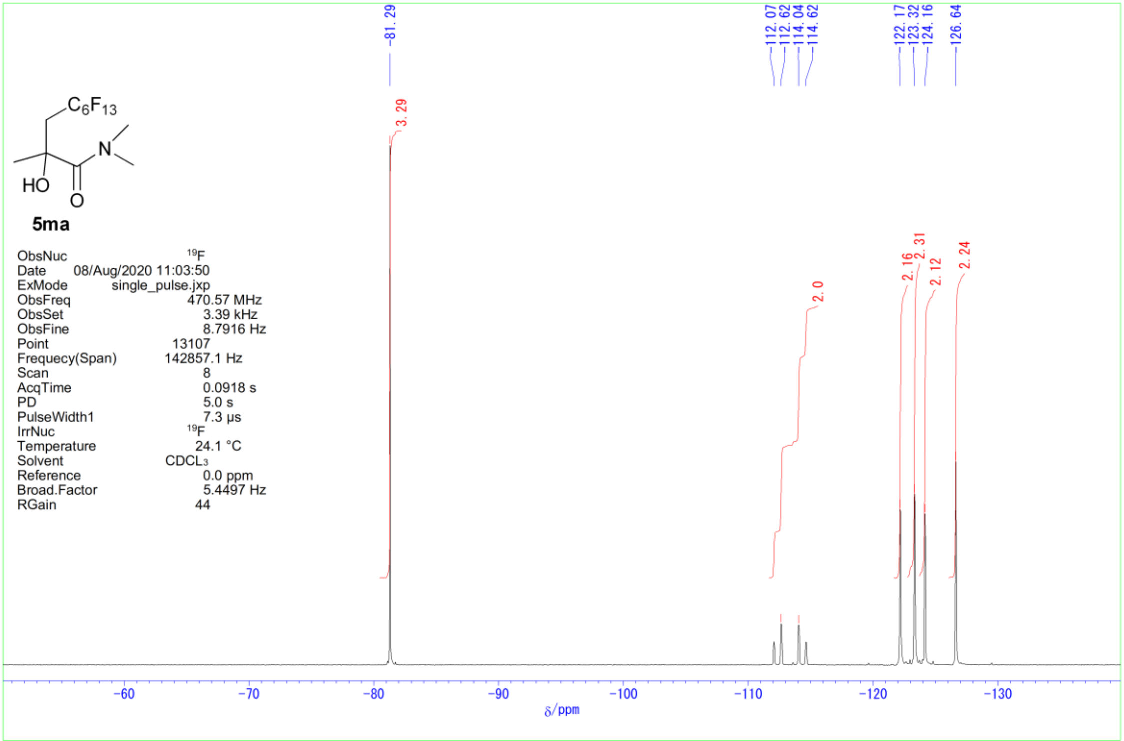

5na: ^{1}H NMR ($CDCl_3$, 400 MHz)

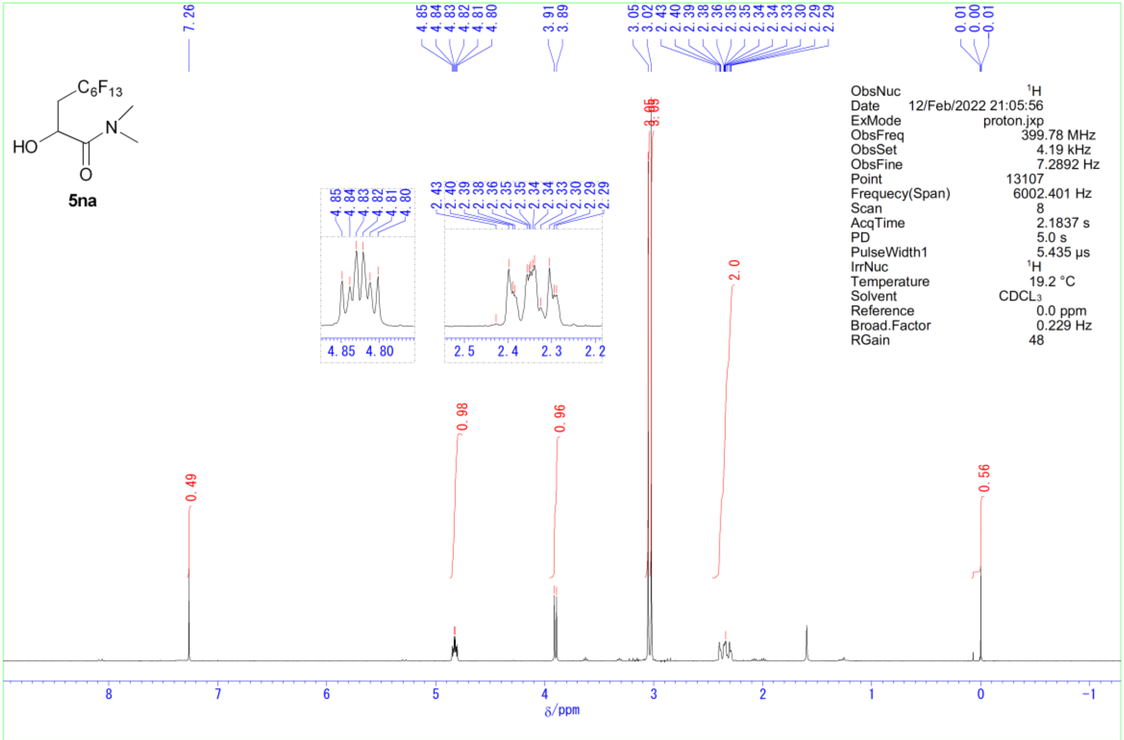

5na: ^{13}C NMR (CDCl$_3$, 151 MHz)

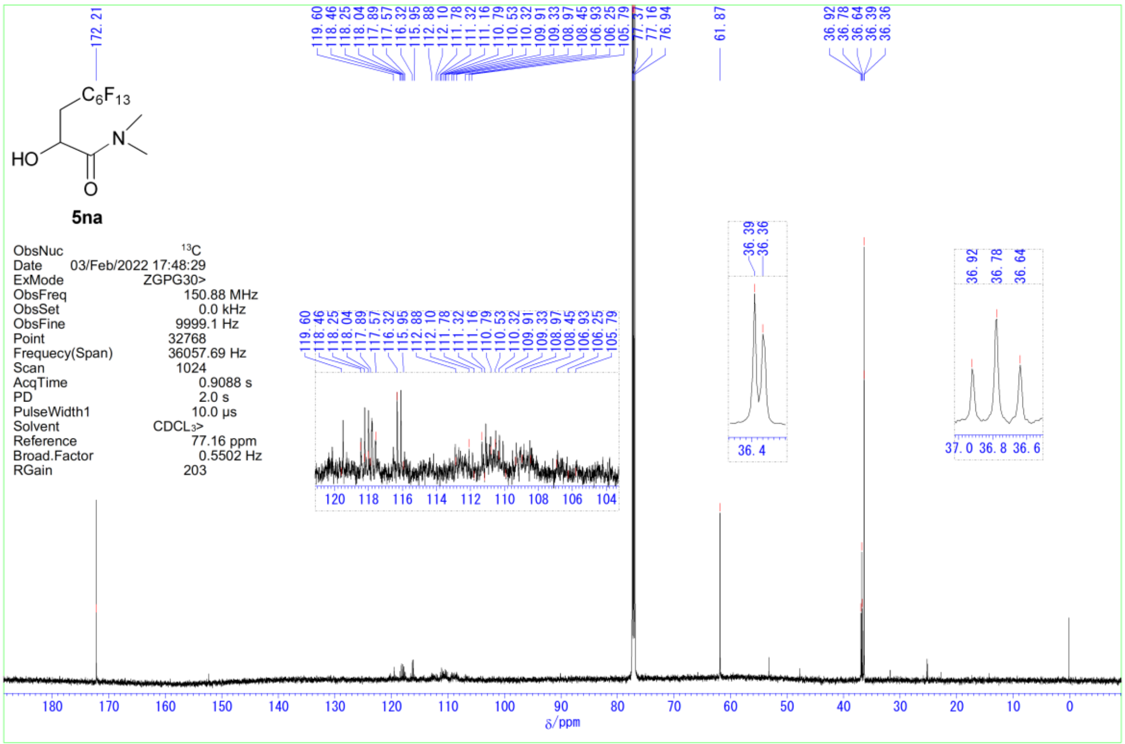

5na: ^{19}F NMR (CDCl$_3$, 376 MHz)

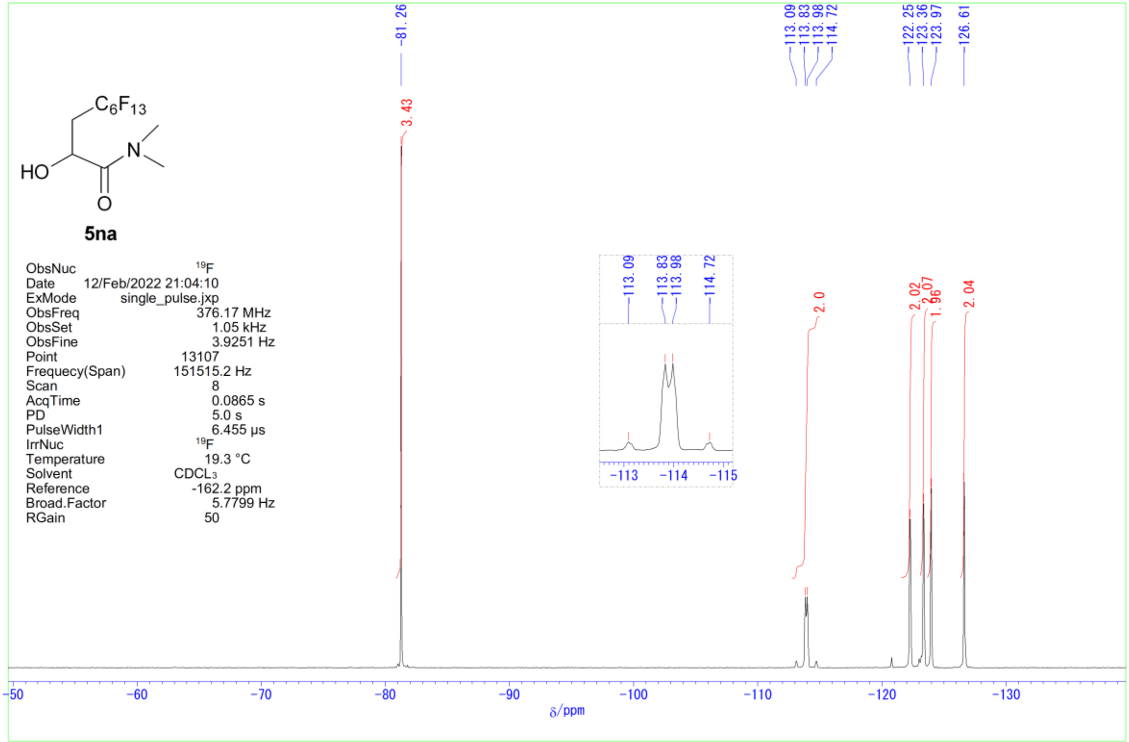

5oa: ^{1}H NMR ($CDCl_3$, 400 MHz)

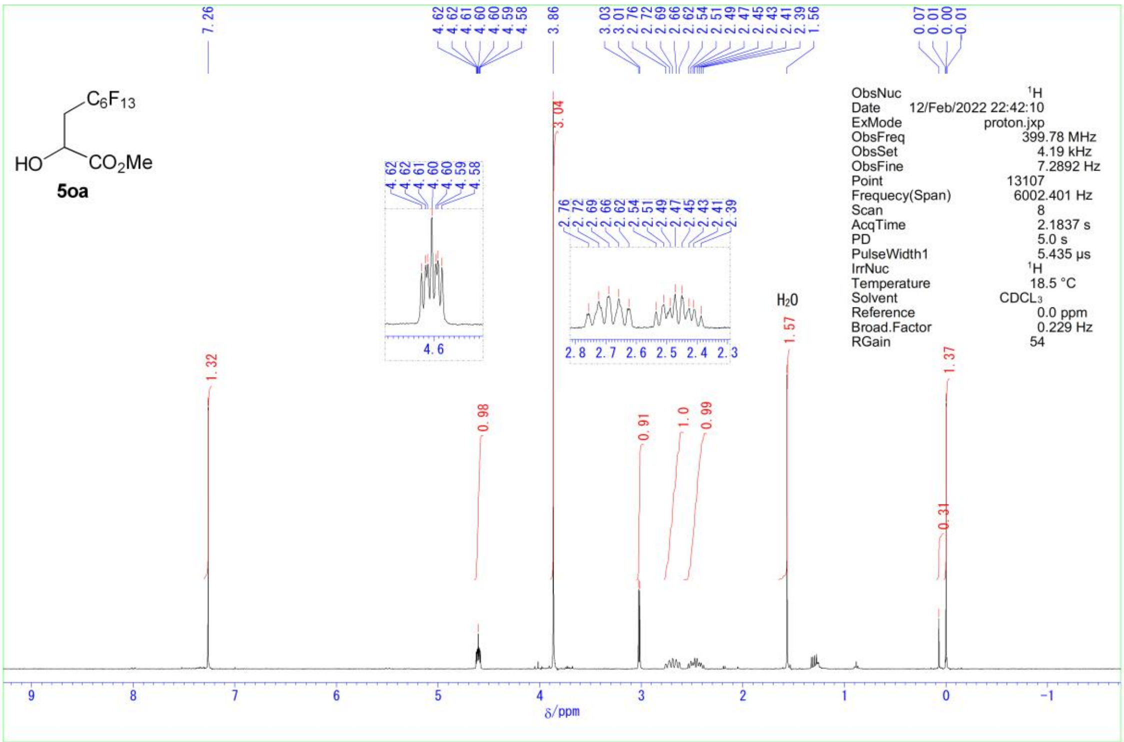

5oa: ^{13}C NMR ($CDCl_3$, 151 MHz)

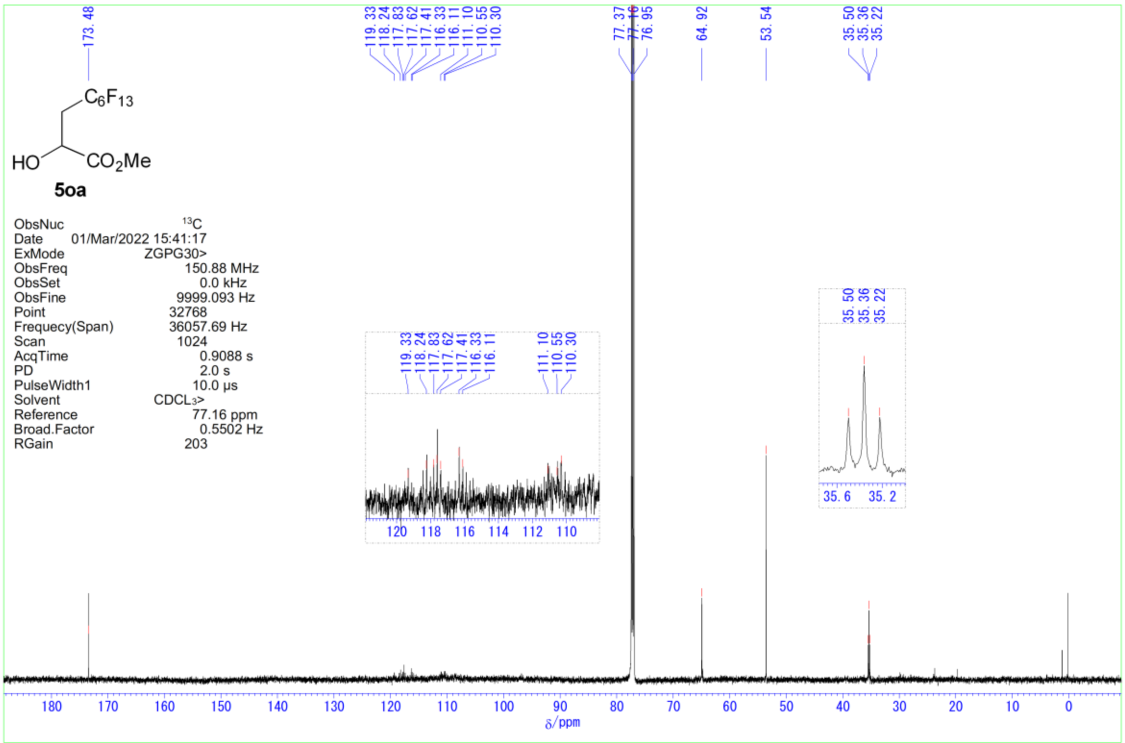

5oa: ^{19}F NMR (CDCl$_3$, 376 MHz)

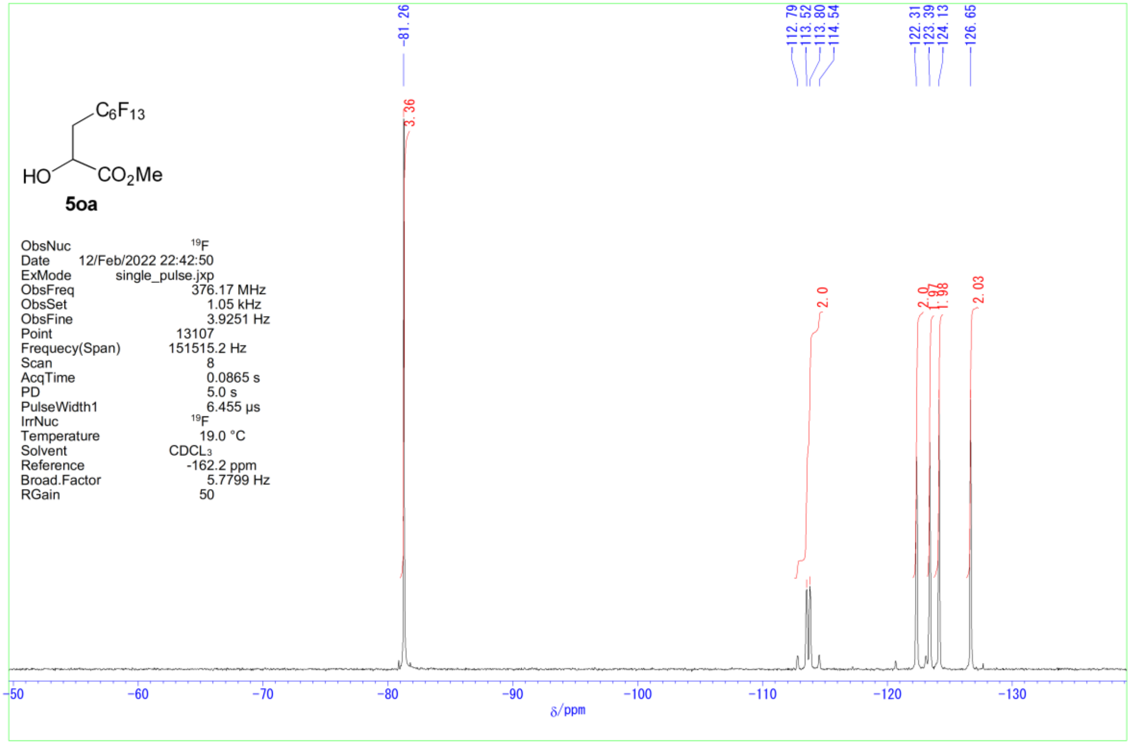

5pa: ^{1}H NMR (CDCl$_3$, 400 MHz)

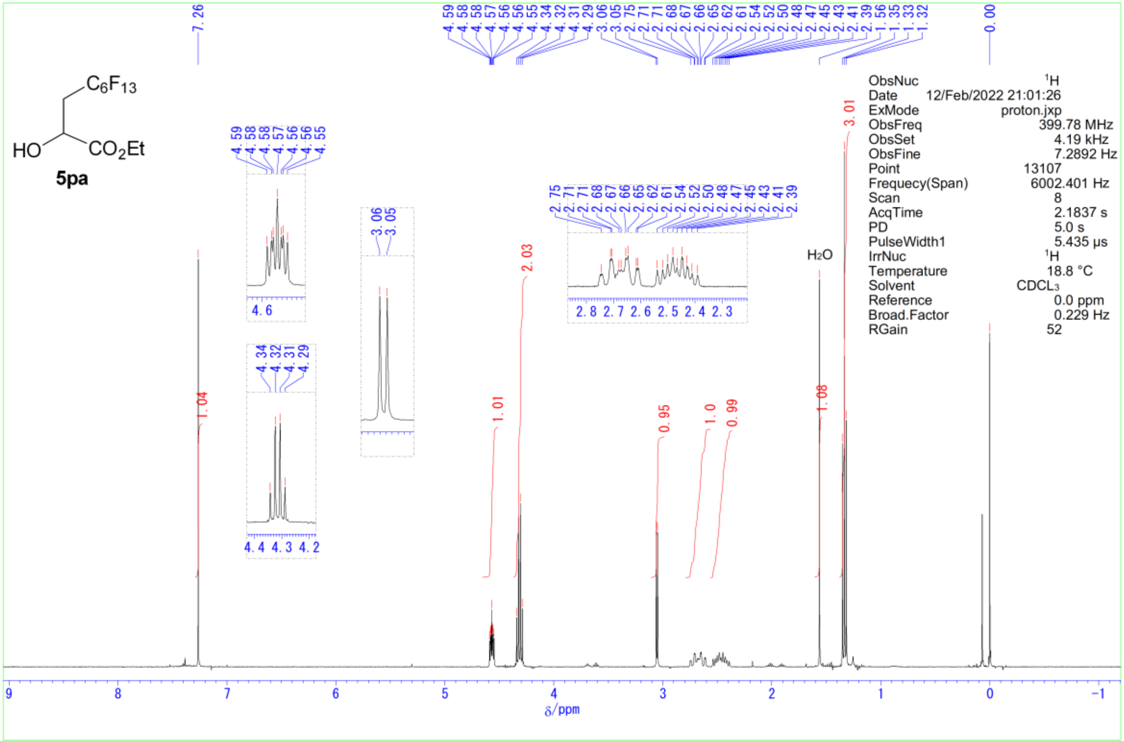

5pa: ^{13}C NMR ($CDCl_3$, 151 MHz)

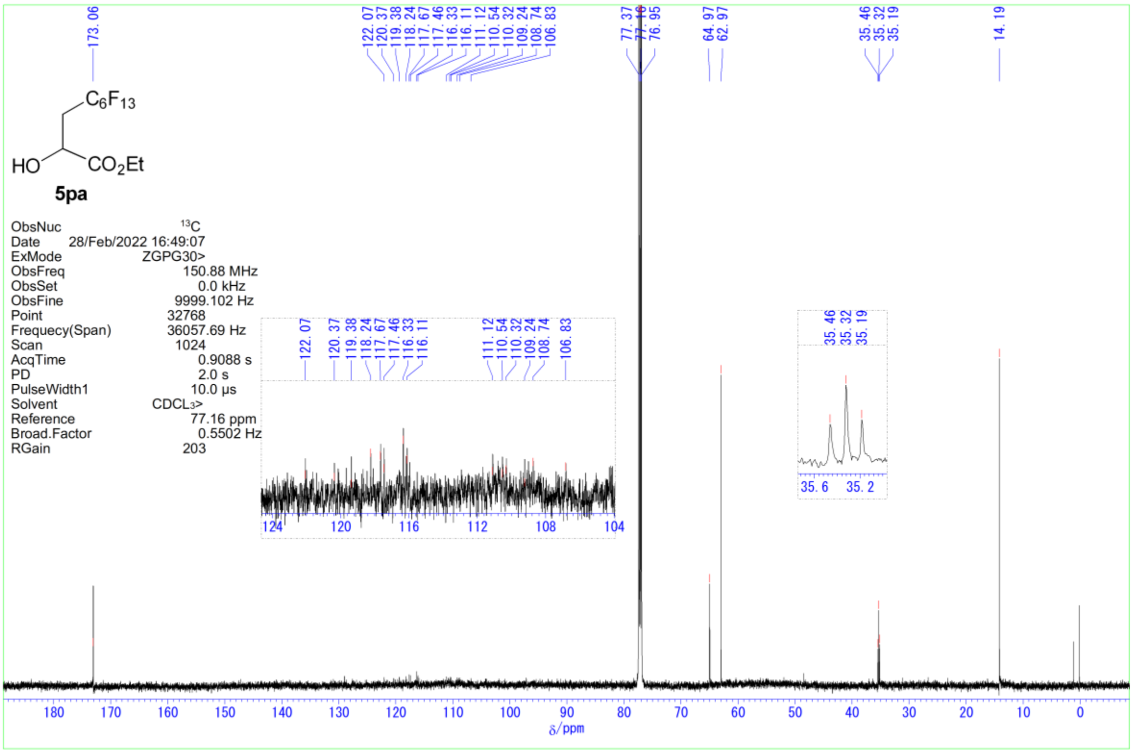

5pa: ^{19}F NMR ($CDCl_3$, 376 MHz)

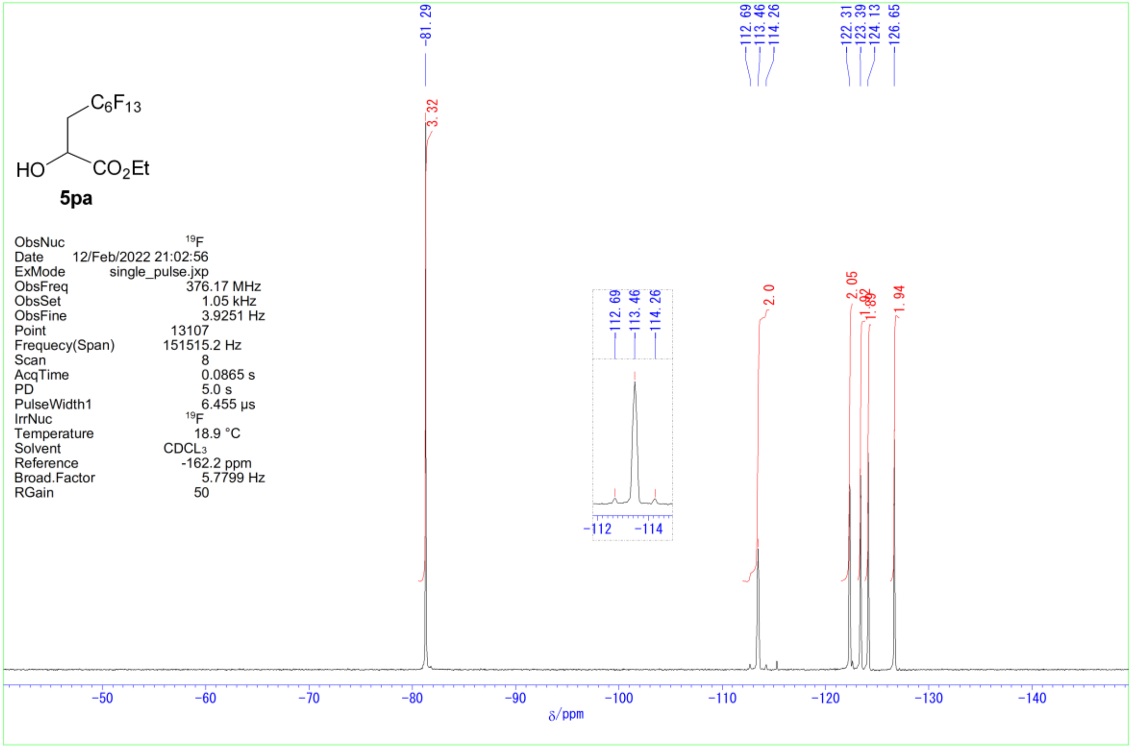

5qa: ^{1}H NMR ($CDCl_3$, 400 MHz)

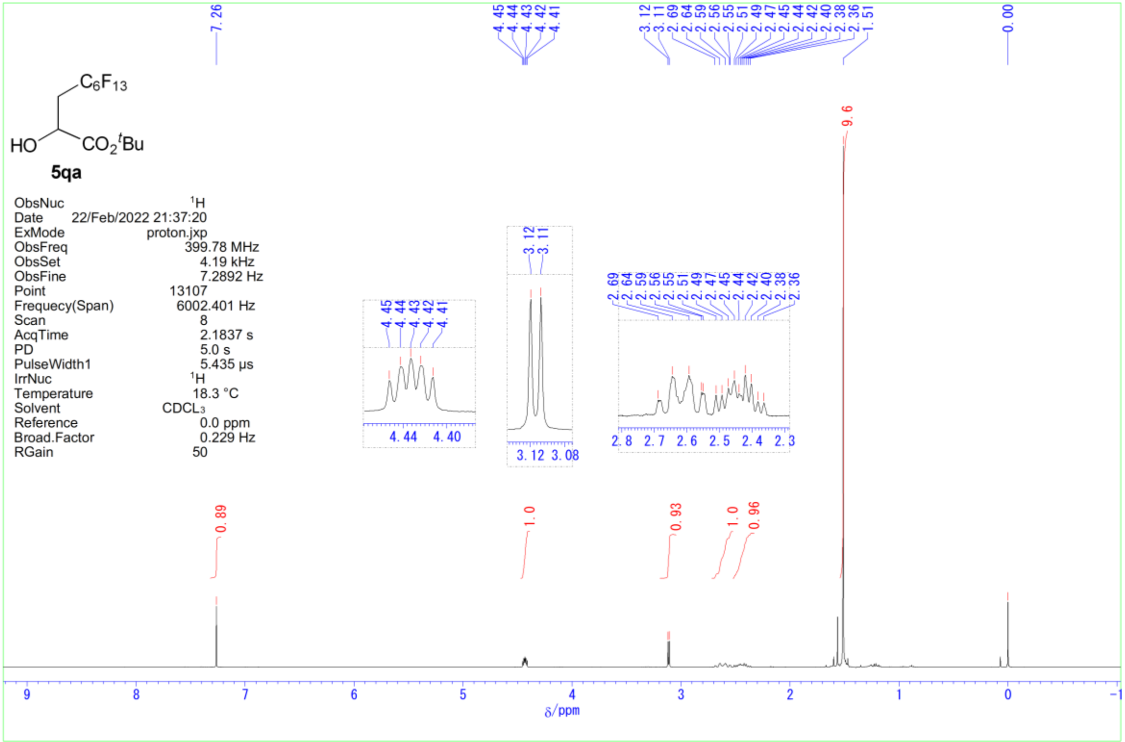

5qa: ^{13}C NMR ($CDCl_3$, 151 MHz)

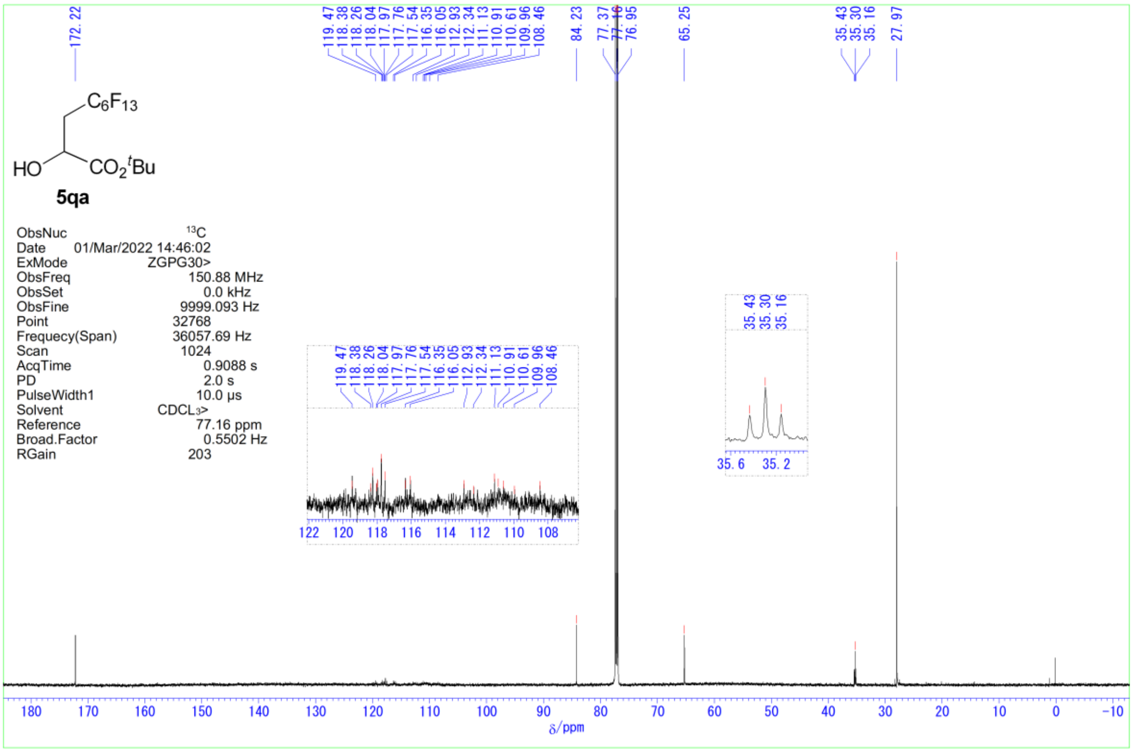

5qa: ^{19}F NMR (CDCl$_3$, 376 MHz)

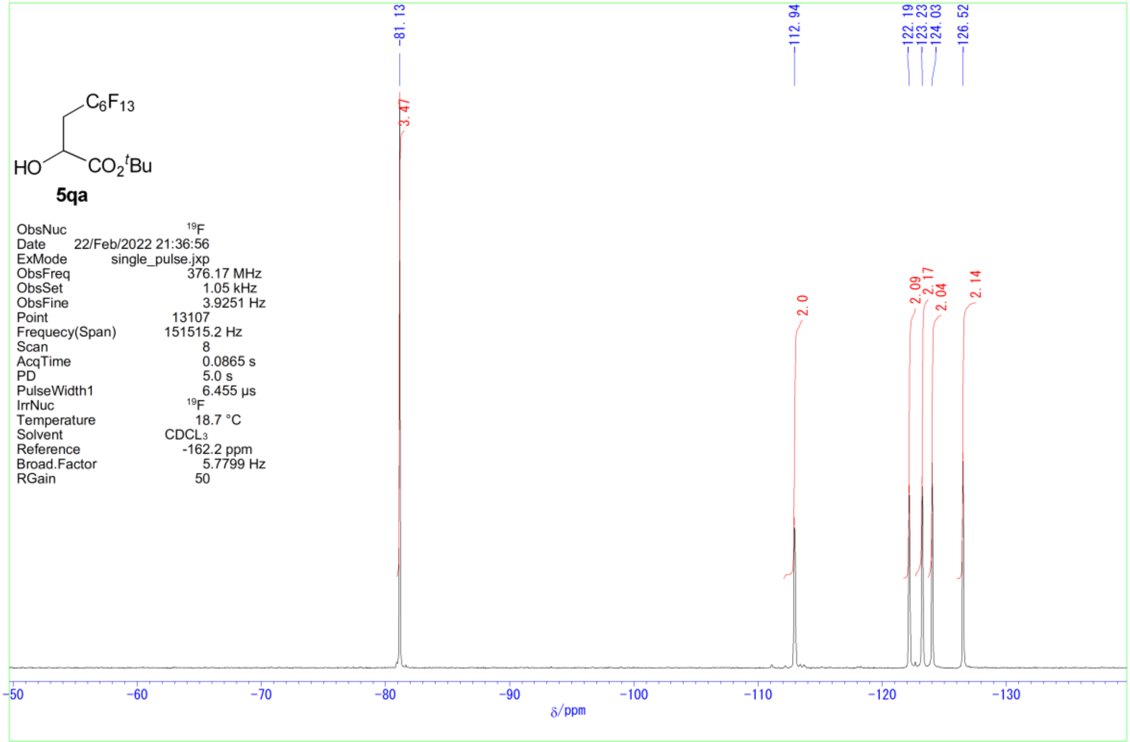

5ra: ^{1}H NMR (CDCl$_3$, 400 MHz)

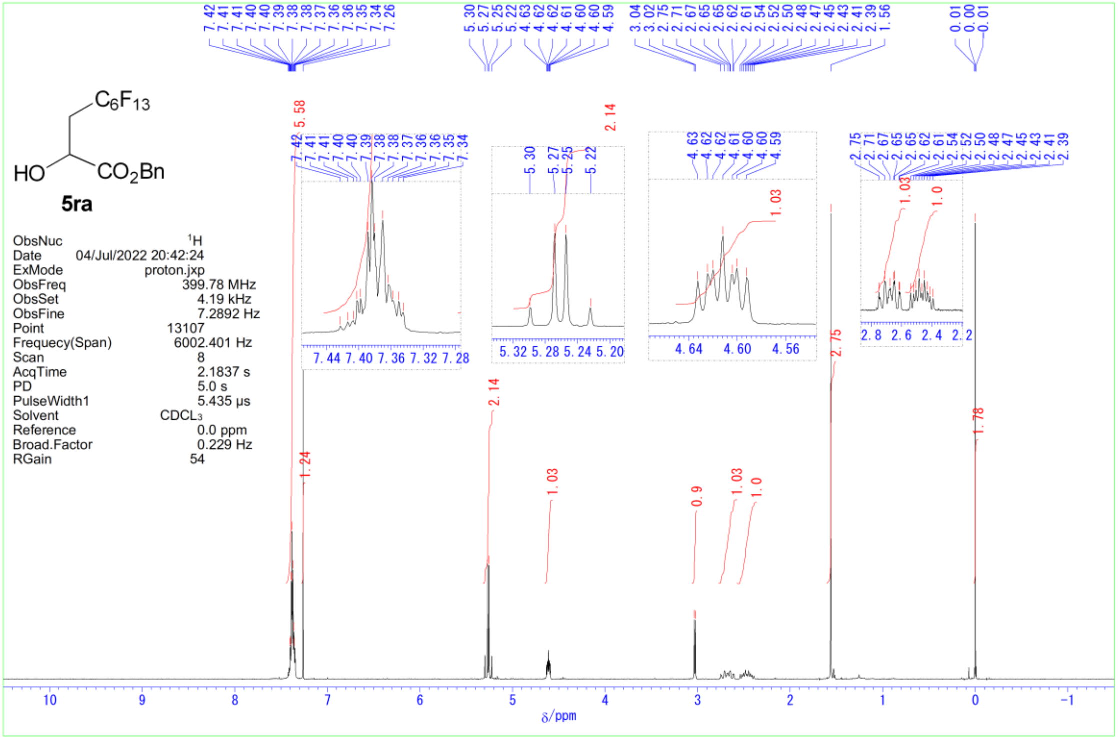

5ra: ^{13}C NMR ($CDCl_3$, 151 MHz)

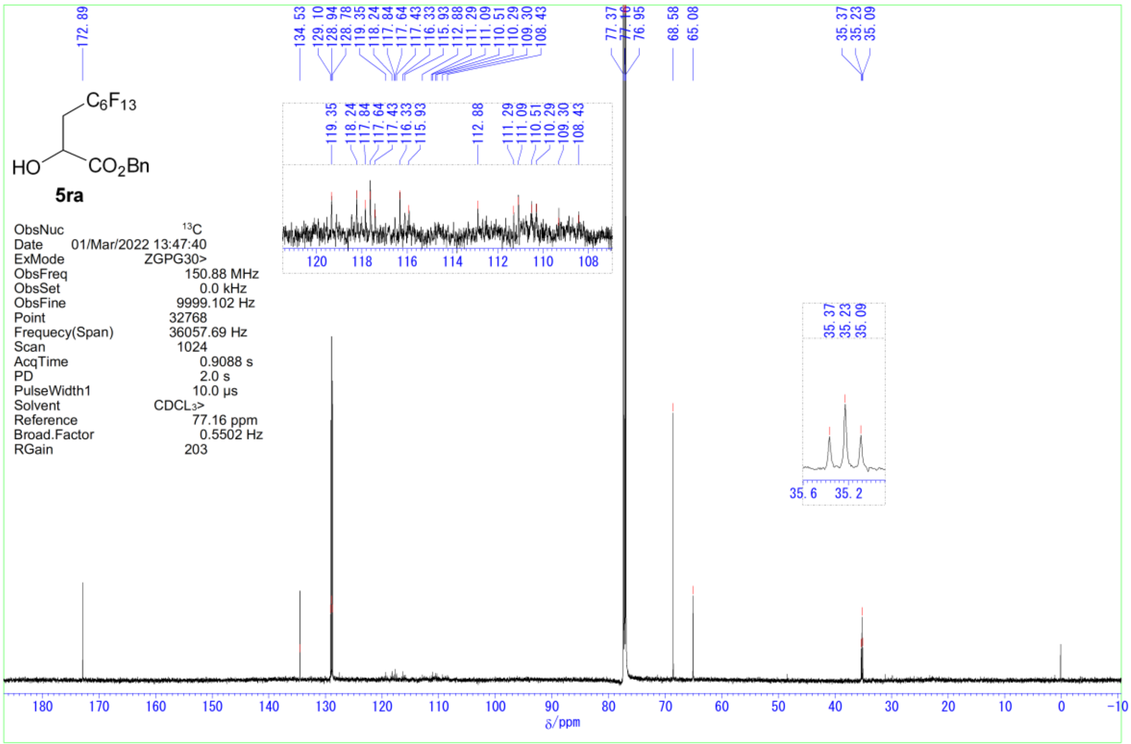

5ra: ^{19}F NMR ($CDCl_3$, 376 MHz)

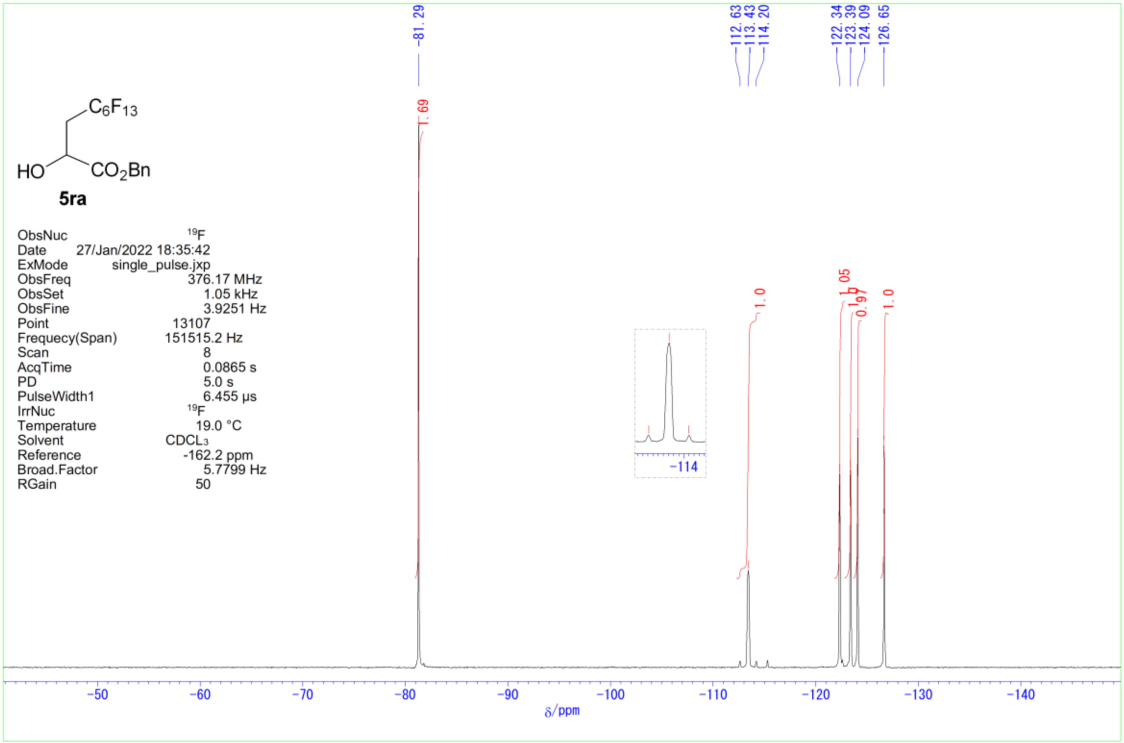

5sa: ^{1}H NMR (CDCl$_3$, 500 MHz)

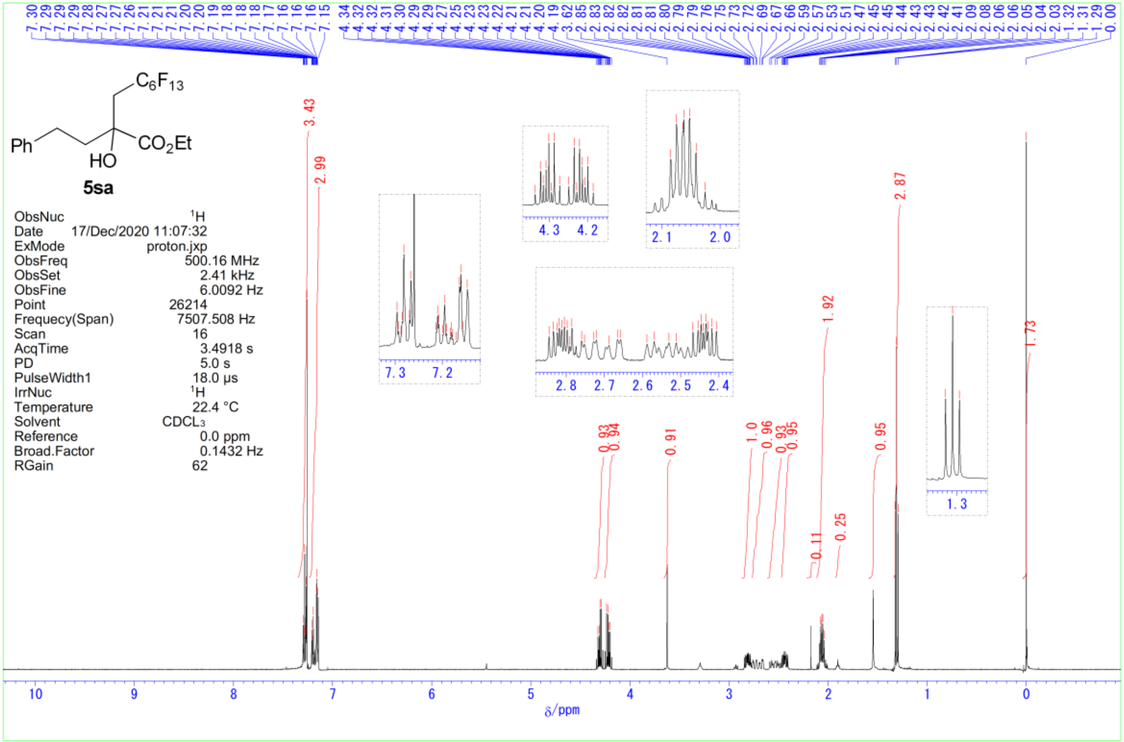

5sa: ^{13}C NMR (CDCl$_3$, 151 MHz)

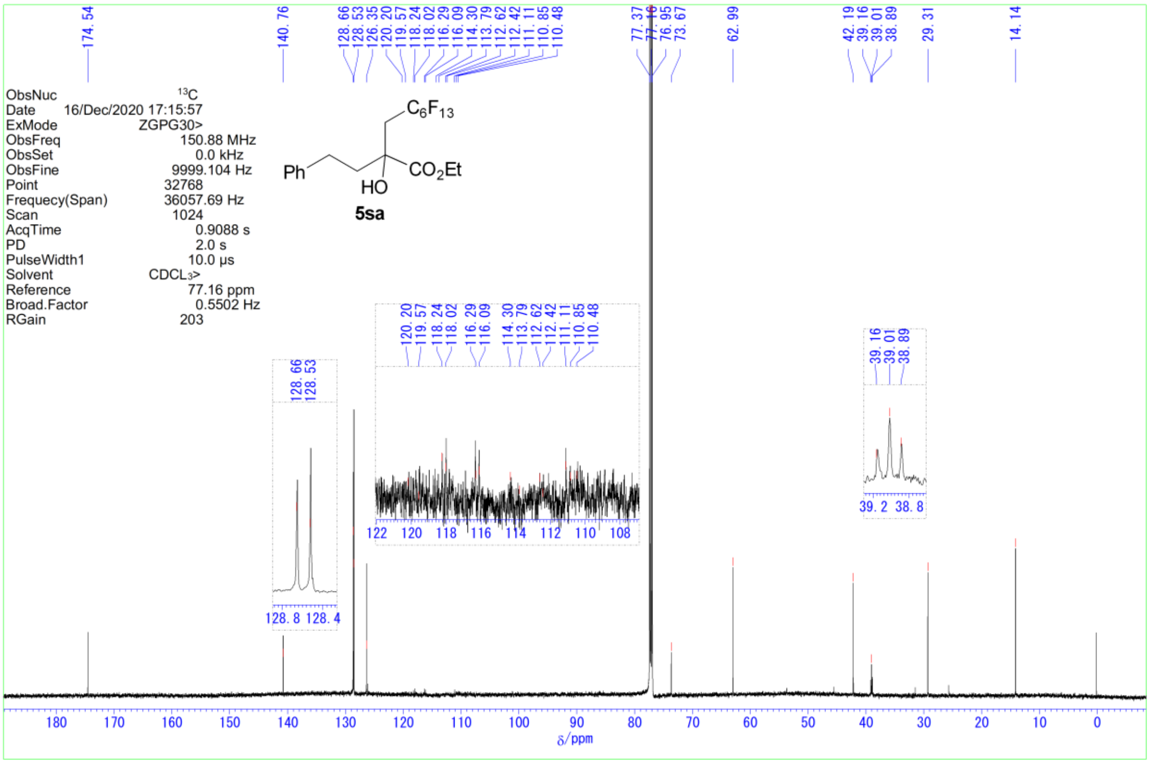

5sa: ^{19}F NMR ($CDCl_3$, 471 MHz)

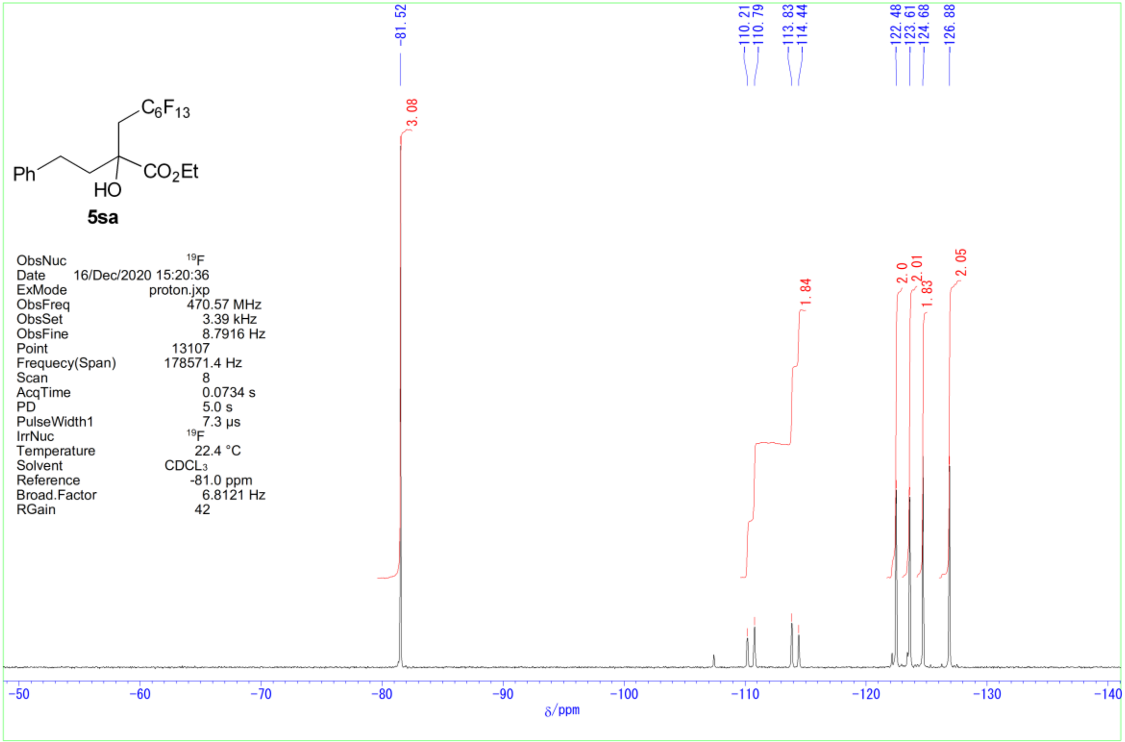

5ta: ^{1}H NMR ($CDCl_3$, 400 MHz)

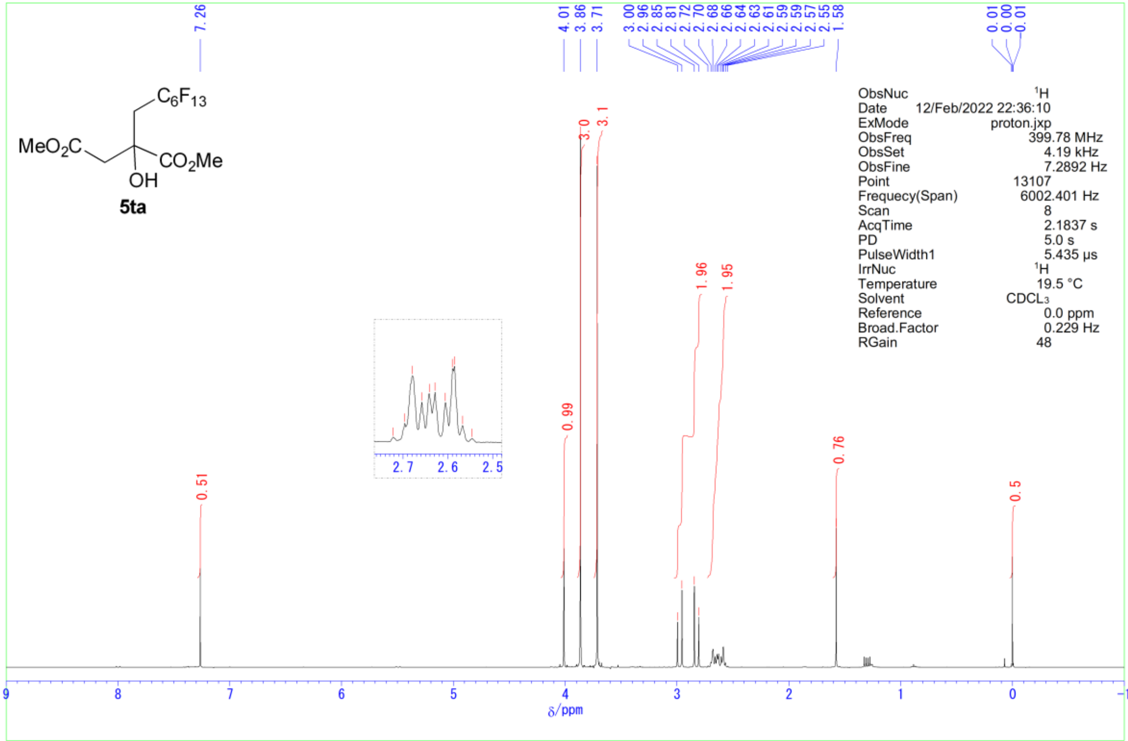

5ta: ^{13}C NMR ($CDCl_3$, 151 MHz)

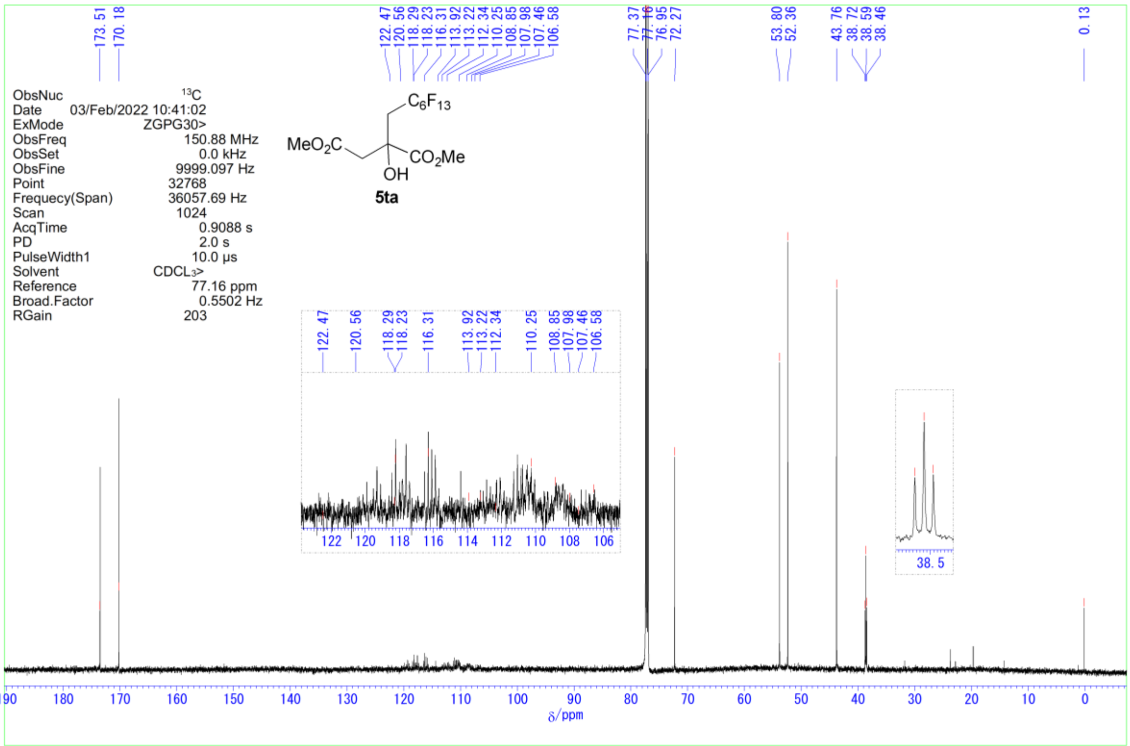

5ta:^{19}F NMR ($CDCl_3$, 376 MHz)

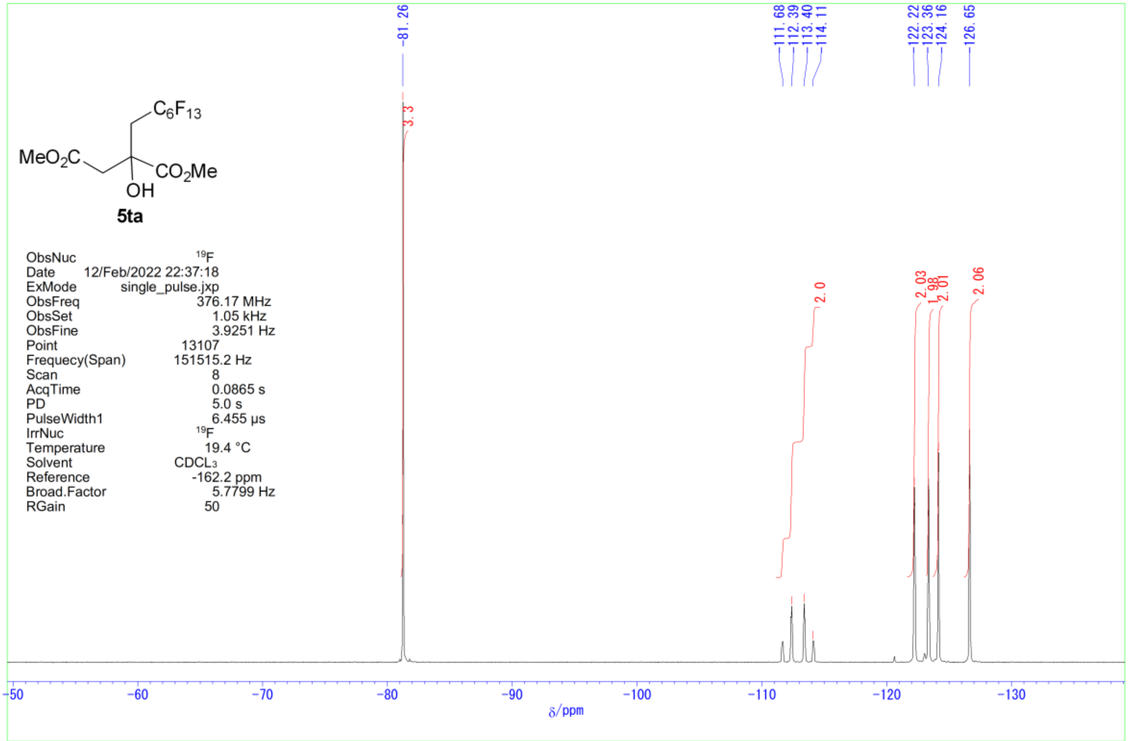

5ua: ^{1}H NMR ($CDCl_3$, 400 MHz)

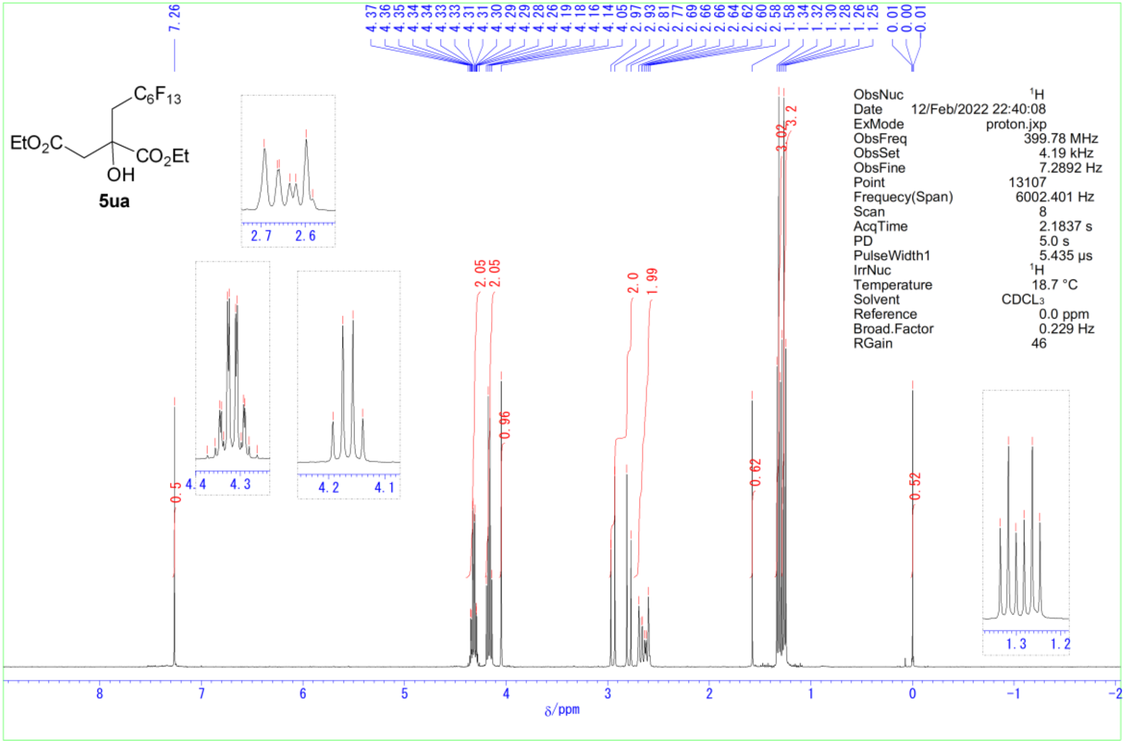

5ua: ^{13}C NMR ($CDCl_3$, 151 MHz)

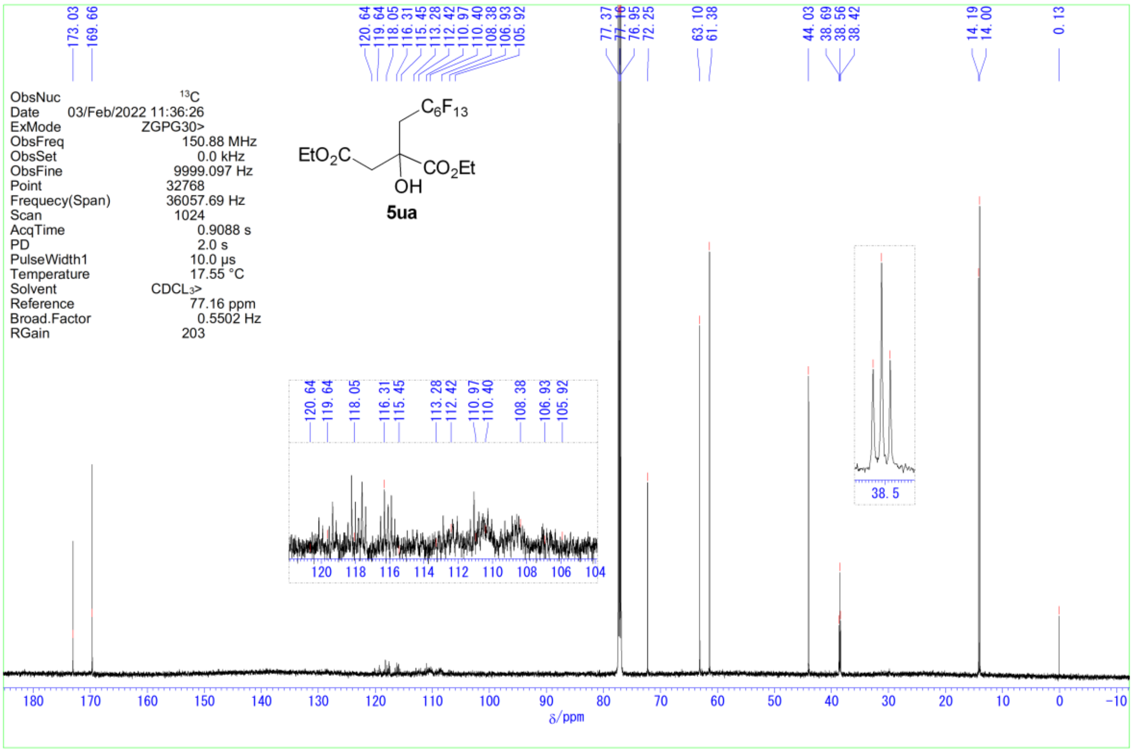

5ua: ^{19}F NMR ($CDCl_3$, 376 MHz)

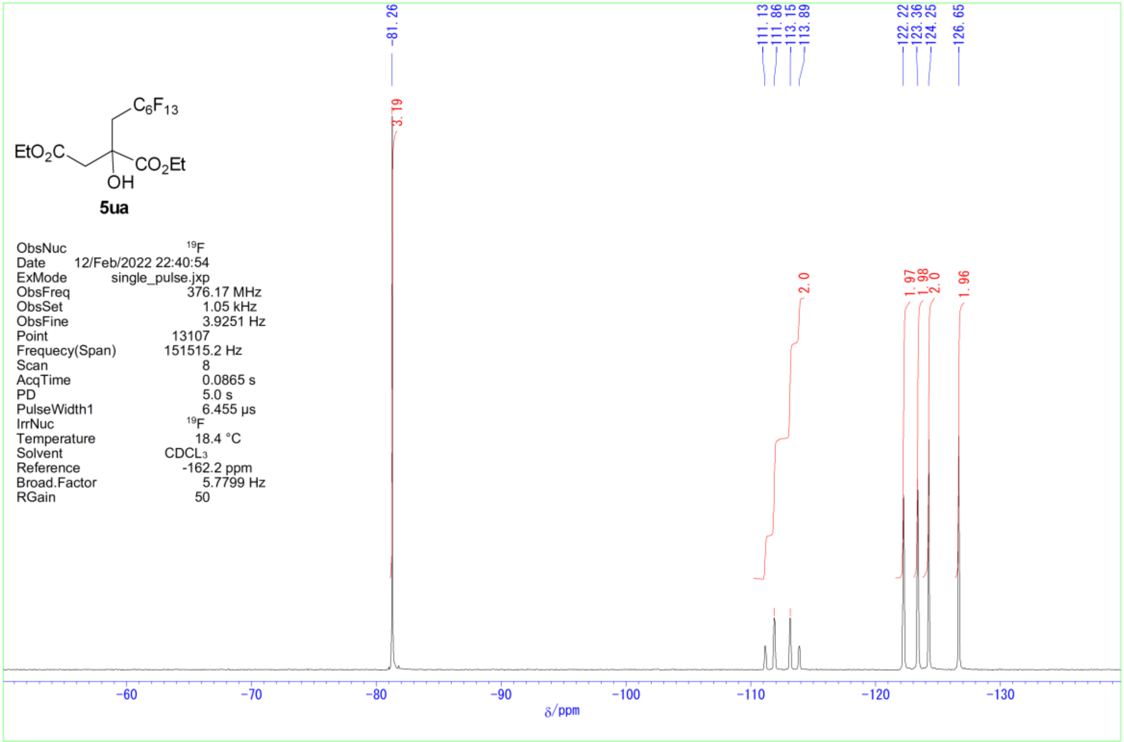

5ab: ^{1}H NMR ($CDCl_3$, 400 MHz)

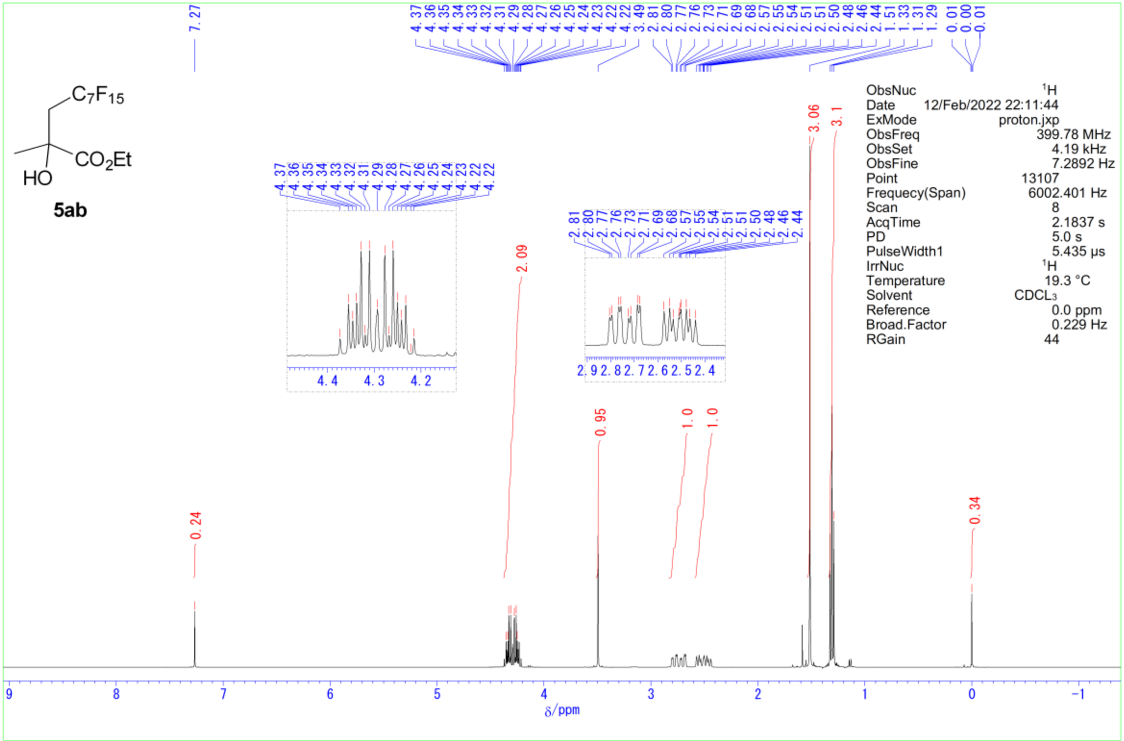

5ab: ^{13}C NMR (CDCl$_3$, 151 MHz)

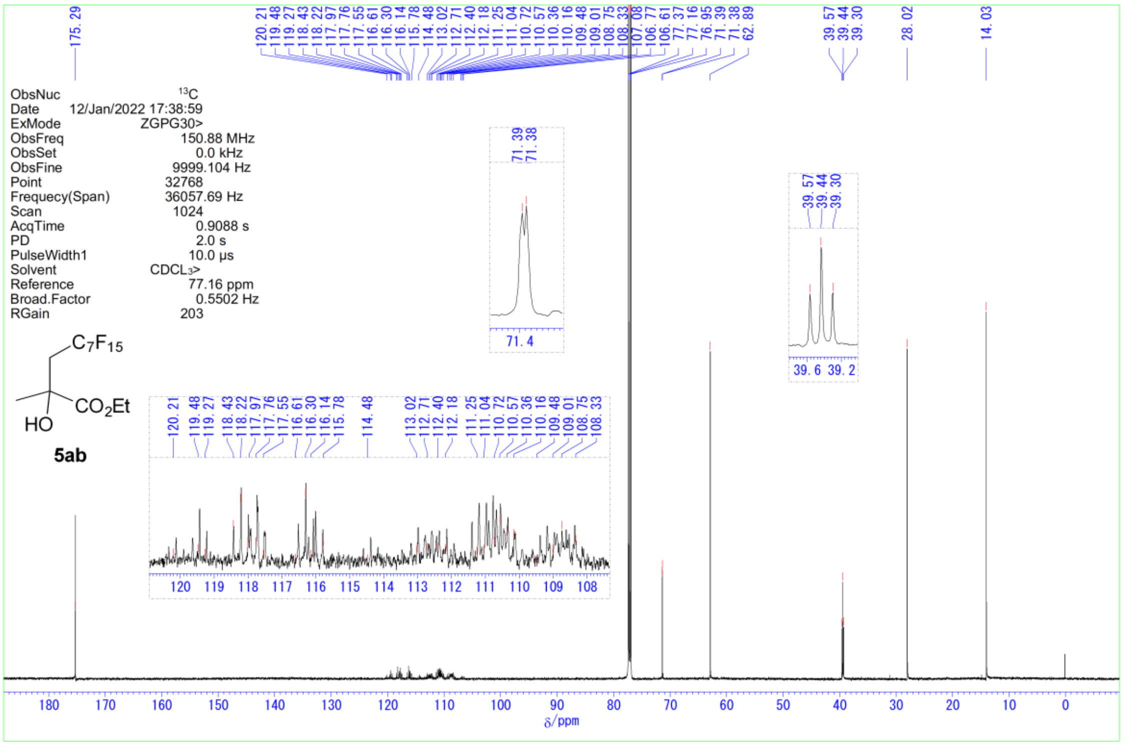

5ab: ^{19}F NMR (CDCl$_3$, 376 MHz)

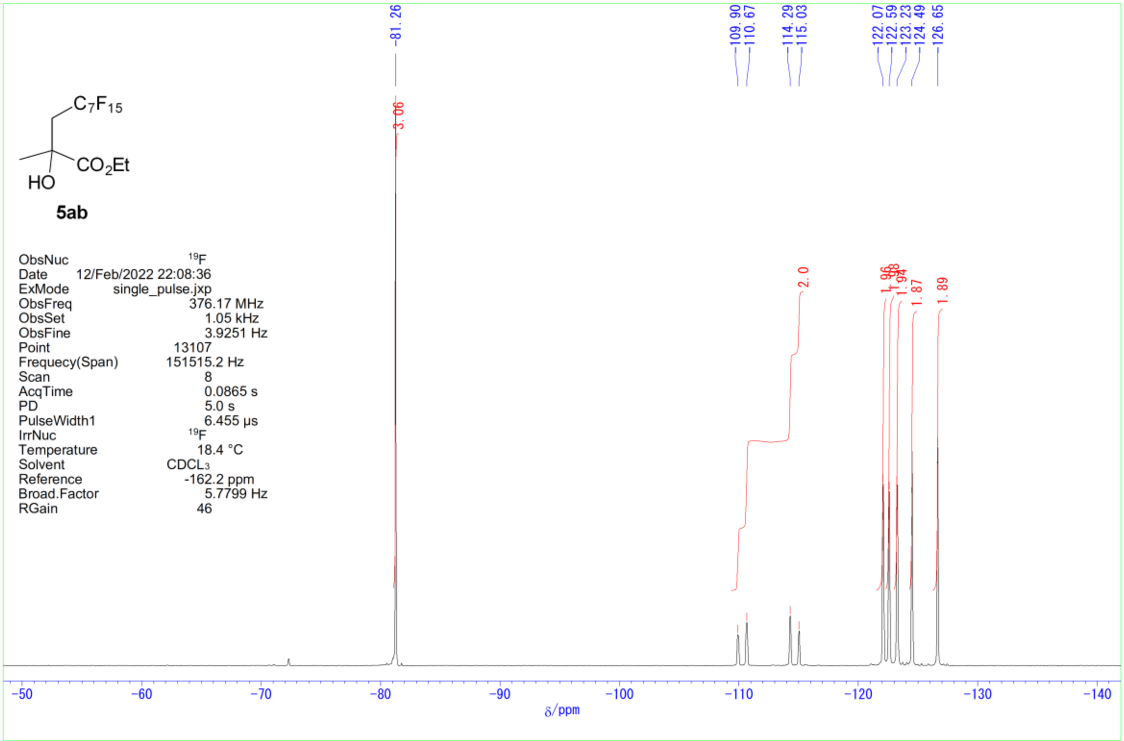

5ac: ^{1}H NMR ($CDCl_3$, 400 MHz)

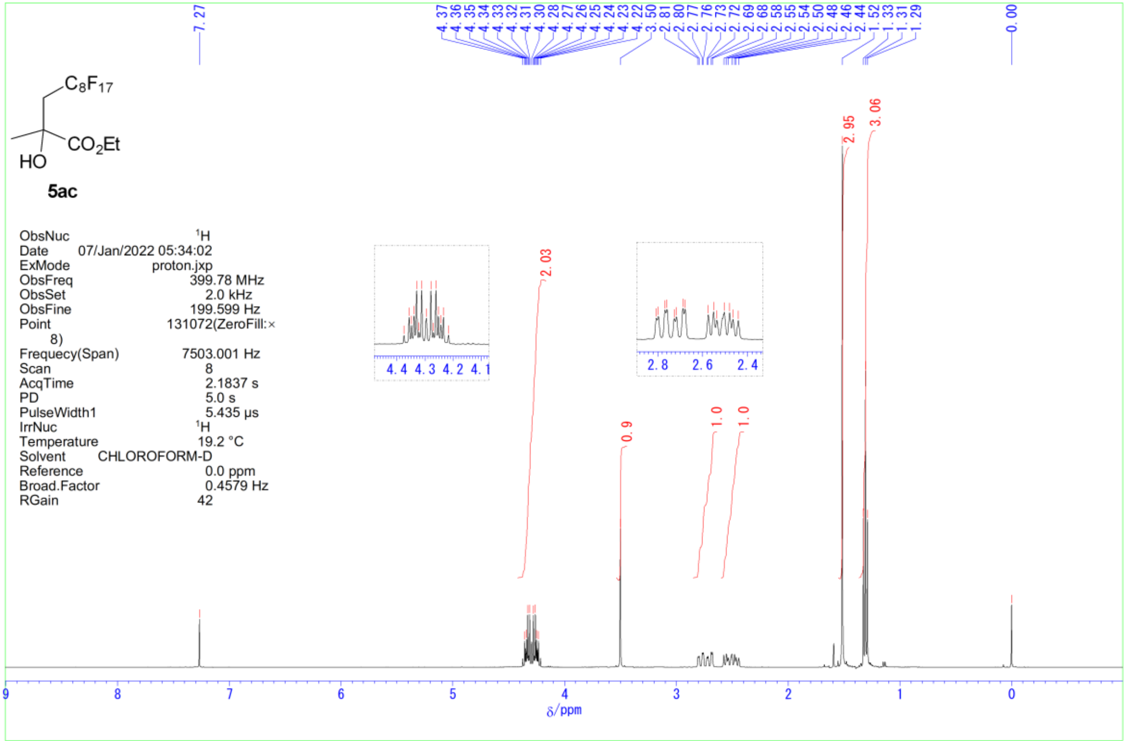

5ac: ^{13}C NMR ($CDCl_3$, 151 MHz)

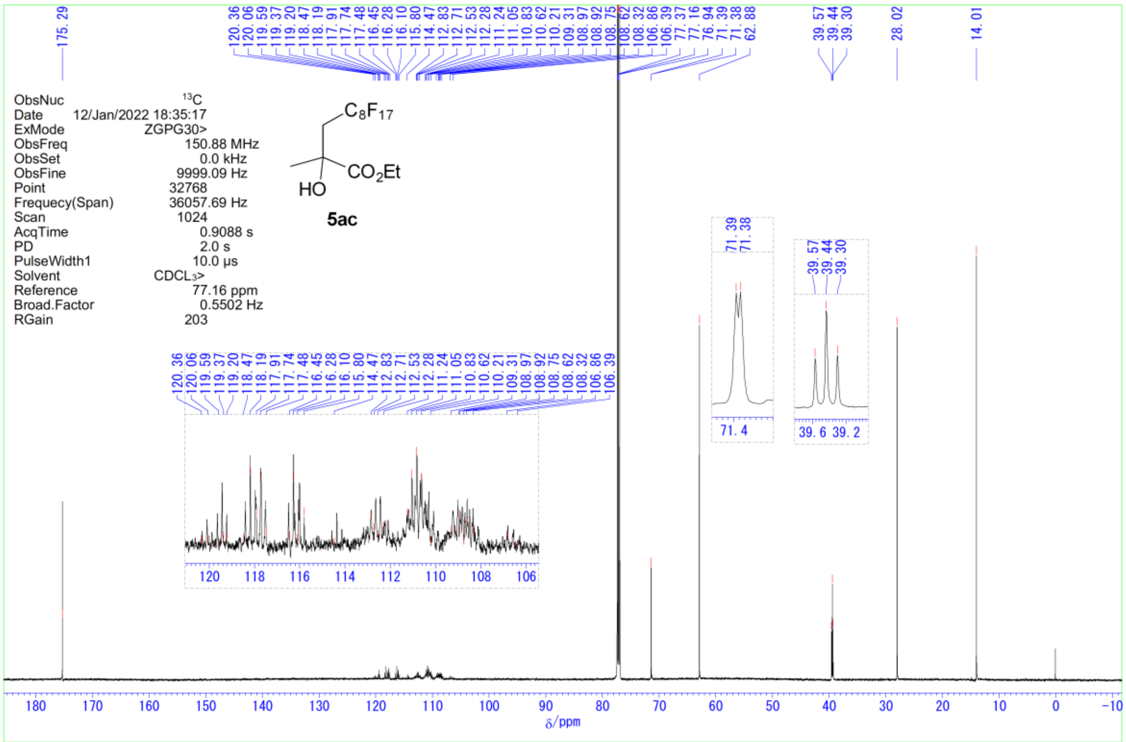

5ac: ^{19}F NMR ($CDCl_3$, 376 MHz)

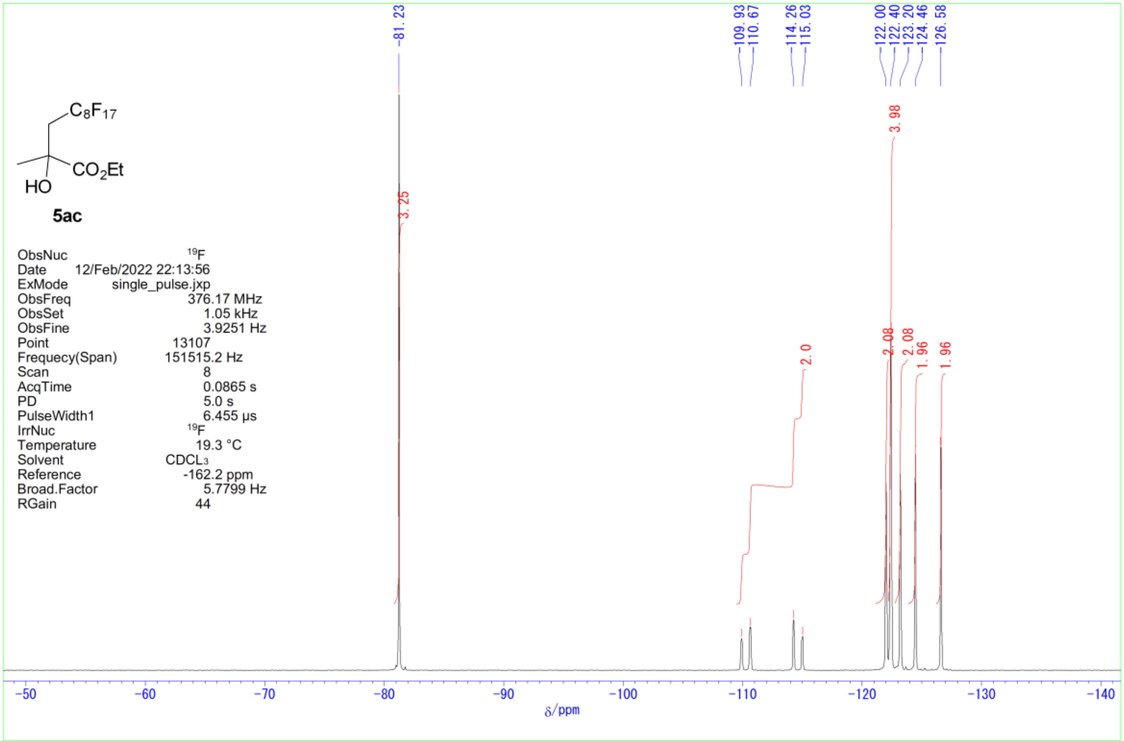

5ad: ^{1}H NMR ($CDCl_3$, 400 MHz)

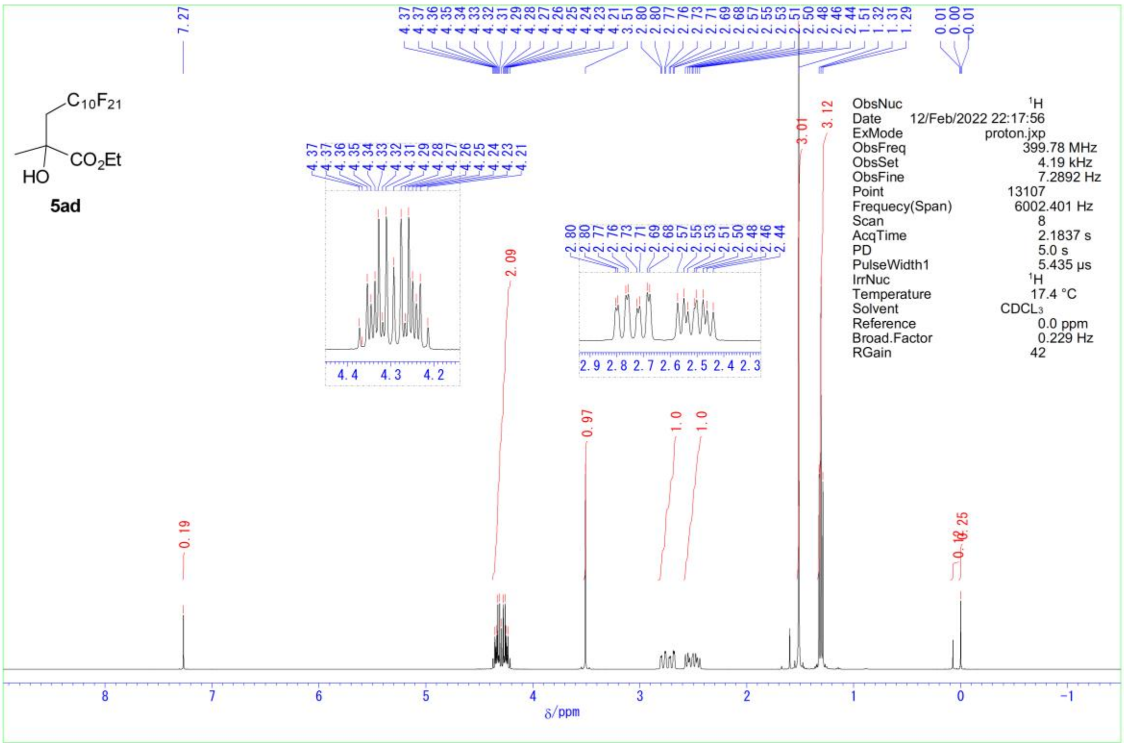

5ad: ^{13}C NMR ($CDCl_3$, 151 MHz)

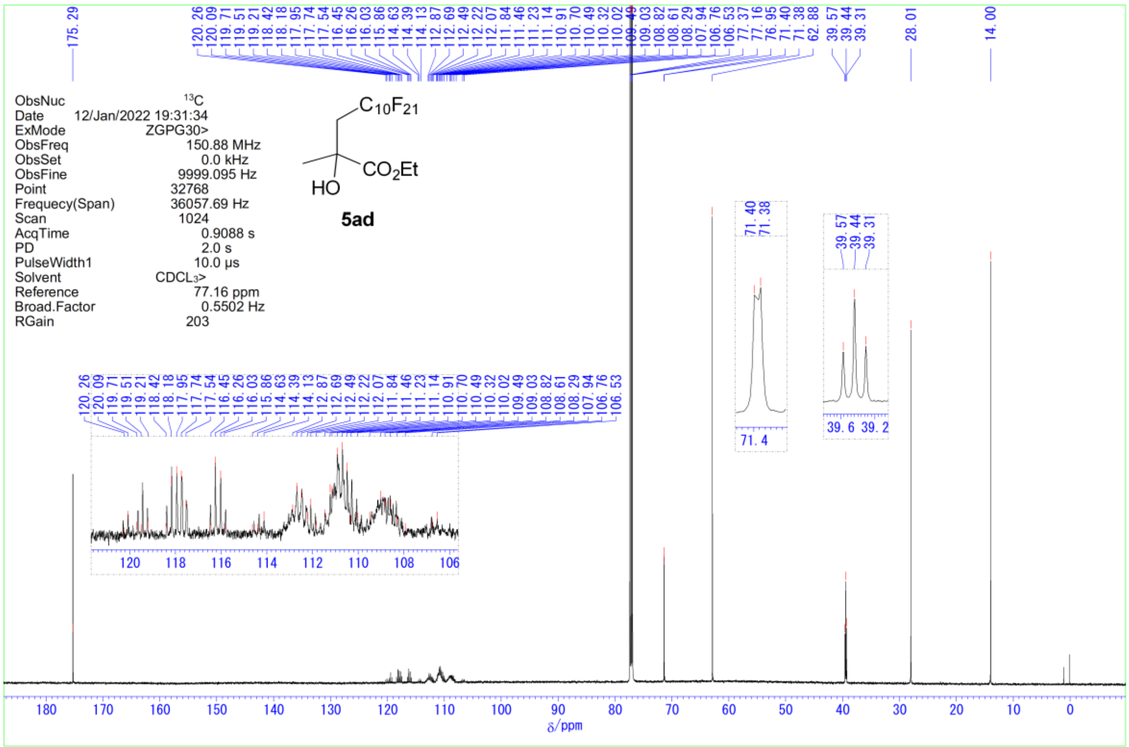

5ad: ^{19}F NMR ($CDCl_3$, 376 MHz)

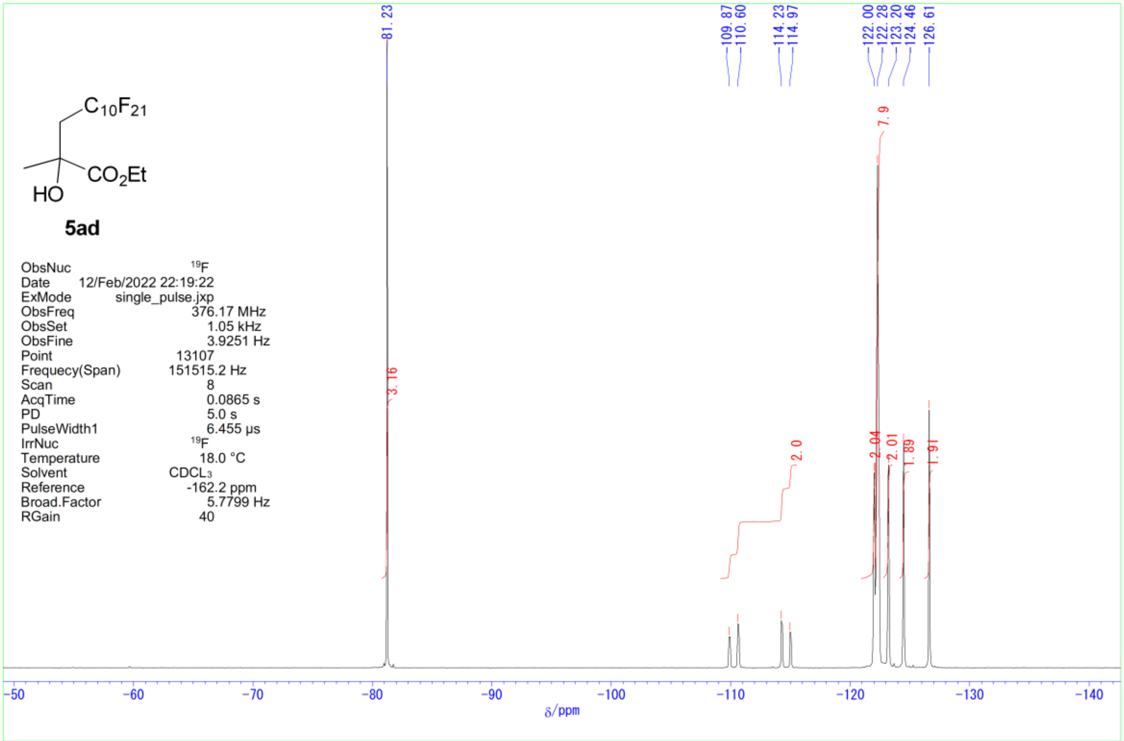

5ae: ^{1}H NMR ($CDCl_3$, 400 MHz)

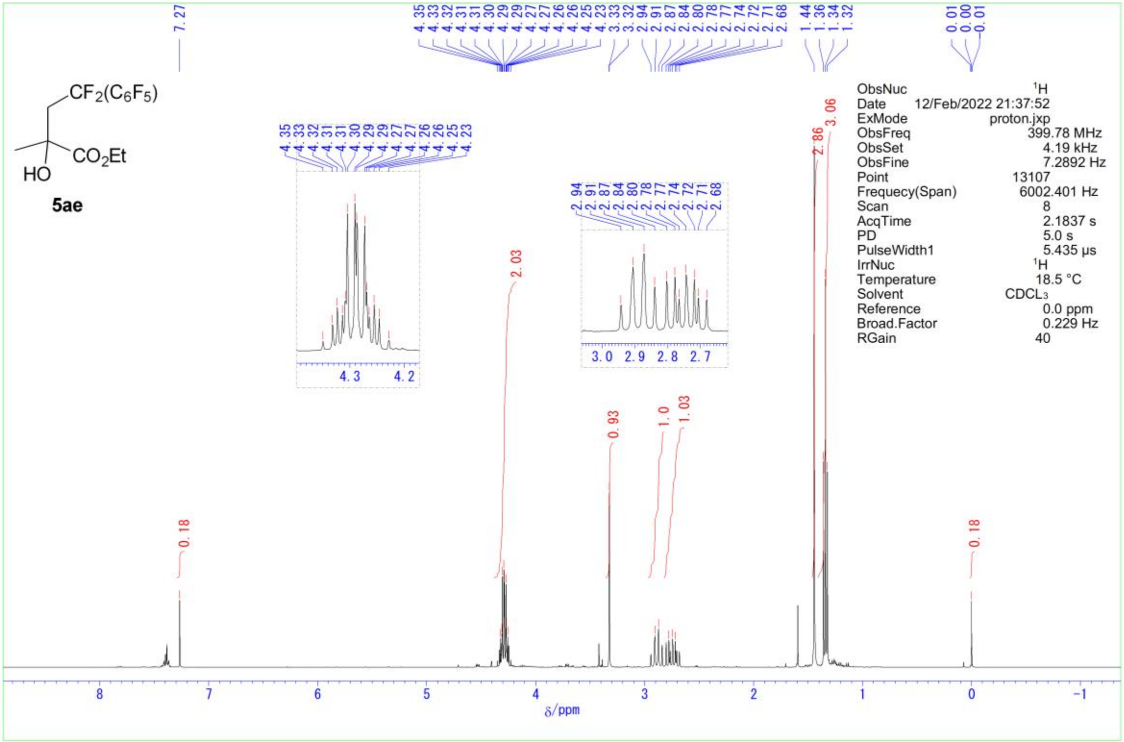

5ae: ^{13}C NMR ($CDCl_3$, 151 MHz)

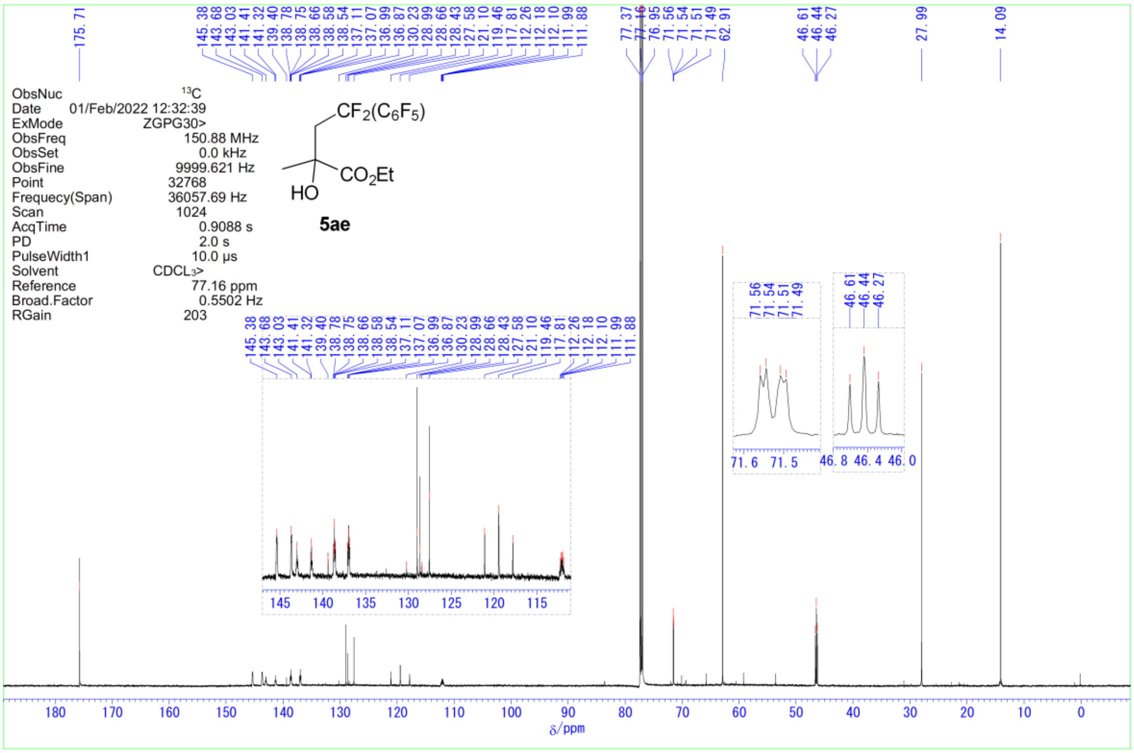

5ae: ^{19}F NMR ($CDCl_3$, 376 MHz)

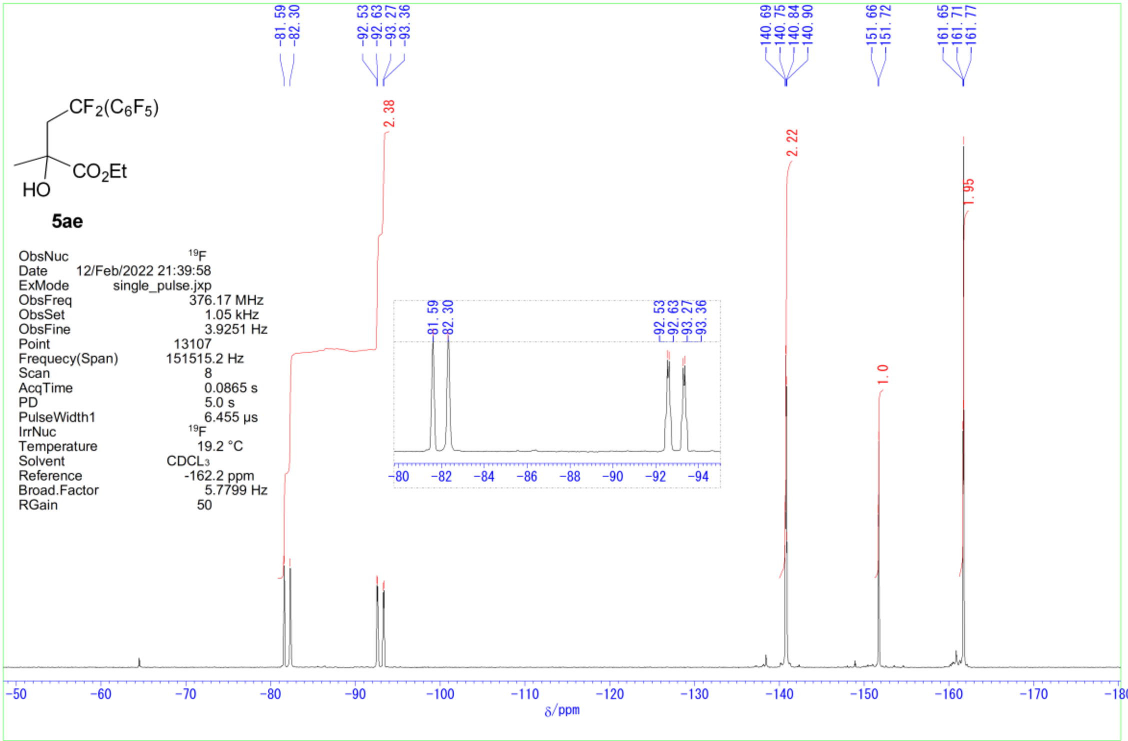

5df: ^{1}H NMR ($CDCl_3$, 400 MHz)

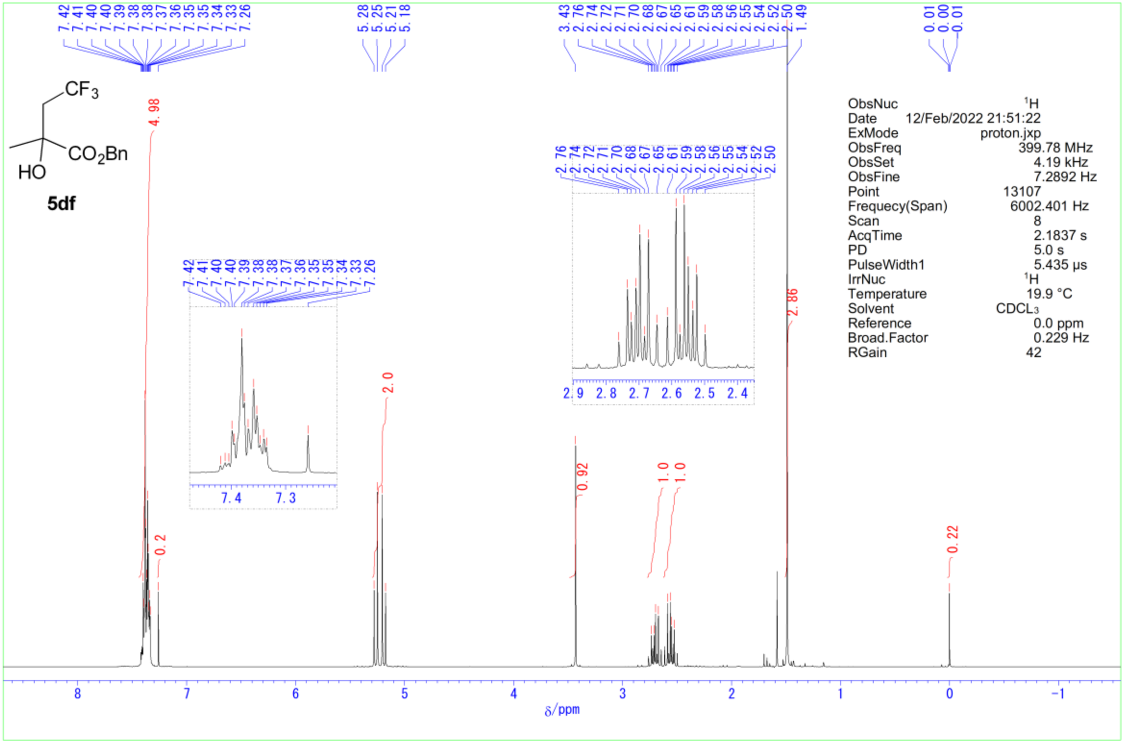

5df: ^{13}C NMR (CDCl$_3$, 151 MHz)

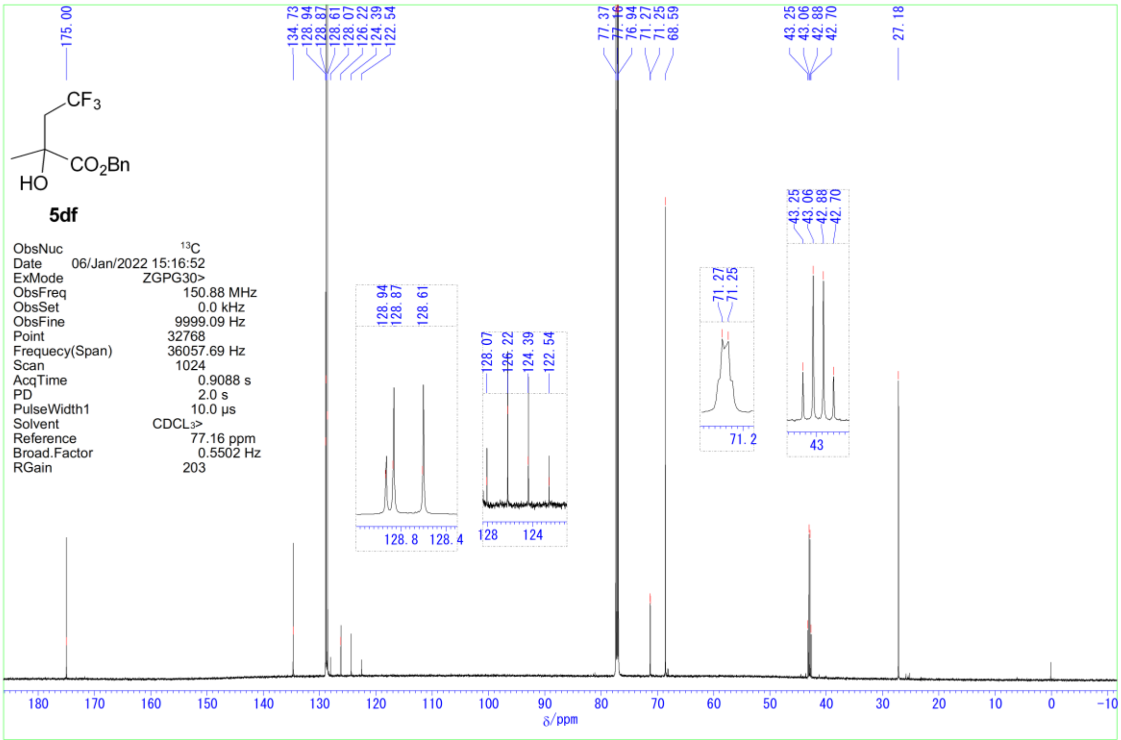

5df: ^{19}F NMR (CDCl$_3$, 376 MHz)

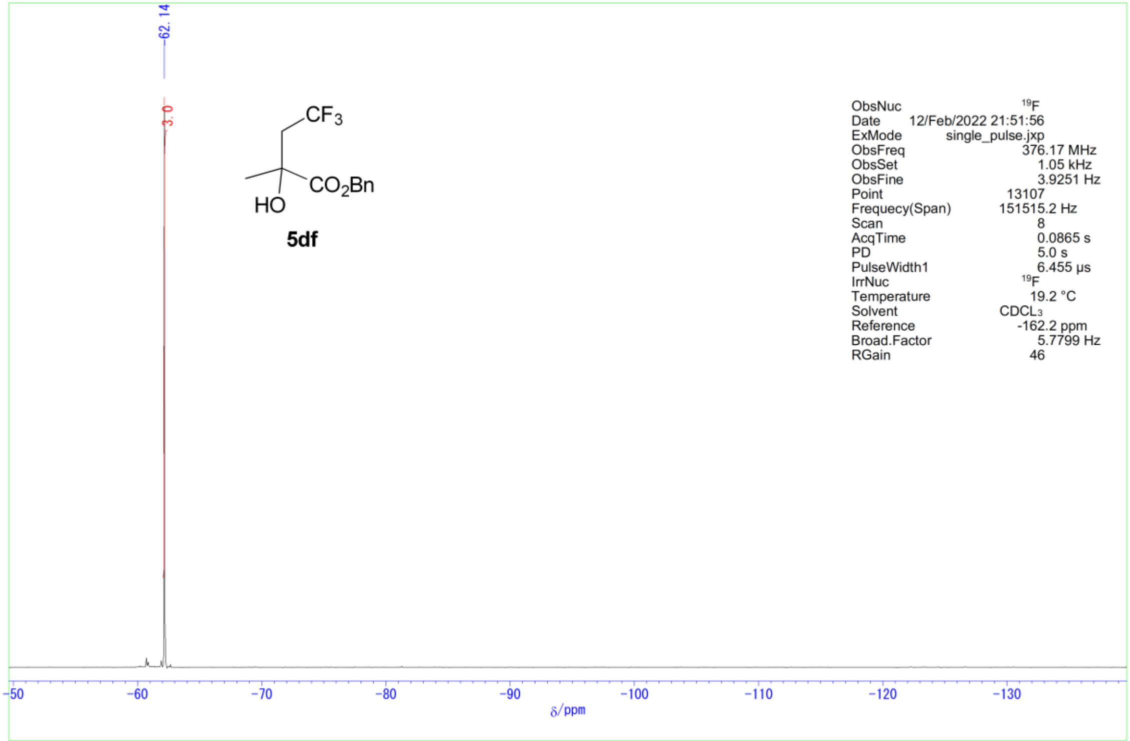

5dg: ^{1}H NMR ($CDCl_3$, 400 MHz)

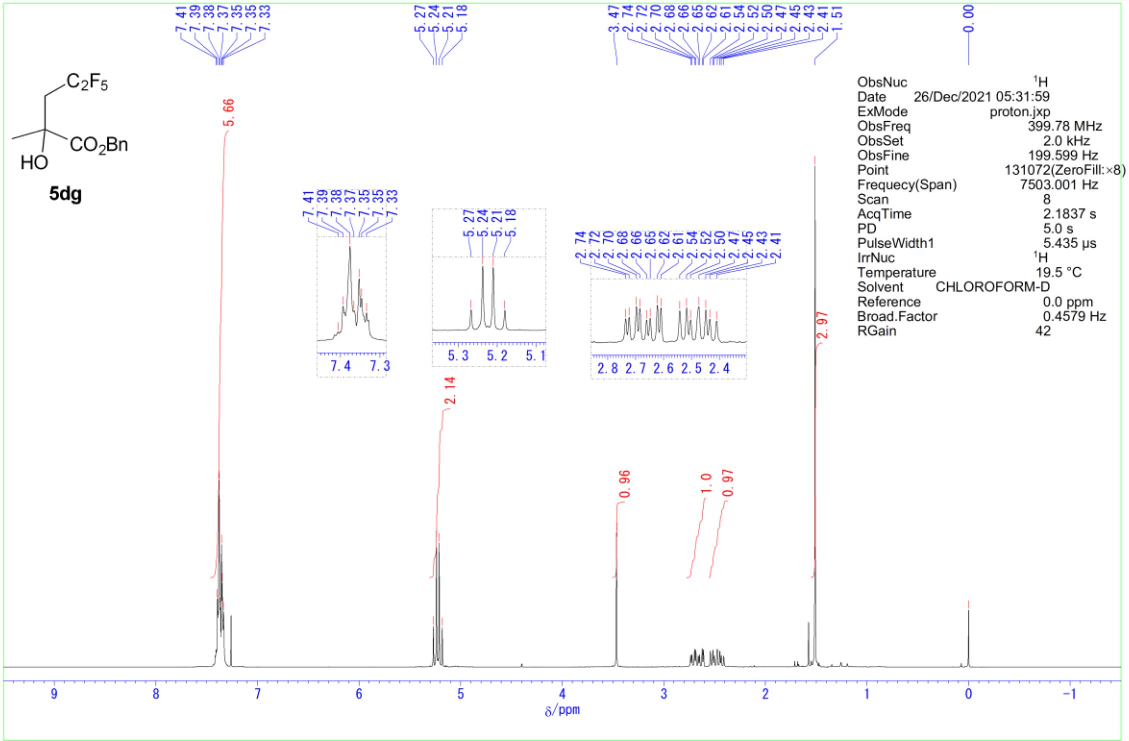

5dg: ^{13}C NMR ($CDCl_3$, 151 MHz)

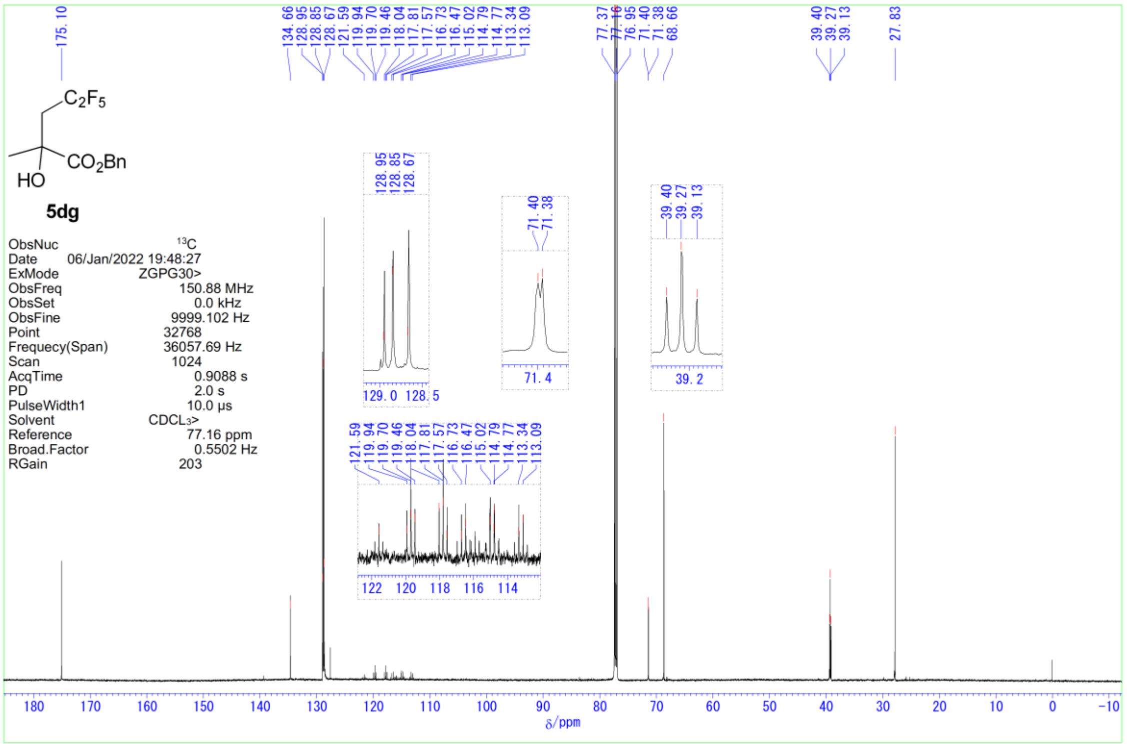

5dg: ^{19}F NMR ($CDCl_3$, 376 MHz)

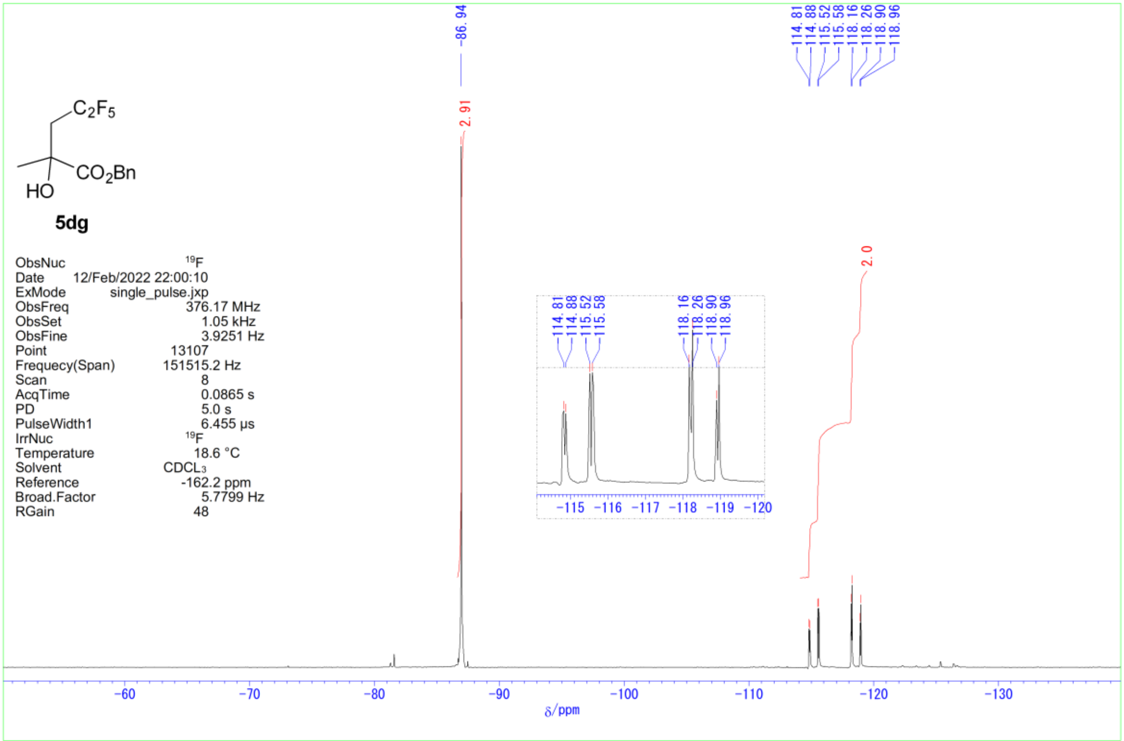

5dh: ^{1}H NMR ($CDCl_3$, 400 MHz)

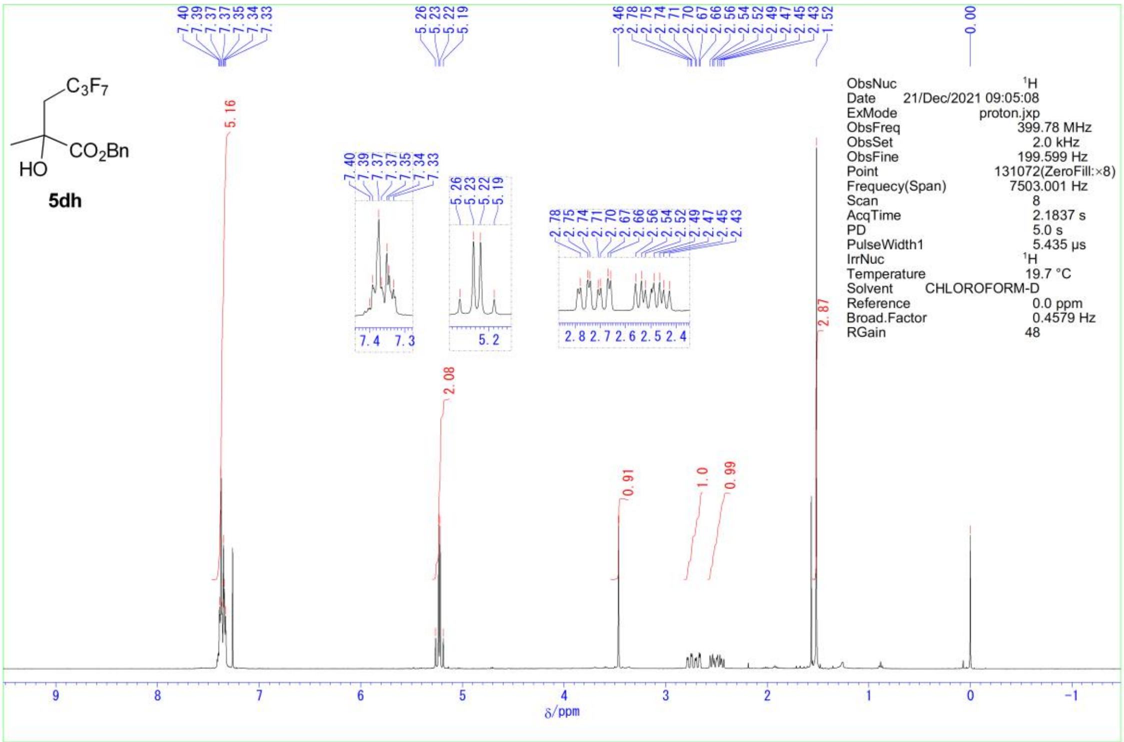

5dh: ^{13}C NMR ($CDCl_3$, 151 MHz)

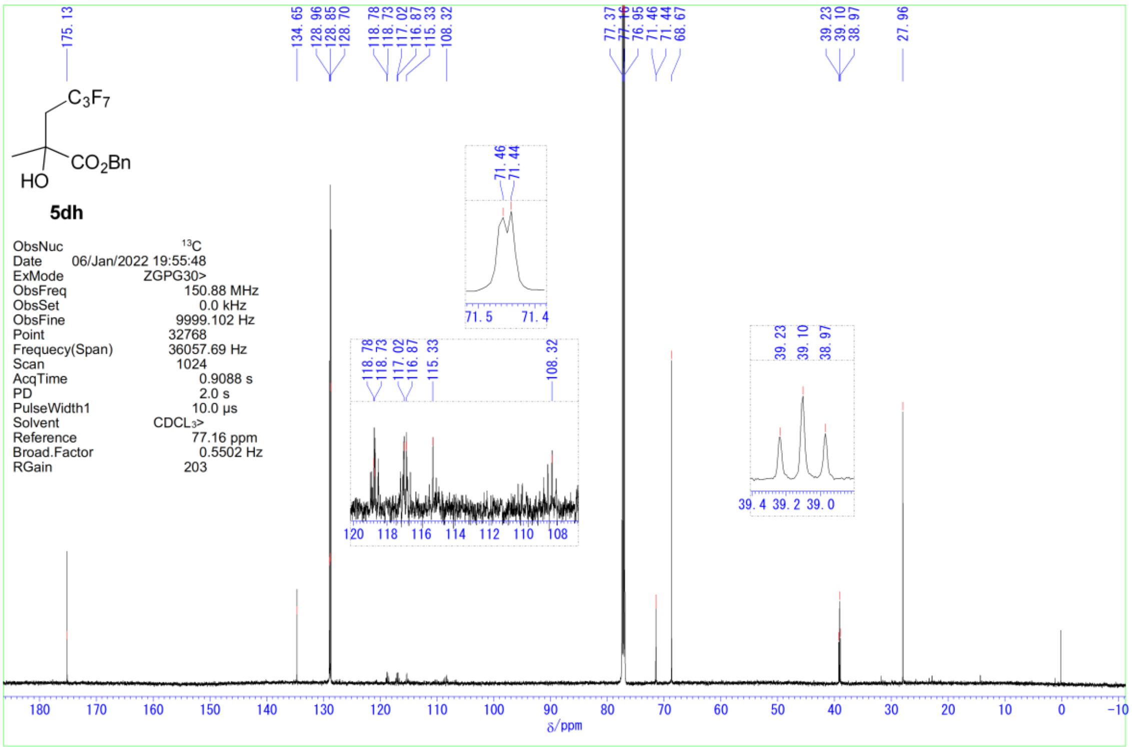

5dh: ^{19}F NMR ($CDCl_3$, 376 MHz)

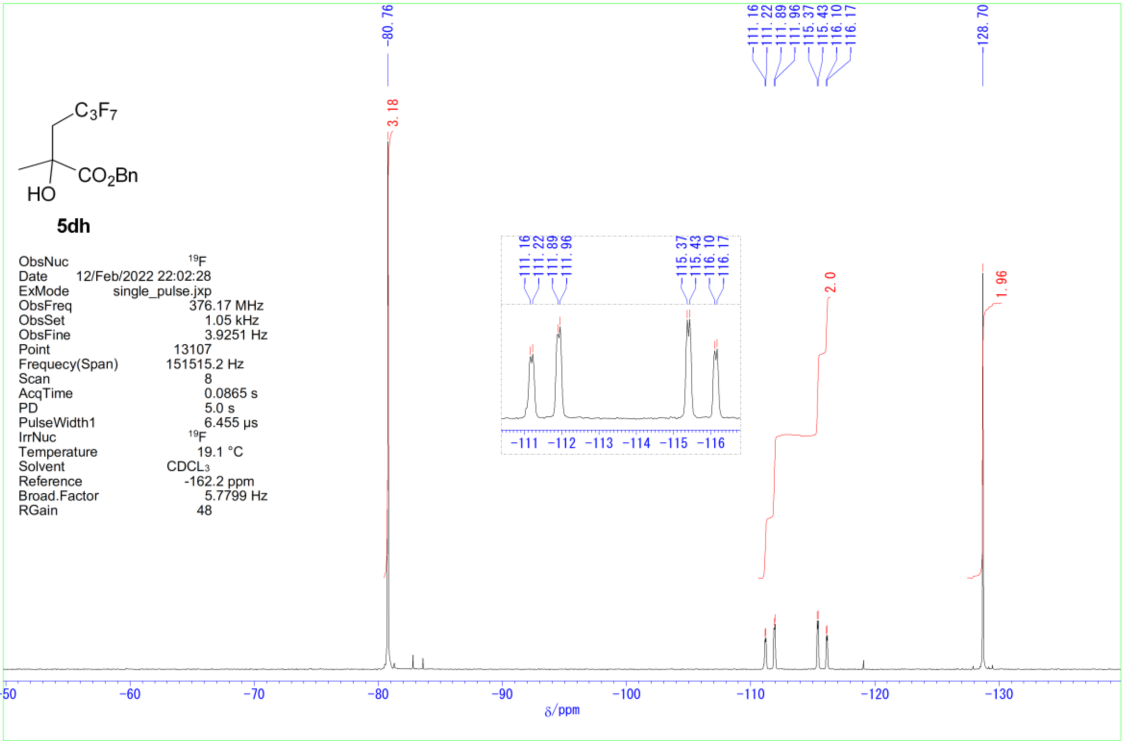

5di: ^{1}H NMR ($CDCl_3$, 400 MHz)

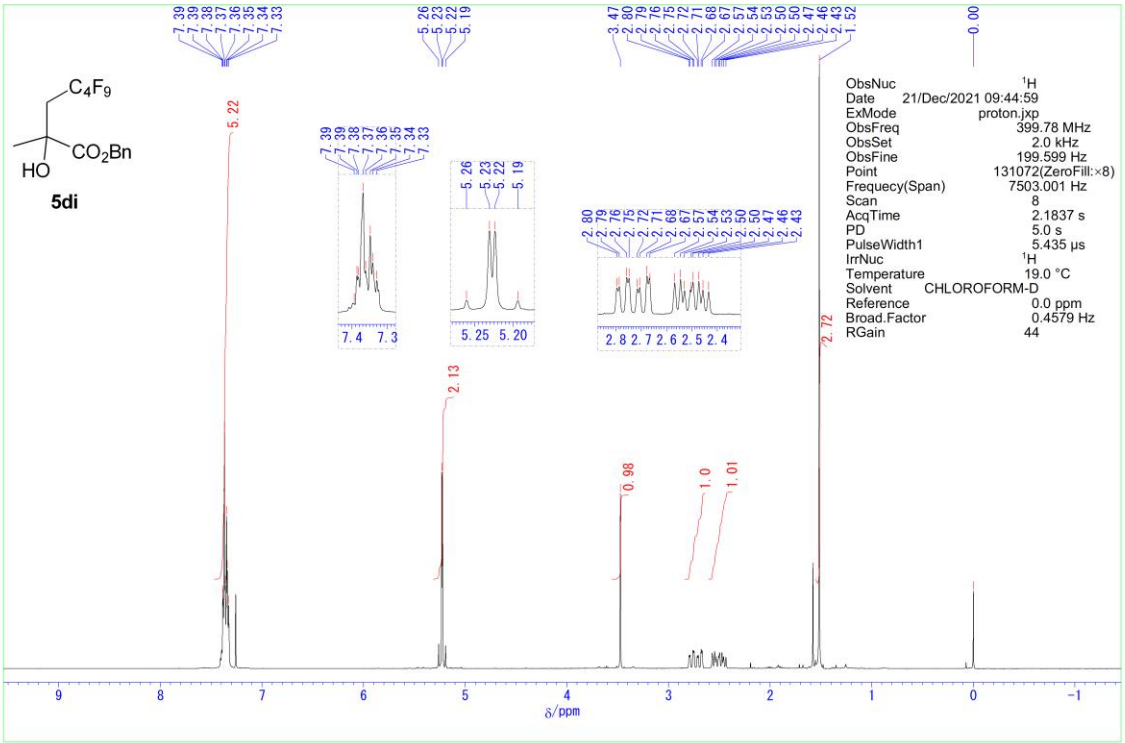

5di: ^{13}C NMR ($CDCl_3$, 151 MHz)

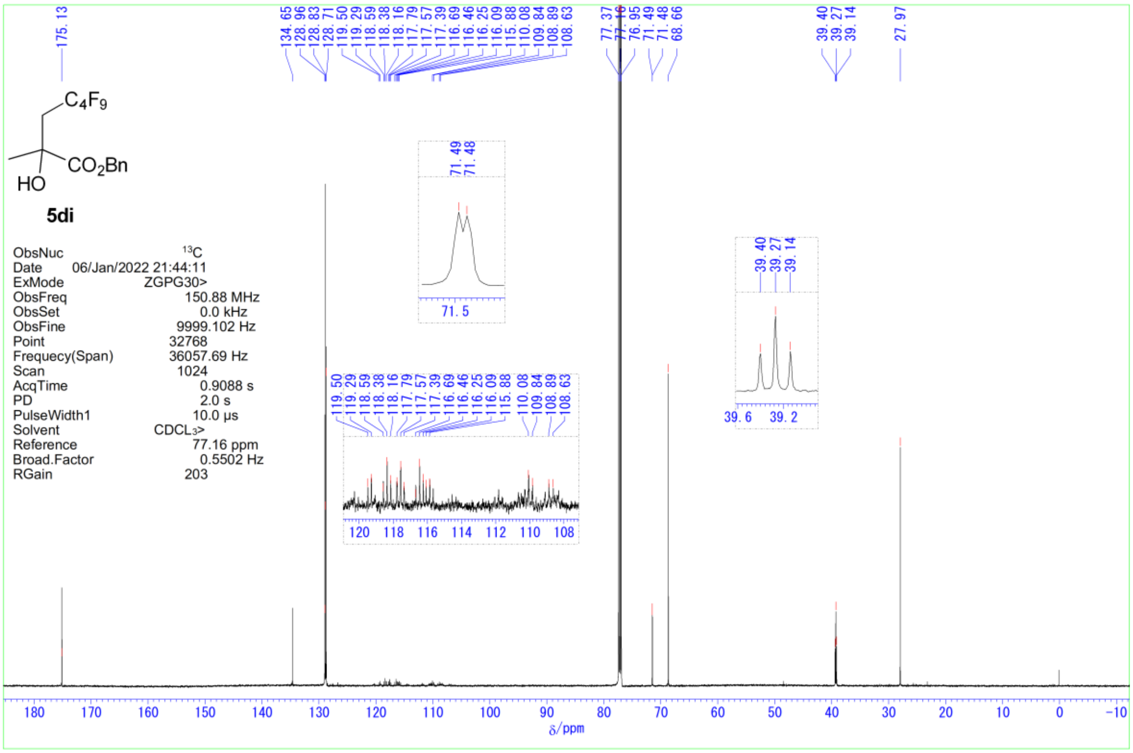

5di: ^{19}F NMR ($CDCl_3$, 376 MHz)

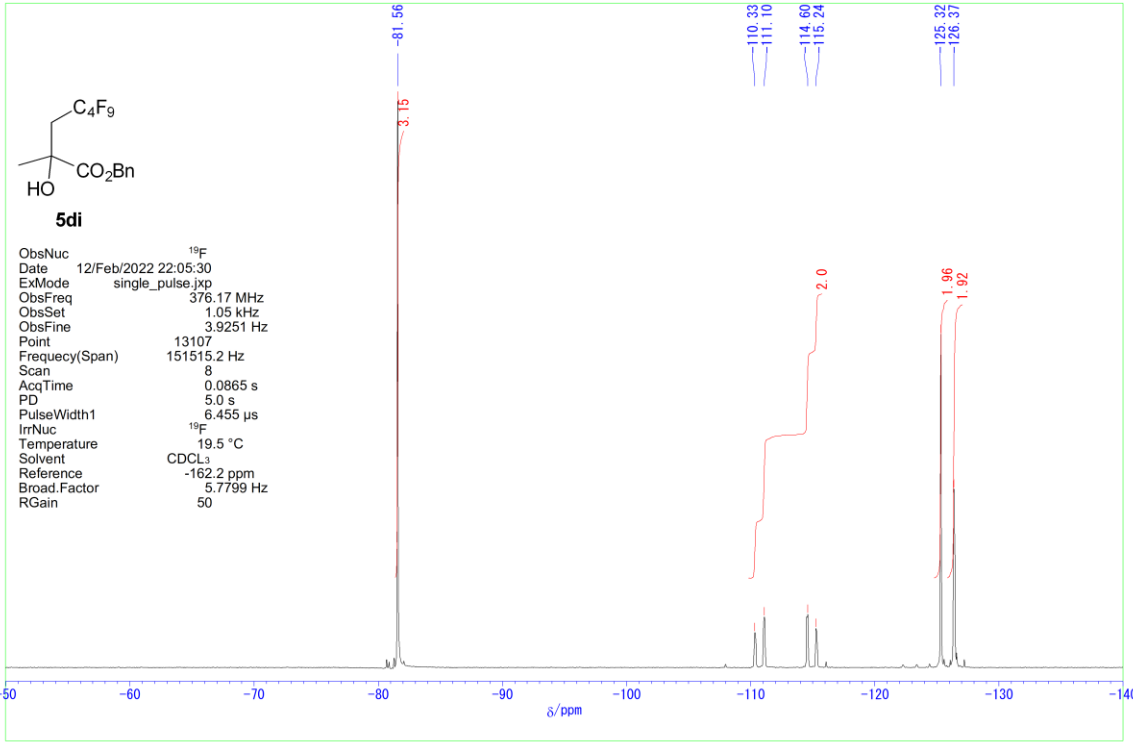

5dj: ^{1}H NMR ($CDCl_3$, 400 MHz)

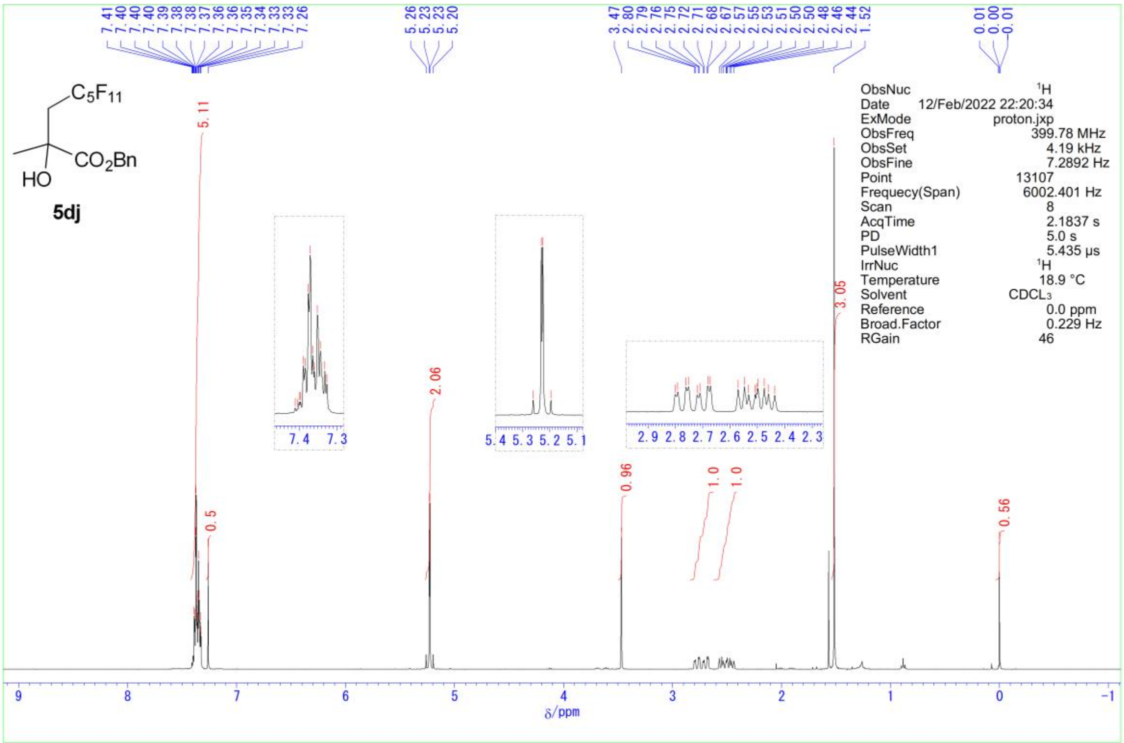

5dj: ^{13}C NMR ($CDCl_3$, 151 MHz)

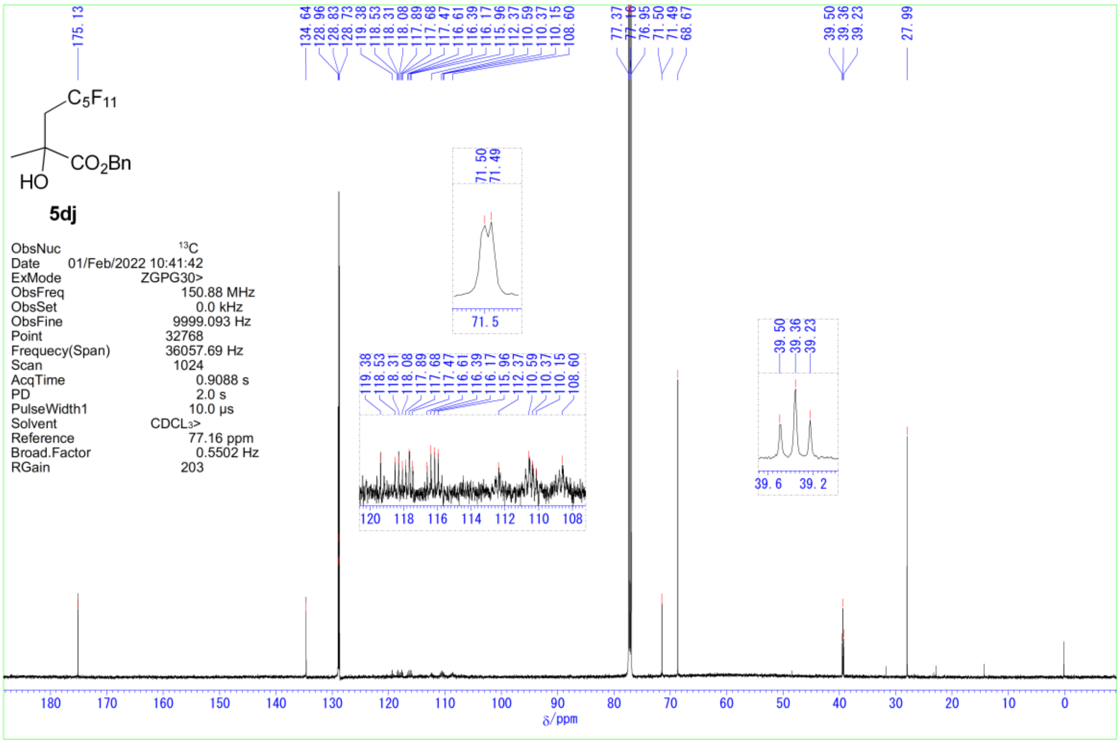

5dj: ^{19}F NMR ($CDCl_3$, 376 MHz)

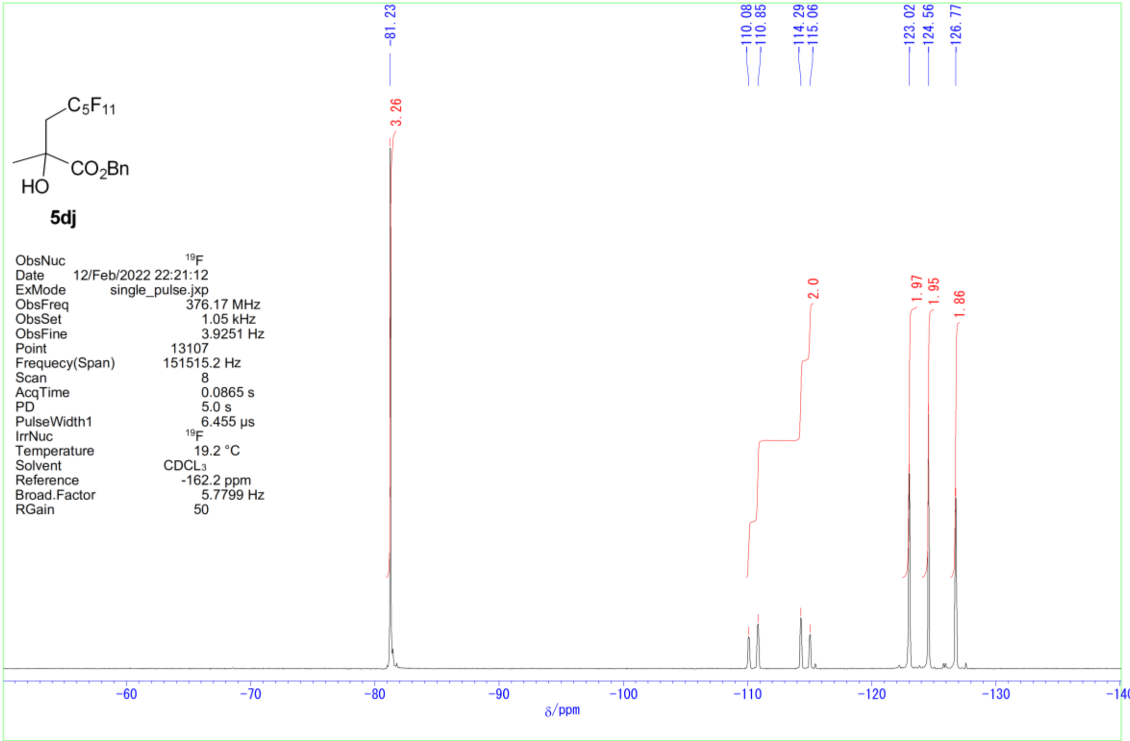

5dk: ^{1}H NMR ($CDCl_3$, 400 MHz)

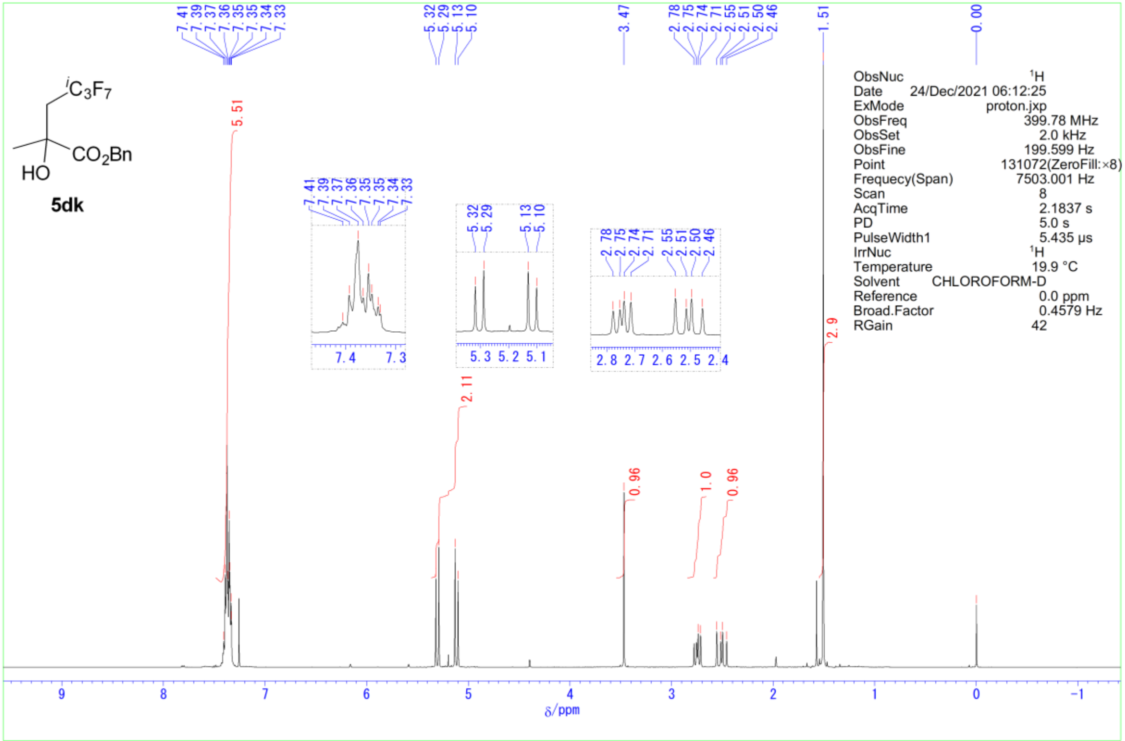

5dk: ^{13}C NMR ($CDCl_3$, 151 MHz)

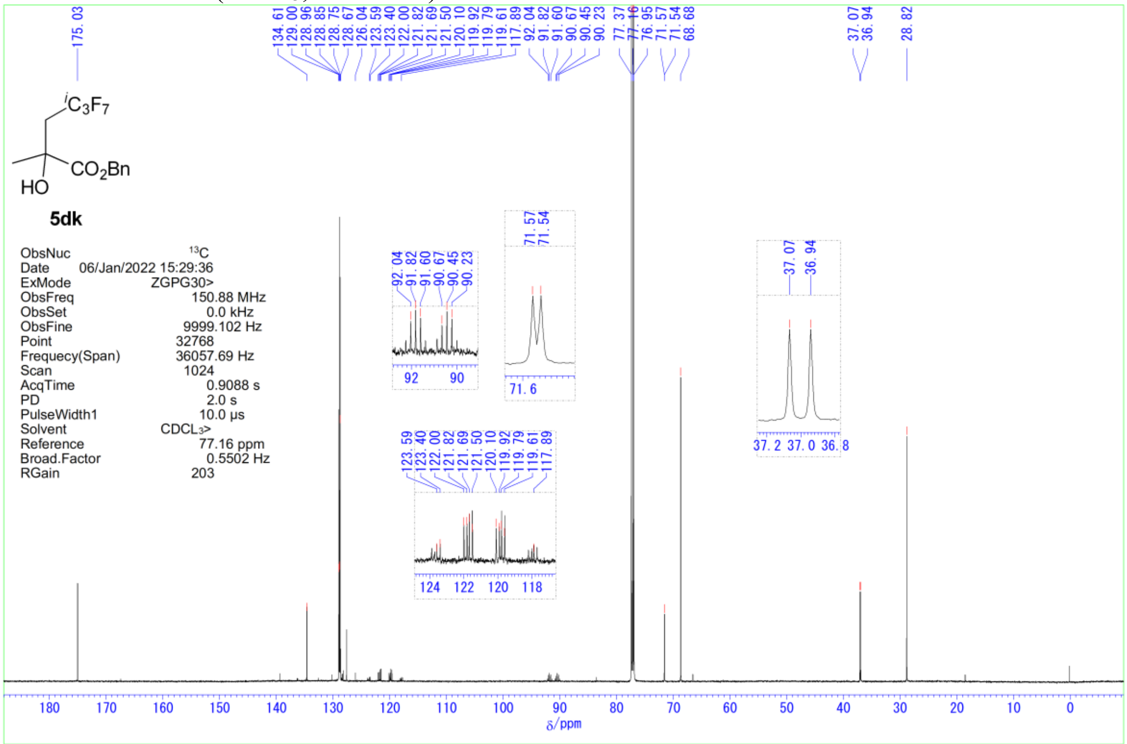

5dk: ^{19}F NMR ($CDCl_3$, 376 MHz)

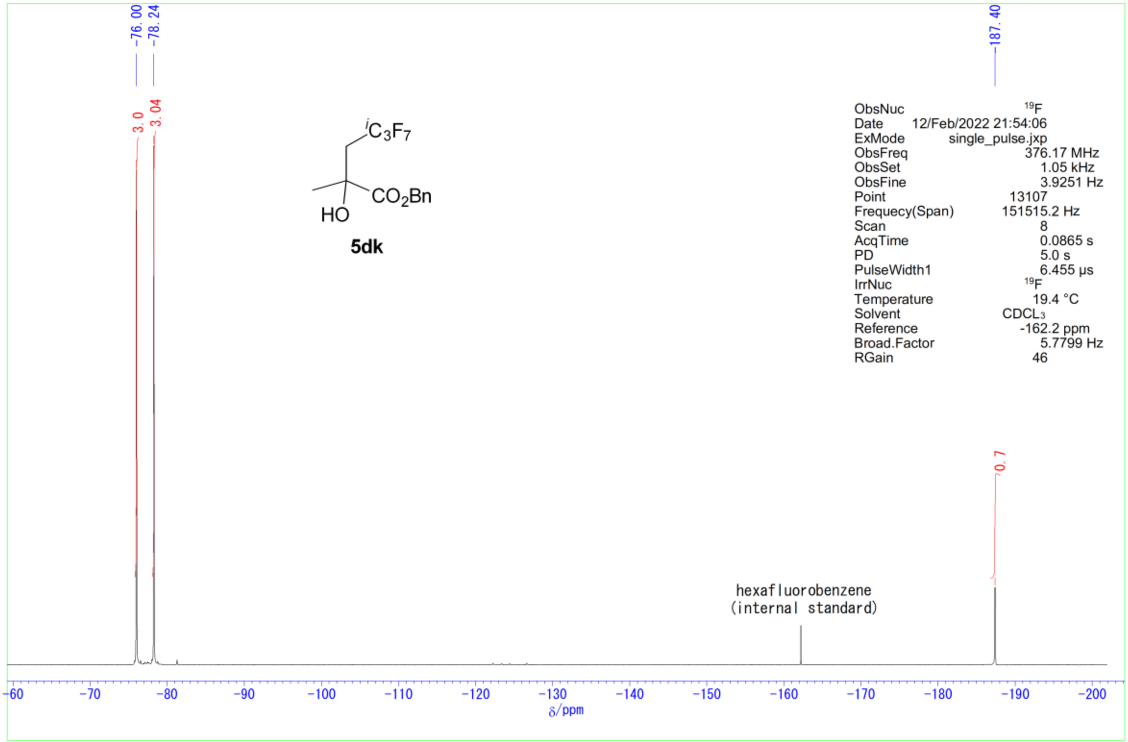

5dl: ^{1}H NMR ($CDCl_3$, 400 MHz)

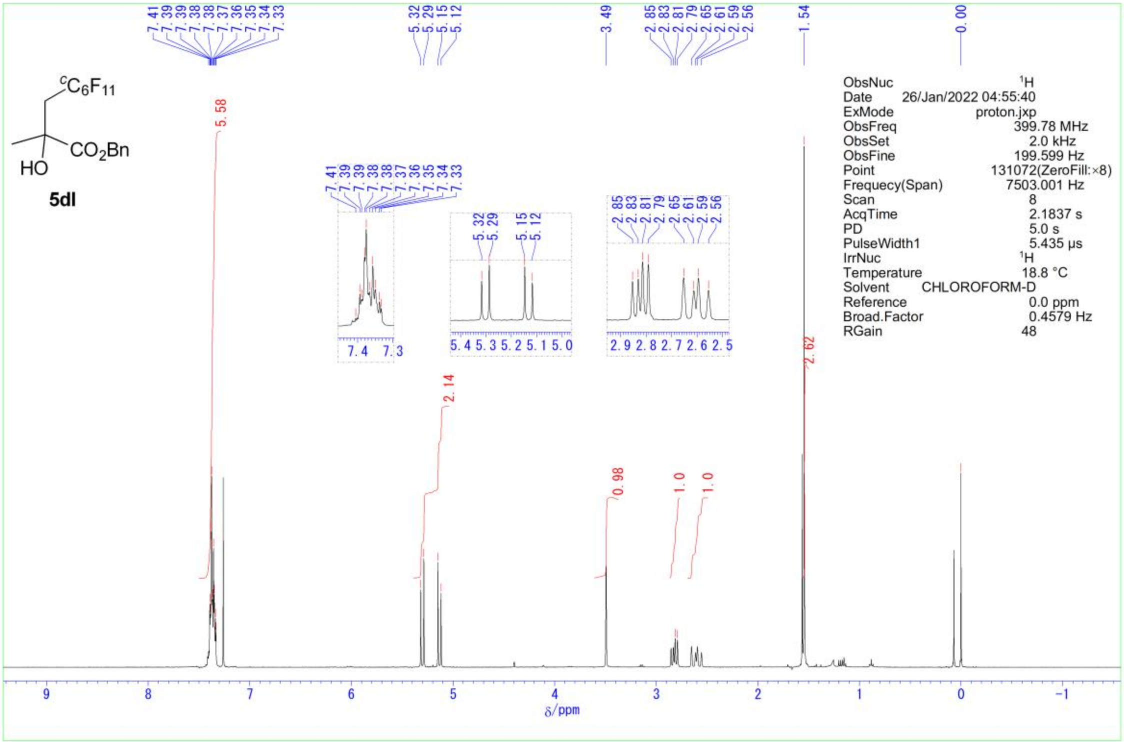

5dl: ^{13}C NMR ($CDCl_3$, 151 MHz)

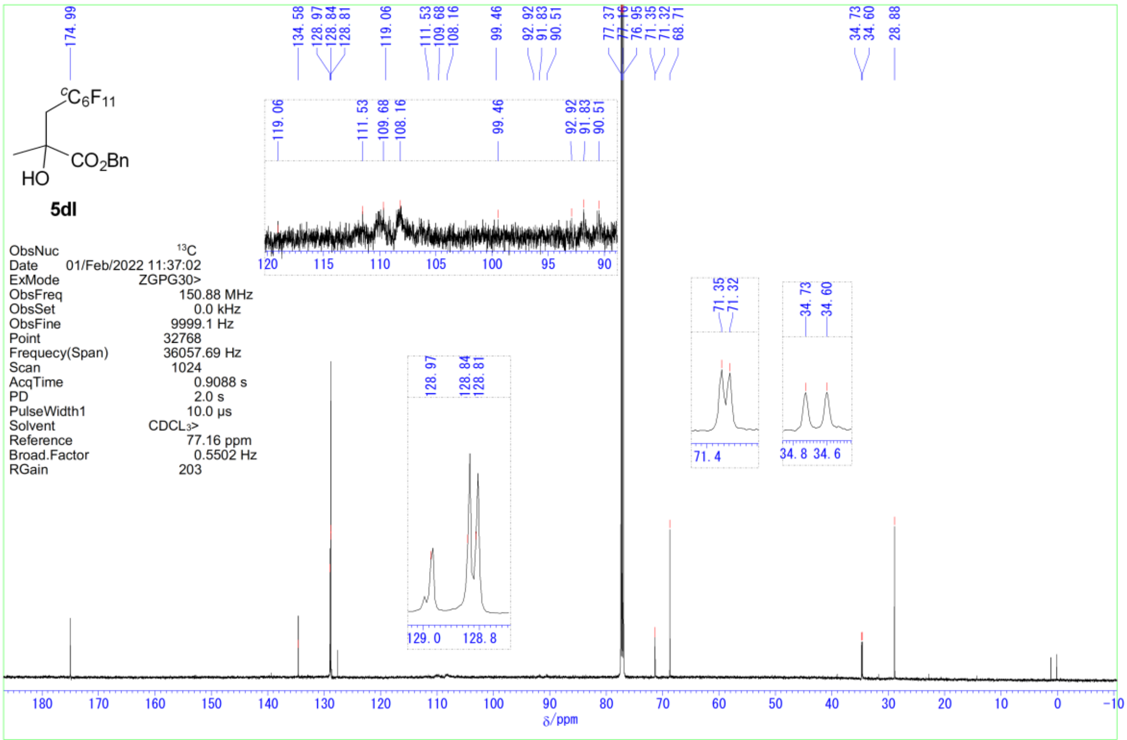

5dl: ^{19}F NMR ($CDCl_3$, 376 MHz)

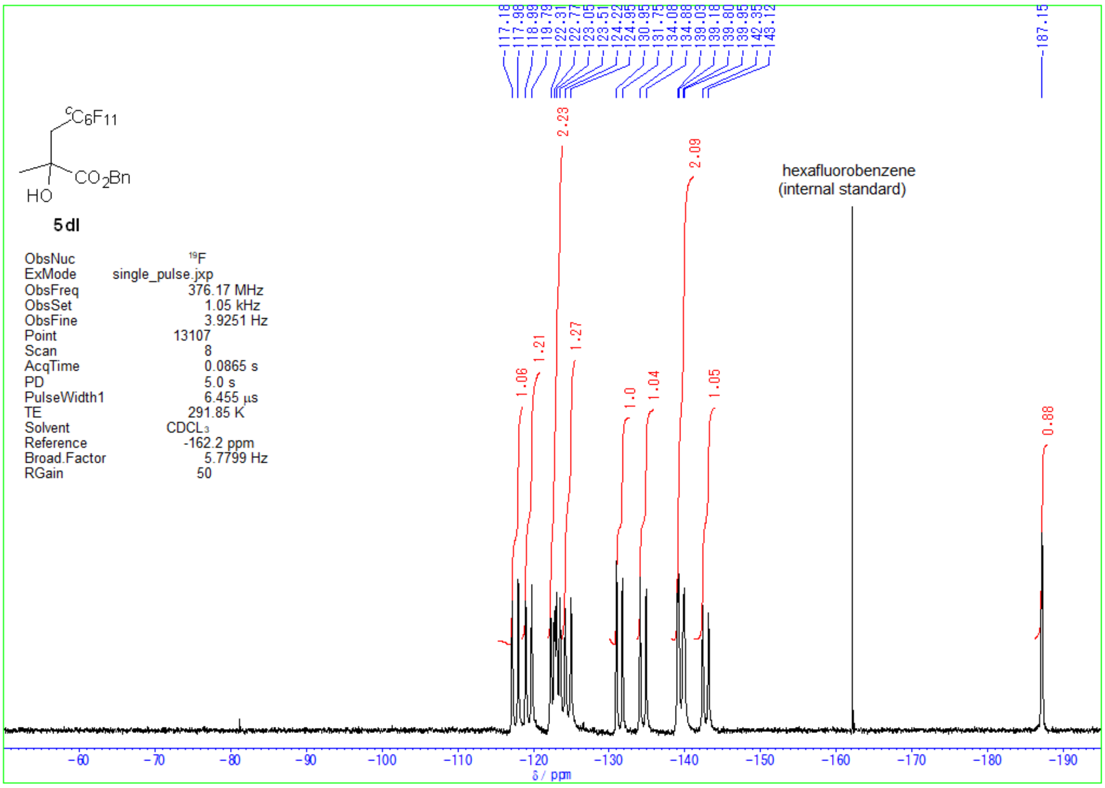

5dm: ^{1}H NMR ($CDCl_3$, 400 MHz)

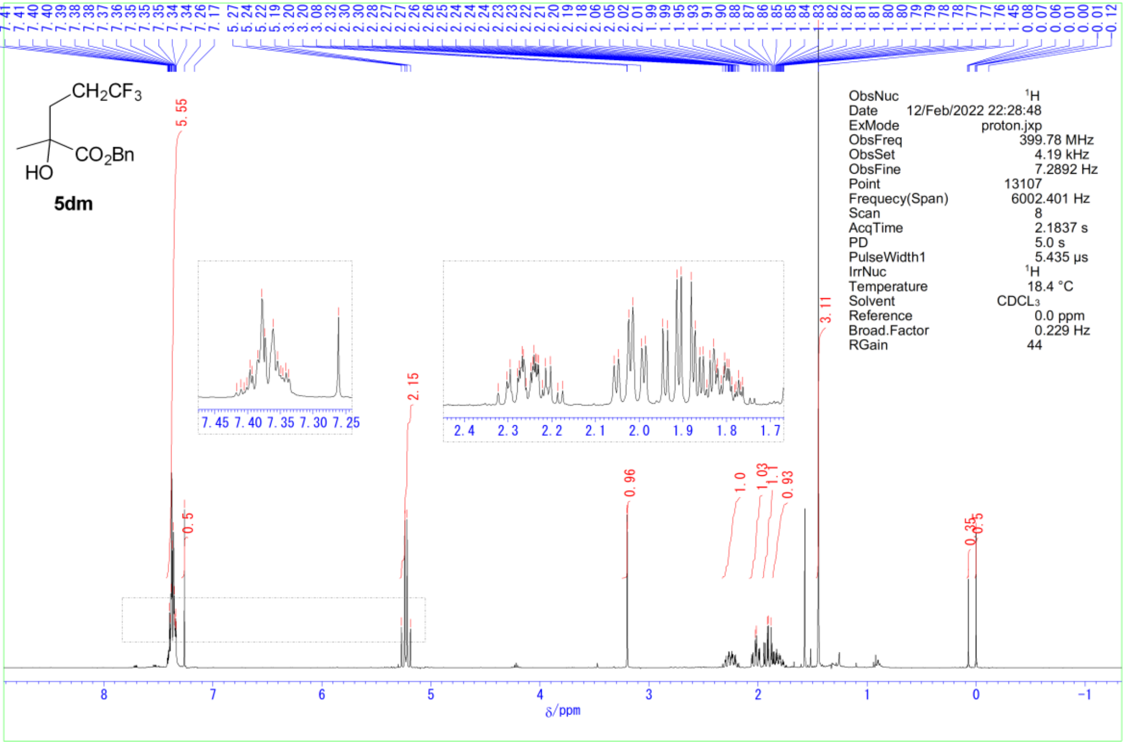

5dm: ^{13}C NMR ($CDCl_3$, 151 MHz)

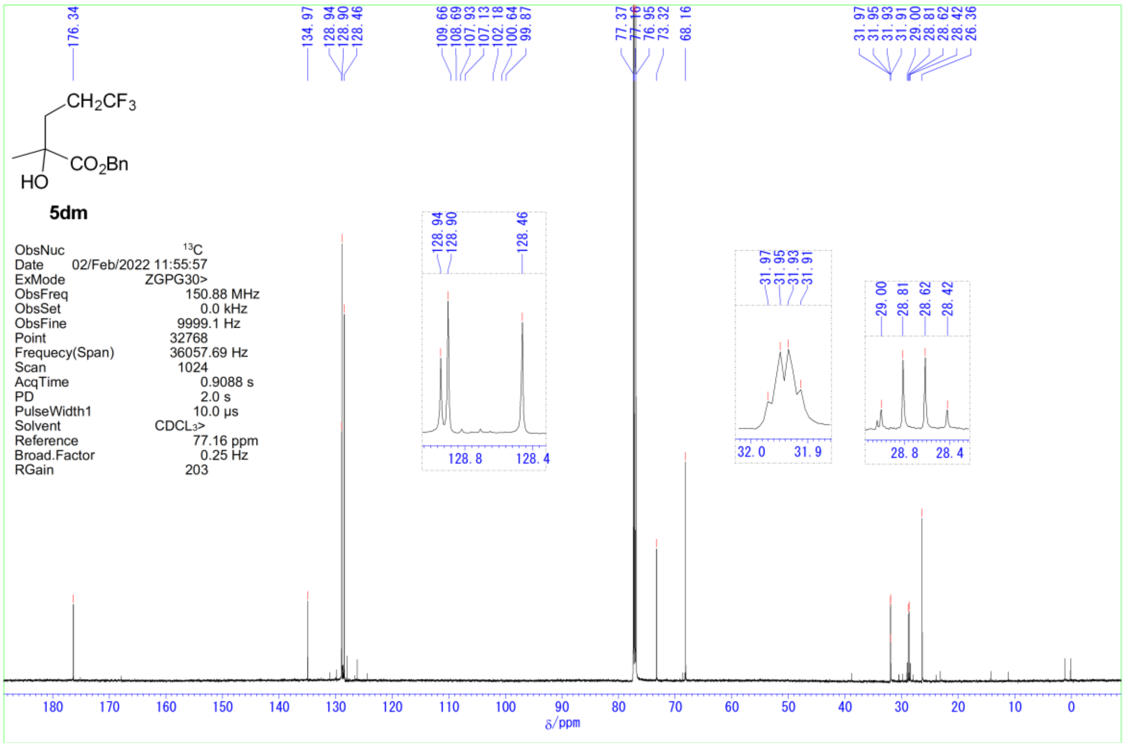

5dm: ^{19}F NMR ($CDCl_3$, 376 MHz)

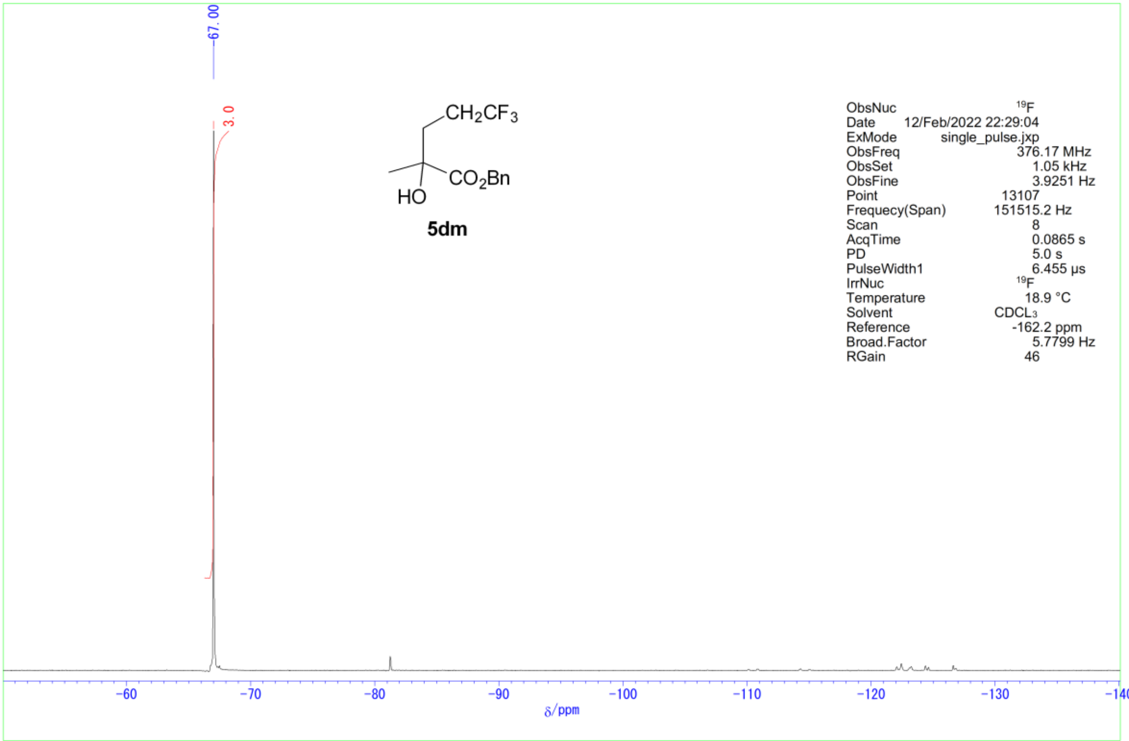

5dn: ^{1}H NMR ($CDCl_3$, 400 MHz)

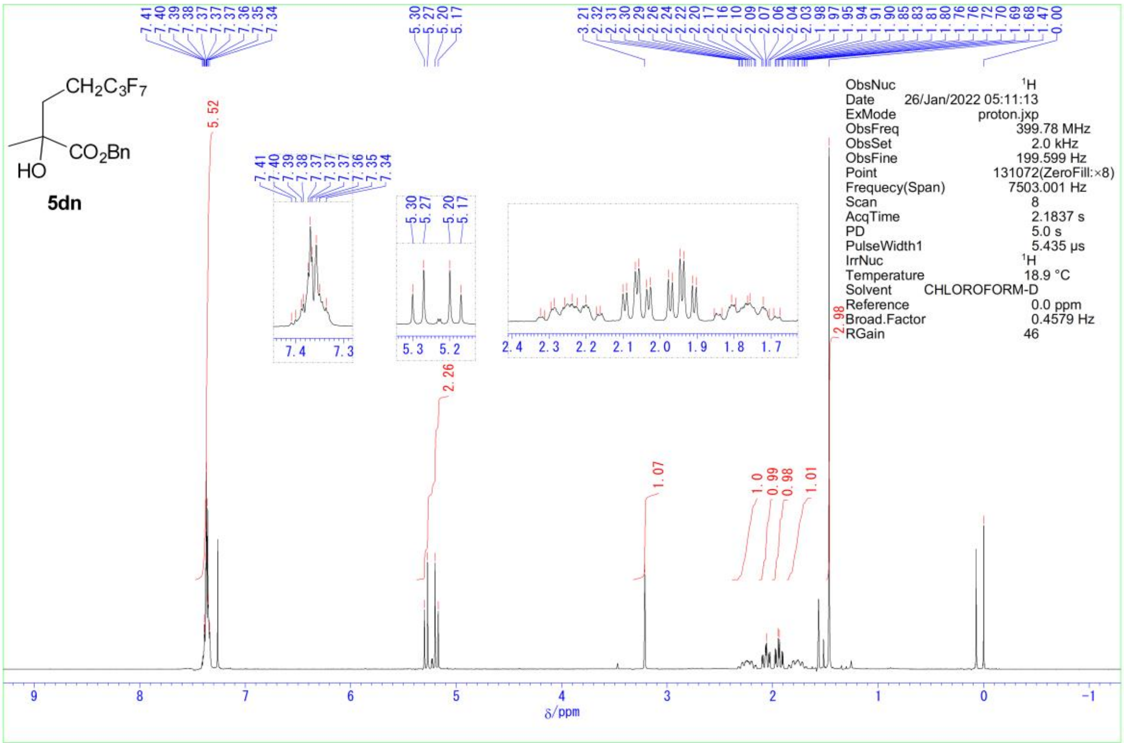

5dn: ^{13}C NMR ($CDCl_3$, 151 MHz)

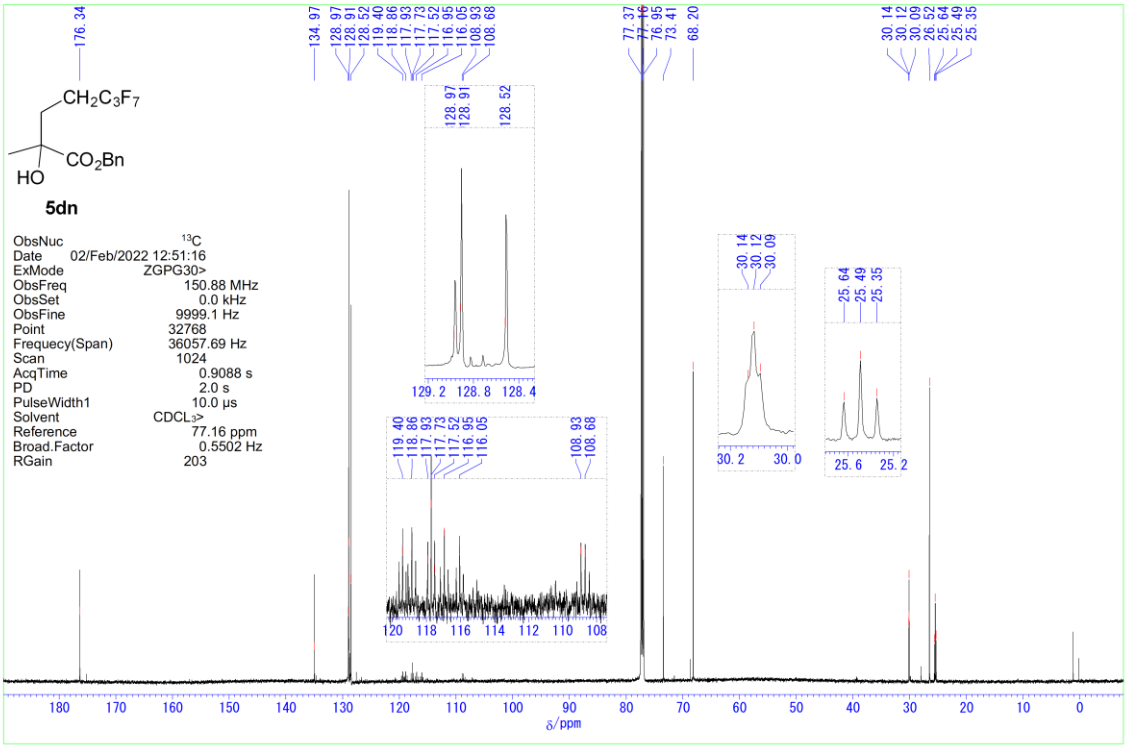

5dn: ^{19}F NMR ($CDCl_3$, 376 MHz)

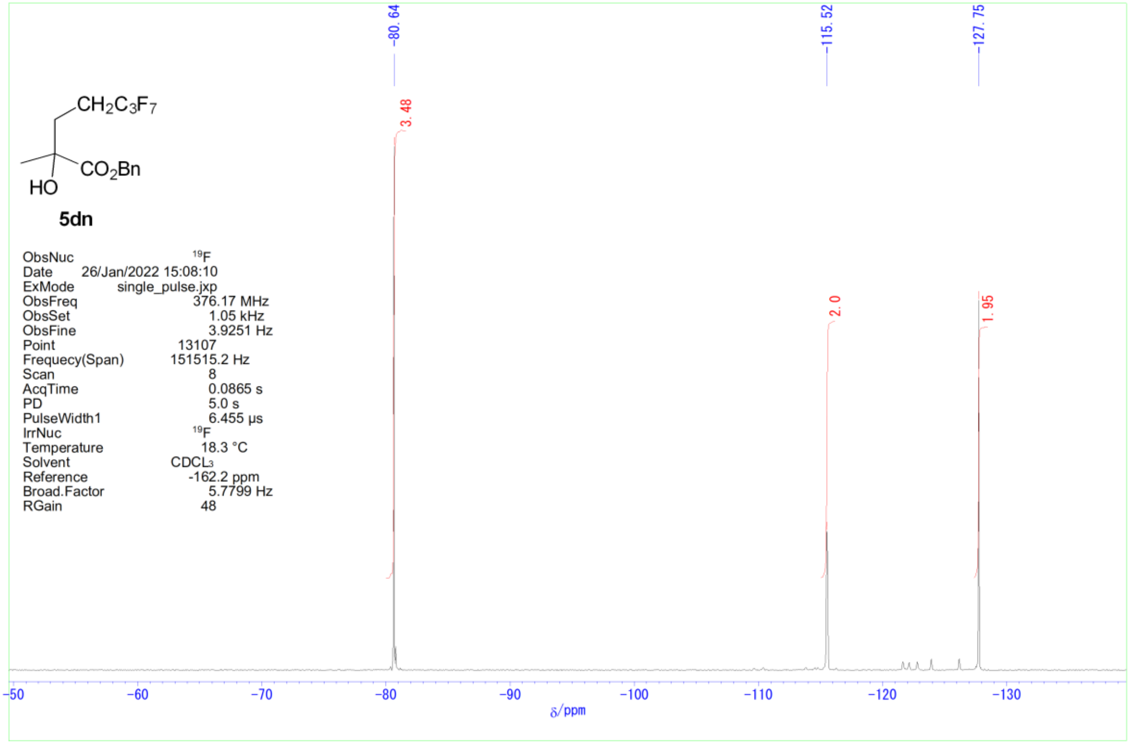

7aa: ^{1}H NMR ($CDCl_3$, 500 MHz)

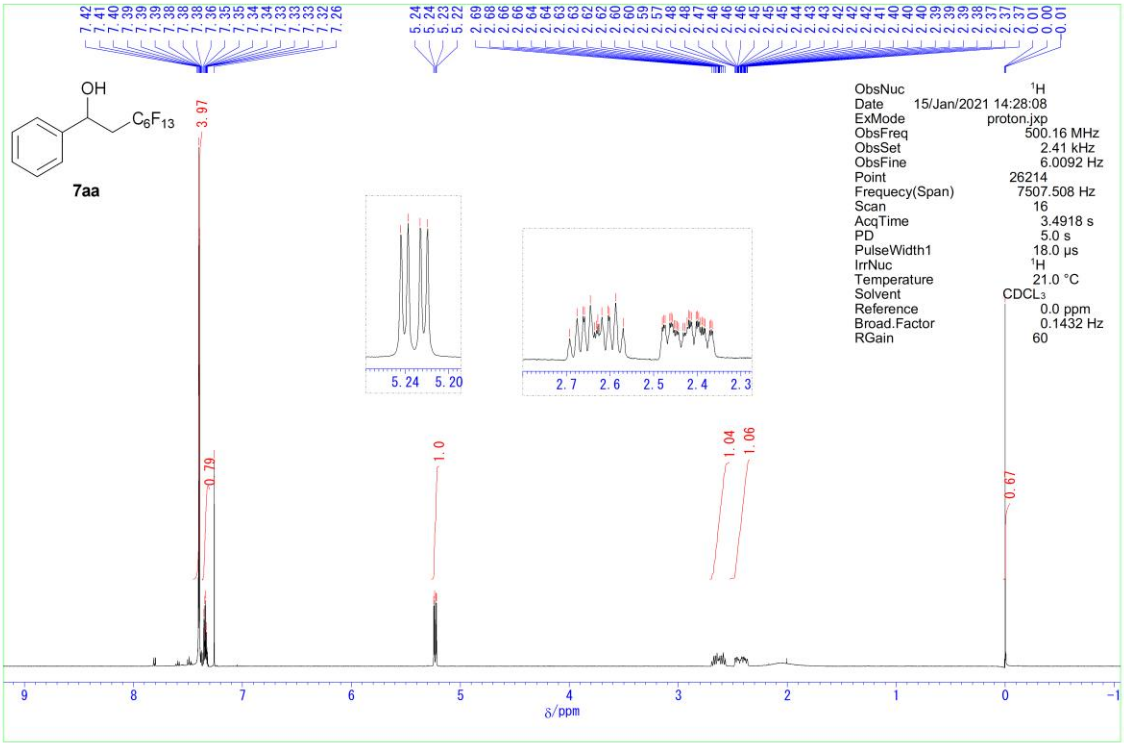

7aa: ^{13}C NMR ($CDCl_3$, 151 MHz)

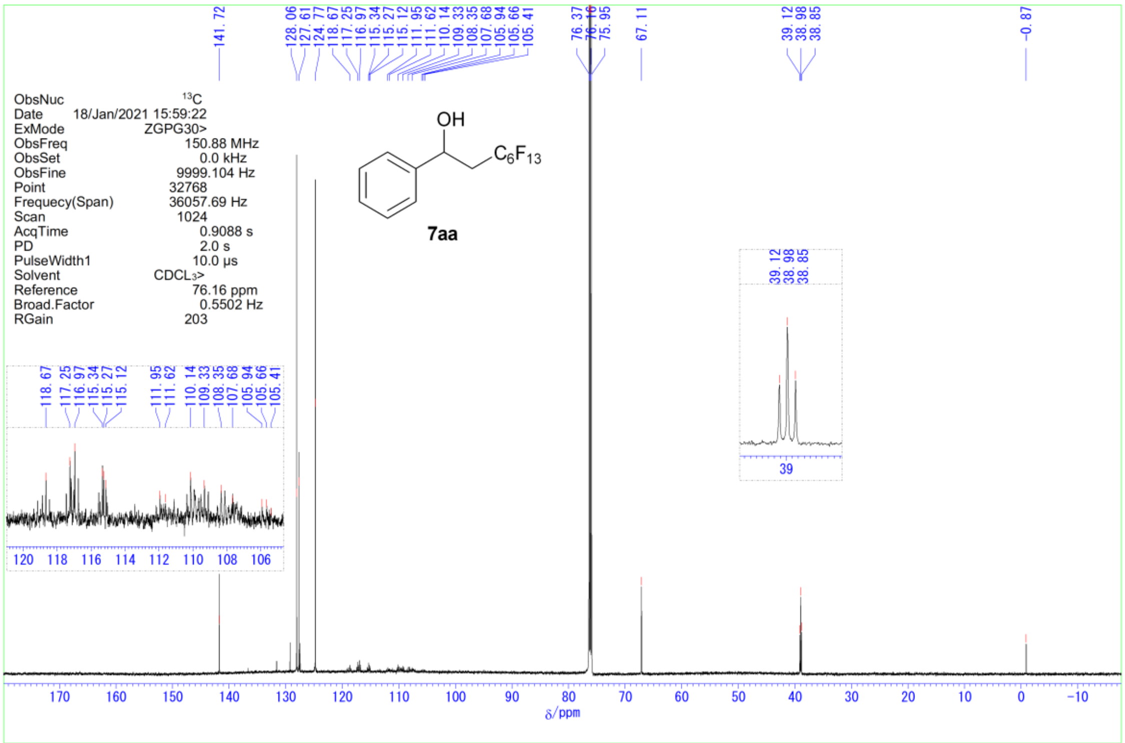

7aa: ^{19}F NMR ($CDCl_3$, 471 MHz)

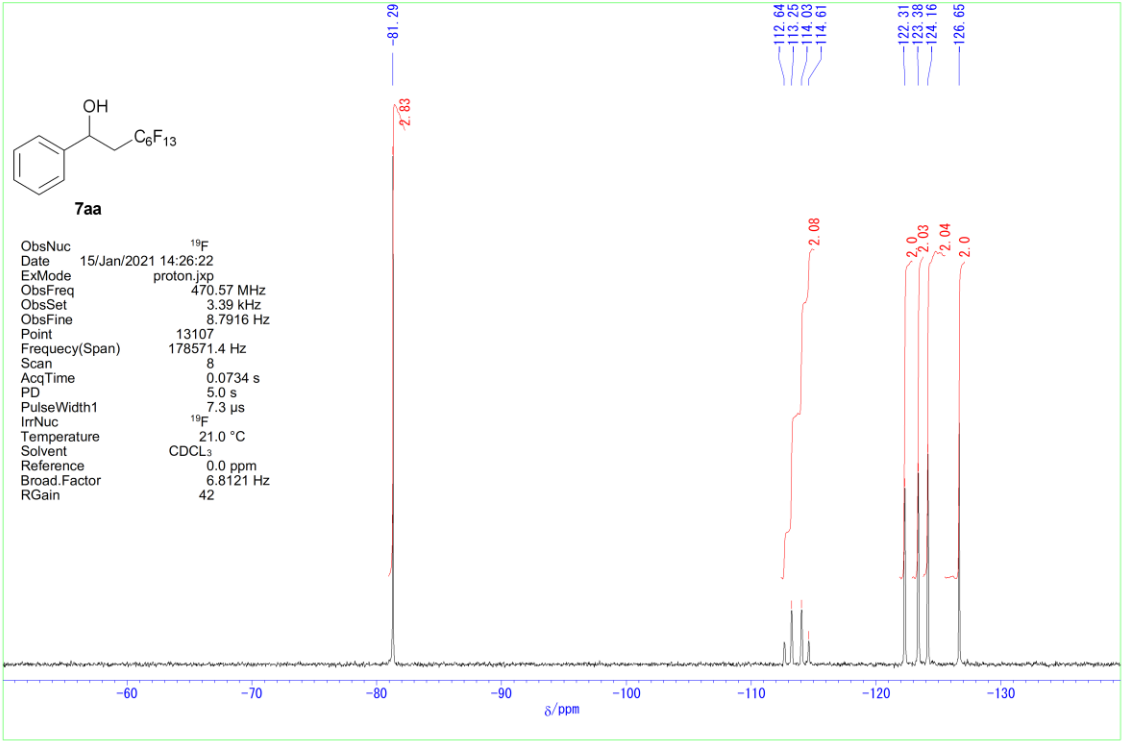

7ba: ^{1}H NMR ($CDCl_3$, 400 MHz)

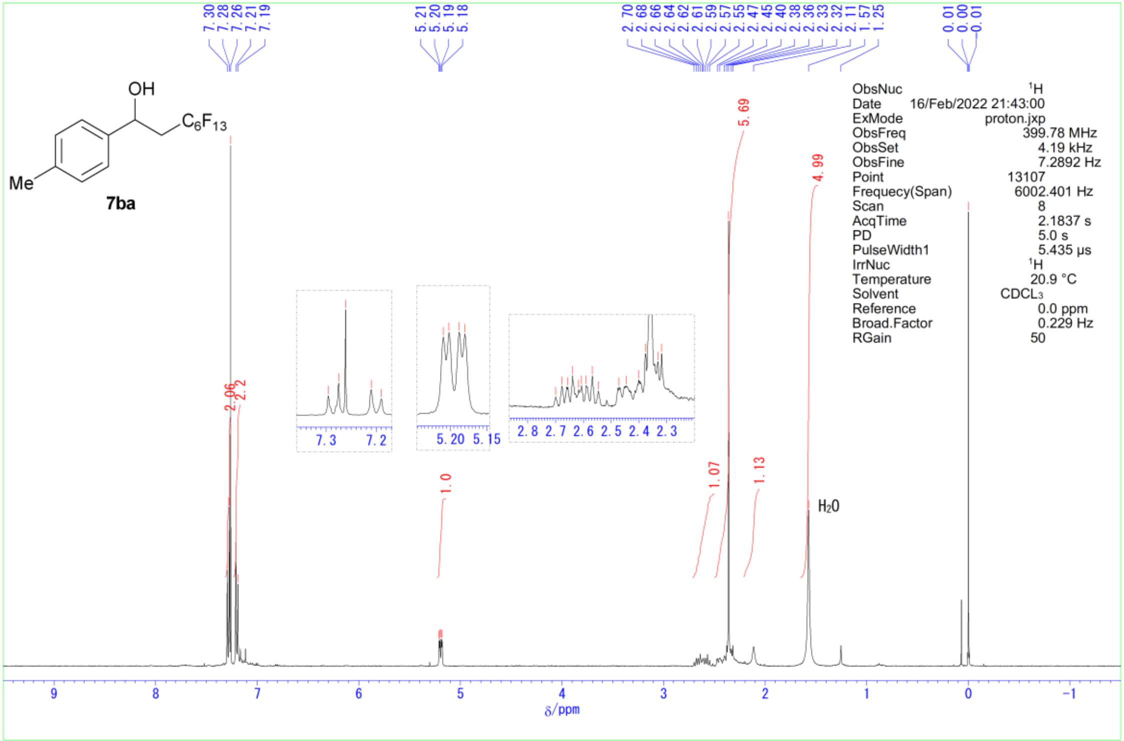

7ba: ^{13}C NMR ($CDCl_3$, 151 MHz)

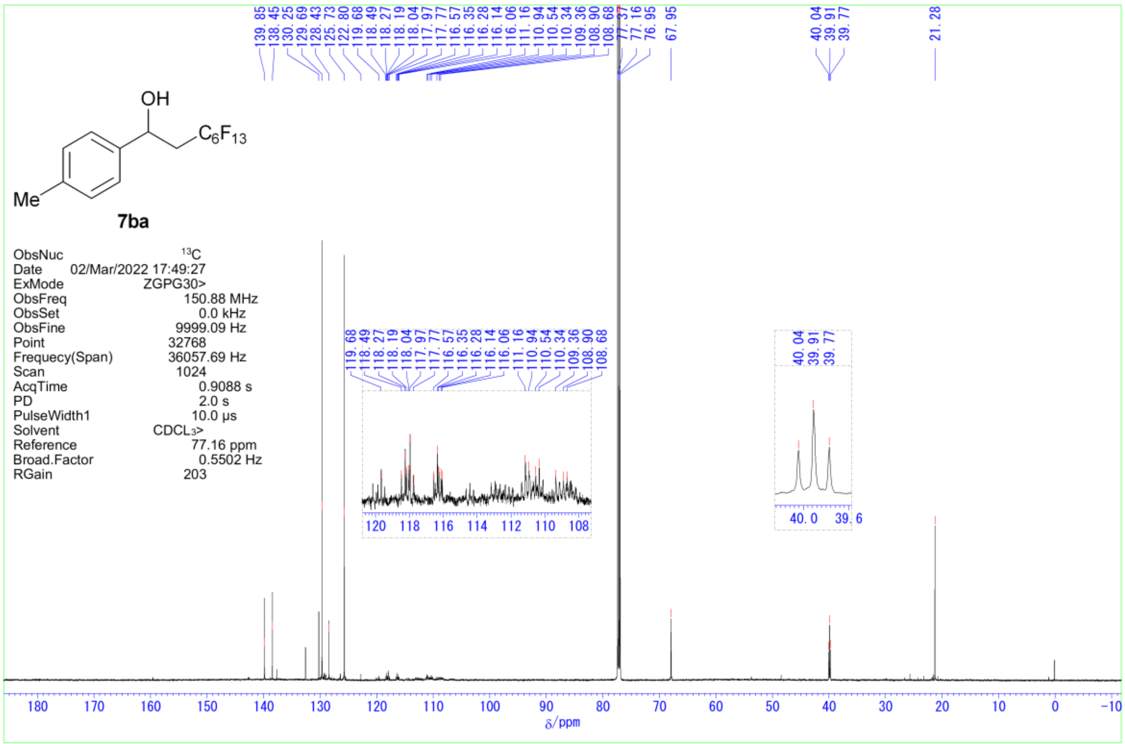

7ba: ^{19}F NMR ($CDCl_3$, 376 MHz)

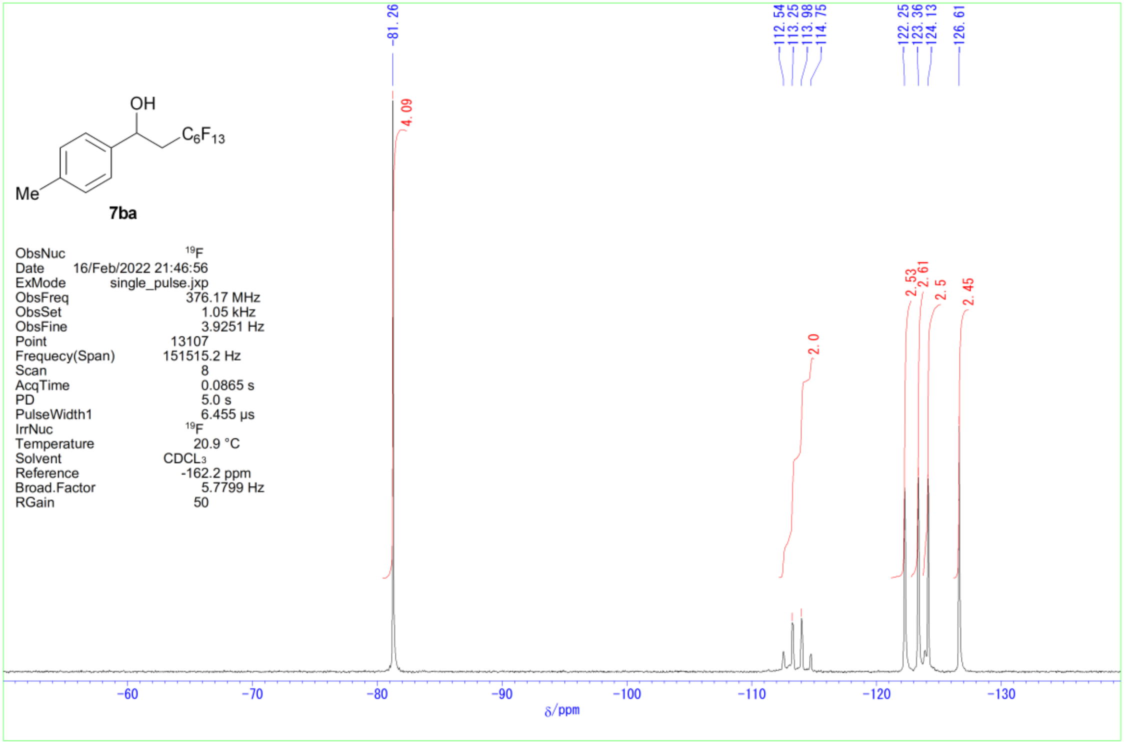

7ca: ^{1}H NMR ($CDCl_3$, 400 MHz)

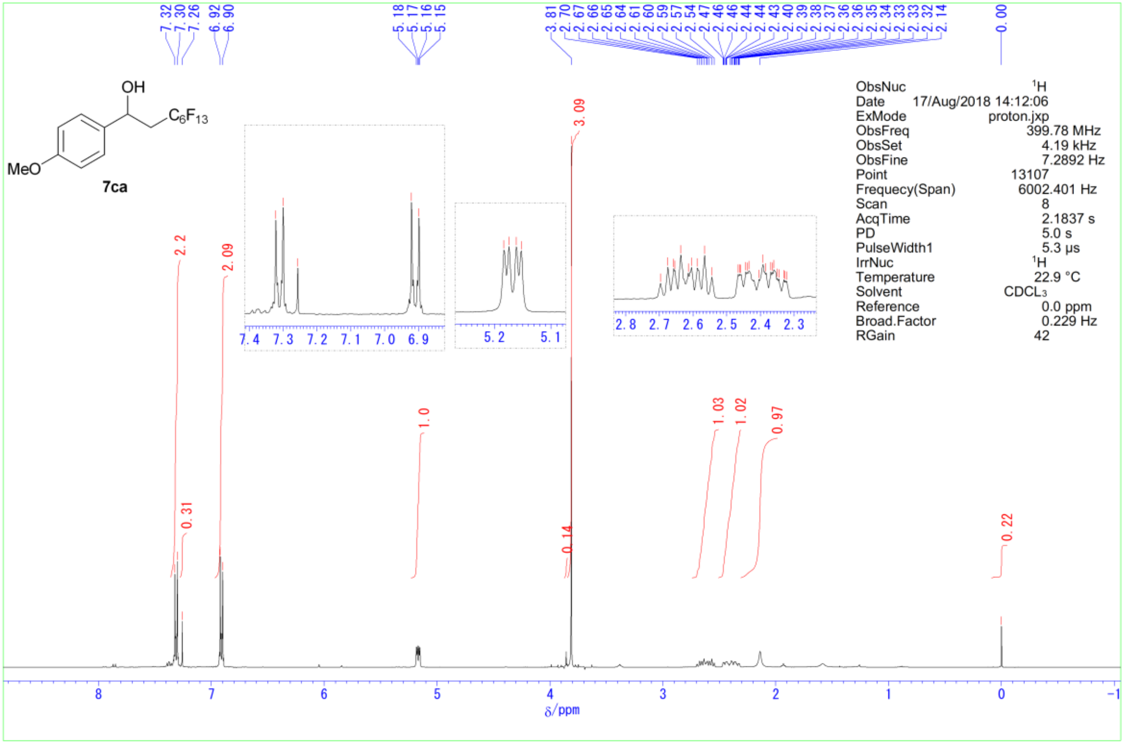

7ca: ^{13}C NMR ($CDCl_3$, 126 MHz)

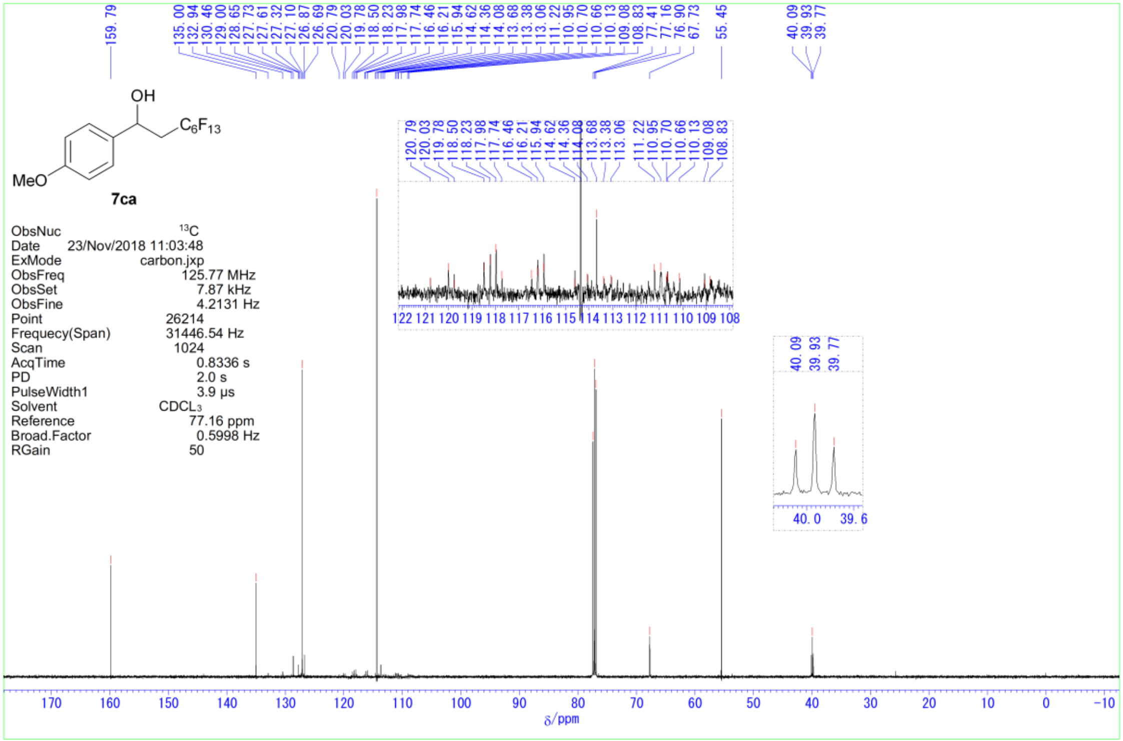

7ca: ^{19}F NMR ($CDCl_3$, 376 MHz)

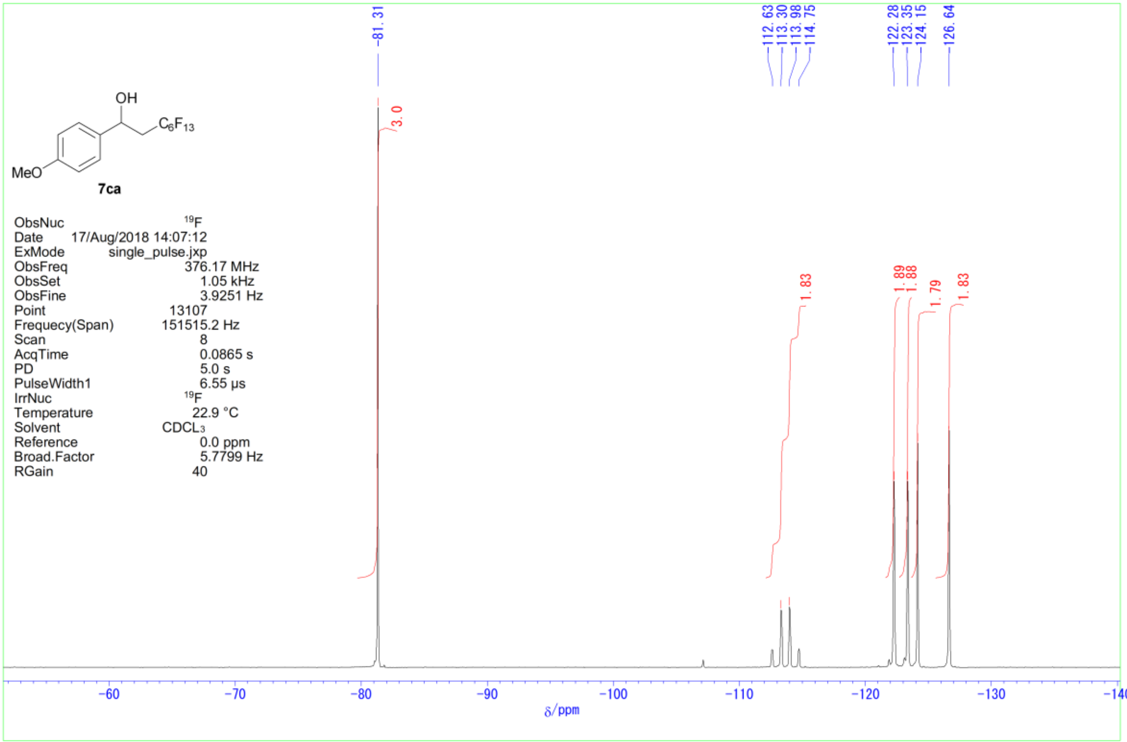

7da: ^{1}H NMR ($CDCl_3$, 500 MHz)

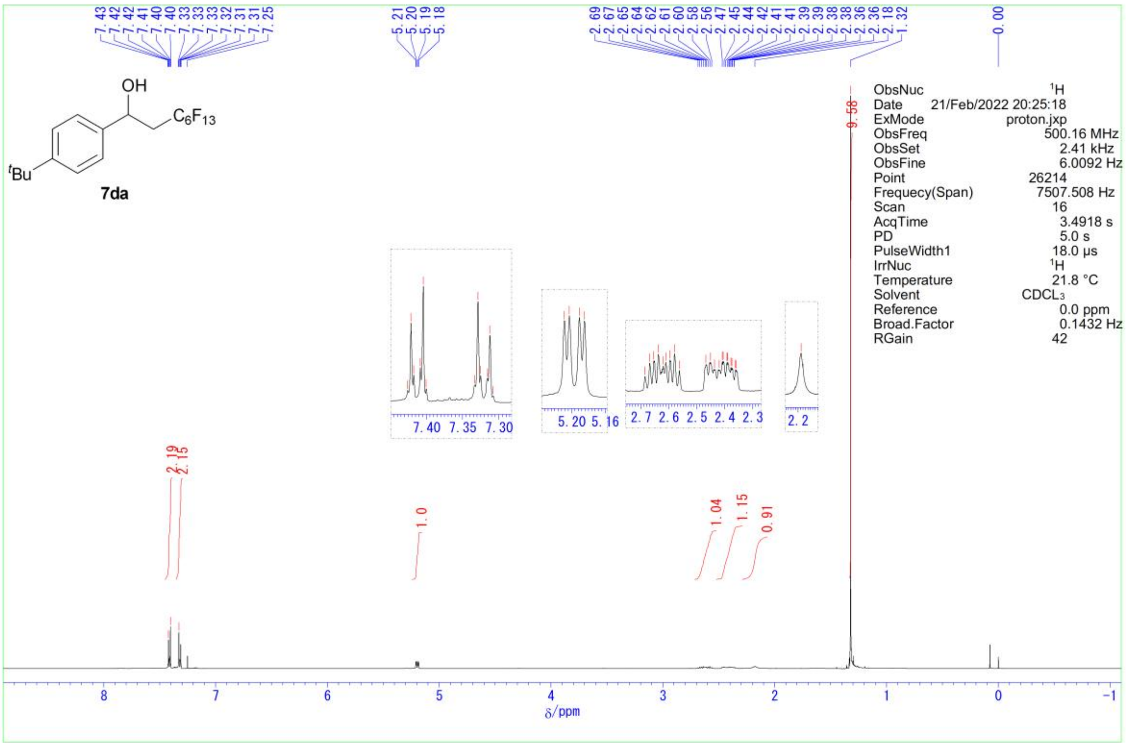

7da: ^{13}C NMR ($CDCl_3$, 151 MHz)

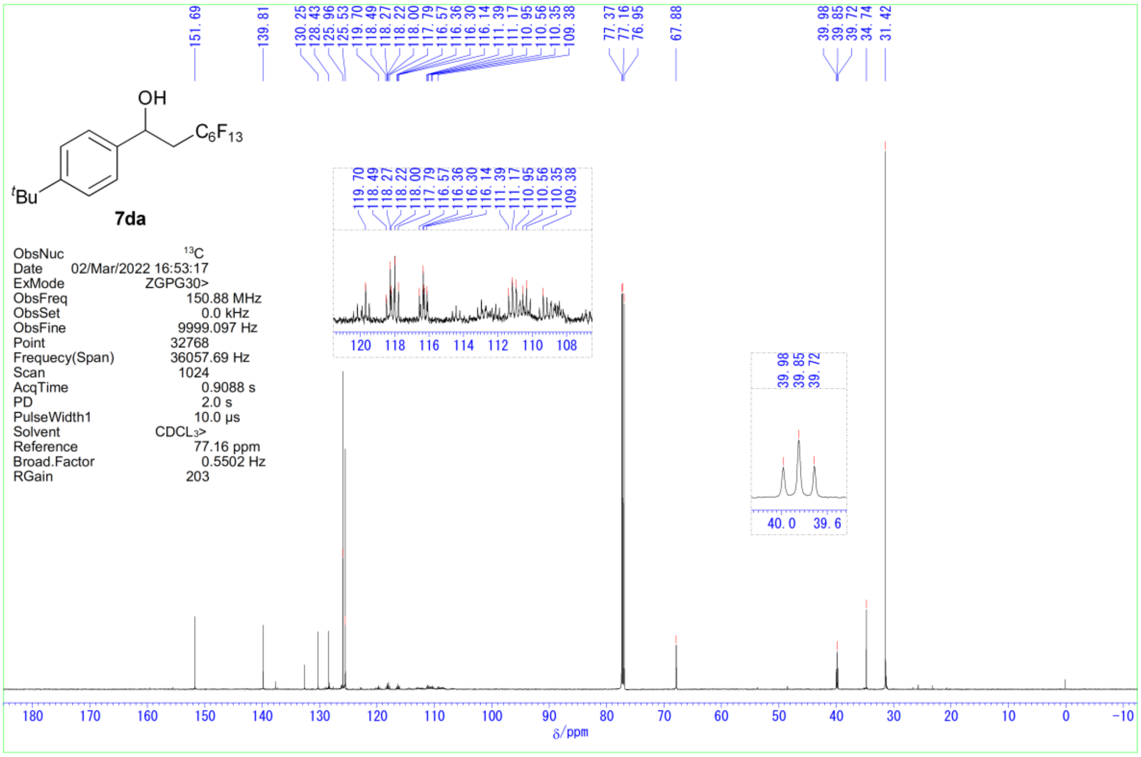

7da: ^{19}F NMR ($CDCl_3$, 471 MHz)

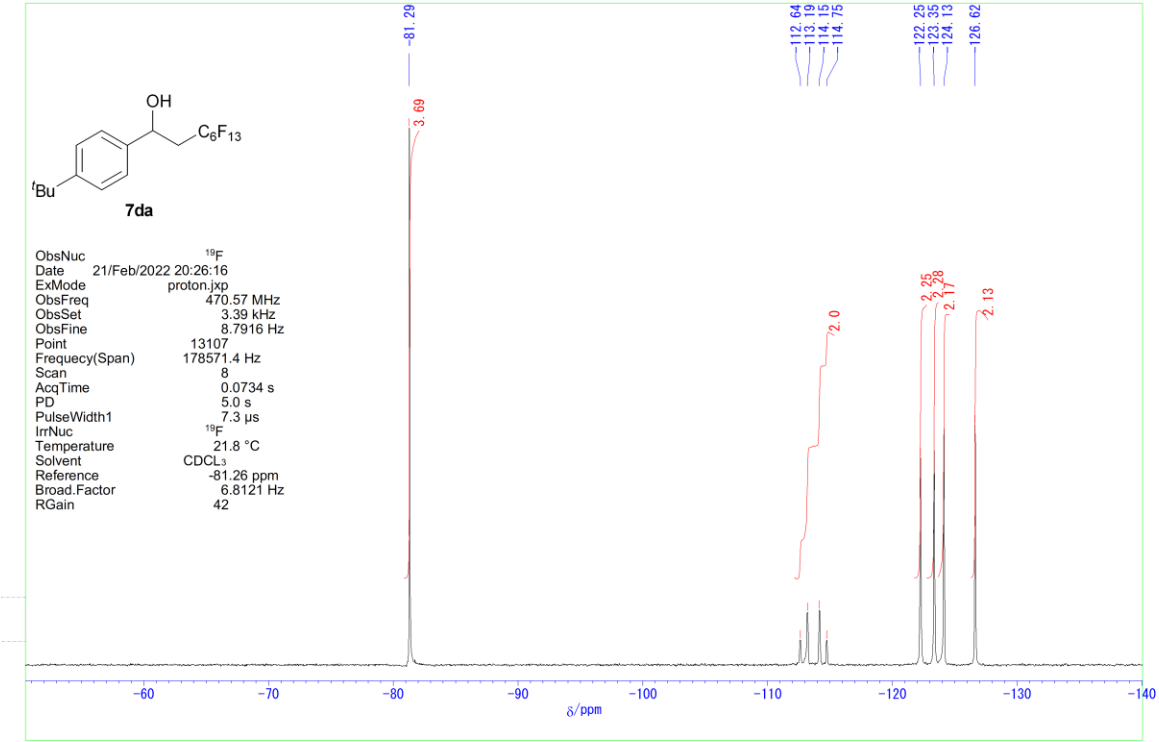

7ea: ^{1}H NMR ($CDCl_3$, 400 MHz)

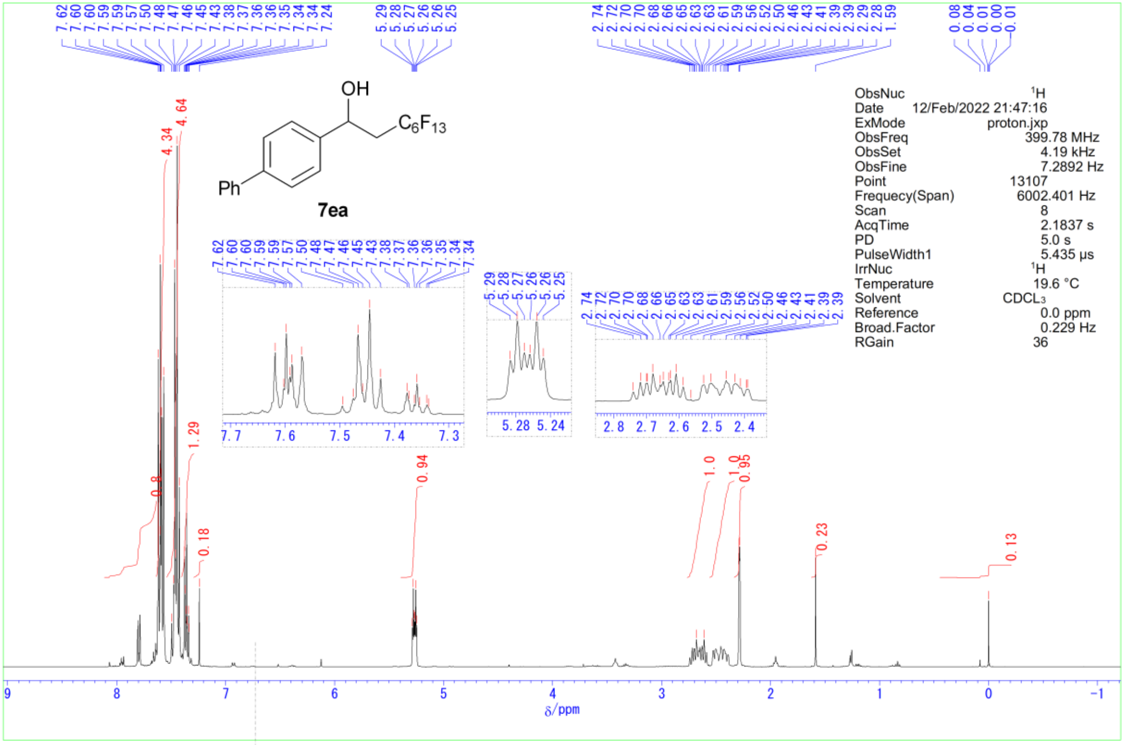

7ea: ^{13}C NMR ($CDCl_3$, 151 MHz)

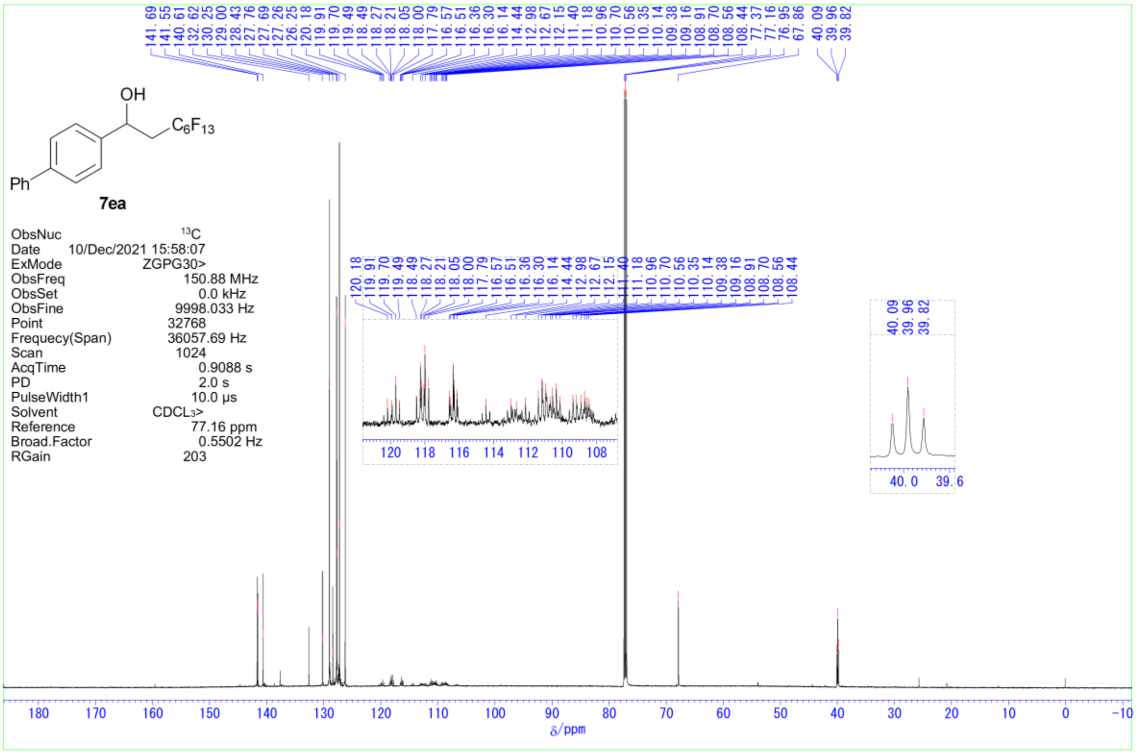

7ea: ^{19}F NMR ($CDCl_3$, 376 MHz)

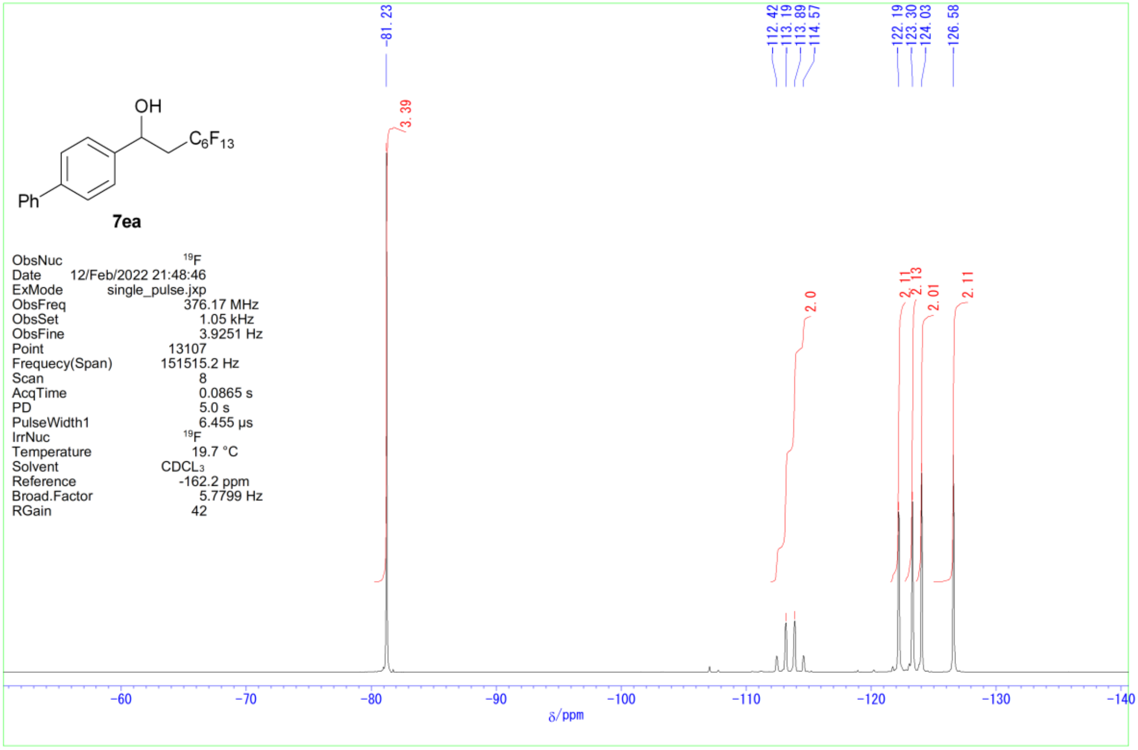

7fa: ^{1}H NMR ($CDCl_3$, 500 MHz)

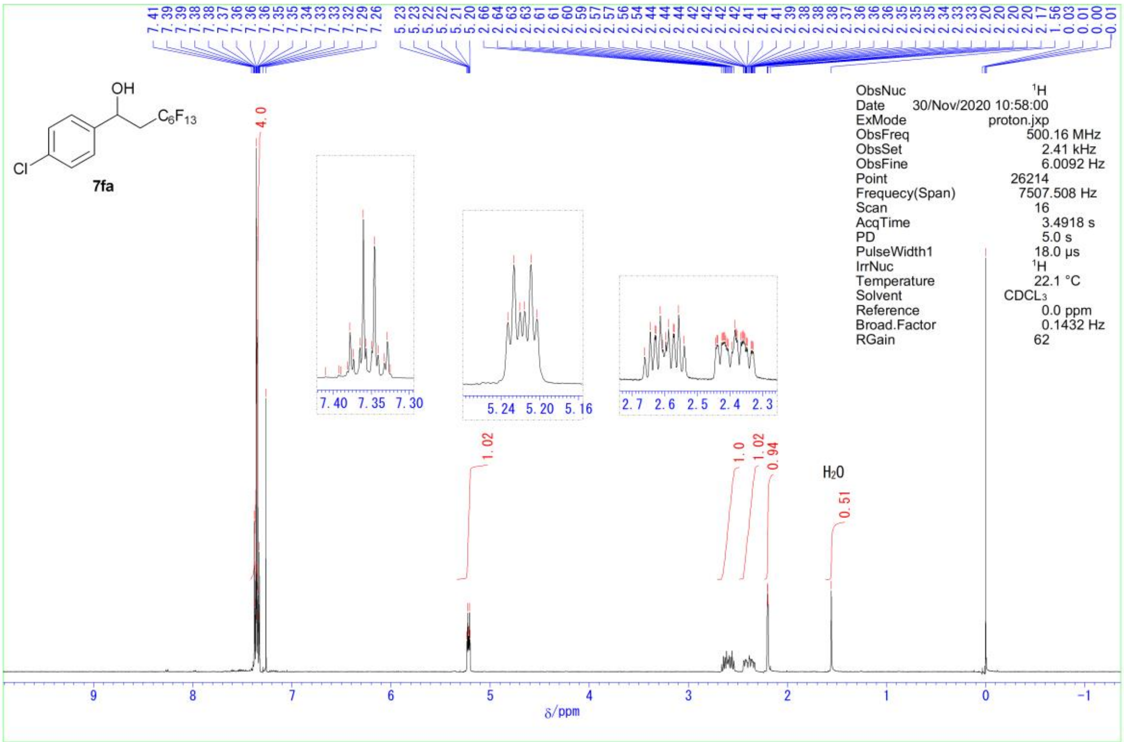

7fa: ^{13}C NMR ($CDCl_3$, 151 MHz)

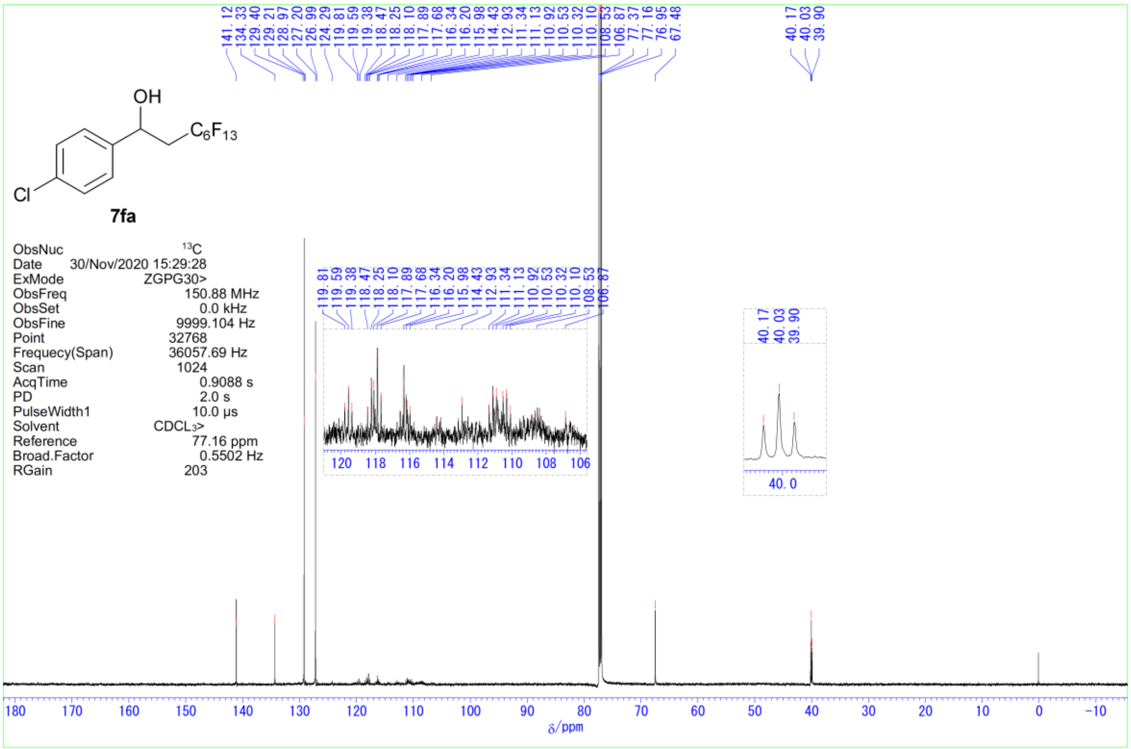

7fa: ^{19}F NMR ($CDCl_3$, 471 MHz)

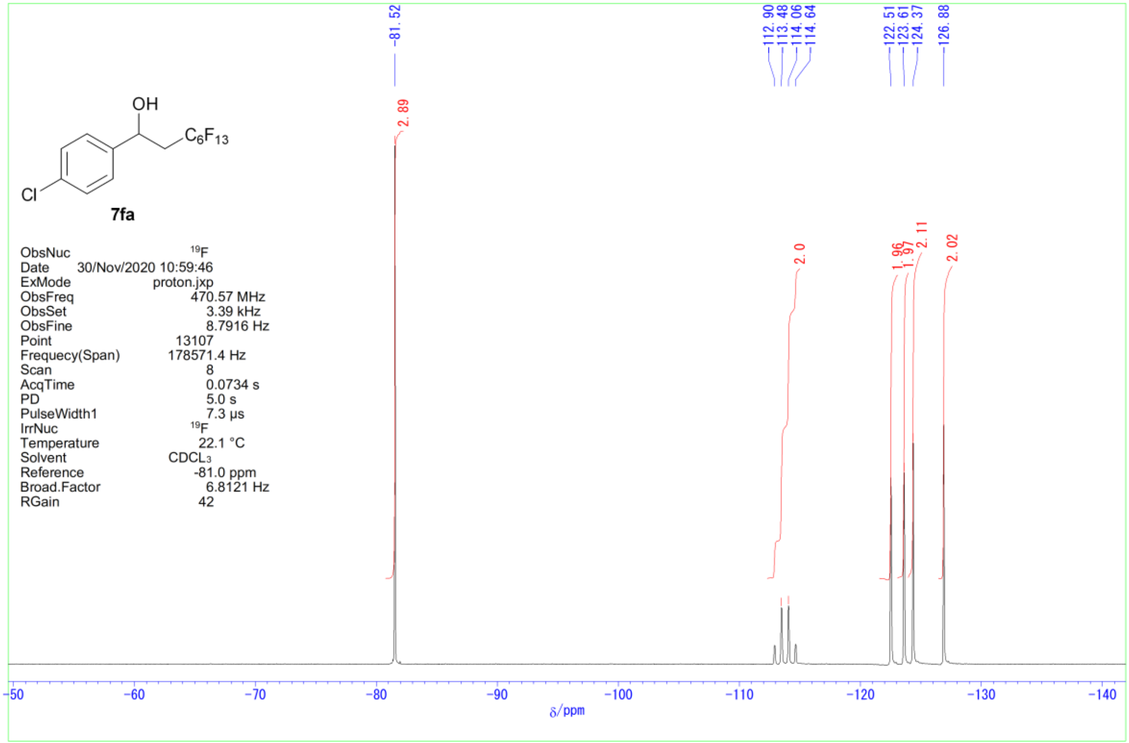

7ga: ^{1}H NMR ($CDCl_3$, 500 MHz)

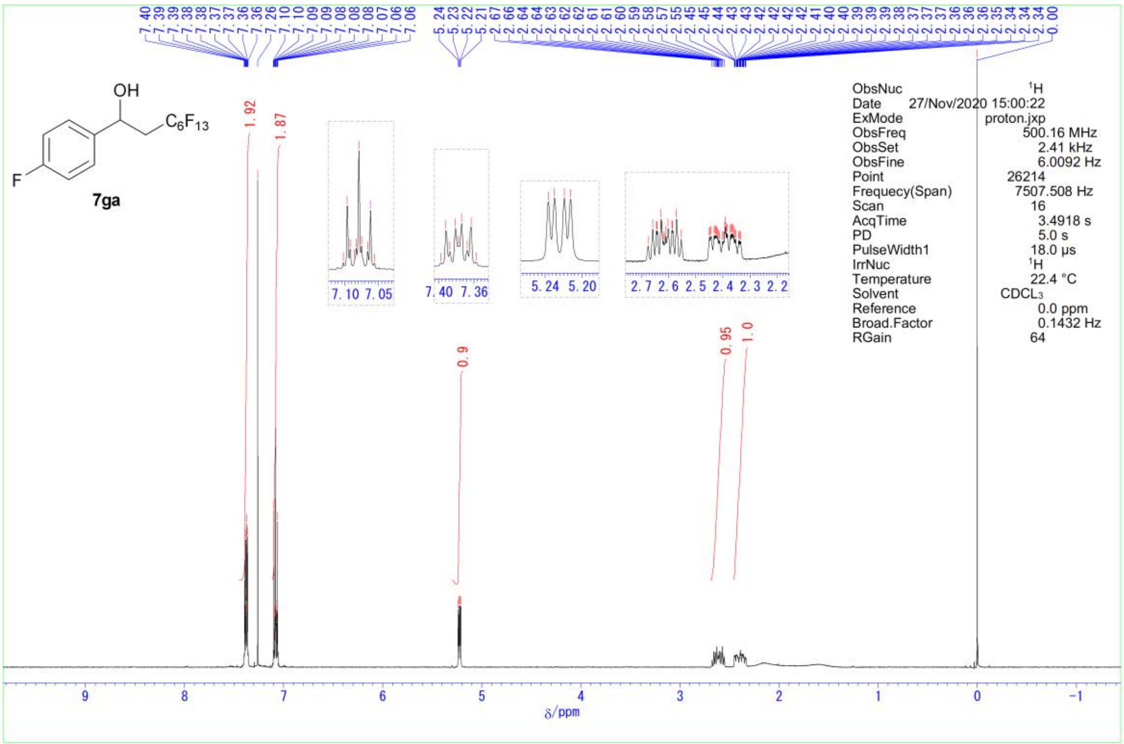

7ga: ^{13}C NMR ($CDCl_3$, 151 MHz)

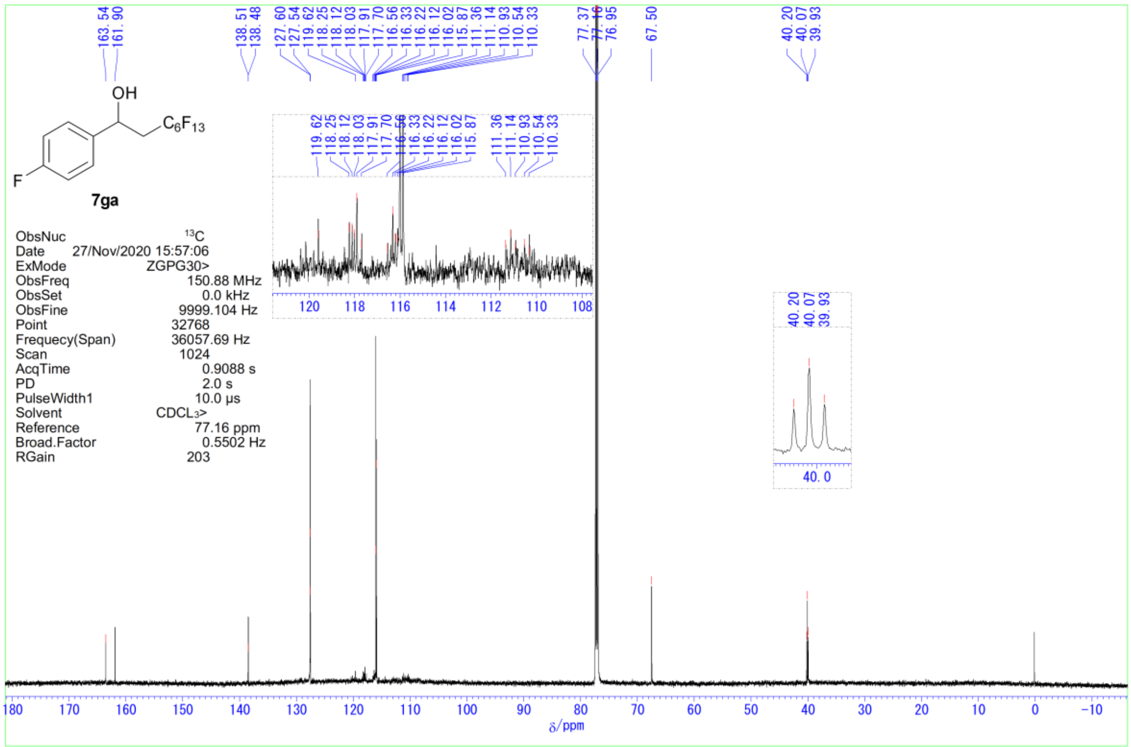

7ga: ^{19}F NMR ($CDCl_3$, 471 MHz)

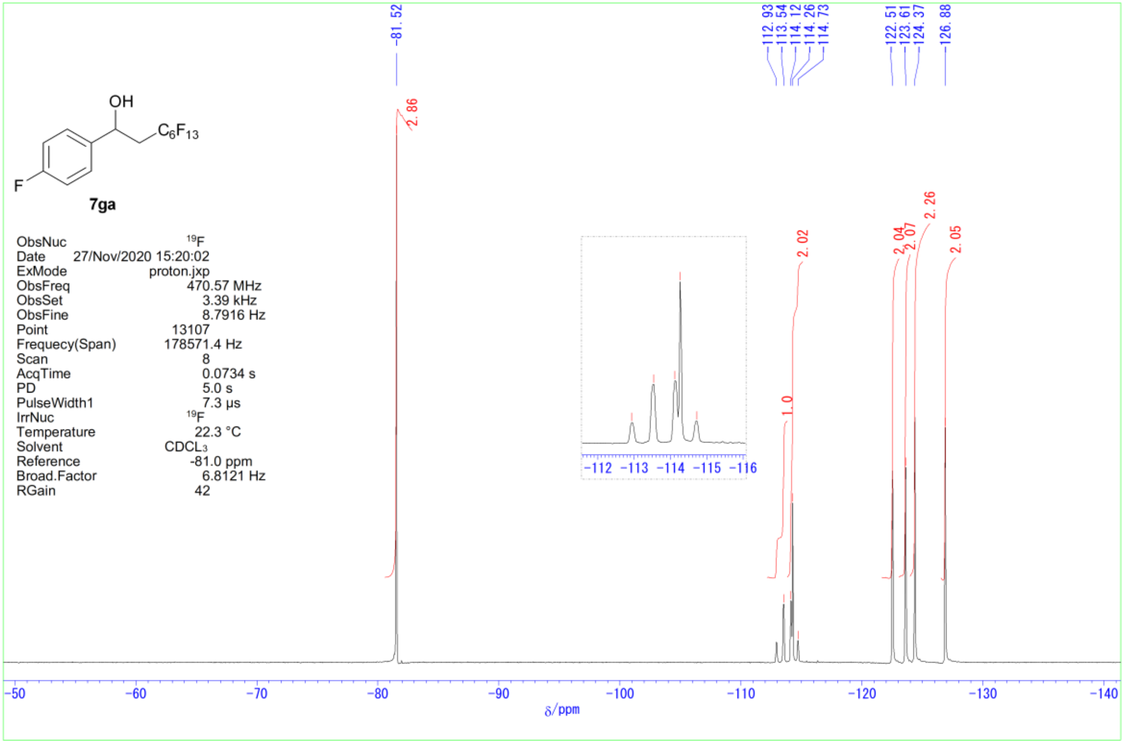

7ha: ^{1}H NMR ($CDCl_3$, 500 MHz)

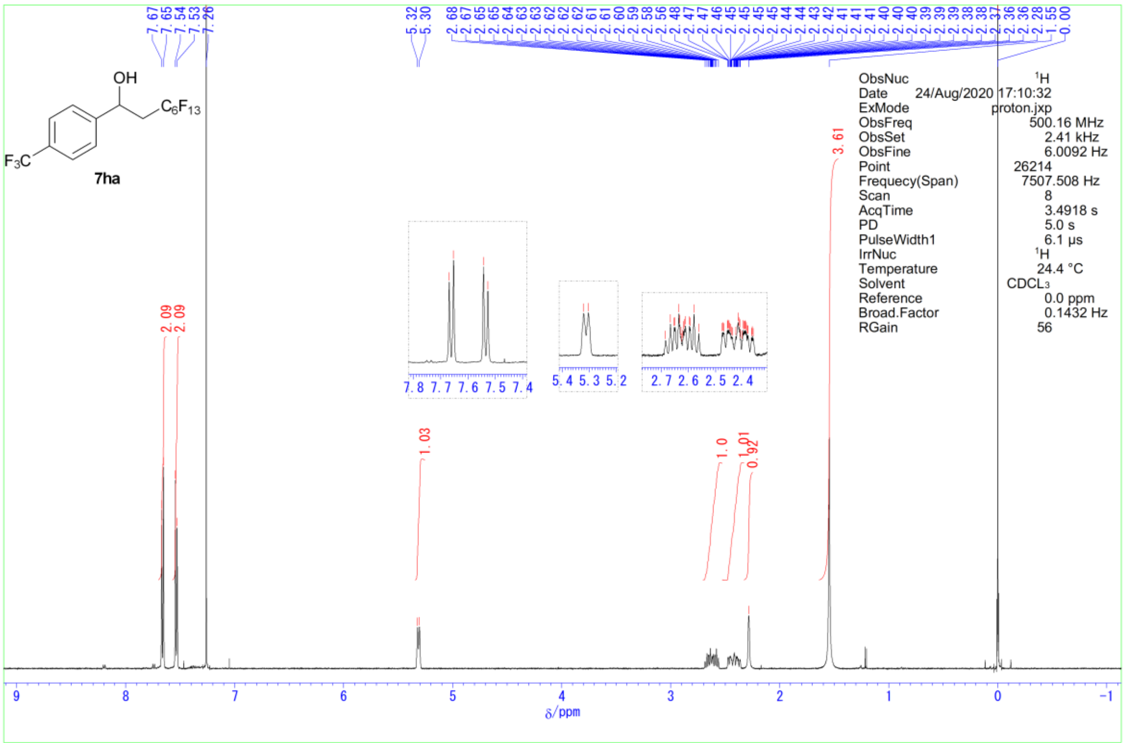

7ha: ^{13}C NMR ($CDCl_3$, 151 MHz)

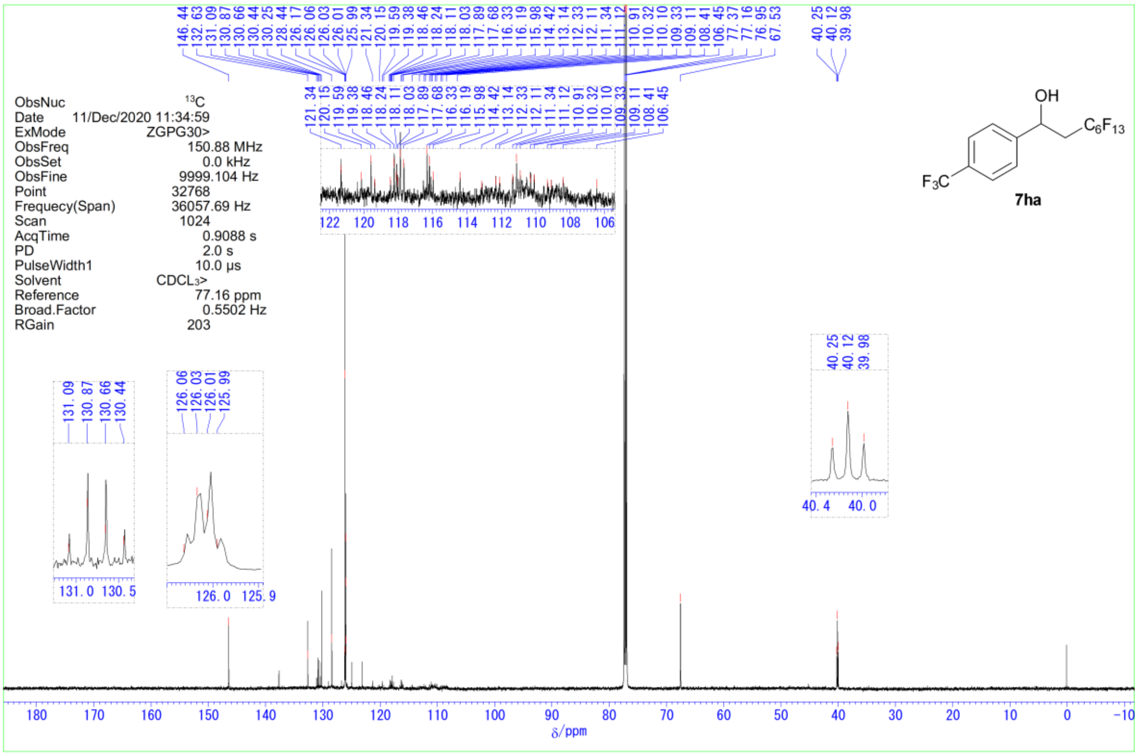

7ha: ^{19}F NMR ($CDCl_3$, 471 MHz)

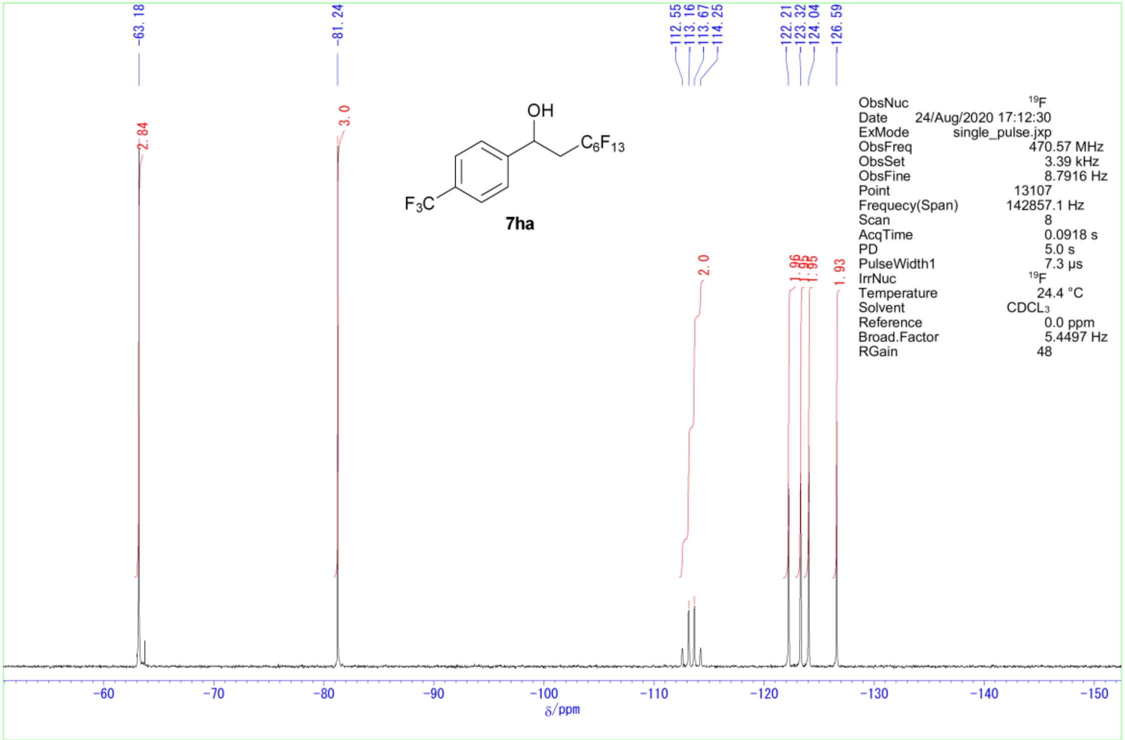

7ia: ^{1}H NMR ($CDCl_3$, 400 MHz)

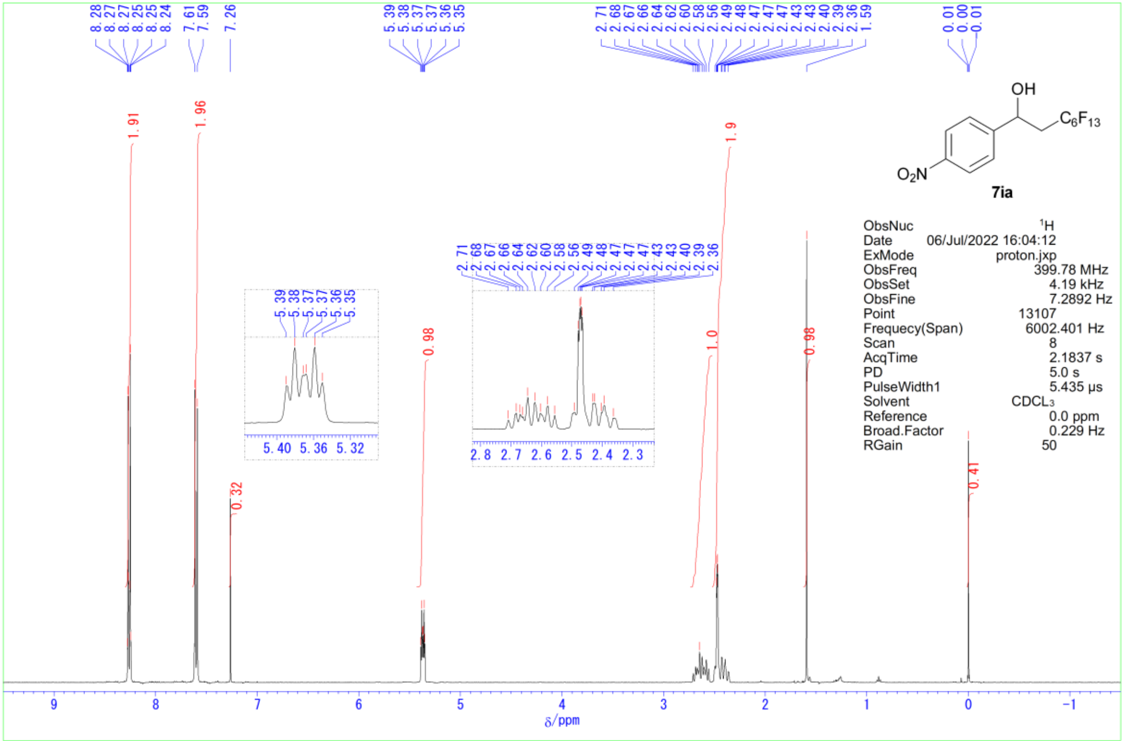

7ia: ^{13}C NMR ($CDCl_3$, 151 MHz)

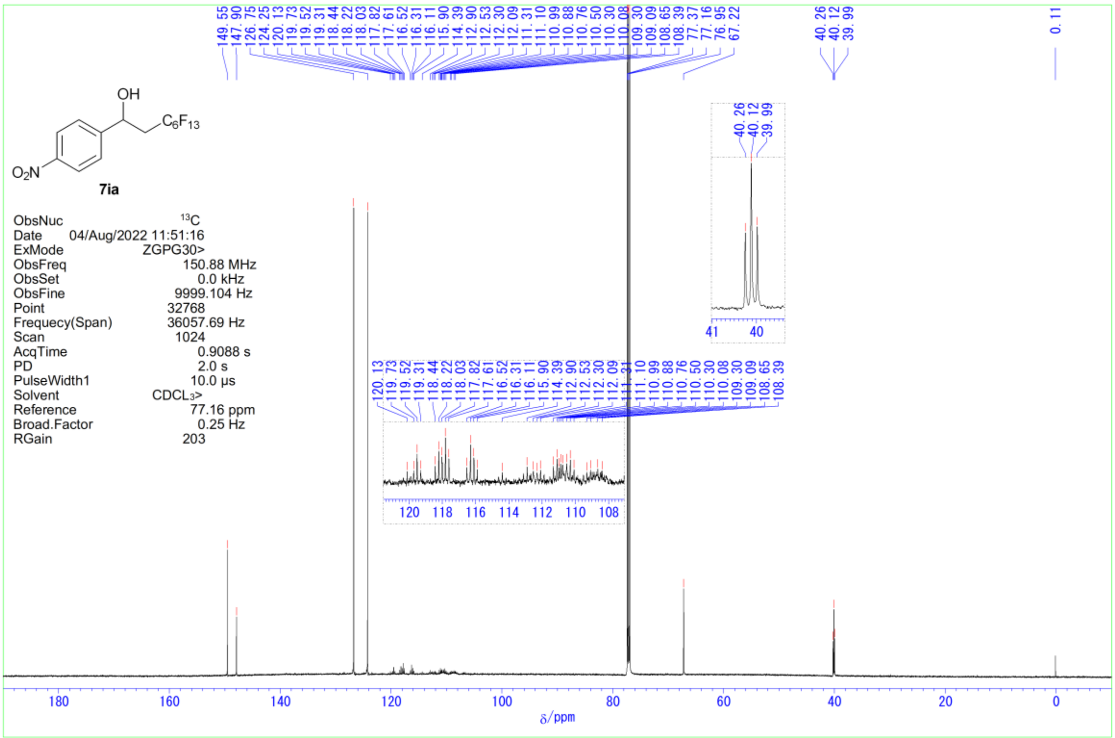

7ia: ^{19}F NMR ($CDCl_3$, 471 MHz)

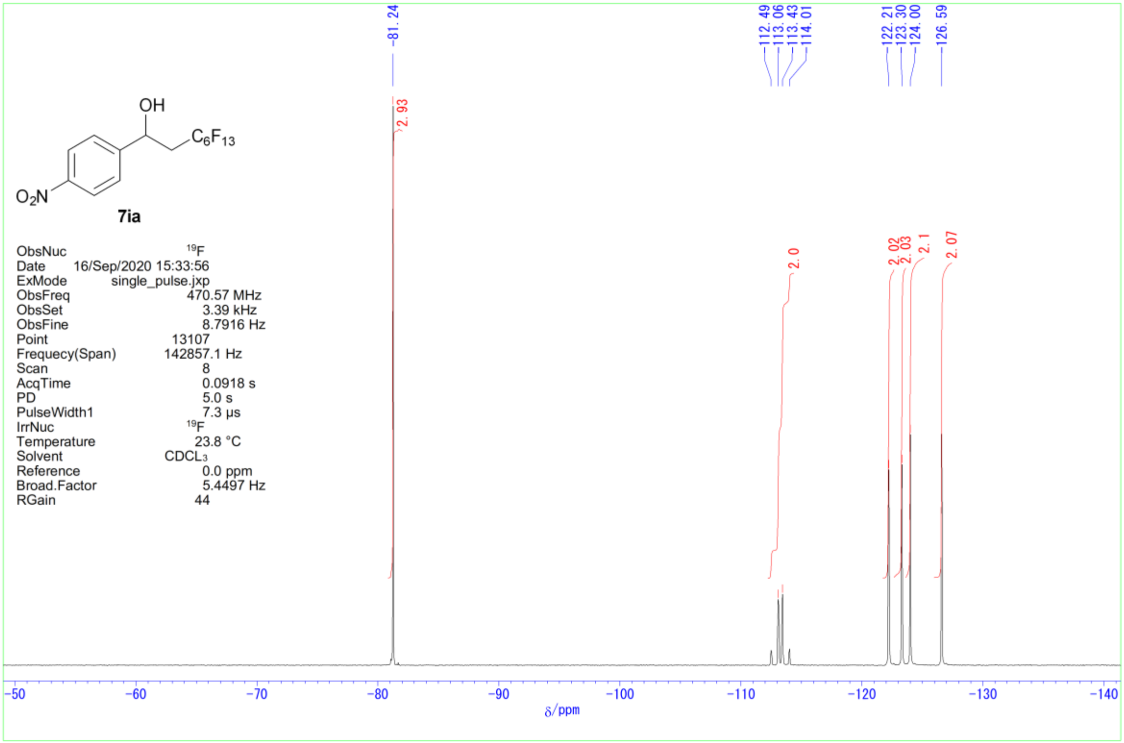

7ja: ^{1}H NMR ($CDCl_3$, 500 MHz)

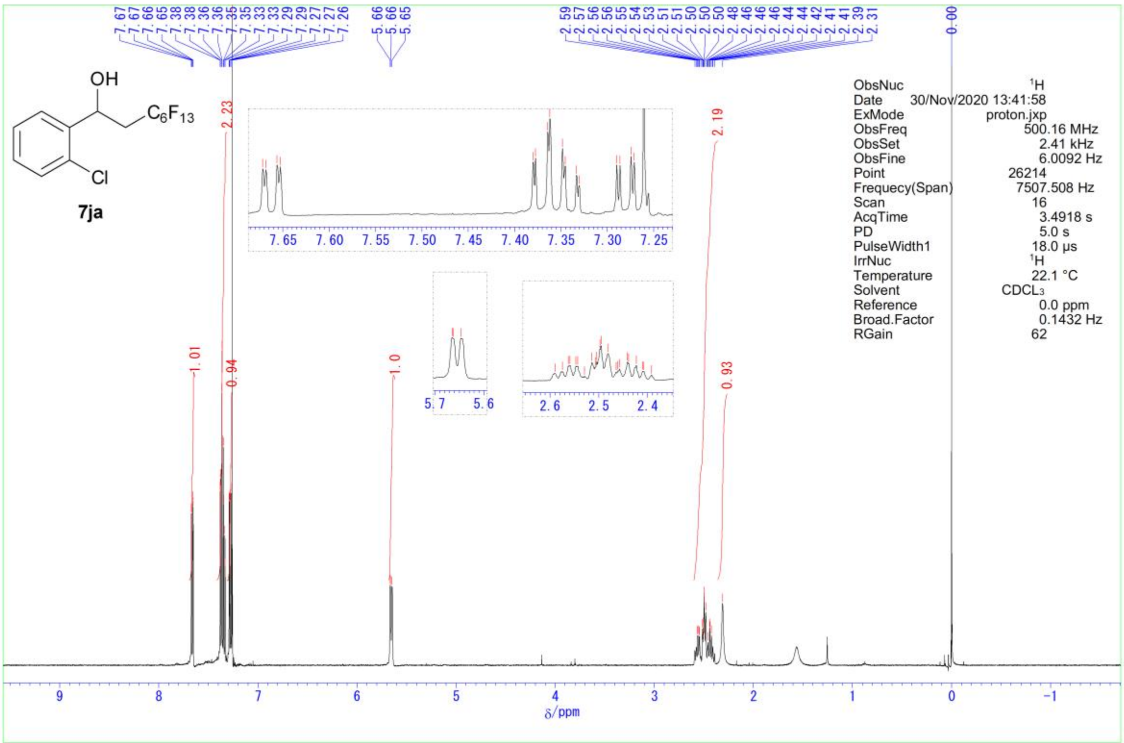

7ja: ^{13}C NMR ($CDCl_3$, 151 MHz)

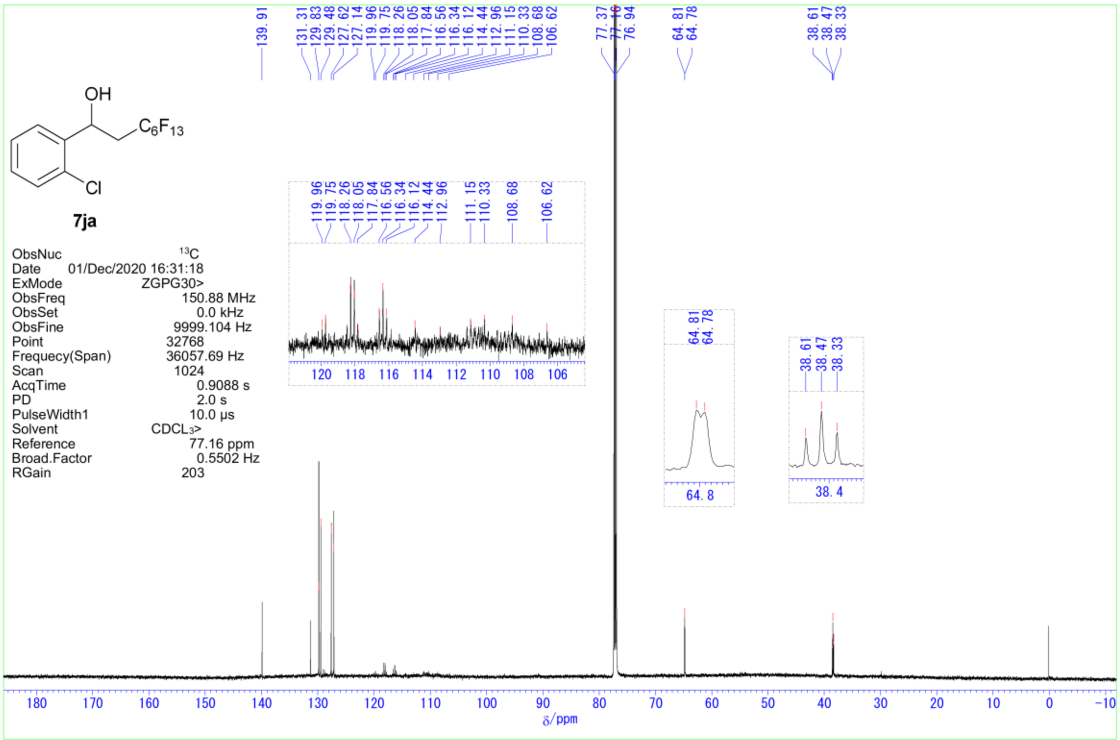

7ja: ^{19}F NMR ($CDCl_3$, 471 MHz)

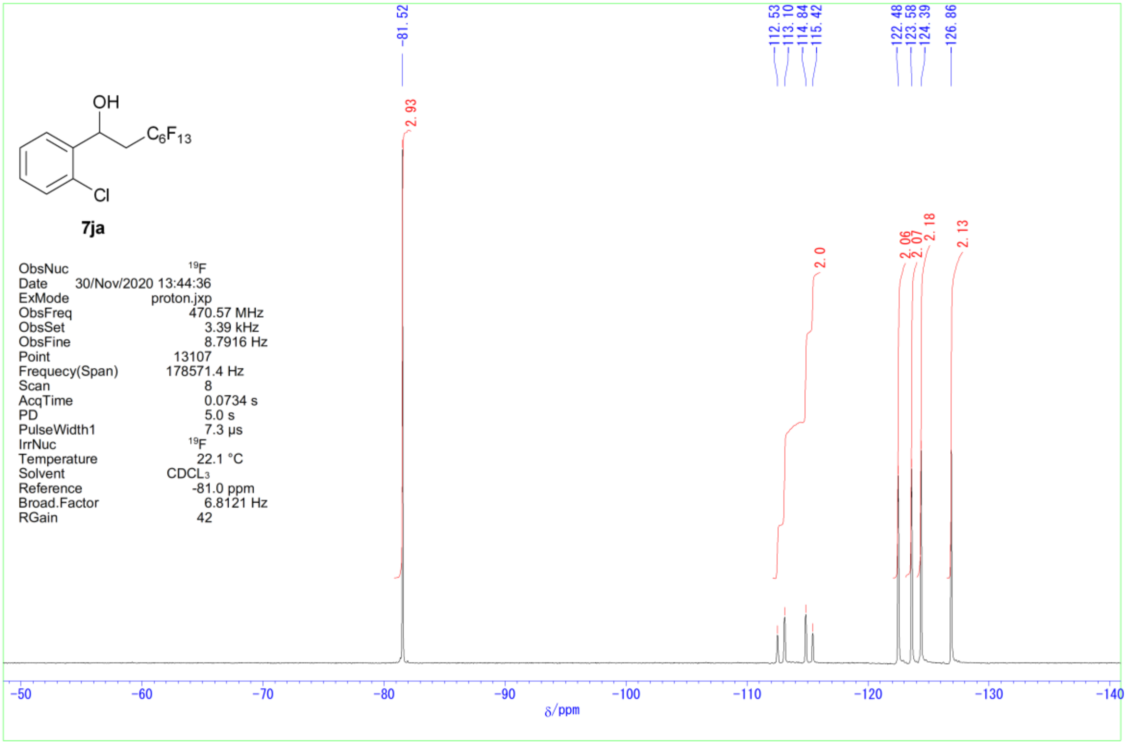

7ka: ^{1}H NMR ($CDCl_3$, 500 MHz)

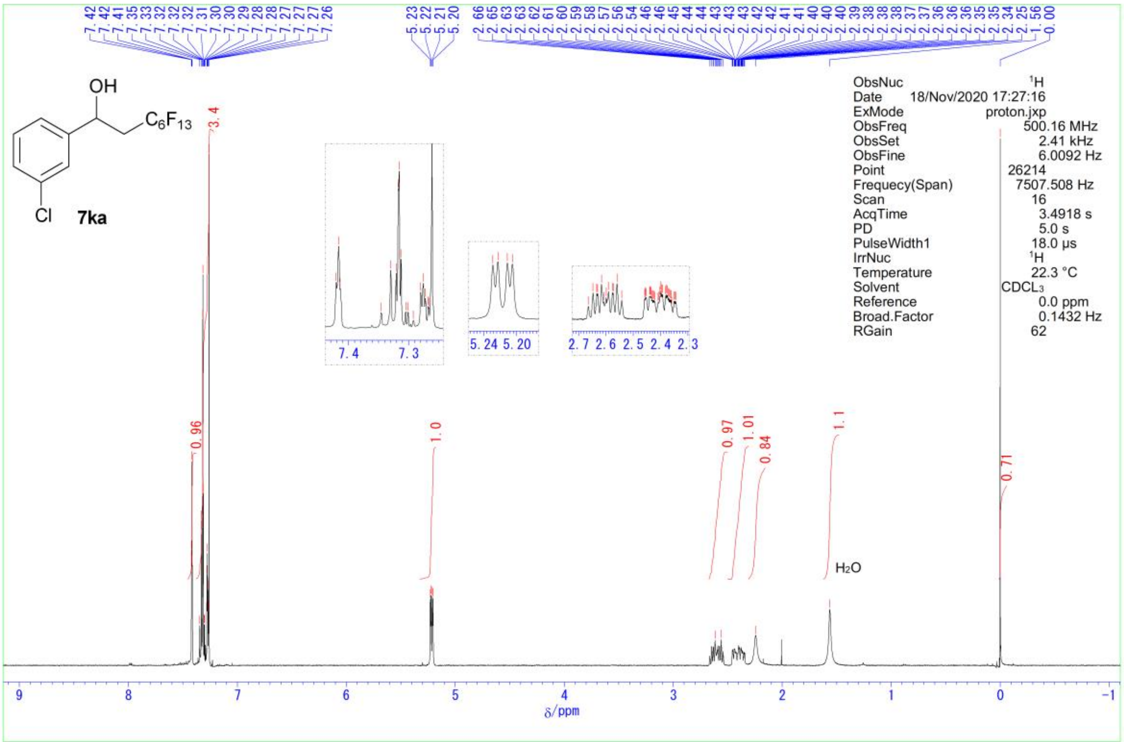

7ka: ^{13}C NMR ($CDCl_3$, 151 MHz)

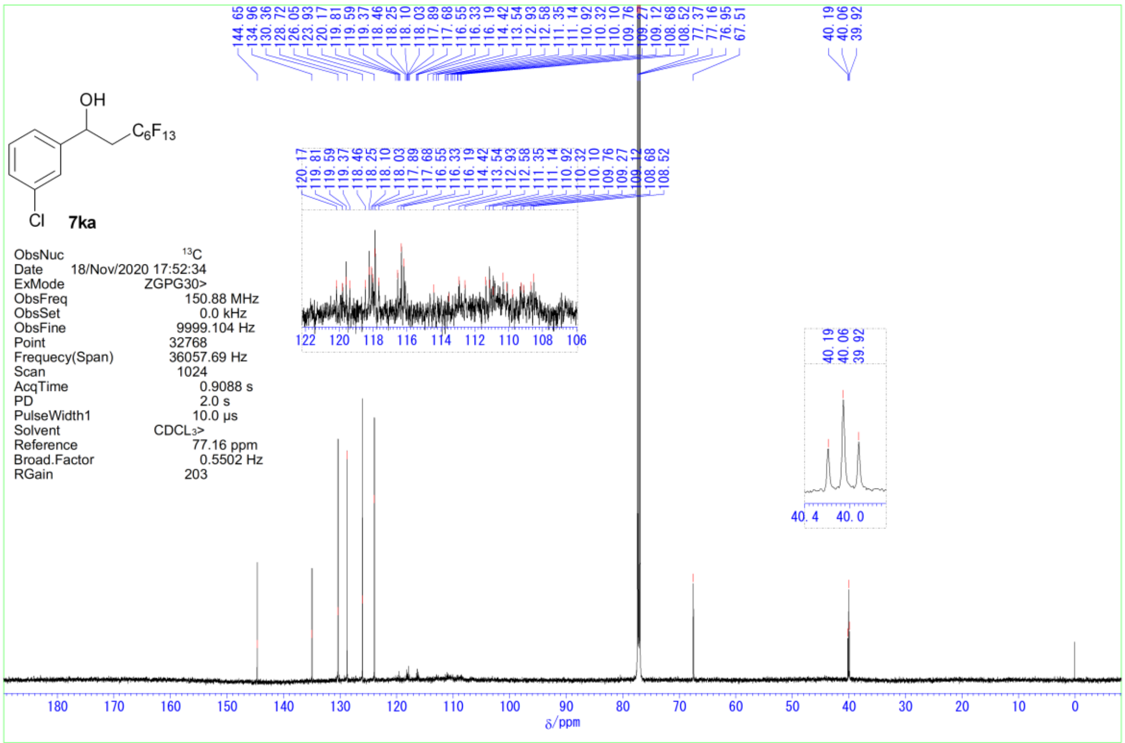

7ka: ^{19}F NMR ($CDCl_3$, 471 MHz)

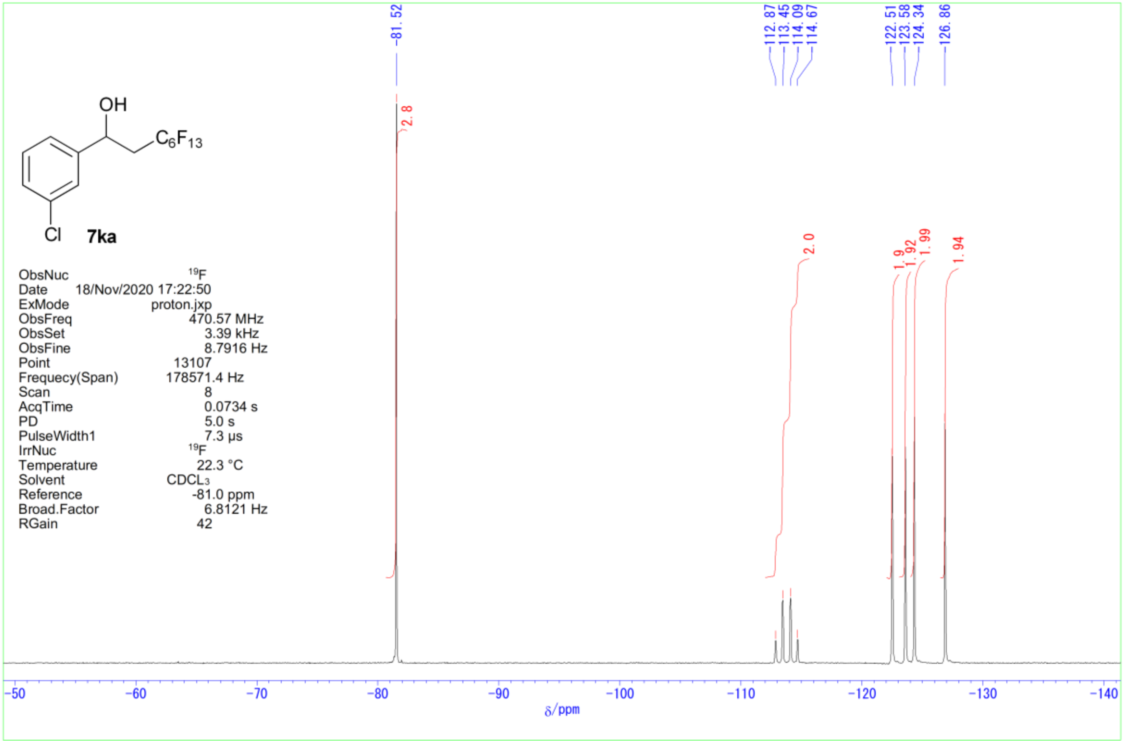

7la: ^{1}H NMR ($CDCl_3$, 500 MHz)

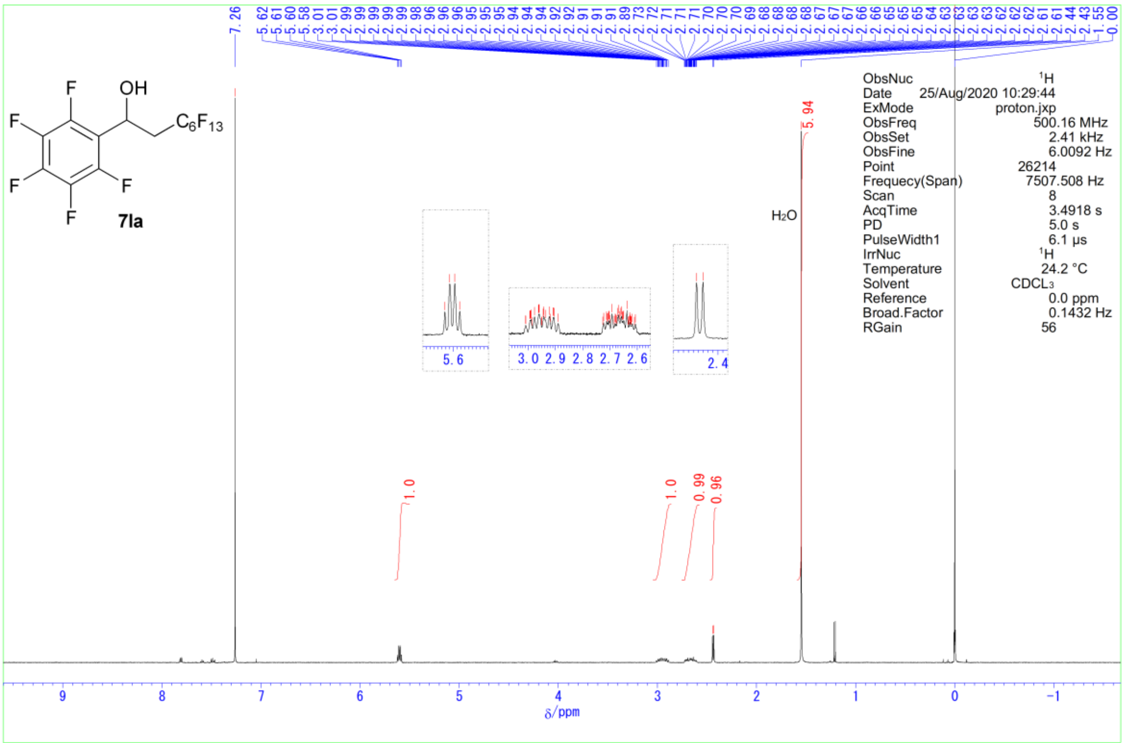

7la: ^{13}C NMR (CDCl$_3$, 151 MHz)

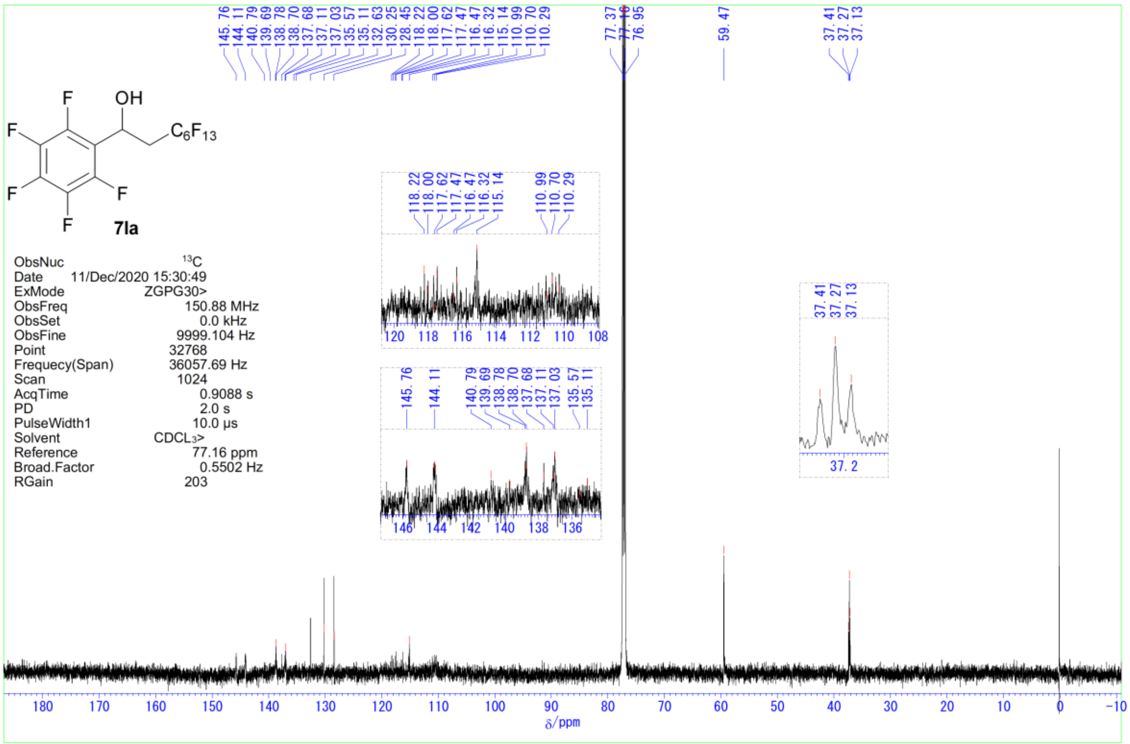

7la: ^{19}F NMR (CDCl$_3$, 471 MHz)

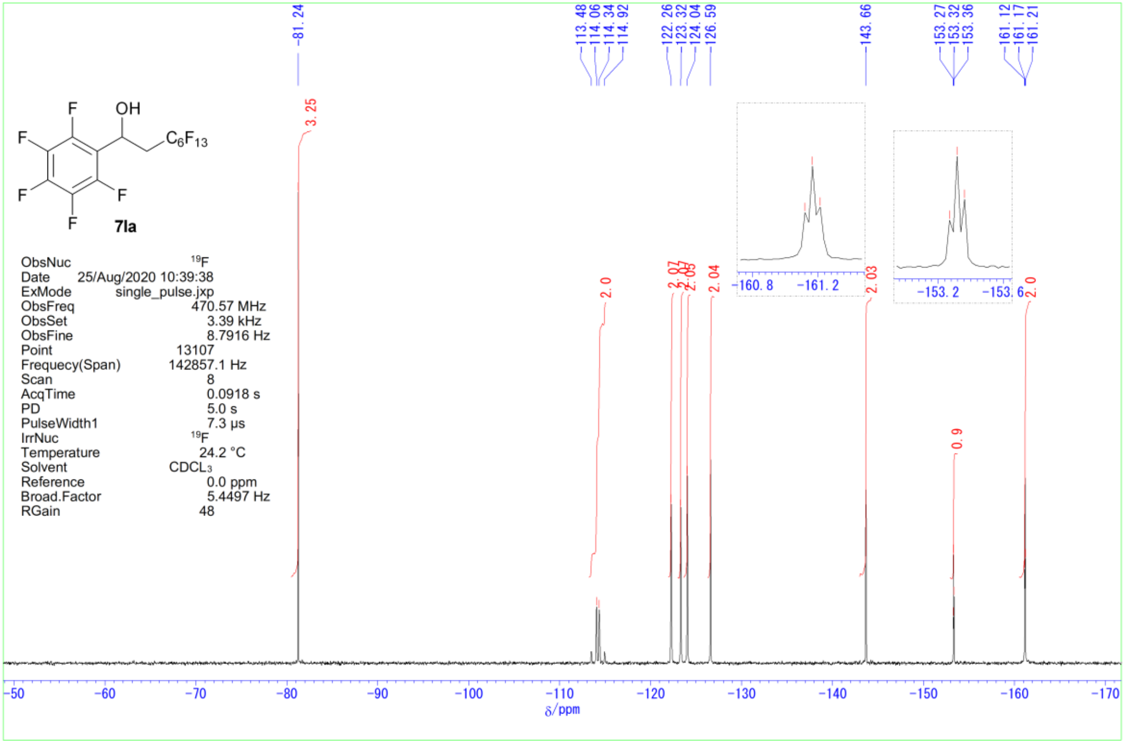

7ma: ^{1}H NMR ($CDCl_3$, 400 MHz)

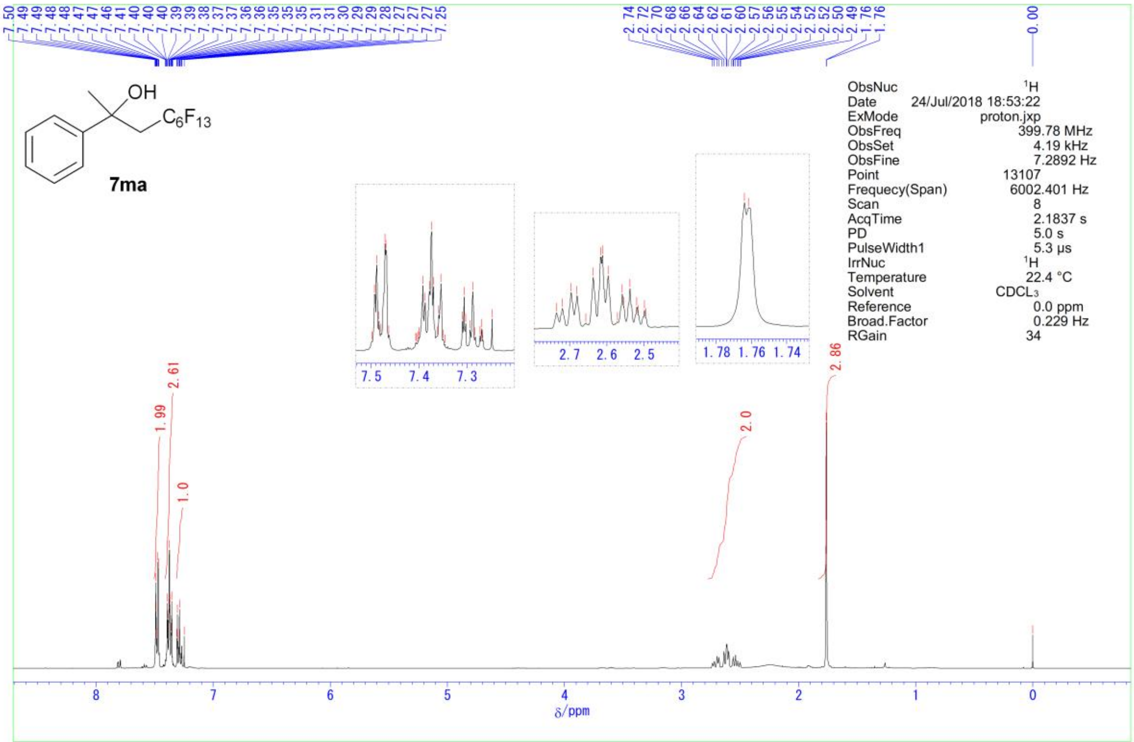

7ma: ^{13}C NMR ($CDCl_3$, 126 MHz)

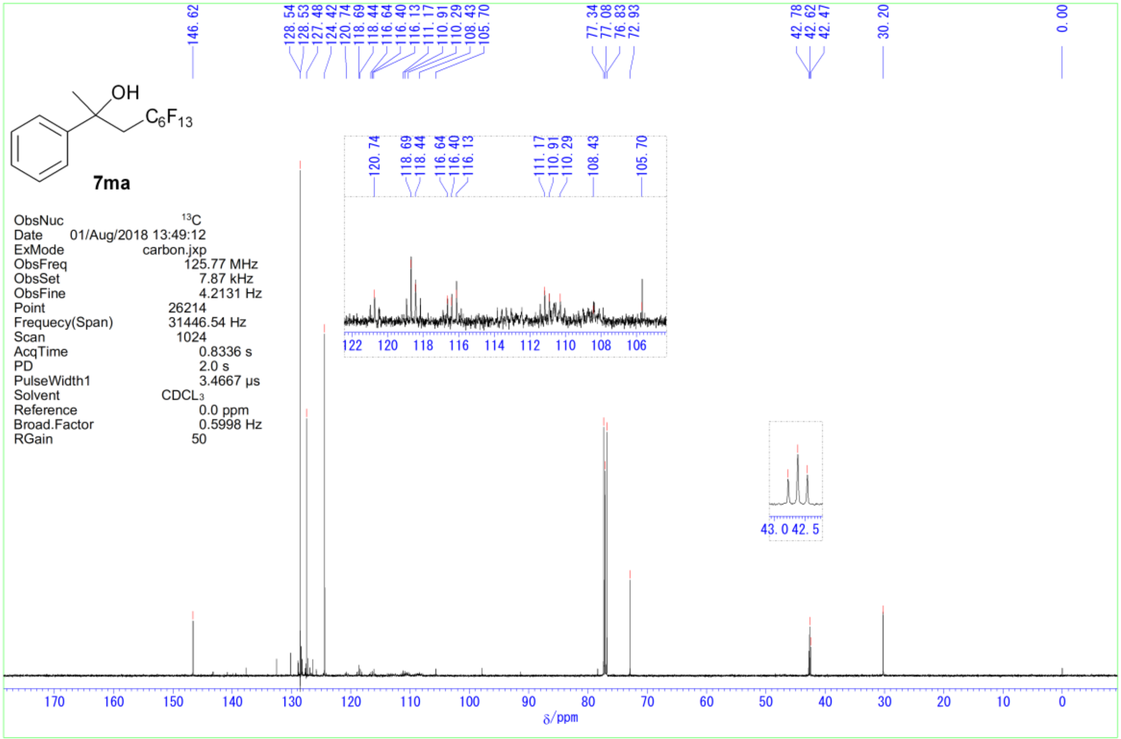

7ma: ^{19}F NMR (CDCl$_3$, 376 MHz)

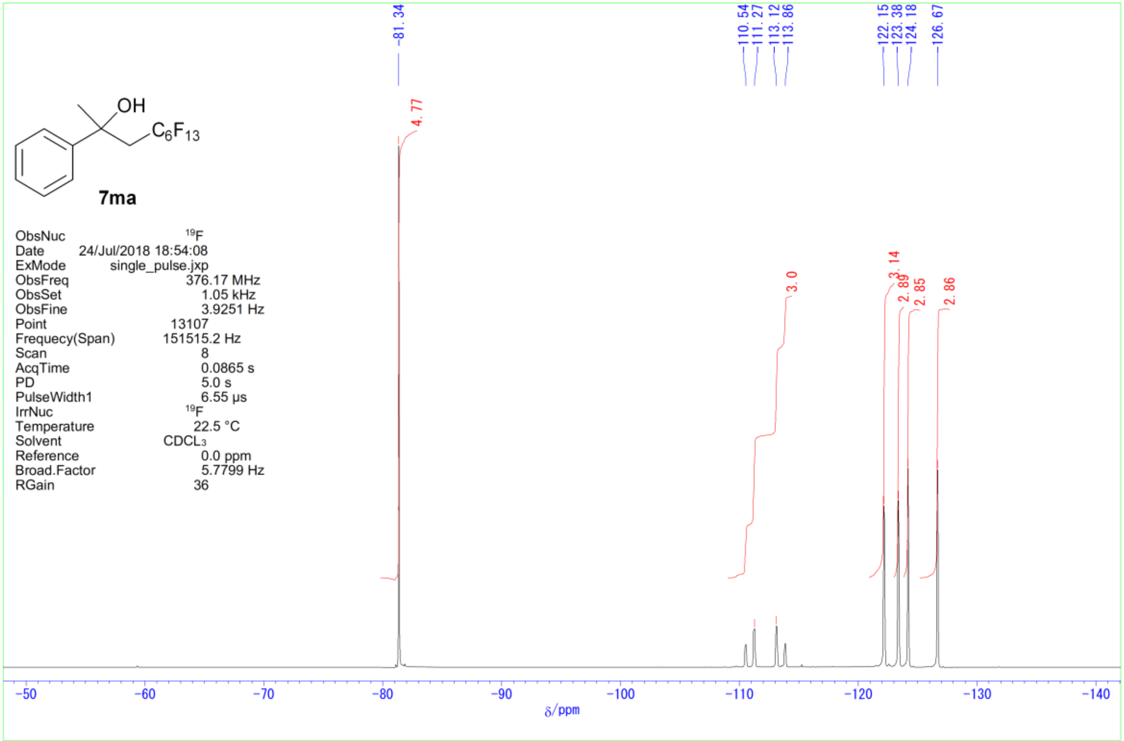

7na: ^{1}H NMR (CDCl$_3$, 400 MHz)

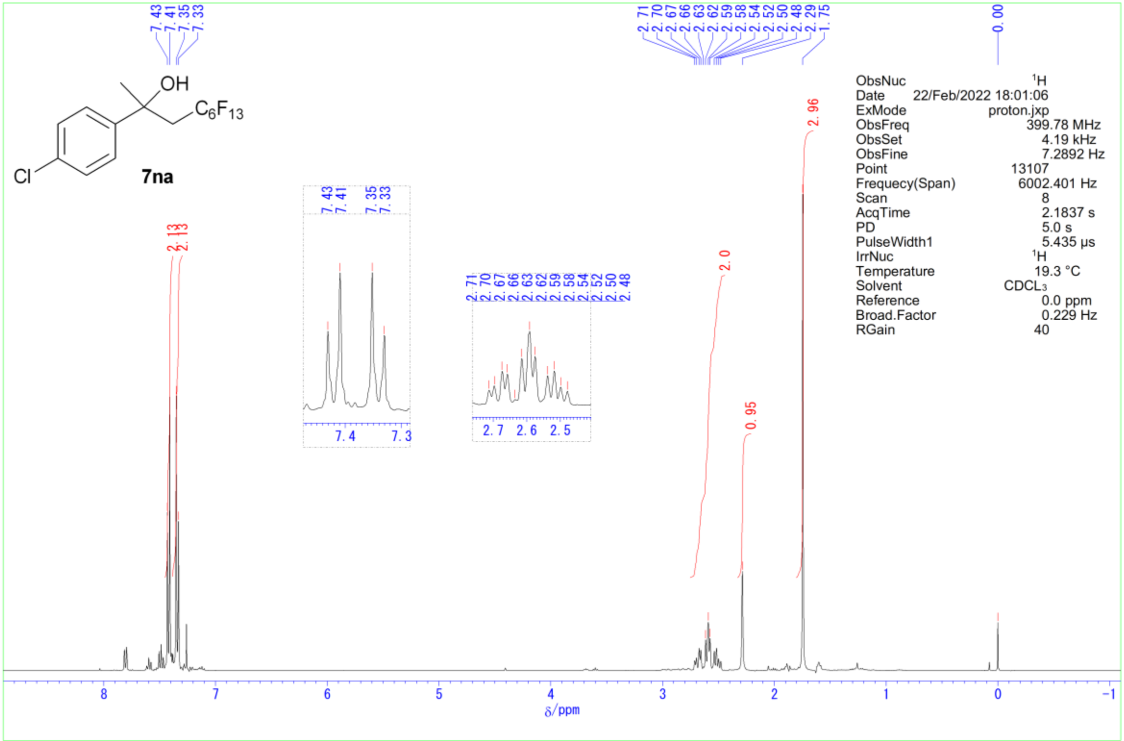

7na: ^{13}C NMR ($CDCl_3$, 151 MHz)

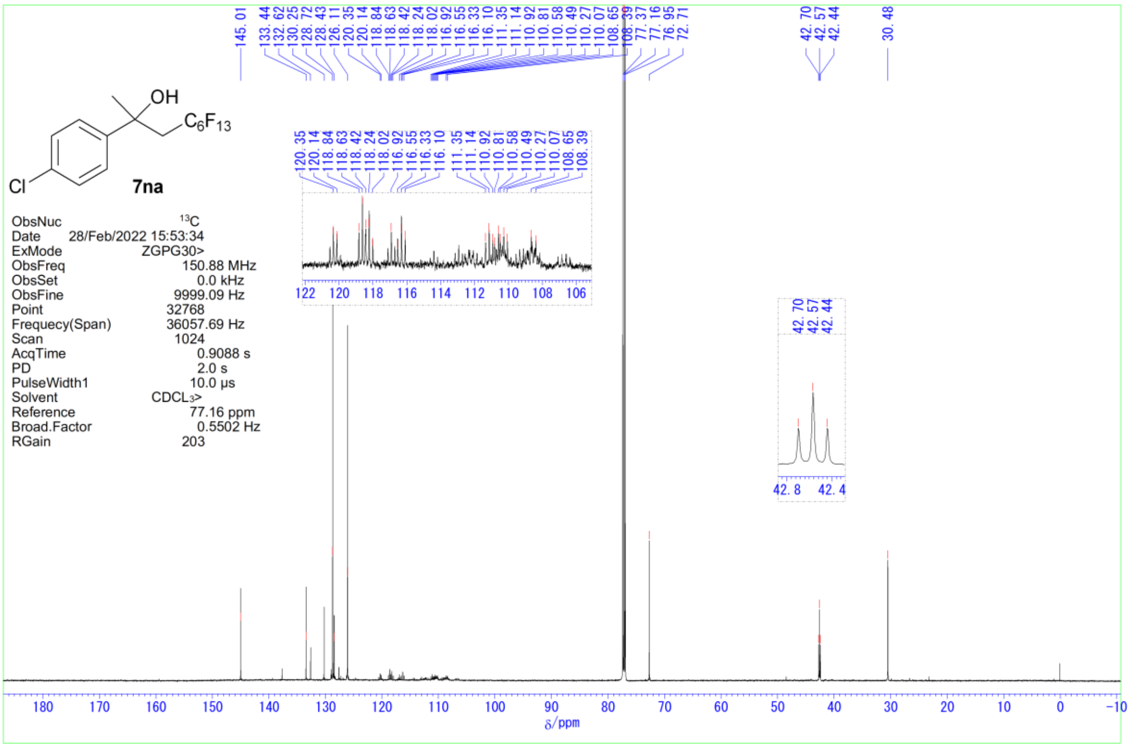

7na: ^{19}F NMR ($CDCl_3$, 376 MHz)

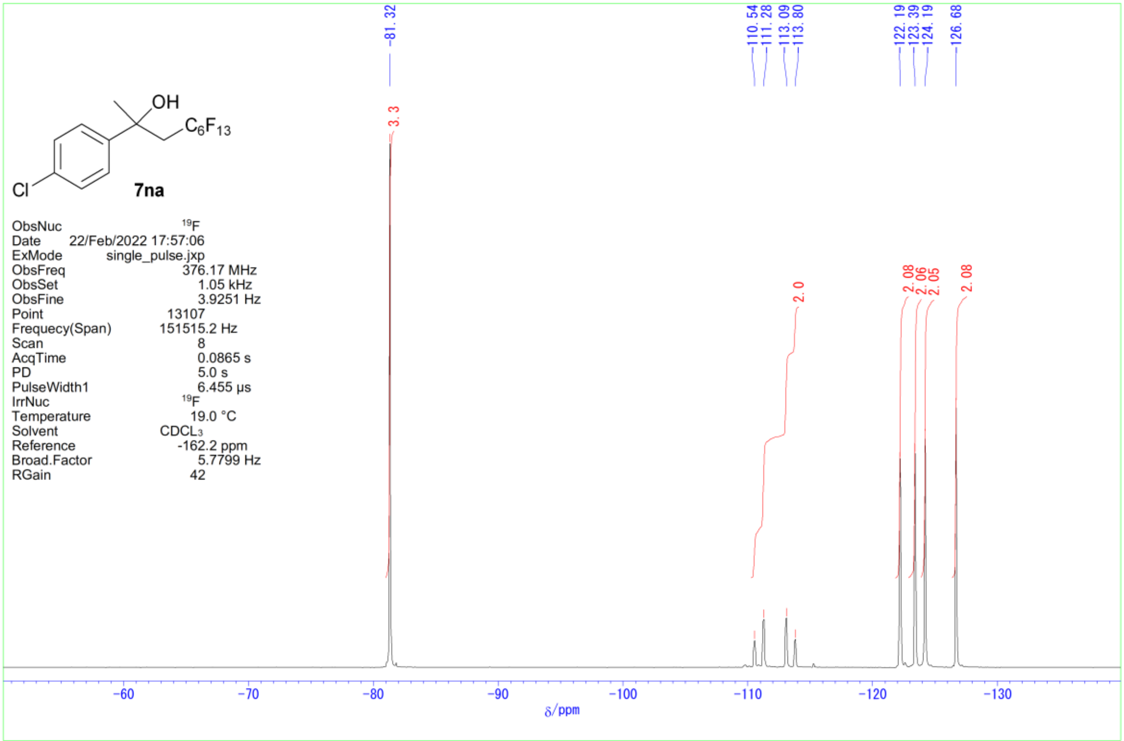

7oa: ^{1}H NMR ($CDCl_3$, 400 MHz)

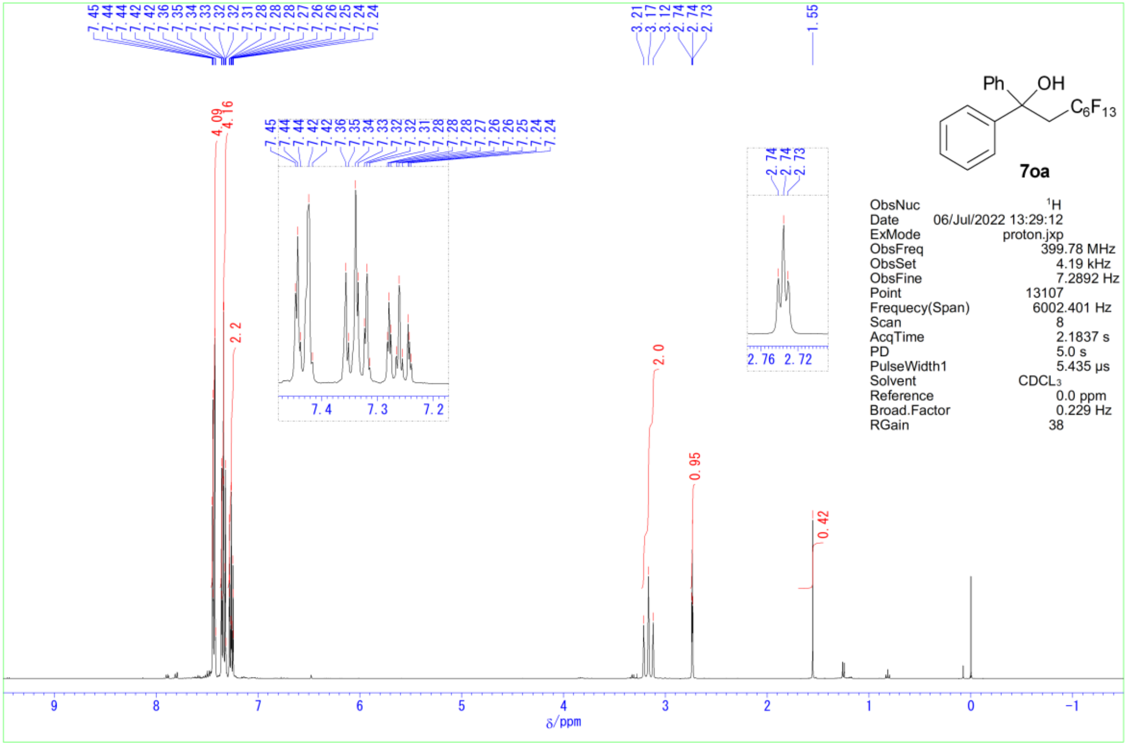

7oa: ^{13}C NMR ($CDCl_3$, 151 MHz)

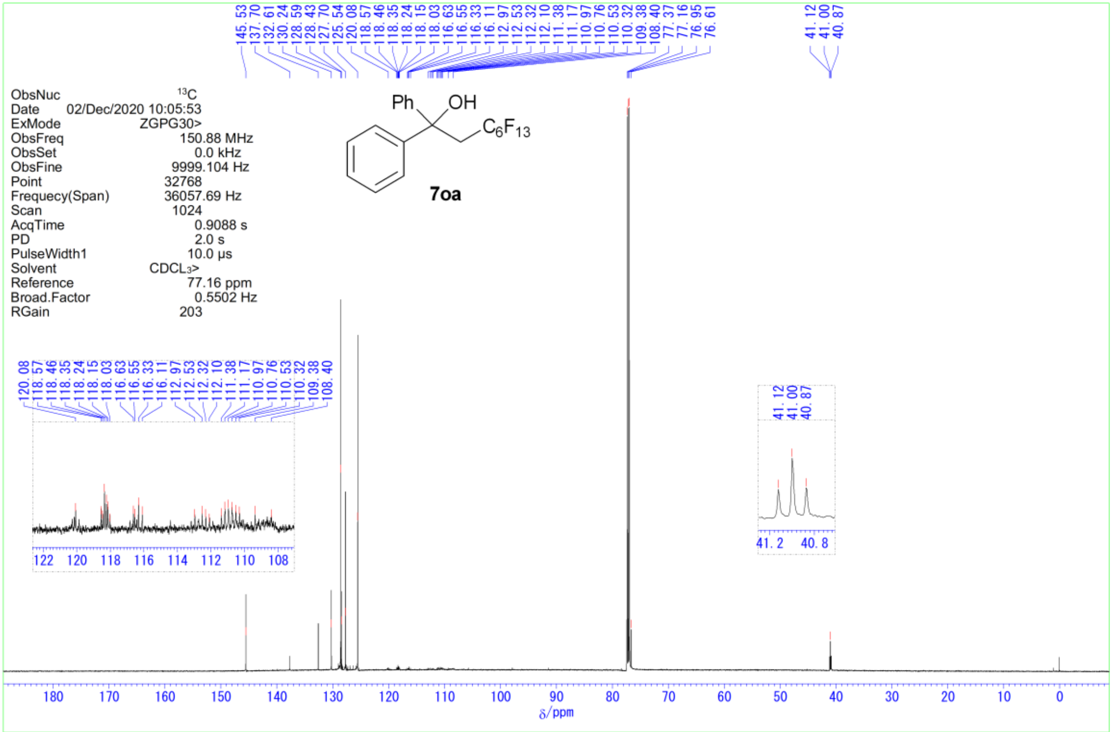

7oa: ^{19}F NMR ($CDCl_3$, 376 MHz)

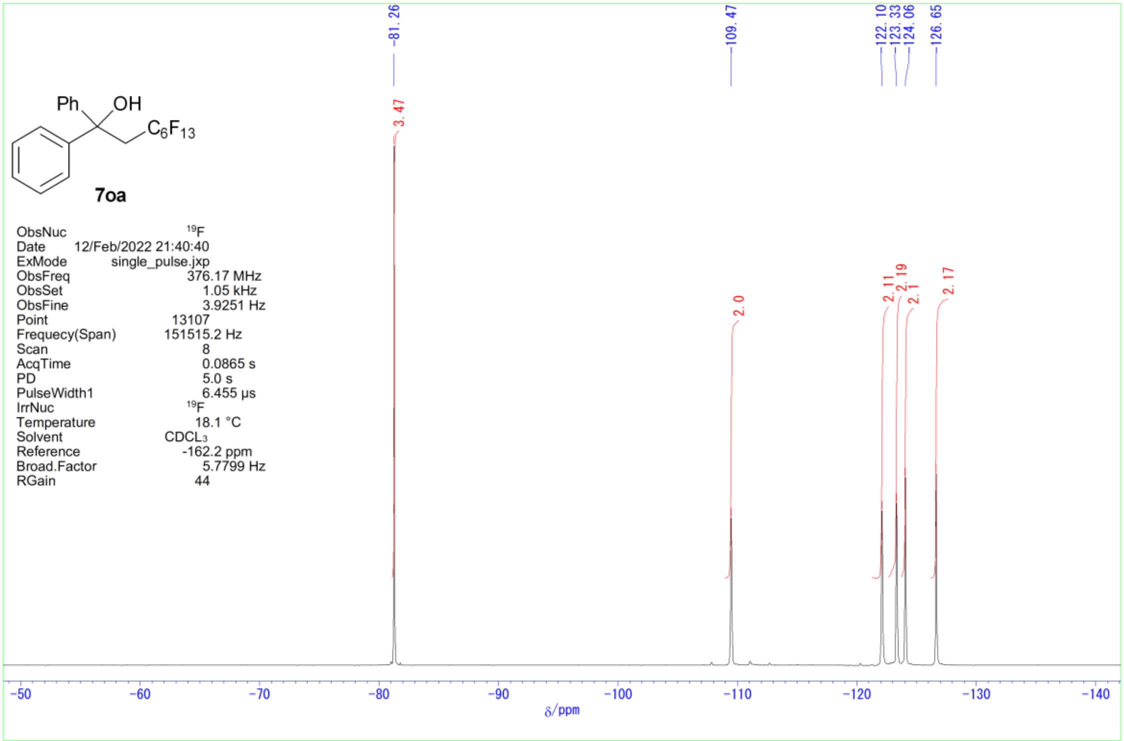

7pa: ^{1}H NMR ($CDCl_3$, 400 MHz)

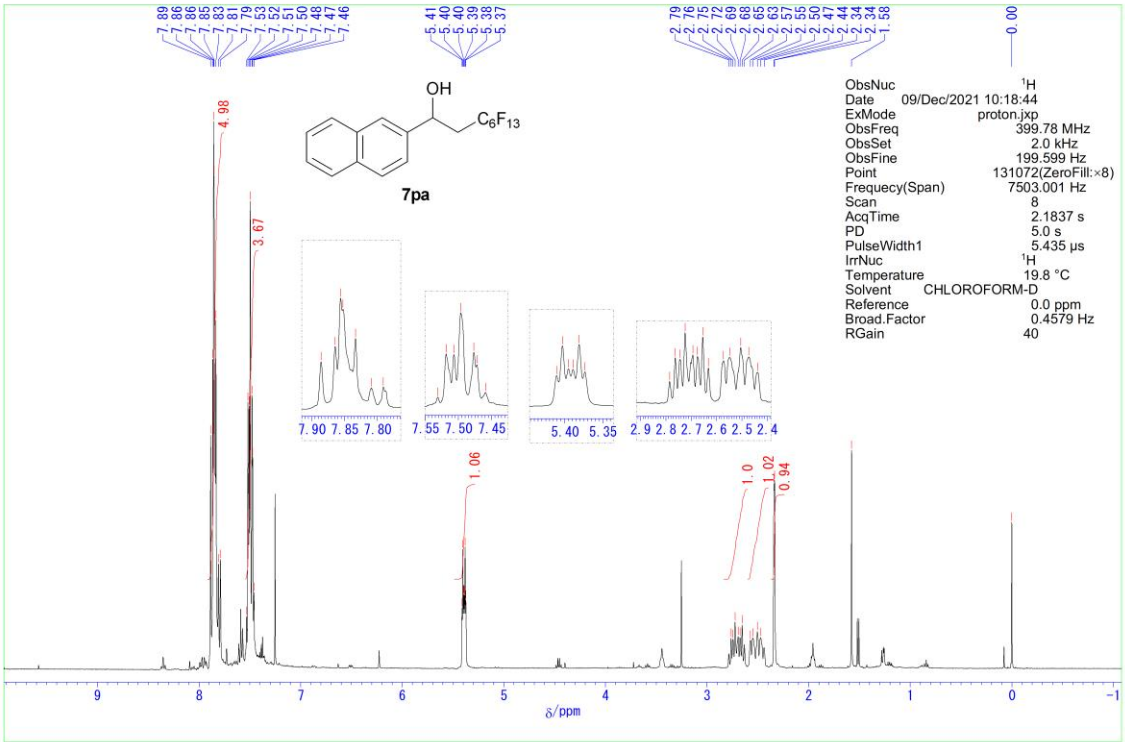

7pa: ^{13}C NMR ($CDCl_3$, 151 MHz)

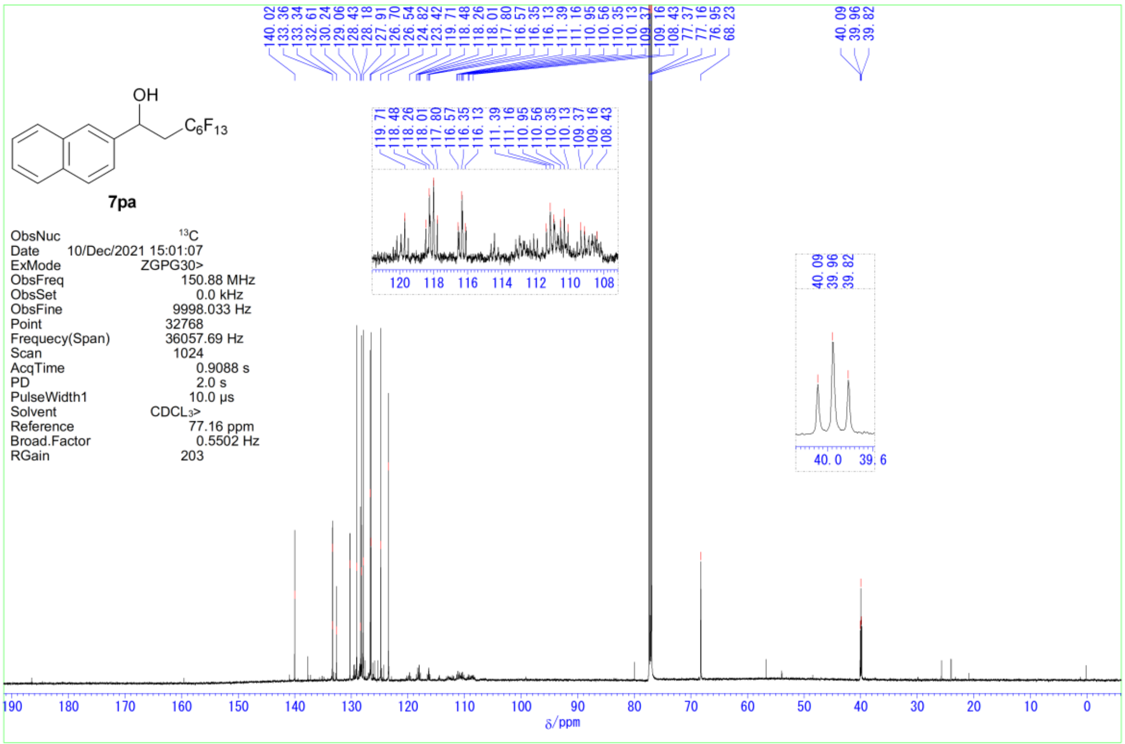

7pa: ^{19}F NMR ($CDCl_3$, 376 MHz)

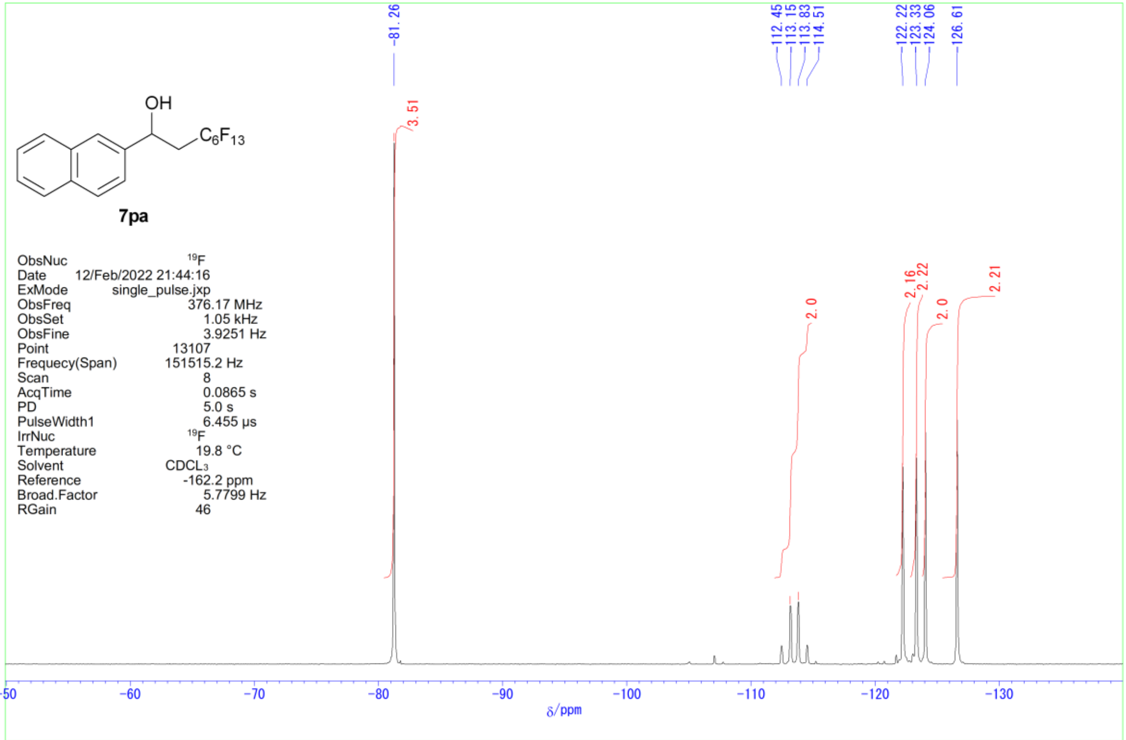

7qa: ^{1}H NMR ($CDCl_3$, 500 MHz)

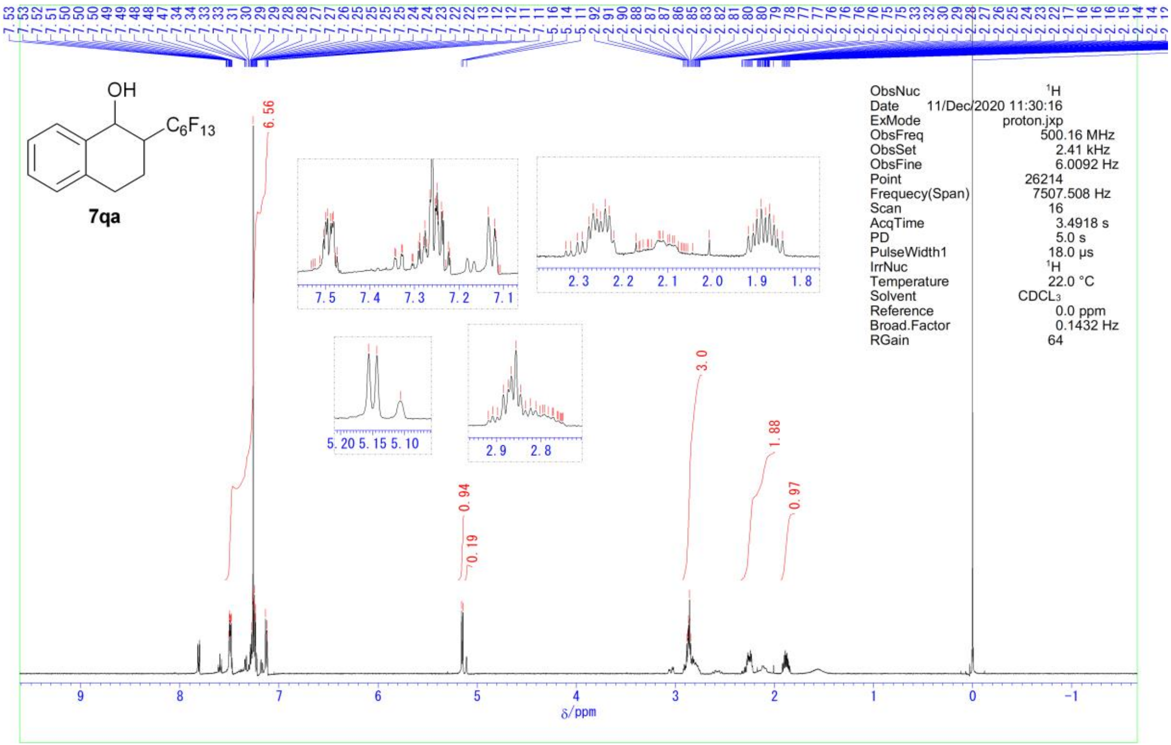

7qa: ^{13}C NMR ($CDCl_3$, 151 MHz)

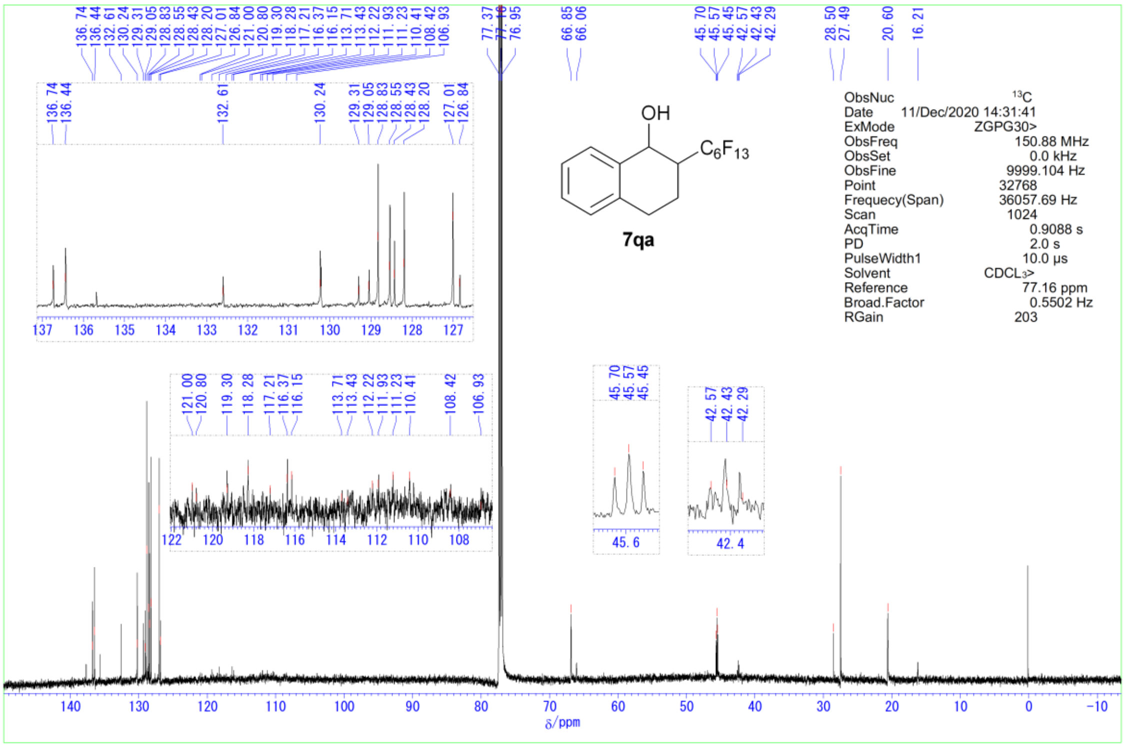

7qa: ^{19}F NMR ($CDCl_3$, 471 MHz)

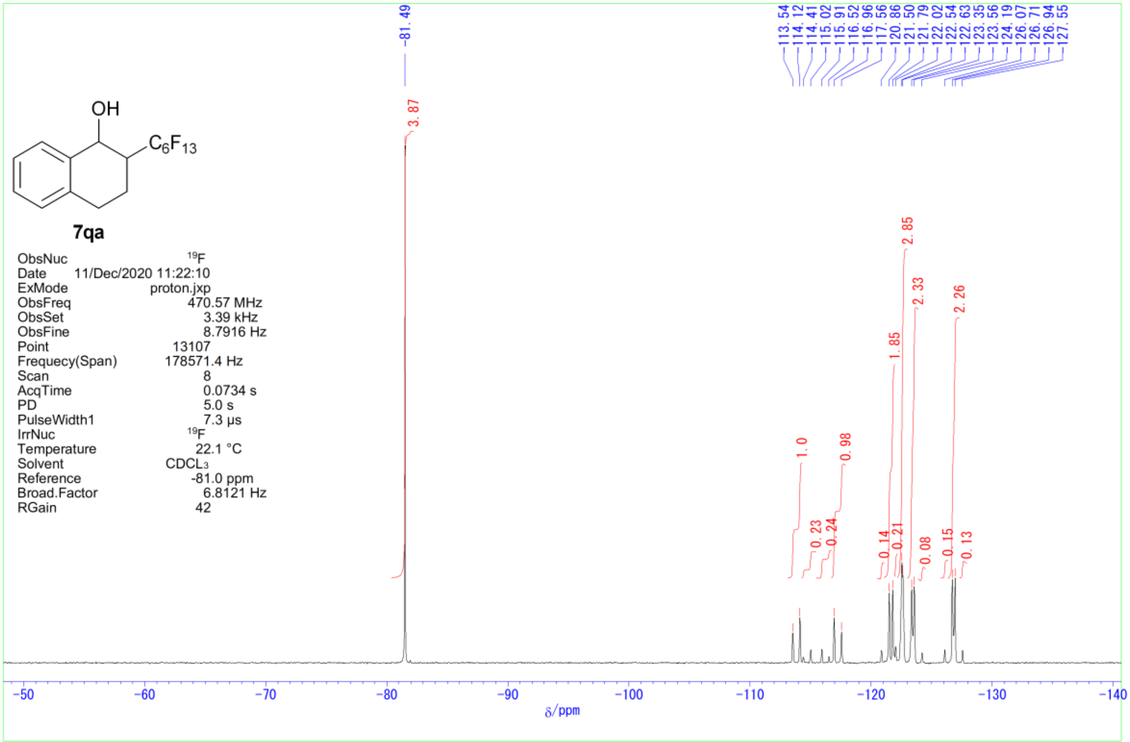

7ra: ^{1}H NMR ($CDCl_3$, 500 MHz)

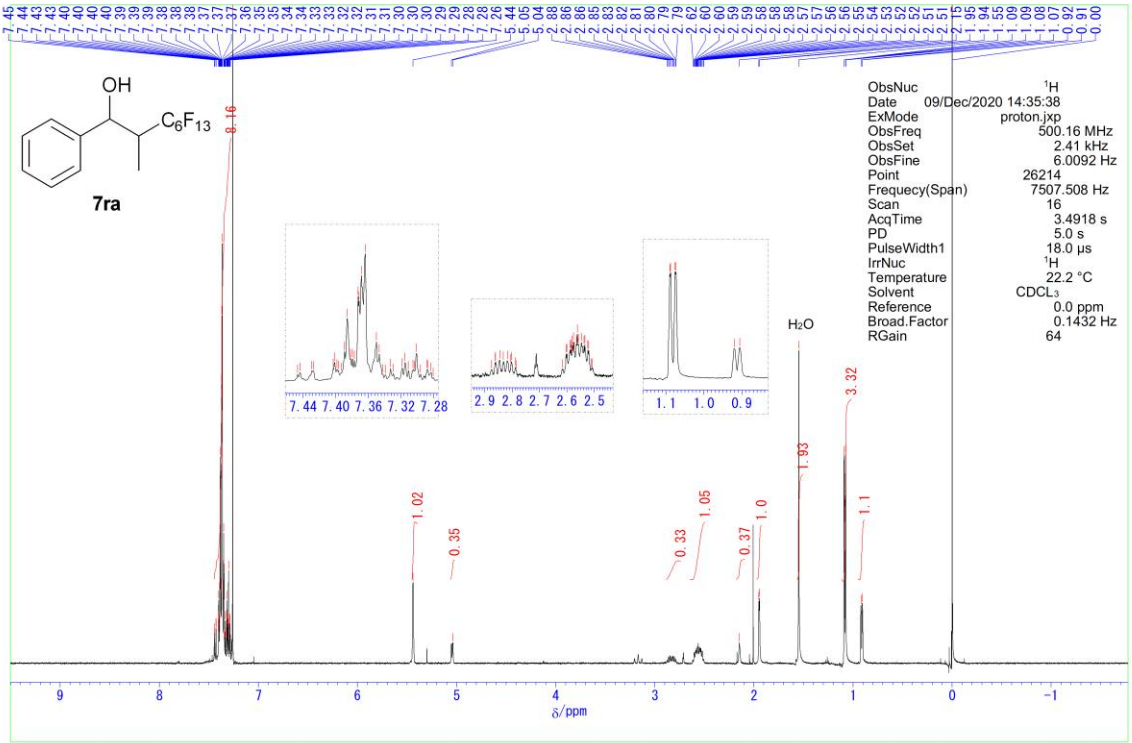

7ra: ^{13}C NMR ($CDCl_3$, 151 MHz)

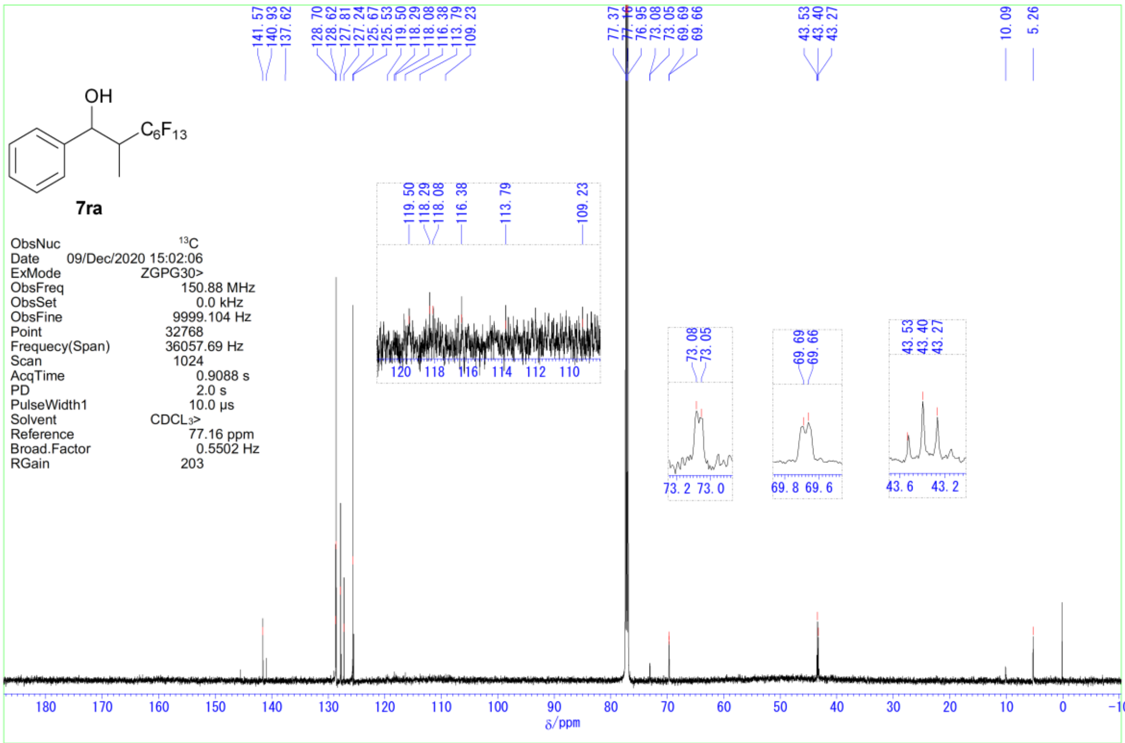

7ra: ^{19}F NMR ($CDCl_3$, 471 MHz)

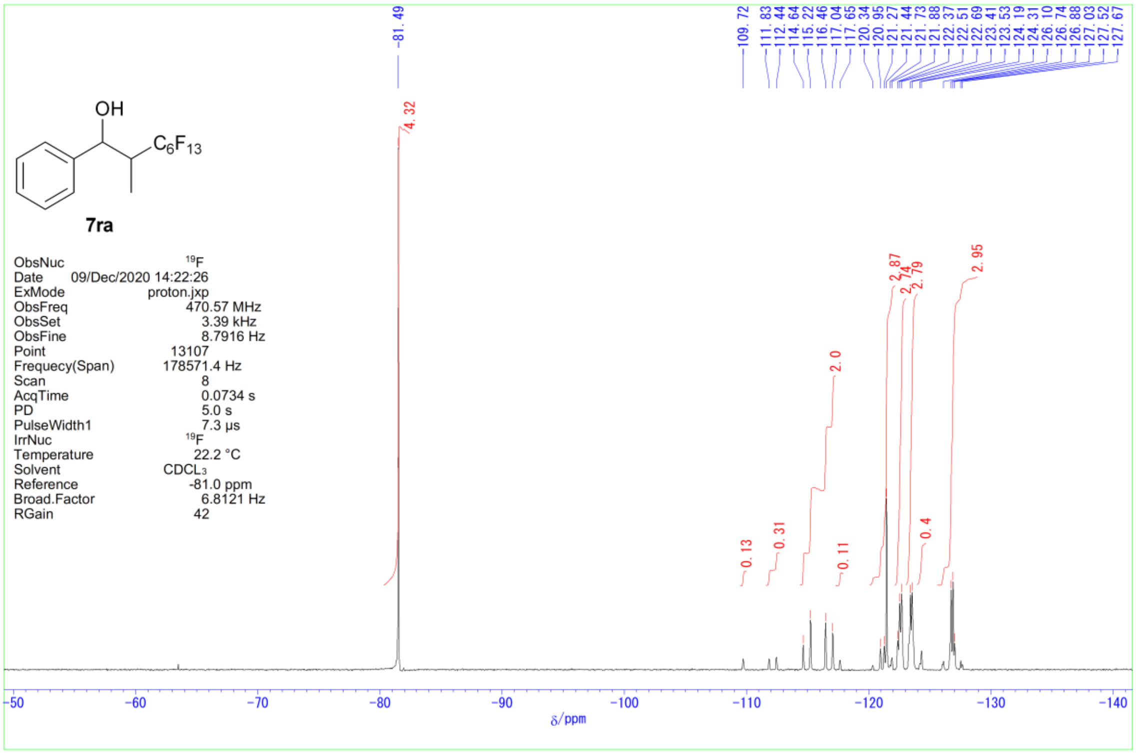

Supplement: RA-012-D2RA06679C-s001 [file RA-012-D2RA06679C-s001.pdf]
